# Orthogonal photoswitching of heterobivalent azobenzene glycoclusters: the effect of glycoligand orientation in bacterial adhesion

**DOI:** 10.3762/bjoc.21.57

**Published:** 2025-04-08

**Authors:** Leon M Friedrich, Thisbe K Lindhorst

**Affiliations:** 1 Otto Diels Institute of Organic Chemistry, Christiana Albertina University of Kiel, Otto-Hahn-Platz 3–4, 24118 Kiel, Germany

**Keywords:** azobenzene glycoconjugates, carbohydrate recognition, docking, FimH, orthogonal photoswitching

## Abstract

Carbohydrate recognition is fundamental to a plethora of cellular processes and hence the elucidation of the structural determinants of the recognition process is a prerequisite for understanding and manipulating carbohydrate–protein interactions, such as in the inhibition of carbohydrate-specific bacterial adhesion. For receptor binding, glycoligands have to be properly oriented in three-dimensional space and additionally, secondary interactions exerted by multivalent glycoligands have an effect on affinity. A recently introduced orthogonally photoswitchable heterobivalent azobenzene Glc/Man glycocluster was utilized to examine these aspects of carbohydrate recognition in a bacterial adhesion–inhibition assay. The measured results were systematically contextualized employing new reference compounds such as the respective homobivalent Man/Man glycocluster. An in-depth study comprising the analysis of the photochromic properties and the potential as inhibitors of bacterial adhesion of the synthetic glycophotoswitches in their different isomeric states led to new insights into the role of ligand orientation in carbohydrate recognition. The experimental results were underpinned by molecular modeling.

## Introduction

Carbohydrate–protein interactions are fundamental in cell biology, such as in cell–cell interactions, immune cell trafficking or bacterial adhesion, and therefore carbohydrate recognition is subject of intensive research. In particular, a plethora of synthetic glycoconjugates have been designed and tested to investigate the details of carbohydrate recognition [1–7]. It has turned out that carbohydrate–lectin interactions are orchestrated by a multitude of structural parameters. In addition to the configurational characteristics of specific carbohydrate building blocks, homo- and heteromultivalency effects are crucial [8–12], but also many other aspects of glycoligand presentation govern carbohydrate recognition.

Interestingly, even a subtle structural variation in heterobivalent glycoclusters can result in unexpected differences in lectin binding due to diverse interactions with primary and secondary carbohydrate binding sites of the protein [13]. For example, when an α-ᴅ-mannopyranosyl (Man) and a β-ᴅ-glucopyranosyl (Glc) unit were conjugated such that the relative orientation of the two sugar portions is varied – be it on enantiomeric or regioisomeric scaffold molecules, respectively [14–15], – differing inhibitory properties in carbohydrate-specific bacterial adhesion resulted.

To further investigate the effect of relative ligand orientation in carbohydrate recognition, we and others have utilized photoswitchable glycoconjugates [16–19]. Specifically, the reversible *E*/*Z* isomerization of the azo group in azobenzene glycosides is suited to control the spatial presentation of glycoligands and, for example, switch carbohydrate-specific bacterial adhesion on and off [20–23]. Indeed, glycoazobenzene derivatives are excellent tools to examine the relevance of spatial glycoligand orientation.

**Figure 1:**
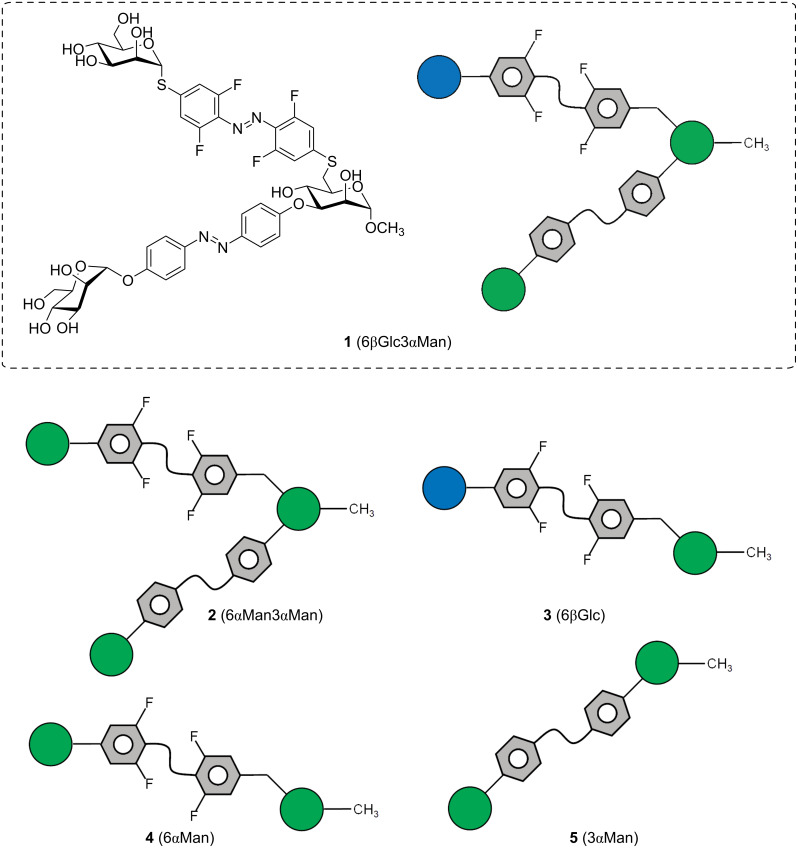
Cartoon of the photoswitchable glycoconjugates investigated in this account. The previously described heterobivalent azobenzene glycocluster 6βGlc3αMan **1** [24] is shown as structural formula and as the corresponding symbolic representation; the other structural formulas are derived accordingly: the homobivalent analog of **1**, 6αMan3αMan **2** and the individual structural components of **1** and **2**, the monovalent glycoazobenzene-functionalized mannosides 6βGlc **3**, 6αMan **4**, and 3αMan **5**. Note that the *ortho*-fluorinated *S*-azobenzene units (ABF_4_) can be isomerized orthogonally to the *O*-azobenzene (AB) photoswitch. (Orthogonal) photoswitching alters the relative spatial orientation of the two sugar units. Glucose (Glc) moieties are colored in blue and mannose (Man) in green according to the symbol nomenclature for carbohydrates (SNFG) [29–30].

In order to expand the potential of photoswitchable glycoconjugates, we have recently introduced the first example of an orthogonally photoswitchable glycocluster in which an azobenzene α-ᴅ-mannoside and an azobenzene β-ᴅ-glucoside unit are conjugated to the 3- and the 6-position of a methyl mannoside scaffold (Figure 1, **1** (6βGlc3αMan)) [24]. Orthogonal photoswitching of the two glycoantennas is guaranteed by (i) *ortho*-fluorination of one azobenzene moiety and (ii) *S*-azobenzene versus *O*-azobenzene conjugation, resulting in significantly shifted UV–vis spectra of the two photoswitchable units. This design laid the basis for a robust switching cycle comprising the *E*_Man_*E*_Glc_, the *E*_Man_*Z*_Glc_, and the *Z*_Man_*Z*_Glc_ isomer of **1** [24].

In this contribution, we address the question of how the different isomers of **1** perform as inhibitors of bacterial adhesion. We have investigated type 1 fimbriae-mediated adhesion of *Escherichia coli* (*E. coli*) bacteria, a process that is fundamental to UPEC (uropathogenic *E. coli*) infections [25]. Type 1 fimbriae are adhesive organelles projecting from the bacterial surface and are terminated by the lectin FimH, which is specific for α-ᴅ-mannopyranosides [26–28]. As shown earlier, a Glc unit can enhance the affinity of the respective glycoconjugate to FimH when conjugated in an appropriate relative orientation to the Man ligand [14–15]. Therefore, it is of particular interest to compare the various isomeric states of the bis-azobenzene glycocluster 6βGlc3αMan **1** representing different relative Man/Glc orientations.

In order to put the biological properties of the previously described photoswitchable heterobivalent cluster glycoside 6βGlc3αMan **1** [24] into perspective, several relevant analogs were synthesized, namely the homobivalent cluster glycoside 6αMan3αMan **2** and the glycoazobenzene portions contained in **1** and **2**, i.e., the glycoazobenzene-functionalized mannosides 6βGlc **3**, 6αMan **4**, and 3αMan **5** (Figure 1). In this account, the photochromic properties of the glycoconjugates **1**–**5** were compared and their potency as inhibitors of Man-specific bacterial adhesion investigated.

## Results and Discussion

### Synthesis

**Scheme 1:**
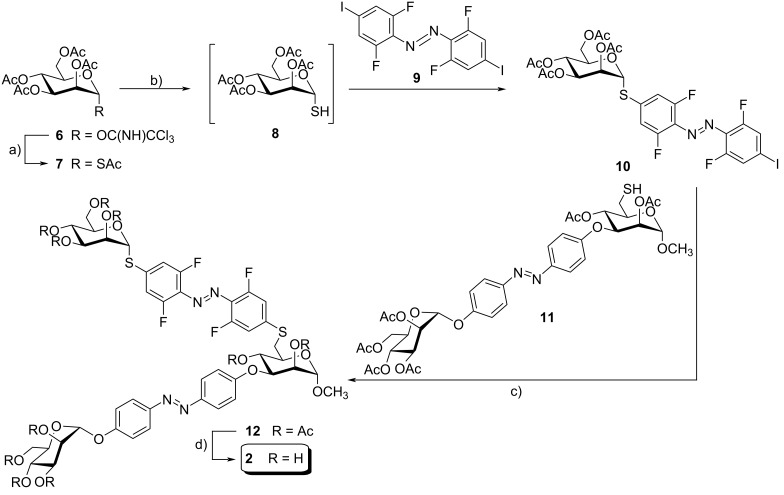
Synthesis of the homobivalent azobenzene glycocluster 6αMan3αMan **2**. Reagents and conditions: a) BF_3_∙Et_2_O, HSAc, dry CH_2_Cl_2_, −10 °C to rt, 18 h, 97%; b) (i) Na_2_CO_3_, MeOH, rt, 3 h; (ii) Xantphos-Pd-G3, Et_3_N, dry THF, −78 °C, 1.5 h, 31% over two steps; c) Xantphos-Pd-G3, Et_3_N, dry THF, rt, 3 h, 59%; d) NaOMe, CH_2_Cl_2_/MeOH 1:2, rt, 2 h, 96%.

For the preparation of the homobivalent glycocluster 6αMan3αMan **2**, the known mannosyl thioacetate **7** [31] was prepared from the trichloroacetimidate **6** [32] and thioacetic acid in an α-selective reaction and excellent yield of 94% (Scheme 1). A chemoselective deprotection of the mannosyl thioacetate **7** to yield the glycosyl thiol **8** was achieved using 0.95 equivalents of sodium carbonate. The crude product was in turn submitted to a Buchwald–Hartwig–Migita cross-coupling reaction [33] with the azobenzene derivative **9** [34] to furnish **10**. This reaction had to be carried out at −78 °C in order to suppress nucleophilic substitution of the *ortho*-fluorine substituents in **9** by the thiol **8**, a reaction that competes with the desired cross-coupling. For the second Buchwald–Hartwig–Migita cross-coupling, the 3-*O*-(mannosyloxyazobenzene)-6-thio-mannoside **11** was employed, which was prepared according to a known procedure applied for the synthesis of the heterobivalent glycocluster 6βGlc3αMan **1** (cf. Supporting Information File 1, Scheme S1). The cross-coupling of thiol **11** with the azobenzene thiomannopyranoside **10** yielded the protected glycocluster **12** in 60% yield. A subsequent Zemplén deacetylation gave the homobivalent glycocluster 6αMan3αMan **2** in quantitative yield (Scheme 1).

**Scheme 2:**
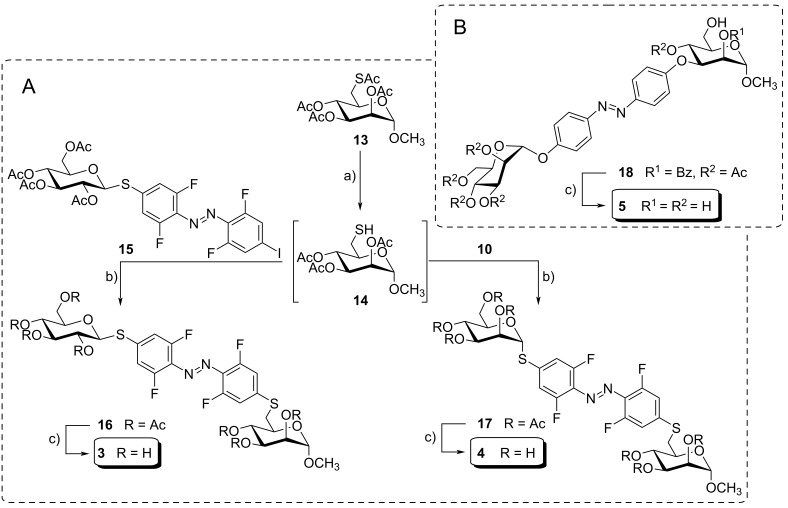
Synthesis of the antennas 6βGlc **3** and 3αMan **4** (A), and 6αMan **5** (B). Reagents and conditions: a) DTT, Et_3_N, dry DMA, rt, 1 d; b) Xantphos-Pd-G3, Et_3_N, dry THF, −10 °C to rt, 20 h, 63% (**16**, over two steps), 73% (**17**, over two steps); c) NaOMe, CH_2_Cl_2_/MeOH 1:2, rt, 3 h, 77% (**3**), quant. (**4**, 4 h), quant. (**5**); DTT: 1,4-dithio-ᴅ-threitol; DMA: dimethylacetamide.

In order to synthesize the glycoazobenzene antennas contained in the glycoclusters **1** and **2**, the β-ᴅ-glucopyranosyloxy- and the α-ᴅ-mannopyranosyloxyazobenzene mannosides 6βGlc **3** and 6αMan **4** were prepared first (Scheme 2A). The synthesis of the β-ᴅ-glucopyranosyl mannoside 6βGlc **3** started from the literature-known *S*,*O*-acetylated mannoside **13** [35], which was chemoselectively deprotected at the 6-position employing DTT (1,4-dithio-ᴅ-threitol) [36] (Scheme 2A). The crude thiol **14** was subsequently subjected to a Buchwald–Hartwig–Migita cross-coupling reaction with the literature-known azobenzene thioglucoside **15** [37], resulting in the (6-thioglucosyl)mannoside **16** in 63% yield over two steps. Zemplén deacetylation [38] gave pure 6βGlc **3** in 77% yield after reversed-phase chromatography (Scheme 2A).

To furnish the glycoazobenzene antenna **4**, complementary to **3**, the azobenzene thiomannopyranoside **10** (cf. Scheme 1), representing the mannose analog of **15**, was used and cross-coupled with **14** to give the acetylated glycoazobenzene antenna **17** in 73% over two steps. Subsequent deacetylation quantitatively yielded 6αMan **4** (Scheme 2A).

Finally, the third required glycoazobenzene antenna, the 3-(α-ᴅ-mannopyranosyl)mannoside 3αMan **5**, was directly obtained from the known mannosyloxyazobenzene mannoside **18** [24] after Zemplén deprotection (Scheme 2B).

### Photochromic properties

Irradiation of the glycoazobenzene-functionalized mannosides 6αMan **3**, 6βGlc **4**, and 3αMan **5** with light of 365, 435, and 520 nm, respectively, led to three photostationary states (PSS) in each case of which the *E*/*Z* ratios were determined by integration of the anomeric proton of the terminal sugar units in the ^1^H NMR spectrum (Table 1 and Supporting Information File 1, Figures S8, S10, and S12). Irradiation with 365 nm light excites the π–π* band of the *ortho*-fluorinated *S*-azobenzene units (ABF_4_) of both **3** and **4** and the π–π* band of the *O*-azobenzene (AB) unit of **5** leading to PSS with *E*/*Z* ratios of 18:82 for **3** and **4** and 3:97 for **5**. Back switching occurs upon irradiation with 435 nm light that excites the n–π* band of the *Z* isomers and leads to almost identical *E*/*Z* ratios (64:36 and 65:35) in all three cases (**3**, **4**, and **5**).

**Table 1:** Comparison of the *E*/*Z* ratios of different PSSs of the heterobivalent bis-azobenzene glycocluster **1** [24] and the homobivalent azobenzene glycocluster 6αMan3αMan **2** and the glycoazobenzene-functionalized individual antennas 6αMan **3**, 6βGlc **4**, and 3αMan **5**. The PSS was reached after irradiation with light of the denoted wavelength for 5 min and determined by NMR spectroscopy.

		PSS 365 nm	PSS 435 nm	PSS 520 nm

**1** ^a,b^	*EE*/*ZE*/*EZ*/*ZZ*	2:15:9:74	56:17:22:5	25:8:66:1
	AB (Man) antenna	11:89	78:22	91:9
	ABF_4_ (Glc) antenna	17:83	73:27	33:67
**2** ^c^	*EE*/*ZE*/*EZ*/*ZZ*^b^	3:12:11:73	40:17:26:17	21:4:63:12
	AB (Man) antenna^d^	3:97	58:42	87:13
	ABF_4_ (Man) antenna^d^	17:83	64:36	21:79
**3** ^e,f^	ABF4 (Glc) antenna	18:82	64:36	23:77
**4** ^e,f^	ABF4 (Man) antenna	18:82	65:35	23:77
**5** ^e,f^	AB (Man) antenna	3:97	64:36	85:15

^a^Recorded in MeCN-*d*_3_:DMSO-*d*_6_ 9:1; ^b^resulting from integration of the anomeric ^1^H/^13^C cross peaks of the scaffold mannoside in the ^1^H,^13^C HSQC NMR spectrum; ^c^recorded in MeCN-*d*_3_/DMSO-*d*_6_ 8:2; ^d^resulting from integration of the anomeric ^1^H/^13^C cross peaks of the mannoside moieties attached to the switching units AB or ABF_4_, respectively, in the ^1^H,^13^C HSQC NMR spectrum; ^e^determined by integration of the H-1’ peaks in the ^1^H NMR spectrum; ^f^recorded in MeCN-*d*_3_/DMSO 8:2 (with DMSO signal suppression). For UV–vis spectra see Supporting Information File 1, Figures S1, S7, S9, and S11.

Irradiation of **5** with 520 nm light, on the other hand, leads to a PSS with more *E* isomer, namely an *E*/*Z* ratio of 85:15. Irradiation of **3** and **4** with light of 520 nm effects an *E* to *Z* isomerization with an *E*/*Z* ratio of 23:77 in both cases, since 520 nm light addresses the bathochromically shifted n–π* band of the *E* isomers due to the *ortho*-fluoro functionalization of the ABF_4_ unit.

The *EE*/*ZE*/*EZ*/*ZZ* isomeric mixture obtained after irradiation of the homobivalent photoswitchable glycocluster 6αMan3αMan **2** had to be determined by integration of the anomeric ^1^H/^13^C cross peak of the scaffold mannoside in the ^1^H,^13^C HSQC spectrum because the proton signals in the ^1^H NMR spectrum are not clearly separated (cf. Supporting Information File 1, Figure S3). This procedure was published earlier for the heterobivalent glycocluster **1** [24], however, in comparison to **1**, the anomeric ^1^H/^13^C cross peaks of the *EE*, *ZE*, *EZ*, and *ZZ* isomers of **2** are less well resolved and therefore, the deduced *EE*/*ZE*/*EZ*/*ZZ* ratios are less accurate. Nevertheless, it can be seen that irradiation of **2** with 365 nm light leads to a *ZZ*-predominated state, exposure to light of the wavelength 435 nm to an *EE*-predominated state and 520 nm light results in a *ZE*-predominated state (Table 1). The *E*/*Z* ratios of the individual glycoazobenzene antennas in **2** could be accurately determined by integration of the respective peaks in the ^1^H,^13^C HSQC spectrum, namely the anomeric ^1^H’/^13^C’ and ^1^H’’/^13^C’’ cross peaks (Table 1 and Supporting Information File 1, Figures S4–S6).

Comparison of the data collected in Table 1 shows that the PSS values of the bis-azobenzene glycoclusters **1** and **2** are in a similar range. Furthermore, the glycoazobenzene antennas comprised in **1** and **2** show a similar switching behavior as the individual antennas 6αMan **3**, 6βGlc **4**, and 3αMan **5**.

**Figure 2:**
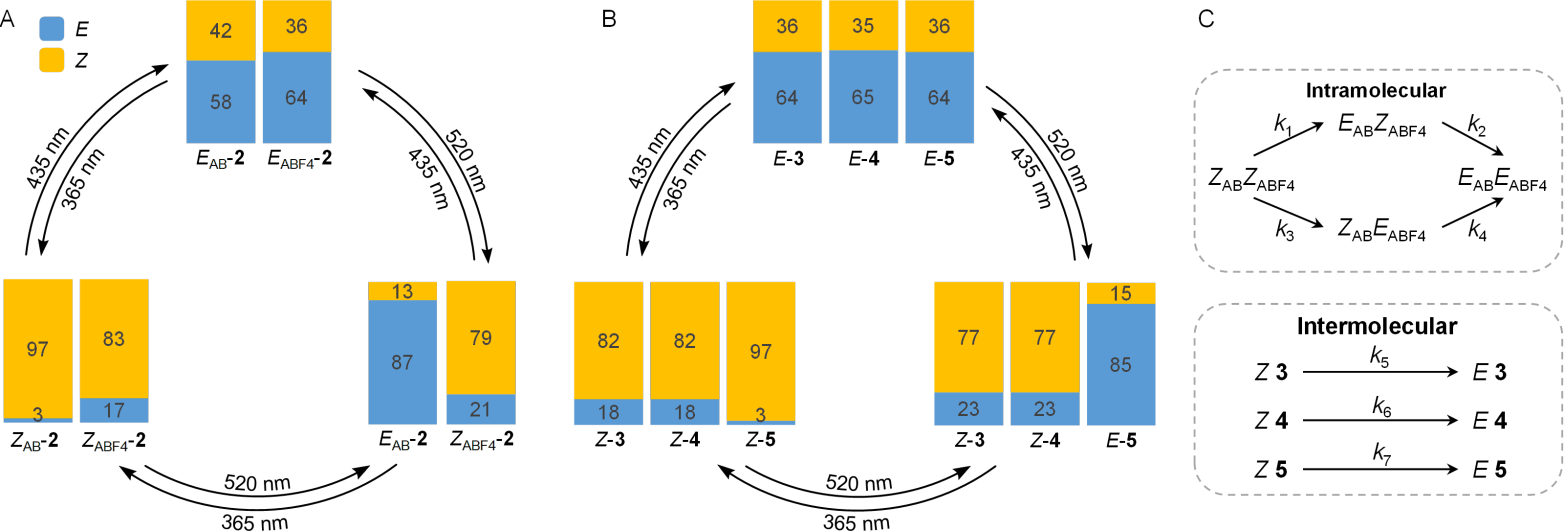
A: Wavelength-selective photoswitching of the α-ᴅ-mannopyranosyloxy-AB and -ABF_4_ antennas comprised in the homobivalent glycocluster **2**. The PSS values after irradiation with 365, 435, and 520 nm light, respectively, are shown (cf. Table 1). B: Wavelength-selective photoswitching of the azobenzene mannosides **3**, **4**, and **5** after irradiation with 365, 435, and 520 nm light (cf. Table 1). C: The thermal relaxation of **2** from *Z*_AB_*Z*_ABF4_ to *E*_AB_*E*_ABF4_ is described by the rate constants *k*_1_–*k*_4_ (top) and thermal relaxation of *Z*-**3**, *Z*-**4**, and *Z*-**5** is described by *k*_5_, *k*_6_, and *k*_7_ (bottom).

Wavelength-selective photoswitching of **2** as well as of **3**, **4**, and **5** is illustrated by the respective switching cycles in Figure 2A and B.

In a next step, thermal relaxation of the metastable *ZZ* and *Z* isomers, respectively, was studied. In **2**, thermal relaxation of the *ZZ* isomer can occur via the *EZ* or the *ZE* isomer (Figure 2C), respectively [24,39]. The kinetics of these processes are described by the rate constants *k*_1_, *k*_2_, *k*_3_, and *k*_4_, where *k*_1_ and *k*_4_ describe thermal relaxation of the AB unit and *k*_2_ and *k*_3_ of the ABF_4_ unit, respectively. For the thermal *Z* to *E* relaxation of the individual glycoazobenzene antennas 6αMan **3**, 6βGlc **4**, and 3αMan **5**, first order reactions were assumed with the rate constants *k*_5_, *k*_7_, and *k*_6_ (Figure 2C).

In order to determine the half-lifes τ_1/2_ and activation energies *E*_a_ of the photoswitching units comprised in the homobivalent glycocluster 6αMan3αMan **2**, the decay of the metastable *ZZ* isomer to the thermodynamically stable *EE* isomer was followed over time starting from the PSS@365 nm. The kinetic traces of the relaxation process were recorded by ^1^H NMR spectroscopy at 37 °C (Supporting Information File 1, Figure S2) and the population of the *EE*, *ZE*, *EZ*, and *ZZ* isomers were plotted against time. Using a published tailor-made fitting program [24], the rate constants *k*_1_–*k*_4_ were extracted from the kinetic traces and are summarized in Table 2. The thermal relaxation of the glycoazobenzene antennas **3**, **4**, and **5**, on the other hand, was monitored by UV–vis spectroscopy at 37 °C and the rate constants *k*_5_, *k*_6_, and *k*_7_ obtained with a first-order exponential decay fit (Table 2 and Supporting Information File 1, Figures S7, S9, and S11). The half-lifes τ_1/2_ and the activation energies *E*_a_ of thermal relaxation were calculated based on the determined rate constants.

**Table 2:** Rate constants (*k*), resulting half-lifes (τ_1/2_) and activation energies (*E*_a_) for the thermal *ZZ* to *EE* isomerization of glycoclusters **1** [24] and **2** and thermal *Z* to *E* isomerization of the glycoantennas **3**, **4**, and **5** at 37 °C.

		*k* [s^−1^]	τ_1/2_ [h]	*E*_a_ [kJ mol^−1^]

**1** ^a^	*k*_1_	2.93E−05 ± 0.08E−05	6.56 ± 0.18	102.9
	*k*_2_	3.71E−06 ± 0.37E−06	52.90 ± 4.68	108.3
	*k*_3_	3.53E−06 ± 0.41E−06	54.16 ± 6.07	108.4
	*k*_4_	3.53E−05 ± 0.41E−05	5.46 ± 0.57	102.4
**2** ^b^	*k*_1_	3.39E−05 ± 0.06E−05	5.67 ± 0.09	102.6
	*k*_2_	2.10E−06 ± 0.25E−06	91.88 ± 9.81	109.7
	*k*_3_	8.28E−07 ± 0.37E−07	23.25 ± 1.01	106.2
	*k*_4_	3.08E−05 ± 0.19E−05	6.25 ± 0.37	102.8
**3** ^c^	*k*_5_	4.03E−06 ± 0.01E−06	47.80 ± 0.13	108.0
**4** ^c^	*k*_6_	3.51E−06 ± 0.02E−06	54.87 ± 0.26	108.4
**5** ^c^	*k*_7_	6.70E−05 ± 0.01E−05	2.88 ± 0.01	71.4

^a^Determined by ^1^H NMR spectroscopy (in MeCN-*d*_3_/DMSO-*d*_6_ 9:1); ^b^determined by ^1^H NMR spectroscopy (in MeCN-*d*_3_/DMSO-*d*_6_ 8:2); ^c^determined by UV–vis spectroscopy (in DMSO).

The kinetics show that thermal relaxation of the ABF_4_ unit (*k*_2_ ≈ 92 h; *k*_3_ ≈ 23 h; *k*_5_ ≈ 48 h, and *k*_6_ ≈ 55 h) is slower than that of the AB unit (*k*_1_ ≈ 6 h; *k*_4_ ≈ 6 h; *k*_7_ ≈ 3 h) in all investigated photoswitches (**1** [24], **2**, **3**, **4**, and **5**). This switching behavior is also reflected in the half-lifes. Note that *k*_3_ is subject to a large error as relaxation from *ZZ* to *ZE* is practically not observed because it is so slow (for kinetic traces cf. Supporting Information File 1, Figure S2). Furthermore, the data reveal that the isolated glycoazobenzene antennas **3**, **4**, and **5** relax faster than the AB and ABF_4_ moieties comprised in the homobivalent glycocluster **2**. This could be due to incomplete electronic decoupling of the antennas as seen earlier in glycocluster **1** [24,40].

The results obtained here for compounds **2**, **3**, **4**, and **5** are in accordance with those previously reported for the heterobivalent photoswitch **1** (cf. Figure 1) [24]. In any case, the half-lifes of the various isomers at 37 °C are long enough to persist during the time a bioassay takes. In the PSS of the *EE*, *EZ*, and *ZZ* states, the respective isomers clearly predominate the mixtures and hence, testing the various isomeric states in bacterial inhibition–adhesion assays can lead to relationships between ligand orientation and anti-adhesive properties of the respective inhibitor.

### Biological testing

For the adhesion-inhibition assays, type-1-fimbriated *E*. *coli* bacteria were employed where the α-ᴅ-mannoside-specific adhesion is mediated by the fimbrial lectin FimH. According to a known protocol [41], the fluorescent GFP (green fluorescent protein)-expressing strain PKL1162 was applied to mannan-coated microtiter plates. In this assay, fluorescence intensity correlates with the number of adhered bacterial cells. The photoswitches **1**–**5** (cf. Figure 1) were used as inhibitors of bacterial adhesion in serial dilutions leading to dose–response inhibition curves from which IC_50_ values for each inhibitor were deduced (cf. Supporting Information File 1, Figures S13–S16). As these values can vary between individual assays quite significantly, IC_50_ values for each inhibitor are compared to methyl α-ᴅ-mannoside (MeMan) measured on the same plate as reference. This leads to relative inhibitory potencies, so-called RIP values.

Because of limited water solubility, the samples had to be dissolved in DMSO and therefore, the effect of pure DMSO was also tested in every individual assay (cf. Supporting Information File 1, Figures S13–S16). DMSO is known for its bactericidal effect and still, the use of this organic solvent in bioassays is common [42]. In general, a DMSO concentration of less than 10% (v/v) is accepted as nontoxic [43]. In any case, the effect of DMSO in the assays was carefully controlled (cf. Supporting Information File 1, Figures S13–S16).

**Figure 3:**
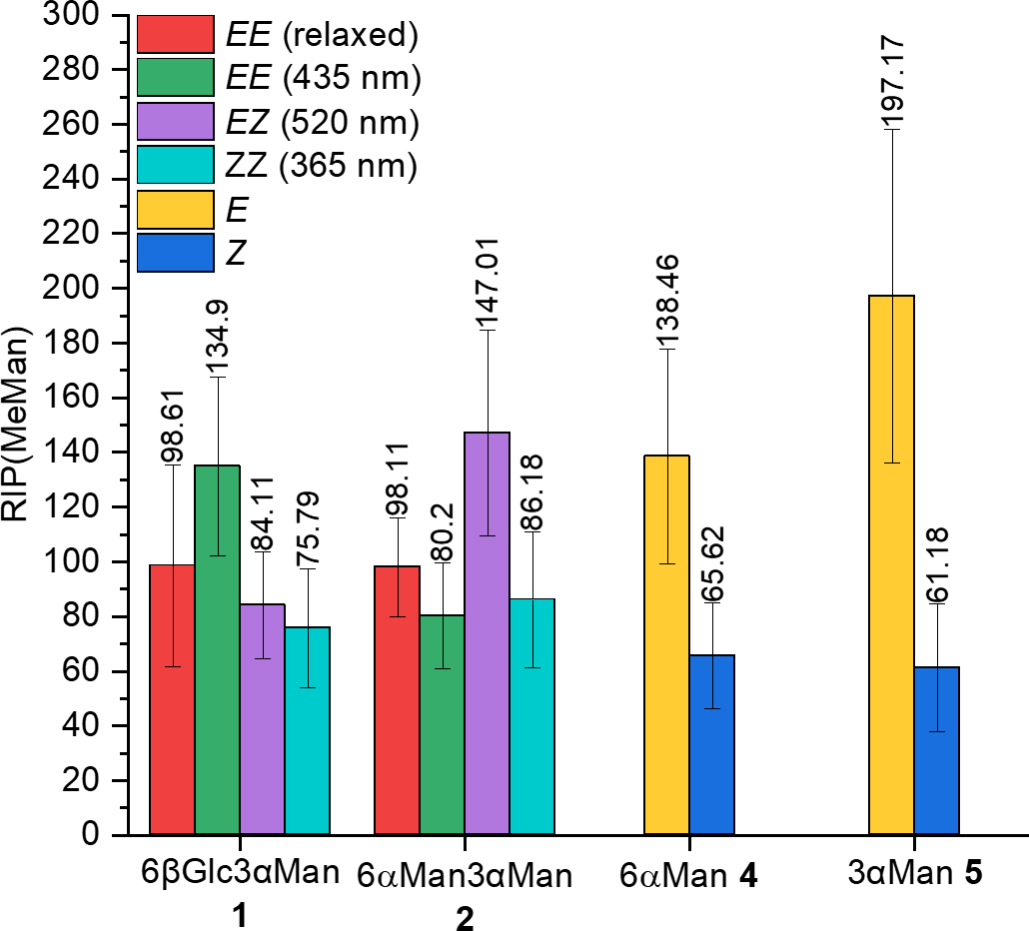
Comparison of the inhibitory potencies of **1**, **2**, **4**, and **5** in the different isomeric states. The depicted RIP(MeMan) values are relative to the reference inhibitor MeMan tested on the same plate. Error bars of the inhibition of *E. coli* (GFP-PKL1162) adhesion to mannan reflect standard deviations (cf. Supporting Information File 1, Table S6).

The testing results are illustrated in Figure 3. All photoswitches comprising an α-ᴅ-mannopyranose ligand bound to an azobenzene unit (**1**, **2**, **4**, and **5**) show a stronger inhibitory potency than MeMan. This can be explained by, i.a., π–π-interactions of the aromatic azobenzene portion with the so-called tyrosine gate at the entrance of the FimH carbohydrate binding domain constituted by Tyr48 and Tyr137 [44]. In case of the glycoazobenzene antennas 6αMan **4** and 3αMan **5**, it is apparent that the inhibitory potency of the individual glycoconjugate is dependent on the configuration of the azobenzene photoswitch (*E* or *Z*). In both cases, the *E* isomers exhibit significantly higher RIP(MeMan) values than the *Z* isomers, approx. two times higher for **4** and approx. three times higher for **5** (cf. Supporting Information File 1, Table S6).

The bis-azobenzene glycoclusters **1** and **2** were tested in four isomeric states, (i) the relaxed state in which both azobenzene units, AB and ABF_4_, are completely present in the *E* form, (ii) the PSS@365, (iii) the PSS@435, and (iv) the PSS@520 nm. It has to be noted that in each PSS, in fact an isomeric mixture was present (cf. Table 1), which is, however, predominated by one specific isomer. Different RIP(MeMan) values were obtained for the different isomers with RIP(MeMan) values varying between 75.8 and 134.9 for **1** and between 80.2 and 147.0 for **2** (Figure 3 and Supporting Information File 1, Table S6). For the heterobivalent glycocluster 6βGlc3αMan **1**, the inhibitory potencies of the four isomeric states can be ranked as follows: *EE*(435 nm) > *EE*(relaxed) > *EZ*(520 nm) > *ZZ*(365 nm). For the homobivalent glycocluster 6αMan3αMan **2**, the inhibitory potencies follow a different ranking: *EZ*(520 nm) > *EE*(relaxed) > *ZZ*(365 nm) > *EE*(435 nm).

For the glycoazobenzene antenna 6βGlc **3** no inhibitory effect can be expected as it does not project an α-ᴅ-mannopyranose ligand, but a β-ᴅ-glucopyranose unit instead. Nevertheless, 6βGlc **3** shows an inhibitory potency which is approx. one order of magnitude higher than that of MeMan under the conditions applied for the assay. This inhibitory effect is due to DMSO, which had to be used to dissolve the sample. Consequently, the RIP relative to DMSO is approx. 1 for both isomers of **3** (cf. Supporting Information File 1, Table S6). In all other cases, comparison of the RIP(MeMan) and the RIP(DMSO) values shows that the obtained inhibitory effect is not only due to DMSO, but is largely effected by the tested glycoconjugates. Though relatively high volume percentages of DMSO were used in the assays, especially for the high sample concentrations (cf. Supporting Information File 1, Figures S13–S16), the analysis of the testing results shows that determination of the inhibitory potencies of the various isomers is indeed valid in spite of the limited water solubility of the samples.

Looking over the measured inhibitory potentials, it can be concluded that (i) the isomeric state of the tested photosensitive glycoconjugates significantly influences their inhibitory properties and (ii) that this effect is clear for 6αMan **4** and 3αMan **5**, whereas the effect of the isomeric state in the bis-azobenzene glycoclusters **1** and **2** is ambiguous.

Based on published work [20–23], we hypothesize that isomerization of the azo group in the tested azobenzene glycoconjugates controls the orientation of the attached glycoligand and therefore, different glycoazobenzene isomers show various affinities for the bacterial lectin FimH as reflected by the measured RIP values. This is clearly seen with compounds **4** and **5**. In the bivalent glycoclusters **1** and **2**, the relative orientation of the two glycoazobenzene antennas is altered upon photoisomerization. Here, the effects are less clear. It can be assumed that one antenna is specifically bound within the FimH carbohydrate recognition domain (CRD), whereas the second antenna can exert more or less favorable secondary binding effects at the periphery of the carbohydrate binding site, depending on the isomeric state. In order to rationalize these hypotheses, we commenced a molecular modeling study to inspect the different isomeric states of the tested azobenzene glycoconjugates **1**–**5** in complex with the bacterial lectin FimH.

### Molecular modeling

Docking studies were performed with the different isomers (*E* and *Z* or *EE*, *EZ*, *ZE*, and *ZZ*) of the azobenzene glycoconjugates **1**, **2**, **4**, and **5**, containing a mannoside ligand, and the bacterial lectin FimH (for **3**, cf. Supporting Information File 1). The calculations were performed with two conformers of FimH, the open and the closed gate conformation, PDB 1KLF [45] and PDB 1UWF [46], respectively. These protein structures differ in the relative orientation of the so-called tyrosine gate, which is formed by Tyr48 and Tyr137 flanking the carbohydrate binding site of FimH [47]. First, Glide [48] as part of the Maestro interface of the Schrödinger software package [49] was used to calculate docking scores (Table 3). More negative docking scores correlate with higher FimH affinity. In a second step, the best docking results were submitted to a MM-GBSA calculation (molecular mechanics with generalized Born and surface-area solvation) [50] leading to the binding energies of the different isomers of **1**, **2**, **4**, and **5** for both protein conformations (Table 3 and Supporting Information File 1, Tables S13–S18).

**Table 3:** The top-ranked results of the molecular modeling study of the various isomers of the ligands 6βGlc3αMan **1**, 6αMan3αMan **2**, 6αMan **4**, and 3αMan **5** are listed together with the RIP(MeMan) values. The best docking scores obtained using Glide for the open and closed gate crystal structures of FimH (PDB: 1KLF and 1UWF) with the corresponding binding energy (MM-GBSA) are shown. Furthermore, the best docking scores obtained using induced fit docking (IFD) for the closed conformation of FimH with the corresponding binding energy (MM-GBSA) of the most stable protein–ligand complexes according to the binding pose metadynamic calculation are shown. Lower docking and binding energy values indicate stronger binding to FimH.

Inhibitor RIP(MeMan)	Glide score		Binding energy [kcal mol^−1^]		IFD score	IFD binding energy [kcal mol^−1^]
	1KLF (open gate)	1UWF (closed gate)	1KLF (open gate)	1UWF (closed gate)	1UWF (closed gate)	1UWF (closed gate)

*EE*-6βGlc3αMan **1** 98.61 (relaxed); 134.90 (435 nm)	−10.520	−12.134	−80.30	−91.17	−342.16	−69.57
*EZ*-6βGlc3αMan **1** 84.11 (520 nm)	−7.452	−12.728	−58.30	−92.79	−343.31	−88.33
*ZZ*-6βGlc3αMan **1** 75.79 (365 nm)	−11.787	−8.698	−71.17	−85.49	−343.48	−107.61
*ZE*-6βGlc3αMan **1** (–)	−10.942	−10.651	−63.99	−74.81	−341.97	−82.98
*EE*-6αMan3αMan **2** 98.11 (relaxed); 80.20 (435 nm)	−8.726	−10.154	−59.07	−86.42	−343.58	−89.99
*EZ*-6αMan3αMan **2** 147.01 (520 nm)	−11.233	−11.273	−80.04	−68.14	−344.66	−69.20
*ZZ*-6αMan3αMan **2** 86.18 (365 nm)	−10.035	−10.528	−65.70	−76.17	−343.63	−96.22
*ZE*-6αMan3αMan **2** (–)	−9.862	−10.181	−80.29	−75.82	−345.03	−76.98
*E*-6αMan **4** 138.46 (relaxed)	−10.440	−10.222	−65.53	−70.01	−340.76	−83.07
*Z*-6αMan **4** 65.62 (365 nm)	−10.190	−9.340	−64.64	−60.96	−340.45	−64.40
*E*-3αMan **5** 197.17 (relaxed)	−8.195	−9.980	−59.19	−71.08	−340.33	−71.41
*Z*-3αMan **5** 61.18 (365 nm)	−10.375	−9.650	−62.18	−59.43	−338.74	−67.74

The determined binding energies for 6αMan **4** and 3αMan **5** in complex with the closed gate conformation of FimH reflect the experimental data much better than the binding energies calculated for the open gate conformation (where very similar values were obtained for the *E* and the *Z* isomers of both compounds). Modeling with the closed gate conformation of FimH led to binding energies of −70.01 kcal mol^−1^ for *E*-**4** and −71.08 kcal mol^−1^ for *E*-**5** versus −60.96 kcal mol^−1^ for *Z*-**4** and −59.43 kcal mol^−1^ for *Z*-**5**. These calculation results correspond to the experimentally determined inhibitory potencies which are significantly higher for the *E* isomers than for the corresponding *Z* isomers. As a consequence, further refinement of the molecular modeling was performed with the closed gate conformation of FimH.

In the next step, induced fit docking (IFD) [51] was employed (cf. Supporting Information File 1, Tables S19–S21). In IFD, not only the ligand but also the carbohydrate binding region of the protein is flexible, allowing for modeling of the ligand–FimH interaction as a dynamic event [52–53]. The five best binding poses of each receptor–ligand complex obtained in IFD were then used in a binding pose metadynamics simulation (MD) [54] over 10 ns to determine the relative stability of the complexes (Supporting Information File 1, Figure S17 and Figure S18). The most stable receptor–ligand complexes so obtained were then submitted to a MM-GBSA calculation to deliver IFD binding energies (Table 3 and Supporting Information File 1, Table S25).

**Figure 4:**
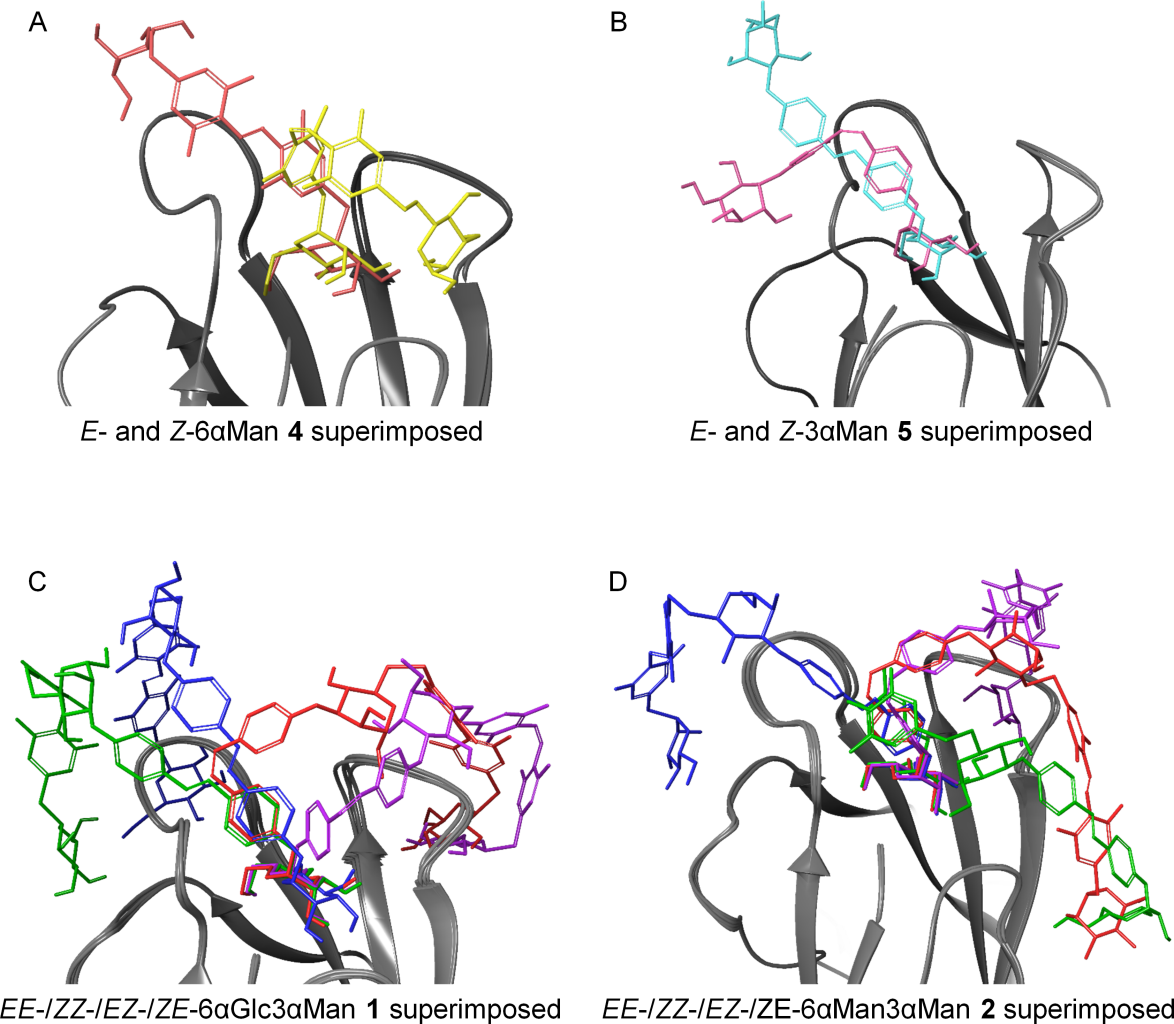
Three-dimensional representation of the superimposed most stable ligand–protein complexes from IFD for *E*- and *Z*-6αMan **4** (A: *E* in red, *Z* in yellow) and *E*- and *Z-*3αMan **5** (B: *E* in turquoise, *Z* in magenta), as well as of the *EE*, *ZZ*, *EZ*, and *ZE* isomers of glycocluster 6βGlc3αMan **1** (C) and of 6αMan3αMan **2** (D). The protein FimH (1UWF) is depicted as ribbon diagram and the ligands are displayed as stick models (glycoclusters **1** and **2**: *EE*: blue; *ZZ*: violet; *EZ*: green; *ZE*: red). Superposition of the isomers shows the similarity of the binding of the terminal mannoside antenna within the FimH CRD and the different orientations of the “rest” of the molecule at the periphery of the CRD (the scaffold mannoside and, for **1** and **2**, the second antenna).

Also the binding energies obtained with IFD for 6αMan **4** and 3αMan **5** are in accordance with the experimental data as they predict correctly that the ligand–FimH complexes formed with the *E* isomers of **4** and **5** are more stable than the complexes formed with the respective *Z* isomers. These differences correspond to the structures of the various ligand–FimH complexes (Figure 4A and B), however, it is difficult to substantiate the measured binding differences in detail. For example, it is not apparent that secondary interactions performed by the scaffold mannoside portion in the periphery of the FimH form the basis of the higher FimH affinity of the *E* isomers (as was argued in similar case [15]) (cf. Supporting Information File 1, Figure S25 and Figure S26). A possible allosteric regulation of the CRD structure [55–56], on the other hand, which might be effected by one of the isomers, cannot be proven at the level of the calculation used here.

For glycoclusters 6βGlc3αMan **1** and 6αMan3αMan **2**, on the other hand, the experimental results obtained in the bioassays cannot be rationalized in this docking study. Even the trend of the IFD binding energies does not match the measured RIP values. However, for comparison of the experimental with the theoretical data it has to be kept in mind that the pure isomers were used for docking whereas biological testing was performed with the PSS mixtures where one isomer was predominant but the only isomer present. After all, molecular modeling illustrates the different FimH binding modes of the various isomers of **1** and **2** (Figure 4C and D). Inspection of these ligand–protein complexes reveals that in all isomers, a terminal α-ᴅ-mannoside ligand is complexed within the FimH carbohydrate binding pocket where it forms the classical network of hydrogen bonds (cf. Supporting Information File 1, Figures S20 and S21 for **1** and Figures S23 and S24 for **2**). Apparently, secondary interactions at the periphery of the FimH CRD, which can be exerted by the scaffold mannoside and the second antenna in **1** or **2**, respectively, can be quite different and sometimes also similar depending on the isomeric state (*E* or *Z*) of the azobenzene hinge, regardless of whether the second antenna projects a glucose (**1**) or a mannose (**2**) moiety. Whereas in compounds **4** and **5**, the *E* isomers show higher binding energies, in the heterobivalent glycocluster 6βGlc3αMan **1**, the *EE* isomer has the lowest and the *ZZ* isomer the highest binding energy. Comparison of the molecular interactions as revealed by molecular modeling shows that the second, non CRD-bound antenna adopts different orientations in the *EE* versus the *ZZ* isomer exerting different secondary interactions with FimH (Supporting Information File 1, Figures S19–S21). In addition to the π–π interaction of the CRD-bound mannose-projecting antenna of the *EE* isomer with Tyr48, the complex is stabilized by hydrogen bonds of the scaffold mannoside to Tyr48 and in case of the glucose moiety to Thr51, Ile52, and Thr53. The high binding energy of the *ZZ* isomer, on the other hand, can be explained by additional hydrogen bonds of the scaffold mannoside to Asn138 and Ser139 (but not to Tyr48) and furthermore, the glucose moiety interacts with Arg92, Asn135, Asn138, and Asp140. The binding energies predicted for the *EZ* and *ZE* isomers of **1** are very similar despite the different spatial orientation of the second glycoantenna. Apparently, the glucose moiety contributes to FimH affinity to a similar extent in both isomeric states by exerting interactions with Asn96, Arg98, and Glu50 in case of the *EZ* isomer and with Arg92, Asn138, and Asn135 in the *ZE* isomer.

Comparison of the *EE* and the *ZZ* isomers of the homobivalent glycocluster 6αMan3αMan **2** also shows very different orientations of the second, non CRD-bound antenna. In case of the *EE* isomer, the π–π interaction of the CRD-complexed glycoazobenzene antenna with Tyr48 and Arg98 and interactions of the second antenna with Arg98 and Asn96 stabilize the ligand–FimH complex. The higher affinity of the *ZZ* isomer can be explained by additional interactions exerted by the second antenna with Arg92, Asn135, Asn138, and Asp140 (equal to what is observed in the *ZZ* and the *EZ* isomer of 6βGlc3αMan **1**) (Supporting Information File 1, Figures S22–S25). In the *EZ* and *ZE* isomers of **2**, the second, non CRD-bound antenna interacts with Gln143 on the backside of the FimH CRD and an additional π–π interaction with Phe142 is seen for the *EZ* isomer. Interestingly, and somewhat counter-intuitively, in both the *EZ* and *ZE* isomers, the *Z*-configured antenna is complexed within the FimH CRD.

Overall, docking studies suggest that the FimH interactions of the individual antennas **4** and **5** closely match the behavior of the analogous antennas comprised in the glycoclusters **1** and **2**. Hence, *E-*3αMan **5** superimposes with the corresponding Man antennas in glycoclusters *EE*-6αGlc3αMan **1** and *EE*-6αMan3αMan **2** (Supporting Information File 1, Figure S27); and *Z*-6αMan **4** superimposes with the CRD-bound antenna comprised in *EZ*-6αMan3αMan **2** (Figure S28).

From the molecular modeling study the following conclusions can be drawn. (i) In all inspected azobenzene glycoconjugates (**1**, **2**, **4**, and **5**), a terminal α-ᴅ-mannopyranoside unit is complexed within the FimH CRD, as expected. (ii) The isomeric state of the azo group in the monovalent azobenzene glycosides **4** and **5** alters the orientation of the non-complexed part of the molecule at the periphery of the FimH CRD and this effect is decisive for the affinity of the respective isomer for FimH, as reflected by the calculated binding energies and the experimentally determined inhibitory potencies. (iii) The interactions which are exerted between the bivalent azobenzene glycosides **1** and **2** in the various isomeric states (*EE*, *EZ*, *ZE*, and *ZZ*) and the lectin FimH reach a complexity which obscures the effect of azo group isomerization. (iv) Calculated binding energies for the highest affinity isomers of **1** and **2**, respectively, (−107.61 and −96.22 kcal mol^−1^) surpass those of the *E* isomers of **4** and **5** (−83.07 and −71.41). This can be attributed to additional secondary interactions at the periphery of the FimH CRD.

## Conclusion

To investigate the effect of relative ligand orientation in carbohydrate recognition, we have recently introduced the first example of an orthogonally photoswitchable glycocluster combining an azobenzene α-ᴅ-mannoside and an azobenzene β-ᴅ-glucoside unit on a methyl mannoside scaffold (**1**) [24]. In this account, the heterobivalent glycocluster **1** was tested as inhibitor of mannose-specific bacterial adhesion in its various isomeric states (*EE*, *EZ*, *ZE*, and *ZZ*) and systematically compared to its homobivalent counterpart **2** and the monovalent glycoantennas **4** and **5**. A careful investigation of the photochromic properties and of the thermal relaxation of **1**, **2**, **4**, and **5** revealed that (i) the new homobivalent glycocluster 6αMan3αMan **2** behaves analogously to the known heterobivalent glycocluster 6βGlc3αMan **1**, meaning that three isomeric states in which the *EE*, *EZ*, and *ZZ* isomers, respectively, are predominating can be selectively reached by light of the appropriate wavelength. (ii) The half-lifes of thermal relaxation are long enough to allow testing of the various isomers in adhesion–inhibition assays with *E. coli*.

The bioassays then revealed that for the monovalent glycoazobenzene photoswitches **4** and **5**, the *E* isomers show a higher inhibitory potency in FimH-mediated bacterial adhesion than the Z isomers (2 to 3 times). Molecular modeling results are in accordance with the experimental trend. Docking clearly shows the differentiated shapes of the respective isomers in complex with FimH. Hence, the isomeric state of a photoswitchable FimH ligand controls its affinity in this case. On the other hand, the inhibitory potencies of the *EE*, *EZ*, and *ZZ* states of **1** and **2** could be less conclusively explained on the level of theory applied here. Again, the molecules adopt different shapes in the various isomeric states, thus confirming our approach to affect molecular shape by isomerization of the azo group in an azobenzene glycoconjugate. In every case, a terminal α-ᴅ-mannopyranoside unit is complexed within the FimH CRD. However, controlling the exact relative orientation of glycoligands in three-dimensional space is difficult in case of **1** and **2** as the degrees of freedom connected to the conformational dynamics are too large. Nevertheless, the FimH-interactions of those parts of the glycocluster, which are not complexed within the FimH CRD, could be analyzed in detail.

It has to be kept in mind that though defined isomers were used in the docking studies, in the biological assays in fact isomeric mixtures were tested. However, as the various PSS are clearly predominated by one isomer (cf. Table 1 and Figure 2A and B), the measured inhibitory potential can be indeed correlated to the impact of glycan conformation within certain boarders of accuracy.

In conclusion, the isomeric state of the azo group in photoswitchable azobenzene glycoconjugates differentiates the orientation of attached glycoligands thus effecting its biological properties (here inhibition of carbohydrate-specific bacterial adhesion). Orthogonally photoswitchable glycoconjugates, in which more than one glycoligand is conjugated to azobenzene units, allow for re-orientation of one glycoligand in the presence of another glycoligand that remains unaffected. It seems to be obvious that an “optoglycomics” approach in the glycosciences in order to investigate carbohydrate function by light can lead to new insights into carbohydrate recognition. However, complex systems like glycoclusters **1** and **2** are capable of many conformational alternatives and therefore, the control of molecular shape by photoisomerization of azobenzene linkers has its limitations. In addition, water solubility requires improvement.

Furthermore, our study, combining synthesis, analysis of photochromic properties, biological testing, and molecular modeling, suggests that future investigations could beneficially apply immobilized orthogonal glycoazobenzene photoswitches. In such a setup, the herein introduced concepts could even lead to more conclusive insights into the role of ligand orientation in carbohydrate recognition on surfaces.

## Supporting Information

File 1 Experimental section and copies of spectra.

## Data Availability

All data that supports the findings of this study is available in the published article and/or the supporting information of this article.
