# Supplementary material for: Orthogonal photoswitching of heterobivalent azobenzene glycoclusters: the effect of glycoligand orientation in bacterial adhesion
Source: Beilstein J Org Chem. 2025 Apr 8;21:736–48. doi: 10.3762/bjoc.21.57 (PMC11995721; doi:10.3762/bjoc.21.57)
Supplement: File 1 — Experimental section and copies of spectra. [file Beilstein_J_Org_Chem-21-736-s001.pdf]

BEILSTEIN JOURNAL OF ORGANIC CHEMISTRY

Supporting Information

for

Orthogonal photoswitching of heterobivalent azobenzene glycoclusters: the effect of glycoligand orientation in bacterial adhesion

Leon M. Friedrich and Thisbe K. Lindhorst

Beilstein J. Org. Chem. **2025,** *21,* 736–748. doi:10.3762/bjoc.21.57

Experimental section and copies of spectra

License and Terms: This is a supporting information file under the terms of the Creative Commons Attribution License (https://creativecommons.org/licenses/by/4.0). Please note that the reuse, redistribution and reproduction in particular requires that the author(s) and source are credited and that individual graphics may be subject to special legal provisions.
The license is subject to the *Beilstein Journal of Organic Chemistry* terms and conditions: (https://www.beilstein-journals.org/bjoc/terms)

Table of contents

1 Synthesis S2

1.1 Materials and methods S2

1.2 Experimental procedures S2

2 Photochromic properties S13

2.1 6αMan3αMan **2** S13

2.2 6βGlc **3** S16

2.3 6αMan **4** S17

2.4 3αMan **5** S18

3 Biological testing S19

3.1 General S19

3.2 Inhibition curves resulting from adhesion-inhibition assays with *E. coli* bacteria (PKL1162) S20

4 Docking studies S26

5 ^{1}H, ^{19}F and ^{13}C NMR spectra S57

6 References S82

1 Synthesis

1.1 Materials and methods

Moisture-sensitive reactions were carried out under a positive pressure of nitrogen in dry glassware. The TLC analysis was carried out on silica gel precast films Alugram® Xtra Sil G/UV254 from Macherey-Nagel. Visualization was accomplished by UV light and/or with 10% sulfuric acid in ethanol, vanillin (3.0 g vanillin and 0.5 mL H_2SO_4 in 100 mL EtOH), followed by heat treatment at approx. 200 °C. Column chromatography was performed on silica gel (particle size 0.04–0.063 nm) from Merck. Reversed-phase chromatography was performed with an Interchim puriFlash® 5.020 system with a Chromabond® RS 25 C_{18} column from Macherey-Nagel. Melting points (mp) were determined with the Melting Point M-560 instrument from Büchi. Optical rotations were measured with an Anton Paar MCP 5100 polarimeter with an internal LED (589 nm) and a cuvette of 10 cm path length at 20 °C in the indicated solvents. For optical rotation measurements neither light exclusion nor thermal relaxation was applied to the samples. Nuclear magnetic resonance (NMR) spectra of the *E*-isomers of the synthetic azobenzene derivatives were recorded after storage of the samples at 40 °C (overnight in $CDCl_3$) or 50 °C (3 days in MeCN-d_3 or DMSO-d_6). Proton (^1H) and carbon (^{13}C) NMR spectra were measured at 300 K on Bruker DRX 500 and Bruker Avance 600 instruments. Spectra were referenced to tetramethylsilane (TMS) or the appropriate solvent signal. 2D NMR techniques (^1H,^1H COSY, ^1H,^{13}C HSQC, ^1H,^{13}C HMBC) were used for unequivocal assignment of the spectra. Data were reported as follows: Chemical shift δ in ppm, multiplicities of signals as s (singlet), d (doublet), t (triplet), q (quartet), qi (quintet), m (multiplet), coupling constants in hertz (Hz), and integration. The spectrometer Spectrum 100 FT-IR from Perkin-Elmer was used to record infrared (IR) spectra. The measurements were performed in reflection mode with a Golden-Gate-Diamond-ATR-unit A531-G. High-resolution (HR) ESI mass spectra were recorded on a ThermoFisher Orbitrap, Q Exactive Plus, from Thermo Scientific. UV–vis spectra were recorded using a Lambda 14 UV/Vis spectrometer from Perkin-Elmer or a UV-2600 i from Shimadzu equipped with a Büchi thermostat using Brand disposable semi-micro cuvettes with a path length of 1 cm. For NMR or UV–vis experiments, photoirradiation was performed either with a UV-LED (Nichia Corporation, emitting 365 nm light, with a FWMH of 10 nm and an intensity of 235 mW/cm² per LED, 1 LED), a blue LED (in house built, emitting 435 nm light, with a FWMH of 13 nm and an intensity of 130 mW/cm² per LED, 2 LEDs), or a green LED (Nichia Corporation, emitting 520 nm light, with a FWMH of 30 nm and an intensity of 190 mW/cm^2 per LED, 5 LEDs).

1.2 Experimental procedures

For NMR assignments numbering and indices were used as explained in **Scheme S3**.

General procedure for the Buchwald–Hartwig–Migita cross-coupling: Thiol (1 equiv), aryl iodide (1 equiv or 2 equiv) and XantPhos-Pd-G3 (5 mol %) were dissolved in degassed dry THF and treated with triethyl amine (1 equiv) at the suitable temperature. After an appropriate time (according to TLC analysis), the reaction mixture was diluted with ethyl acetate and the organic phase was washed with 1 M HCl (1 × 50 mL) and brine (1 × 50 mL). The organic phase was dried over $MgSO_4$, filtered and the solvent was removed under reduced pressure. Dry load column chromatography (cyclohexane/ethyl acetate 5:1 to 1:1) yielded the corresponding product.

General procedure for deacylation: The acetylated compound was dissolved in a mixture of methanol/dichloromethane 2:1 (3 mL) and was treated with sodium methylate (24 µL, 5 M in methanol) at room temperature. The mixture was stirred for 3 h at room temperature and complete conversion of the starting material was controlled by TLC. It was neutralized with Amberlite IR120®, filtered and the solvent was removed under reduced pressure.

2,3,4,6-Tetra-*O*-acetyl-1-*S*-acetyl-1-thio-α-D-mannopyranoside (7): The trichloroacetimidate **6** [1] (2.00 g, 4.06 mmol, 1.00 equiv) and activated molecular sieves (3 Å, 1.1 g) were suspended in dry dichloromethane (50 mL) and under stirring thioacetic acid (618 mg, 572 µL, 8.12 mmol, 2.00 equiv) followed by boron trifluoride etherate (144 mg, 125 µL, 1.01 mmol, 0.25 equiv) were added at −10 °C. The reaction mixture was allowed to warm to room temperature while stirring for 18 h. The reaction was quenched with satd. aq. $NaHCO_3$ (50 mL) and filtered over Celite®. The organic phase was washed with satd. aq. $NaHCO_3$ (3 × 50 mL) and brine (3 × 50 mL), dried over $MeSO_4$, filtered and the solvent was removed under reduced pressure. After purification by column chromatography (cyclohexane/ethyl acetate 1:0 to 1:1) a yellow syrup was obtained (1.60 g, 3.93 mmol, 97%). R_f=0.17 (cyclohexane/ethyl acetate 3:1); $[\alpha]_D^{20}$=+71.1 (*c*=1.0 in dichloromethane); ^{1}H NMR (500 MHz, $CDCl_3$): δ = 5.96 (dd, $^3J_{1,2}$ = 1.9 Hz, 1H, H-1), 5.34 (dd~t, $^3J_{3,4}$ = $^3J_{4,5}$ = 9.6 Hz, 1H, H-4), 5.33 (dd, $^3J_{2,3}$ = 3.8 Hz, $^3J_{1,2}$ = 2.3 Hz, 1H, H-2), 5.10 (dd, $^3J_{3,4}$ = 10.0 Hz, $^3J_{2,3}$ = 3.3 Hz, 1H, H-3), 4.29 (dd, $^2J_{6a,6b}$ = 12.4 Hz, $^3J_{5,6a}$ = 4.8 Hz, 1H, H-6a), 4.08 (dd, $^2J_{6a,6b}$ = 12.5 Hz, $^3J_{5,6b}$ = 2.5 Hz, 1H, H-6b), 3.93 (ddd, $^3J_{4,5}$ = 10.0 Hz, $^3J_{5,6a}$ = 4.7 Hz, $^3J_{5,6b}$ = 2.7 Hz, 1H, H-6b), 2.43 (s, 3H, $SCOCH_3$), 2.19, 2.09, 2.05, 2.00 (each s, each 3H,12H, 4 $COCH_3$) ppm; ^{13}C NMR (126 MHz, $CDCl_3$): δ = 190.36 (S*C*OCH3), 170.63, 169.89, 169.82, 169.51 (4 *C*OCH3), 80.16 (C-1), 72.43 (C-5), 70.98 (C-2), 69.84 (C-3), 65.65 (C-4), 62.13 (C-6), 31.25 (SCO*C*H3), 20.87, 20.70, 20.65, 20.59 (4 CO*C*H3) ppm; IR (ATR): ν_{max}/cm^{-1} = 2969 (w), 1743 (vs), 1713 (s), 1432 (w), 1368 (s), 1219 (vs), 1111 (s), 1050 (s); HRMS (ESI): *m*/*z*: calcd. for $C_{16}H_{22}O_{10}S+NH_4^+$: 424.12720 $[M+NH_4]^+$; found: 424.12641.

4-(2,2’,6,6’-Tetrafluoro-4’-iodo-azobenzene) 2,3,4,6-tetra-*O*-acetyl-1-thio-α-D-mannopyranoside (10): The thioacetate **7** (350 mg, 861 µmol, 1.00 equiv) and sodium carbonate (86.7 mg, 818 µmol, 0.95 equiv) were dissolved in dry methanol (15 mL) and the mixture was stirred for 2.5 h at room temperature. It was neutralized with Amberlite IR120®, filtered and the solvent was removed under reduced pressure. The obtained crude product and 2,2’,6,6’-tetrafluoro-4,4’-diiodoazobenzene (**9**) [2] (872 mg, 1.72 mmol, 2.00 equiv) were treated according to the general procedure of the Buchwald–Hartwig–Migita cross-coupling with a temperature of −78 °C over 2 h in degassed dry THF (20 mL). Column chromatography (cyclohexane/ethyl acetate 5:1 to 1:1) yielded the product as a red amorphous solid (199 mg, 267 µmol, 31%). R_f=0.33 (cyclohexane/ethyl acetate 3:1); $[\alpha]_D^{20}$=+62.8 (*c*=0.8 in dichloromethane); the ^{1}H, ^{1}H NOESY NMR spectrum shows no cross peaks neither between H-1 and H-3 nor between H-1 and H-5, confirming the anomeric α-configuration. *E*-isomer: ^{1}H NMR (600 MHz, DMSO-d_6): δ = 7.87 – 7.83 (m, 2H, H_c), 7.58 – 7.54 (m, 2H, $H_{c'}$), 6.19 (d, $^3J_{1,2}$ = 1.5 Hz, 1H, H-1), 5.40 (dd, $^3J_{2,3}$ = 3.3 Hz, $^3J_{1,2}$ = 1.6 Hz, 1H, H-2), 5.18 (dd~t, $^3J_{3,4}$ = $^3J_{4,5}$ = 9.9 Hz, 1H, H-4), 5.13 (dd, $^3J_{3,4}$ = 10.1 Hz, $^3J_{2,3}$ = 3.3 Hz, 1H, H-3), 4.35 (ddd, $^3J_{4,5}$ = 9.2 Hz, $^3J_{5,6a}$ = 6.2 Hz, $^3J_{5,6b}$ = 2.3 Hz, 1H, H-5), 4.20 (dd, $^2J_{6a,6b}$ = 12.3 Hz, $^3J_{5,6a}$ = 6.3 Hz, 1H, H-6a), 4.04 (dd, $^2J_{6a,6b}$ = 12.4 Hz, $^3J_{5,6b}$ = 2.3 Hz, 1H, H-6b), 2.15, 2.06, 1.99, 1.92 (each s, each 3H, 12H, 4 $COCH_3$) ppm; ^{13}C NMR (151 MHz, DMSO-d_6): δ = 169.85, 169.74, 169.55, 169.48 (4 *C*OCH3), 154.62 (dd, $^1J_{C,F}$ = 261.1 Hz, $^3J_{C,F}$ = 4.8 Hz, 2 C_b), 154.23 (dd, $^1J_{C,F}$ = 266.5 Hz, $^3J_{C,F}$ = 3.8 Hz, 2 $C_{b'}$), 139.70 (t, $^3J_{C,F}$ = 11.5 Hz, 2 C_d), 130.30 (t, $^2J_{C,F}$ = 10.0 Hz, 2 C_a), 129.15 (t, $^2J_{C,F}$ = 10.0 Hz, 2 $C_{a'}$), 122.53 (d, $^2J_{C,F}$ = 22.9 Hz, 2 C_c), 113.84 (d, $^2J_{C,F}$ = 22.6 Hz, 2 $C_{c'}$), 98.01 (t, $^3J_{C,F}$ = 10.4 Hz, 2 $C_{d'}$), 82.40 (C-1), 69.74 (C-5), 69.20 (C-2), 68.83 (C-3), 65.34 (C-4), 61.79 (C-6), 20.60, 20.45, 20.38, 20.24 (4 CO*C*H3) ppm; ^{19}F NMR (471 MHz, DMSO-d_6): δ = -119.64 ($F_{b/b'}$), -120.36 ($F_{b/b'}$) ppm; IR (ATR): ν_{max}/cm^{-1} = 3082 (w), 2936 (w), 1747 (vs), 1602 (s), 1564 (m), 1416 (m), 1369 (m), 1223 (vs), 1104 (w), 1046 (vs); HRMS (ESI): *m*/*z*: calcd. for $C_{26}H_{23}F_4IN_2O_9S+H^+$: 743.01779 $[M+H]^+$; found: 743.01745.

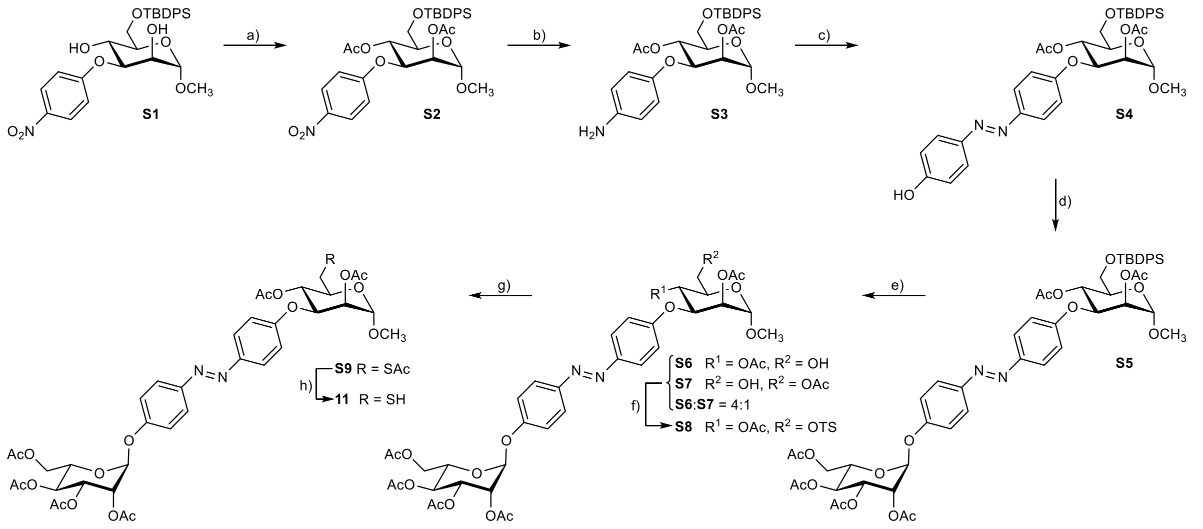

Scheme S1: Synthesis of azobenzene bis-6-thiomannoside **11**. Reagents and conditions: a) Ac_2O, pyridine, rt, 16 h, 98%; b) H_2, Pd/C (10%), MeOH, rt, 18 h, 53%; c) (i) 1 M HCl, $NaNO_2$, H_2O, MeOH, 0 °C to rt, 1.5 h; (ii) PhOH, KOH, MeOH, 0 C to rt, 2 h, 92%; d) $BF_3{\cdot}Et_2O$, HSAc, dry CH_2Cl_2, 0 °C to rt, 18 h, 96%; e) TBAF (1 M in THF), AcOH, THF, 0 °C to rt, 20 h (quant., **S6**:**S7** = 4:1); f) TsCl, I_2, DMAP, dry pyridine, rt, 2 h, 57% over 2 steps; g) KSAc, MeCN, 85 °C, 5 h, rt, 18 h, 96%; h) DTT, NEt_3, DMA, rt, 18 h, quant. DTT: 1,4-dithio-D-threitol, DMA: dimethylacetamide.

Methyl 2,4-di-*O*-acetyl-6-*O*-(*tert*-butyldiphenyl)silyl-3-*O*-*p*-nitrophenyl-α-D-mannopyranoside (S2): The 2,4-OH-free mannoside **S1** [3] (776 mg, 1.40 mmol, 1.00 equiv) was dissolved in a mixture of dry pyridine and acetic anhydride 2:1 (18 mL) and stirred for 16 h at room temperature. The solution was diluted with ethyl acetate (50 mL) and the organic phase was washed with 1 M HCl (3 × 25 mL), satd. aq. $NaHCO_3$ (3 × 25 mL) and brine (3 × 25 mL). Then, the organic phase was dried over $MgSO_4$, filtered, the solvent was removed under reduced pressure and codestillation (3 × 100 mL of toluene) delivered the raw product. After purification by column chromatography (cyclohexane/ethyl acetate 3:1) the product was obtained as a colorless firm foam (873 mg, 1.37 mmol, 98%). R_f=0.66 (cyclohexane/ethyl acetate 3:1); $[\alpha]_D^{20}$=+64.5 (*c*=0.9 in dichloromethane); ^{1}H NMR (600 MHz, $CDCl_3$): δ = 8.20 – 8.16 (m, 2H, H_{meta}), 7.73 – 7.66 (m, 4H, $H_{TBDPS\text{-}ortho}$), 7.45 – 7.41 (m, 2H, $H_{TBDPS\text{-}para}$), 7.40 – 7.36 (m, 4H, $H_{TBDPS\text{-}meta}$), 7.02 – 6.96 (m, 2H, H_{ortho}), 5.54 (dd~t, $^3J_{3,4}$ = $^3J_{4,5}$ = 9.8 Hz, 1H, H-4), 5.34 (dd, $^3J_{2,3}$ = 3.3 Hz, $^3J_{1,2}$ = 1.9 Hz, 1H, H-2), 4.85 (dd, $^3J_{3,4}$ = 9.6 Hz, $^3J_{2,3}$ = 3.3 Hz, 1H, H-3), 4.81 (d, $^3J_{1,2}$ = 1.9 Hz, 1H, H-1), 3.85 (ddd, $^3J_{4,5}$ = 9.8 Hz, $^3J_{5,6a}$ = 5.3 Hz, $^3J_{5,6b}$ = 2.1 Hz, 1H, H-5), 3.80 (dd, $^2J_{6a,6b}$ = 11.3 Hz, $^3J_{5,6a}$ = 5.4 Hz, 1H, H-6a), 3.73 (dd, $^2J_{6a,6b}$ = 11.3 Hz, $^3J_{5,6b}$ = 2.1 Hz, 1H, H-6b), 3.45 (s, 3H, OCH_3), 2.13, 1.77 (each s, each 3H, 6H, 2 $COCH_3$), 1.08 (s, 9H, $C(CH_3)_3$) ppm; ^{13}C NMR (151 MHz, $CDCl_3$): δ = 170.08, 169.24 (2 $COCH_3$), 162.70 (C_{ipso}), 142.19 (C_{para}), 135.75, 135.65 (4 $C_{TBDPS\text{-}ortho}$), 133.25, 133.18 (2 $C_{TBDPS\text{-}ipso}$), 129.74 (2 $C_{TBDPS\text{-}para}$), 127.69, 127.65 (4 $C_{TBDPS\text{-}meta}$), 125.92 (C_{meta}), 115.80 (C_{ortho}), 98.22 (C-1), 74.91 (C-3), 71.60 (C-5), 68.97 (C-2), 67.11 (C-4), 62.81 (C-6), 55.20 (OCH_3), 26.70 ($C(CH_3)_3$), 20.85, 20.59 (2 $COCH_3$), 19.26 ($C(CH_3)_3$) ppm; IR (ATR): ν_{max}/cm^{-1} = 3073 (w), 2932 (m), 2857 (m), 1751 (vs), 1609 (m), 1592 (s), 1517 (s), 1493 (m), 1342 (vs), 1256 (vs), 1218 (vs), 1109 (vs), 1045 (vs), 864 (s), 702 (vs); HRMS (ESI): m/z: calcd. for $C_{33}H_{39}NO_{10}Si$+NH_4^+: 655.26815 $[M+NH_4]^+$; found: 655.26733.

Methyl 2,4-di-*O*-acetyl-6-*O*-(*tert*-butyldiphenyl)silyl-3-*O*-*p*-aminophenyl-α-D-mannopyranoside (S3): The *p*-nitrophenyl-substituted compound **S2** (1.83 g, 2.88 mmol, 1.00 equiv) was dissolved in a baffled flask and dissolved in dry methanol (100 mL). Under nitrogen atmosphere palladium (10% on carbon, 765 mg, 719 µmol, 0.25 equiv) was added, a hyrogen atmosphere was set and the reaction mixture was vigorously stirred for 18 h at room temperature. It was filtered over Celite® and the solvent was removed under reduced pressure. After purification by column chromatography (cyclohexane/ethyl acetate 3:1 to 1:1) the product was obtained as a bright red firm foam (929 mg, 1.53 mmol, 53%). R_f=0.21 (cyclohexane/ethyl acetate 3:1); $[\alpha]_D^{20}$=+50.0 (*c*=0.7 in dichloromethane); ^{1}H NMR (500 MHz, $CDCl_3$): δ = 7.73 – 7.65 (m, 4H, $H_{TBDPS\text{-}ortho}$), 7.46 – 7.33 (m, 6H, $H_{TBDPS\text{-}para}$, $H_{TBDPS\text{-}meta}$), 6.77 – 6.71 (m,

2H, H$_{ortho}$), 6.61 – 6.55 (m, 2H, H$_{meta}$), 5.41 (dd~t, $^3J_{3,4}$ = $^3J_{4,5}$ = 9.8 Hz, 1H, H-4), 5.27 (dd, $^3J_{2,3}$ = 3.4 Hz, $^3J_{1,2}$ = 1.8 Hz, 1H, H-2), 4.76 (d, $^3J_{1,2}$ = 1.9 Hz, 1H, H-1), 4.45 (dd, $^3J_{3,4}$ = 9.8 Hz, $^3J_{2,3}$ = 3.4 Hz, 1H, H-3), 3.83 – 3.77 (m, 2H, H-5, H-6a), 3.74 – 3.68 (m, 1H, H-6b), 3.40 (s, 3H, OCH_3), 2.14, 1.83 (each s, each 3H, 6H, 2 $COCH_3$), 1.07 (s, 9H, $C(CH_3)_3$) ppm; ^{13}C NMR (126 MHz, $CDCl_3$): δ = 170.41, 169.61 (2 *C*OCH$_3$), 150.92 (C$_{ipso}$), 141.41 (C$_{para}$), 135.74, 135.65 (4 C$_{TBDPS\text{-}ortho}$), 133.38, 133.32 (2 C$_{TBDPS\text{-}ipso}$), 129.66 (2 C$_{TBDPS\text{-}para}$), 127.65, 127.61 (4 C$_{TBDPS\text{-}meta}$), 118.78 (C$_{ortho}$), 116.16 (C$_{meta}$), 98.33 (C-1), 76.55 (C-3), 71.75 (C-5), 69.68 (C-2), 67.74 (C-4), 63.17 (C-6), 55.02 (OCH_3), 26.71 (C(*C*H$_3$)$_3$), 21.02, 20.77 (2 CO*C*H$_3$), 19.24 (*C*(CH$_3$)$_3$) ppm; IR (ATR): ν_{max}/cm^{-1} = 3460 (w), 3369 (w), 2931 (m), 2857 (w), 1746 (vs), 1508 (vs), 1428 (w), 1371 (m), 1219 (vs), 1110 (vs), 1077 (vs), 1044 (vs), 824 (s), 702 (s); HRMS (ESI): m/z: calcd. for $C_{33}H_{41}NO_8Si+NH_4^+$: 625.29397 [*M*+NH$_4$]$^+$; found: 625.29346.

Methyl 2,4-di-*O*-acetyl-6-*O*-(*tert*-butyldiphenyl)silyl-3-*O*-(4'-hydroxyazobenzene)-α-D-manno-pyranoside (S4): The *p*-aminophenyl-substituted compound **S3** (892 mg, 1.47 mmol, 1.00 equiv) was dissolved in methanol (15 mL) and 1 M HCl (7.3 mL, 5.00 equiv) was added. To this, an aq. solution of sodium nitrite (152 mg, 2.20 mmol, 1.50 equiv in 2 mL dist. water) was added dropwise at 0 °C. The reaction mixture was stirred mechanically at 0 °C for 10 min, followed by 1 h at room temperature. Then, a solution of phenol (553 mg, 5.87 mmol, 4.00 equiv) and potassium hydroxide (330 mg, 5.87 mmol, 4.00 equiv) in methanol (15 mL) cooled to 0 °C was added dropwise to the reaction mixture at 0 °C and it was further stirred for 2 h at room temperature. The reaction mixture was diluted with ethyl acetate (100 mL) and the organic phase was washed with satd. aq. $Na_2S_2O_3$ (3 × 50 mL), satd. aq. $NaHCO_3$ (2 × 50 mL) and brine (2 × 50 mL). The organic phase was dried over $MgSO_4$, filtered and the solvent was removed under reduced pressure. Purification by column chromatography (cyclohexane/ethyl acetate 3:1) gave the product as a yellow foam (963 mg, 1.35 mmol, 92%). R_f=0.34 (cyclohexane/ethyl acetate 3:1); $[\alpha]_D^{20}$=+70.6 (*c*=0.5 in dichloromethane); ^{1}H NMR (600 MHz, $CDCl_3$): δ = 7.84 – 7.76 (m, 4H, H$_b$, H$_{b'}$), 7.73 – 7.67 (m, 4H, H$_{TBDPS\text{-}ortho}$), 7.45 – 7.35 (m, 6H, H$_{TBDPS\text{-}para}$, H$_{TBDPS\text{-}meta}$), 7.04 – 6.99 (m, 2H, H$_c$), 6.90 – 6.85 (m, 2H, H$_{c'}$), 5.73 (s, 1H, OH), 5.52 (dd~t, $^3J_{3,4}$ = $^3J_{4,5}$ = 9.8 Hz, 1H, H-4), 5.39 (dd, $^3J_{2,3}$ = 3.4 Hz, $^3J_{1,2}$ = 1.8 Hz, 1H, H-2), 4.82 (d, $^3J_{1,2}$ = 1.6 Hz, 1H, H-1), 4.81 (d, $^3J_{2,3}$ = 3.3 Hz, $^3J_{3,4}$ = 9.7 Hz, 1H, H-3), 3.86 (ddd, $^3J_{4,5}$ = 9.9 Hz, $^3J_{5,6a}$ = 5.6 Hz, $^3J_{5,6b}$ = 2.0 Hz, 1H, H-5), 3.82 (dd, $^2J_{6a,6b}$ = 11.2 Hz, $^3J_{5,6a}$ = 5.6 Hz, 1H, H-6a), 3.73 (dd, $^2J_{6a,6b}$ = 11.2 Hz, $^3J_{5,6b}$ = 2.0 Hz, 1H, H-6b), 3.46 (s, 3H, OCH_3), 2.16, 1.78 (each s, each 3H, 6H, 2 $COCH_3$), 1.08 (s, 9H, $C(CH_3)_3$) ppm; ^{13}C NMR (151 MHz, $CDCl_3$): δ = 170.57, 169.66 (2 *C*OCH$_3$), 159.67 (C$_d$), 158.13 (C$_{d'}$), 147.70 (C$_a$), 147.03 (C$_{a'}$), 135.76, 135.66 (4 C$_{TBDPS\text{-}ortho}$), 133.32, 133.25 (4 C$_{TBDPS\text{-}ipso}$), 129.71 (2 C$_{TBDPS\text{-}para}$), 127.68, 127.64 (4 C$_{TBDPS\text{-}meta}$), 124.70 (C$_{b'}$), 124.30 (C$_b$), 116.46 (C$_c$), 115.72 (C$_{c'}$), 98.31 (C-1), 74.76 (C-3), 71.68 (C-5), 69.56 (C-2), 67.51 (C-4), 62.98 (C-6), 55.16 (OCH_3), 26.72 (C(*C*H$_3$)$_3$), 20.98, 20.67 (2 CO*C*H$_3$), 19.26 (*C*(CH$_3$)$_3$) ppm; IR (ATR): ν_{max}/cm^{-1} = 3414 (br), 2931 (m), 2857 (w), 1752 (s), 1725 (s), 1588 (s), 1496 (s), 1372 (s), 1219 (vs), 1140 (s), 1111 (s), 1046 (s), 844 (m), 702 (m); HRMS (ESI): m/z: calcd. for $C_{39}H_{44}N_2O_8Si+H^+$: 713.28888 [*M*+H]$^+$; found: 713.28784.

Methyl 2,4-di-*O*-acetyl-6-*O*-(*tert*-butyldiphenyl)silyl-3-*O*-[4'-(2,3,4,6-tetra-*O*-acetyl-α-D-manno-pyranosyloxy)azobenzene]-α-D-mannopyranoside (S5): The azobenzene derivative **S4** (938 mg, 1.32 mmol, 1.00 equiv), trichloroacetimidate **6** [1] (790 mg, 1.61 mmol, 1.22 equiv) and activated molecular sieves (3 Å) were suspended in dry dichloromethane (48 mL). At 0 °C, boron trifluoride etherate (0.25 mL, 1.97 mmol, 1.50 equiv) was added and the reaction mixture was allowed to warm to room temperature while stirring for 18 h. Then, satd. aq. $NaHCO_3$ (100 mL) was added and it was filtered over Celite®. The phases were separated and the organic phase was washed with satd. aq. $NaHCO_3$ (3 × 50 mL) and brine (3 × 50 mL). It was dried over $MgSO_4$, filtered and the solvent was removed under reduced pressure. Purification by column chromatography (cyclohexane/ethyl acetate 3:1) yielded the product as a yellow foam (877 mg, 841 µmol, 64%). The isolated starting material was further treated accordingly to the described procedure with trichloroacetimidate **6** (1.50 equiv) and boron trifluoride etherate (1.50 equiv) to increase the total yield of the product (1.32 g, 1.27 mmol, 96%). R_f=0.15 (cyclohexane/ethyl acetate 3:1); $[\alpha]_D^{20}$=+88.6 (*c*=1.0 in dichloromethane); ^{1}H NMR (600 MHz, $CDCl_3$) δ 7.89 – 7.83 (m, 4H, H$_{b'}$, H$_b$), 7.73 – 7.67 (m, 4H, H$_{TBDPS\text{-}ortho}$), 7.46 – 7.35 (m, 6H, H$_{TBDPS\text{-}para}$, H$_{TBDPS\text{-}meta}$), 7.23 – 7.19 (m, 2H, H$_{c'}$), 7.06 – 7.00 (m, 2H, H$_c$), 5.61 (d, $^3J_{1',2'}$ = 1.8 Hz, 1H, H-1'), 5.58 (dd, $^3J_{3',4'}$ = 10.0 Hz, $^3J_{2',3'}$ = 3.5 Hz, 1H, H-3'), 5.52 (dd~t, $^3J_{3,4}$ = $^3J_{4,5}$ = 9.8 Hz, 1H, H-4), 5.48 (dd, $^3J_{2',3'}$ = 3.6 Hz, $^3J_{1',2'}$ =

1.9 Hz, 1H, H-2'), 5.39 (dd~t, $^3J_{3',4'}$ = $^3J_{4',5'}$ = 10.2 Hz, 1H, H-4'), 5.36 (dd, $^3J_{2,3}$ = 3.4 Hz, $^3J_{1,2}$ = 1.9 Hz, 1H, H-2), 4.82 (d, $^3J_{1,2}$ = 1.7 Hz, 1H, H-1), 4.81 (d, $^3J_{3,4}$ = 9.6 Hz, $^3J_{2,3}$ = 3.3 Hz, 1H, H-3), 4.30 (dd, $^2J_{6'a,6'b}$ = 12.4 Hz, $^3J_{5',6'a}$ = 5.6 Hz, 1H, H-6'a), 4.13 – 4.07 (m, 2H, H-5', H-6'b), 3.86 (ddd, $^3J_{4,5}$ = 9.8 Hz, $^3J_{5,6a}$ = 5.7 Hz, $^3J_{5,6b}$ = 1.9 Hz, 1H, H-5), 3.82 (dd, $^2J_{6a,6b}$ = 11.2 Hz, $^3J_{5,6a}$ = 5.6 Hz, 1H, H-6a), 3.73 (dd, $^2J_{6a,6b}$ = 11.2 Hz, $^3J_{5,6b}$ = 2.0 Hz, 1H, H-6b), 3.46 (s, 3H, OCH_3), 2.22, 2.15, 2.06, 2.05, 2.03, 1.77 (each s, each 3H, 18H, 6 $COCH_3$), 1.08 (s, 9H, $C(CH_3)_3$) ppm; ^{13}C NMR (151 MHz, $CDCl_3$): δ = 170.52, 170.21, 169.96, 169.93, 169.73, 169.45 (6 *C*OCH$_3$), 160.06 (C_d), 157.24 ($C_{d'}$), 148.42 ($C_{a'}$), 147.58 (C_a), 135.73, 135.64 (4 $C_{TBDPS\text{-}ortho}$), 133.30, 133.23 (4 $C_{TBDPS\text{-}ipso}$), 129.68, (2 $C_{TBDPS\text{-}para}$), 127.65, 127.62 (4 $C_{TBDPS\text{-}meta}$), 124.48 (C_b), 124.30 ($C_{b'}$), 116.70 ($C_{c'}$), 116.50 (C_c), 98.26 (C-1), 95.67 (C-1'), 74.77 (C-3), 71.66 (C-5), 69.48 (C-2), 69.38 (C-5'), 69.26 (C-2'), 68.79 (C-3'), 67.46 (C-4), 65.86 (C-4'), 62.97 (C-6), 62.06 (C-6'), 55.12 (OCH_3), 26.69 (C(*C*H$_3$)$_3$), 20.92, 20.86, 20.70, 20.68, 20.63 (6 CO*C*H$_3$), 19.23 (*C*(CH$_3$)$_3$) ppm; IR (ATR): ν_{max}/cm^{-1} = 2931 (w), 2857 (w), 1747 (vs), 1597 (m), 1583 (m), 1496 (m), 1368 (m), 1213 (vs), 1131 (m), 1111 (m), 1035 (s), 846 (m), 703 (m); HRMS (ESI): m/z: calcd. for $C_{53}H_{62}N_2O_{18}Si+H^+$: 1043.38397 [*M*+H]$^+$; found: 1043.38365.

Methyl 2,4-di-*O*-acetyl-3-*O*-[4'-(2,3,4,6-tetra-*O*-acetyl-α-D-mannopyranosyloxy)azobenzene]-6-*O*-tosyl-α-D-mannopyranoside (S8): The TBDPS-protected mannoside **S5** (1.26 g, 1.21 mmol, 1.00 equiv) was dissolved in THF (65 mL) and at 0 °C first acetic acid (0.35 mL, 6.04 mmol, 5.07 equiv) followed by tetrabutylammonium fluoride (3.0 mL, 3.00 mmol, 2.48 equiv, 1 M in THF) were added. The mixture was stirred for 30 min at 0 °C followed by 20 h of stirring at room temperature. To the solution satd. aq. $NaHCO_3$ (100 mL) was added and it was diluted with ethyl acetate (200 mL). The phases were separated and the organic phase was washed with satd. aq. $NaHCO_3$ (2 × 50 mL) and brine (3 × 50 mL), dried over $MgSO_4$ and filtered. The solvent was removed under reduced pressure and column chromatography (cyclohexane/ethyl acetate 1:1 to 0:1) gave the desilylated product **S6** as a mixture together with the 4-position-unprotected by-product (quant. **S6**:**S7** = 4:1). The mixture of **S6**:**S7**, tosyl chloride (453 mg, 2.38 mmol, 1.97 equiv), iodine (754 mg, 2.97 mmol, 2.46 equiv) and DMAP (145 mg, 1.19 mmol, 0.98 equiv) were dissolved in dry pyridine (30 mL) and stirred for 2 h at room temperature. The reaction mixture was diluted with ethyl acetate (300 mL) and washed with 1 M HCl (1 × 100 mL), brine (1 × 100 mL), satd. aq. $Na_2S_2O_3$ (3 × 100 mL), 1 M HCl (3 × 50 mL) and brine (3 × 50 mL). The organic phase was dried over $MgSO_4$, filtered and the solvent was removed under reduced pressure. Purification by column chromatography (cyclohexane/ethyl acetate 1:1) gave the product as a yellow foam (655 mg, 683 µmol, 57%).

S6: R_f=0.06 (cyclohexane/ethyl acetate 1:1); $[\alpha]_D^{20}$=+103.9 (*c*=1.0 in dichloromethane); ^{1}H NMR (500 MHz, $CDCl_3$): δ = 7.91 – 7.83 (m, 4H, H_b, $H_{b'}$), 7.24 – 7.18 (m, 2H, $H_{c'}$), 7.09 – 7.01 (m, 2H, H_c), 5.61 (d, $^3J_{1',2'}$ = 1.8 Hz, 1H, H-1'), 5.58 (dd, $^3J_{3',4'}$ = 10.0 Hz, $^3J_{2',3'}$ = 3.6 Hz, 1H, H-3'), 5.48 (dd, $^3J_{2',3'}$ = 3.5 Hz, $^3J_{1',2'}$ = 1.9 Hz, 1H, H-2'), 5.43 (dd~t, $^3J_{3,4}$ = $^3J_{4,5}$ = 9.8 Hz,1H, H-4), 5.40 (dd, $^3J_{2,3}$ = 3.5 Hz, $^3J_{1,2}$ = 1.8 Hz, 1H, H-2), 5.39 (dd~t, $^3J_{3',4'}$ = $^3J_{4',5'}$ = 10.1 Hz, 1H, H-4'), 4.87 (dd, $^3J_{3,4}$ = 9.7 Hz, $^3J_{2,3}$ = 3.4 Hz, 1H, H-3), 4.82 (d, $^3J_{1,2}$ = 1.8 Hz, 1H, H-1), 4.30 (dd, $^2J_{6'a,6'b}$ = 12.3 Hz, $^3J_{5',6'a}$ = 5.5 Hz, 1H, H-6'a), 4.13 – 4.07 (m, 2H, H-5', H-6'b), 3.82 – 3.65 (m, 3H, H-5, H-6a, H-6b), 3.45 (s, 3H, OCH_3), 2.45 (dd, $^3J_{6a,OH}$ = 8.8 Hz, $^3J_{6b,OH}$ = 5.3 Hz, 1H, OH-6), 2.22, 2.18, 2.06, 2.05, 2.04, 1.98 (each s, each 3H, 18H, 6 $COCH_3$) ppm; ^{13}C NMR (126 MHz, $CDCl_3$): δ = 170.89, 170.51, 170.11, 169.96, 169.93, 169.72 (6 *C*OCH$_3$), 159.93 (C_d), 157.30 ($C_{d'}$), 148.41 ($C_{a'}$), 147.71 (C_a), 124.52 (C_b), 124.33 ($C_{b'}$), 116.71 ($C_{c'}$), 116.58 (C_c), 98.67 (C-1), 95.68 (C-1'), 74.24 (C-3), 70.69 (C-5), 69.39 (C-5), 69.27 (C-2'), 69.25 (C-2), 68.78 (C-3'), 67.80 (C-4), 65.87 (C-4'), 62.06 (C-6'), 61.36 (C-6), 55.38 (OCH_3), 20.94, 20.87, 20.75, 20.68 (6 CO*C*H$_3$) ppm; IR (ATR): ν_{max}/cm^{-1} = 3508 (br), 2937 (br), 1746 (vs), 1597 (m), 1583 (m), 1496 (m), 1369 (m), 1215 (vs), 1130 (s), 1084 (s), 1033 (s), 846 (m), 733 (m); HRMS (ESI): m/z: calcd. for $C_{37}H_{44}N_2O_{18}+H^+$: 805.26619 [*M*+H]$^+$; found: 805.26559.

S8: R_f=0.46 (cyclohexane/ethyl acetate 1:1); $[\alpha]_D^{20}$=+94.2 (*c*=0.8 in dichloromethane); ^{1}H NMR (500 MHz, $CDCl_3$): δ = 7.89 – 7.83 (m, 4H, H_b, $H_{b'}$), 7.83 – 7.79 (m, 2H, H_{meta}), 7.39 – 7.33 (m, 2H, H_{ortho}), 7.24 – 7.17 (m, 2H, $H_{c'}$), 7.04 – 6.97 (m, 2H, H_c), 5.61 (d, $^3J_{1',2'}$ = 1.9 Hz, 1H, H-1'), 5.58 (dd, $^3J_{3',4'}$ = 10.0 Hz, $^3J_{2',3'}$ = 3.5 Hz, 1H, H-3'), 5.48 (dd, $^3J_{2',3'}$ = 3.5 Hz, $^3J_{1',2'}$ = 1.8 Hz, 1H, H-2'), 5.38 (dd~t, $^3J_{3',4'}$ = $^3J_{4',5'}$ = 10.1 Hz, 1H, H-4'), 5.34 (dd, $^3J_{2,3}$ = 3.2 Hz, $^3J_{1,2}$ = 2.0 Hz, 1H, H-2), 5.32 (dd~t, $^3J_{3,4}$ = $^3J_{4,5}$ = 9.9 Hz, 1H, H-4), 4.79 (dd, $^3J_{3,4}$ = 9.6 Hz, $^3J_{2,3}$ = 3.4 Hz, 1H, H-3), 4.72 (d, $^3J_{1,2}$ = 1.8 Hz, 1H, H-1), 4.32 – 4.27 (m, 1H, H-6'a), 4.17 – 4.13 (m, 2H, H-6a, H-6b), 4.13 – 4.02 (m, 3H, H-5, H-5', H-6'b), 3.41 (s, 3H,

OCH_3), 2.46 (s. 3H, CCH_3), 2.22, 2.14, 2.06, 2.05, 2.03, 1.90 (each s, each 3H, 18H, 6 $COCH_3$) ppm; ^{13}C NMR (126 MHz, $CDCl_3$): δ = 170.51, 169.99, 169.96, 169.93, 169.77, 169.71 (6 *C*OCH_3), 159.74 (C_d), 157.31 ($C_{d'}$), 148.38 ($C_{a'}$), 147.74 (C_a), 145.00 (C_{para}), 132.76 (C_{ipso}), 129.83 (C_{meta}), 128.05 (C_{ortho}), 124.51 (C_b), 124.34 ($C_{b'}$), 116.71 ($C_{c'}$), 116.51 (C_c), 98.38 (C-1), 95.68 (C-1'), 74.26 (C-3), 69.39 (C-5'), 69.26 (C-2'), 69.10 (C-2), 68.78 (C-3'), 68.68 (C-5), 68.60 (C-6), 67.57 (C-4), 65.86 (C-4'), 62.06 (C-6'), 55.48 (OCH_3), 29.70 ($PhCH_3$), 21.66, 20.87, 20.68, 20.63 (6 $COCH_3$) ppm; IR (ATR): ν_{max}/cm^{-1} = 2961 (w), 1749 (vs), 1597 (m), 1584 (m), 1496 (m), 1368 (m), 1219 (vs), 1177 (m), 1133 (m), 1085 (m), 1050 (m), 847 (w); HRMS (ESI): m/z: calcd. for $C_{44}H_{50}N_2O_{20}S+H^+$: 959.27505 [*M*+H]$^+$; found: 959.27481.

Methyl 2,4-di-*O*-acetyl-6-*S*-acetyl-3-*O*-[4'-(2,3,4,6-tetra-*O*-acetyl-α-D-mannopyranosyloxy) azobenzene]-6-thio-α-D-mannopyranoside (S9): The toslyated mannoside **S8** (656 mg, 684 µmol, 1 equiv) was dissolved in acetonitrile (50 mL) and potassium thioacetate (391 mg, 3.42 mmol, 5 equiv) was added. The reaction mixture was stirred for 5 h at 85 °C and further 18 h at room temperature. Then, it was diluted with ethyl acetate (100 mL) and the organic phase was washed with brine (3 × 50 mL), dried over $MgSO_4$ and the solvent was removed under reduced pressure. Purification by column chromatography (cyclohexane/ethyl acetate 1:1) delivered the product as an orange foam (567 mg, 657 µmol, 96%). R_f=0.62 (cyclohexane/ethyl acetate 1:1); $[\alpha]_D^{20}$=+86.4 (*c*=1.0 in dichloromethane); ^{1}H NMR (500 MHz, $CDCl_3$): δ = 7.90 – 7.83 (m, 4H, H_b, $H_{b'}$), 7.24 – 7.19 (m, 2H, $H_{c'}$), 7.05 – 7.00 (m, 2H, H_c), 5.61 (d, $^3J_{1',2'}$ = 1.8 Hz, 1H, H-1'), 5.58 (dd, $^3J_{3',4'}$ = 10.0 Hz, $^3J_{2',3'}$ = 3.6 Hz, 1H, H-3'), 5.48 (dd, $^3J_{2',3'}$ = 3.6 Hz, $^3J_{1',2'}$ = 1.9 Hz, 1H, H-2'), 5.39 (dd~t, $^3J_{3,4}$ = $^3J_{4,5}$ = 9.8 Hz, 1H, H-4), 5.38 (dd~t, $^3J_{3',4'}$ = $^3J_{4',5'}$ = 10.1 Hz, 1H, H-4'), 5.35 (dd, $^3J_{2,3}$ = 3.4 Hz, $^3J_{1,2}$ = 1.8 Hz, 1H, H-2), 4.78 (dd, $^3J_{3,4}$ = 9.6 Hz, $^3J_{2,3}$ = 3.4 Hz, 1H, H-3), 4.73 (d, $^3J_{1,2}$ = 1.8 Hz, 1H, H-1), 4.30 (dd, $^2J_{6'a,6'b}$ = 12.3 Hz, $^3J_{5',6'a}$ = 5.6 Hz, 1H, H-6'a), 4.15 – 4.06 (m, 2H, H-5', H-6'b), 3.86 (ddd, $^2J_{4,5}$ = 10.3 Hz, $^3J_{5,6a}$ = 8.4 Hz, $^3J_{5,6b}$ = 2.7 Hz, 1H, H-5), 3.42 (s, 3H, OCH_3), 3.33 (dd, $^2J_{6a,6b}$ = 14.1 Hz, $^3J_{5,6b}$ = 2.7 Hz, 1H, H-6b), 3.01 (dd, $^2J_{6a,6b}$ = 14.1 Hz, $^3J_{5,6b}$ = 8.4 Hz, 1H, H-6a), 2.37 (s, 3H, $SCOCH_3$), 2.22, 2.18, 2.06, 2.05, 2.03, 2.00 (each s, each 3H, 18H, 6 $COCH_3$) ppm; ^{13}C NMR (126 MHz, $CDCl_3$): δ = 194.89 (S*C*OCH_3), 170.51, 170.08, 170.01, 169.96, 169.93, 169.72 (6 *C*OCH_3), 159.92 (C_d), 157.28 ($C_{d'}$), 148.41 ($C_{a'}$), 147.68 (C_a), 124.49 (C_b), 124.32 ($C_{b'}$), 116.71 ($C_{c'}$), 116.53 (C_c), 98.44 (C-1), 95.68 (C-1'), 74.38 (C-3), 70.07 (C-5), 70.00 (C-4), 69.39 (C-5'), 69.34 (C-2), 69.27 (C-2'), 68.79 (C-3'), 65.87 (C-4'), 62.07 (C-6'), 55.26 (OCH_3), 30.52 (C-6), 30.47 (SCO*C*H_3), 20.94, 20.86, 20.81, 20.68 (6 CO*C*H_3) ppm; IR (ATR): ν_{max}/cm^{-1} = 3478 (w), 2927 (w), 1746 (vs), 1694 (m), 1597 (m), 1583 (m), 1496 (m), 1368 (m), 1212 (vs), 1129 (m), 1084 (m), 1053 (m), 1031 (s), 846 (w); HRMS (ESI): m/z: calcd. for $C_{39}H_{46}N_2O_{18}S+H^+$: 863.25392 [*M*+H]$^+$; found: 863.25351.

Methyl 2,4-di-*O*-acetyl-3-*O*-[4'-(2,3,4,6-tetra-*O*-acetyl-α-D-mannopyranosyloxy)azobenzene]-6-thio-α-D-mannopyranoside (11): The thioacetate **S9** (557 mg, 645 µmol, 1 equiv) and 1,4-dithio-D-threitol (338 mg, 2.19 mmol, 3.4 equiv) was dissolved in dry *N*,*N*-dimethylacetamide (6 mL) and trimethylamine (0.18 mL, 129 µmol, 0.20 equiv) was added. The reaction mixture was stirred for 28 h at room temperature, was diluted with toluene (50 mL) and the org. phase was washed with 1 M HCl (3 x 20 mL) and brine (2 × 20 mL). Then, the organic phase was dried over $MgSO_4$, it was filtered and the solvent was removed under reduced pressure. Column chromatography purification (cyclohexane/ethyl acetate 2:1 to 1:2) gave the product as an orange foam (530 mg, 645 µmol, quant.). R_f=0.29 (cyclohexane/ethyl acetate 1:1); $[\alpha]_D^{20}$=+79.9 (*c*=1.0 in dichloromethane); ^{1}H NMR (500 MHz, $CDCl_3$): δ = 7.90 – 7.82 (m, 4H, H_b, $H_{b'}$), 7.24 – 7.18 (m, 2H, $H_{c'}$), 7.08 – 6.99 (m, 2H, H_c), 5.61 (d, $^3J_{1',2'}$ = 1.8 Hz, 1H, H-1'), 5.58 (dd, $^3J_{3,4}$ = 10.0 Hz, $^3J_{2',3'}$ = 3.6 Hz, 1H, H-3'), 5.48 (dd, $^3J_{2',3'}$ = 3.5, $^3J_{1',2'}$ = 1.8 Hz, 1H, H-2'), 5.43 – 5.34 (m, 3H, H-2, H-4, H-4'), 4.81 (dd, $^3J_{3,4}$ = 9.7 Hz, $^3J_{2,3}$ = 3.4 Hz, 1H, H-3), 4.79 (d, $^3J_{1,2}$ = 1.6 Hz, 1H, H-1), 4.30 (dd, $^2J_{6'a,6'b}$ = 12.4 Hz, $^3J_{5',6a'}$ = 5.6 Hz, 1H, H-6'a), 4.15 – 4.06 (m, 2H, H-5', H-6'b), 3.85 (ddd, $^3J_{4,5}$ = 10.4 Hz, $^3J_{5,6a}$ = 8.3 Hz, $^3J_{5,6b}$ = 2.7 Hz, 1H, H-5), 3.49 (s, 3H, OCH_3), 2.77 (ddd, $^2J_{6a,6b}$ = 14.7 Hz, $^3J_{5,6a}$ = 8.5, $^3J_{6a,SH}$ = 6.4 Hz, 1H, H-6a), 2.64 (ddd, $^2J_{6a,6b}$ = 14.0 Hz, $^3J_{6b,SH}$ = 10.3, $^3J_{5,6b}$ = 2.6 Hz, 1H, H-6b), 2.22, 2.18, 2.06, 2.05, 2.03, 1.94 (each s, each 3H, 18H, 6 $COCH_3$), 1.84 (dd, $^3J_{6b,SH}$ = 10.3 Hz, $^3J_{6a,SH}$ = 6.4 Hz, 1H, SH) ppm; ^{13}C NMR (126 MHz, $CDCl_3$): δ = 170.51, 170.08, 169.96, 169.93, 169.89, 169.72 (6 *C*OCH_3), 159.92 (C_d), 157.29 ($C_{d'}$), 148.40 ($C_{a'}$), 147.68 (C_a), 124.51 (C_b), 124.32 ($C_{b'}$), 116.71 ($C_{c'}$), 116.51 (C_c), 98.45 (C-1), 95.68 (C-1'), 74.46 (C-3), 71.40 (C-5), 70.20 (C-4), 69.39 (C-5'), 69.33 (C-2), 69.27 (C-2'), 68.79 (C-3'), 65.86 (C-4'), 62.06 (C-6'), 55.38 (OCH_3), 26.23 (C-6), 20.93, 20.86, 20.78, 20.69, 20.67 (6 CO*C*H_3) ppm; IR (ATR): ν_{max}/cm^{-1} = 3481 (w), 2927 (w), 1746

(vs), 1597 (m), 1584 (m), 1496 (m), 1369 (m), 1215 (vs), 1132 (m), 1084 (m), 1035 (s), 846 (w); HRMS (ESI): m/z: calcd. for $C_{37}H_{44}N_2O_{17}S+H^+$: 821.24335 [*M*+H]$^+$; found: 821.24282.

Methyl 2,4-di-*O*-acetyl-3-*O*-[4’-(2,3,4,6-tetra-*O*-acetyl-α-D-mannopyranosyloxy)azobenzene]-6-*S*-[4’-*S*-(2,3,4,6-tetra-*O*-acetyl-α-D-mannopyranosyl)-2,6,2’,6’-tetrafluoro-4’-thio-azobenzene]-α-D-mannopyranoside (12): The thiol **11** (80.7 mg, 91.4 µmol, 1.00 equiv) and aryl iodide **10** (67.9 mg, 91.4 µmol, 1.00 equiv) were treated according to the general procedure of the Buchwald–Hartwig–Migita cross-coupling at room temperature over 2 h in degassed dry THF (2 mL). Column chromatography (cyclohexane/ethyl acetate 5:1 to 1:1) yielded the product as a red amorphous solid (82.6 mg, 57.5 µmol, 59%). R_f=0.29 (cyclohexane/ethyl acetate 1:1); $[\alpha]_D^{20}$=+106.2 (*c*=0.7 in dichloromethane); ^{1}H NMR (500 MHz, DMSO-d_6): δ = 7.91 – 7.83 (m, 4H, $H_{b'}$, H_b), 7.59 – 7.52 (m, 2H, $H_{g'}$), 7.43 – 7.37 (m, 2H, H_g), 7.37 – 7.32 (m, 2H, $H_{c'}$), 7.16 – 7.11 (m, 2H, H_c), 6.18 (d, $^3J_{1'',2''}$ = 1.3 Hz, 1H, H-1”), 5.91 (d, $^3J_{1',2'}$ = 1.5 Hz, 1H, H-1’), 5.41 (dd, $^3J_{2'',3''}$ = 3.3 Hz, $^3J_{1'',2''}$ = 1.6 Hz, 1H, H-2”), 5.42 – 5.38 (m, 1H, H-2’), 5.42 – 5.35 (m, 1H, H-3’), 5.31 (dd~t, $^3J_{3,4}$ = $^3J_{4,5}$ = 10.3 Hz, 1H, H-4), 5.30 – 5.29 (m, 1H, H-2), 5.22 (dd~t, $^3J_{3',4'}$ = $^3J_{4',5'}$ = 9.8 Hz, 1H, H-4’), 5.19 (dd~t, $^3J_{3'',4''}$ = $^3J_{4'',5''}$ = 10.1 Hz, 1H, H-4”), 5.14 (dd, $^3J_{3'',4''}$ = 10.1 Hz, $^3J_{2'',3''}$ = 3.4 Hz, 1H, H-3”), 4.88 (dd, $^3J_{3,4}$ = 9.5 Hz, $^3J_{2,3}$ = 3.4 Hz, 1H, H-3), 4.84 (d, $^3J_{1,2}$ = 1.6 Hz, 1H, H-1), 4.37 (ddd, $^3J_{4'',5''}$ = 9.2 Hz, $^3J_{5'',6''a}$ = 6.2 Hz, $^3J_{5'',6''b}$ = 2.4 Hz, 1H, H-5”), 4.21 (dd, $^2J_{6''a,6''b}$ = 12.6 Hz, $^3J_{5'',6''a}$ = 6.2 Hz, 1H, H-6”a), 4.19 (dd, $^2J_{6'a,6'b}$ = 12.8 Hz, $^3J_{5',6'a}$ = 5.9 Hz, 1H, H-6’a), 4.11 – 3.97 (m, 4H, H-5, H-5’, H-6’b, H-6”b), 3.61 (dd, $^2J_{6a,6b}$ = 13.6 Hz, $^3J_{5,6a}$ = 2.4 Hz, 1H, H-6a), 3.33 – 3.28 (m, 1H, H-6b), 3.36 (s, 3H, OCH_3), 2.18, 2.16, 2.15, 2.07, 2.06, 2.00, 2.00, 1.98, 1.94, 1.92 (each s, each 3H, 30H, 10 $COCH_3$) ppm; ^{13}C NMR (126 MHz, DMSO-d_6): δ = 169.76, 169.71, 169.60, 169.56, 169.45, 169.41, 169.34, 169.30 (10 *C*OCH_3), 159.48 (C_d), 156.91 ($C_{d'}$), 154.88 (dd, $^1J_{C,F}$ = 262.1 Hz, $^3J_{C,F}$ = 5.3 Hz, 2 C_f), 154.39 (dd, $^1J_{C,F}$ = 261.2 Hz, $^3J_{C,F}$ = 5.2 Hz, 2 $C_{f'}$), 147.47 ($C_{a'}$), 146.83 (C_a), 145.15 (t, $^3J_{C,F}$ = 11.0 Hz, 2 C_h), 138.45 (t, $^3J_{C,F}$ = 11.1 Hz, 2 $C_{h'}$), 129.28 (t, $^2J_{C,F}$ = 10.0 Hz, 2 $C_{e'}$), 127.57 (t, $^2J_{C,F}$ = 9.7 Hz, 2 C_e), 124.23 (C_b), 123.93 ($C_{b'}$), 117.32 ($C_{c'}$), 116.74 (C_c), 113.87 (d, $^2J_{C,F}$ = 22.9 Hz, 2 $C_{g'}$), 110.66 (d, $^2J_{C,F}$ = 23.1 Hz, 2 C_g), 97.70 (C-1), 94.91 (C-1’), 82.43 (C-1”), 74.18 (C-3), 69.57 (C-5”), 69.26 (C-4), 69.09 (C-2”), 68.78(C-5’), 68.71 (C-3”), 68.57 (C-5), 68.36 (C-3’), 68.31 (C-2), 68.11 (C-2’), 65.23 (C-4”), 65.02 (C-4’), 61.68 (C-6”), 61.52 (C-6’), 54.53 (OCH_3), 32.63 (C-6), 20.58, 20.51, 20.47, 20.32, 20.27, 20.25, 20.11 (10 CO*C*H_3) ppm; ^{19}F NMR (471 MHz, DMSO-d_6): δ = -119.50 ($F_{f/f'}$), -120.24($F_{f/f'}$) ppm; IR (ATR): ν_{max}/cm^{-1} = 2962 (w), 1748 (vs), 1609 (m), 1496 (w), 1369 (m), 1218 (vs), 1131 (m), 1042 (s), 900 (w), 846 (w); HRMS (ESI): *m/z*: calcd. for $C_{63}H_{66}F_4N_4O_{26}S_2+H^+$: 1435.34157 [*M*+H]$^+$; found: 1435.34065.

Methyl 3-*O*-[4’-(α-D-mannopyranosyloxy)azobenzene]-6-*S*-[4’-*S*-(α-D-mannopyranosyl)-2,6,2’,6’-tetrafluoro-4’-thio-azobenzene]-α-D-mannopyranoside (2): The acetylated cluster **12** (20.2 mg, 14.1 µmol, 1.00 equiv) was treated according to the general procedure for deacylation and after purification by column chromatography (Sephadex LH-20, methanol) the product was obtained as a red amorphous solid (13.7 mg, 13.5 µmol, 96%). $[\alpha]_D^{20}$=+155.4 (*c*=0.3 in methanol); ^{1}H NMR (500 MHz, methanol-d_4): δ = 7.89 – 7.79 (m, 4H, H_b, $H_{b'}$), 7.36 – 7.29 (m, 2H, $H_{g'}$), 7.28 – 7.22 (m, 2H, $H_{c'}$), 7.22 – 7.14 (m, 4H, H_g, H_c), 5.73 (d, $^3J_{1'',2''}$ = 1.6 Hz, 1H, H-1”), 5.59 (d, $^3J_{1',2'}$ = 1.9 Hz, 1H, H-1’), 4.71 (d, $^3J_{1,2}$ = 1.8 Hz, 1H, H-1), 4.56 (dd, $^3J_{3,4}$ = 9.2 Hz, $^3J_{2,3}$ = 3.1 Hz, 1H, H-3), 4.14 (dd, $^3J_{2,3}$ = 3.2 Hz, $^3J_{1,2}$ = 1.8 Hz, 1H, H-2), 4.08 (dd, $^3J_{2'',3''}$ = 3.3 Hz, $^3J_{1'',2''}$ = 1.6 Hz, 1H, H-2”), 4.04 (dd, $^3J_{2',3'}$ = 3.4 Hz, $^3J_{1',2'}$ = 1.9 Hz, 1H, H-2’), 4.01 (t, $^3J_{3,4}$ = $^3J_{4,5}$ = 9.4 Hz, 1H, H-4), 3.98 – 3.91 (m, 1H, H-5”), 3.93 (dd, $^3J_{3',4'}$ = 9.5 Hz, $^3J_{2',3'}$ = 3.4 Hz, 1H, H-3’), 3.86 (dd, $^2J_{6'a,6'b}$ = 12.0 Hz, $^3J_{5',6'a}$ = 2.4 Hz, 1H, H-6’a), 3.88 – 3.82 (m, 1H, H-5), 3.81 – 3.63 (m, 7H, H-6’b, H-6”a, H-4”, H-4’, H-6”b, H-6a, H-3”), 3.60 (ddd, $^3J_{4',5'}$ = 9.9 Hz, $^3J_{5',6'a}$ = 5.3 Hz, $^3J_{5',6'b}$ = 2.6 Hz, 1H, H-5’), 3.42 (s, 3H, OCH_3), 3.28 (dd, $^2J_{6a,6b}$ = 14.0 Hz, $^3J_{5,6b}$ = 8.9 Hz, 1H, H-6b) ppm; ^{13}C NMR (126 MHz, methanol-d_4): δ = 161.99 (C_d), 159.95 ($C_{d'}$), 157.20 (dd, $^1J_{C,F}$ = 262.5 Hz, $^3J_{C,F}$ = 5.1 Hz, 2 C_f), 156.75 (dd, $^1J_{C,F}$ = 262.1 Hz, $^3J_{C,F}$ = 5.0 Hz, 2 $C_{f'}$), 149.30 ($C_{a'}$), 148.65 (C_a), 147.11 (t, $^3J_{C,F}$ = 10.8 Hz, 2 C_h), 142.77 (t, $^3J_{C,F}$ = 10.5 Hz, 2 $C_{h'}$), 131.09 (t, $^2J_{C,F}$ = 9.6 Hz, 2 $C_{a'}$), 129.67 (t, $^2J_{C,F}$ = 9.6 Hz, 2 C_a), 125.43 (C_b), 125.25 ($C_{b'}$), 118.02 ($C_{c'}$), 117.65 (C_c), 114.12 (dd, $^2J_{C,F}$ = 24.2 Hz, $^4J_{C,F}$ = 2.9 Hz, 2 $C_{g'}$), 111.31 (dd, $^2J_{C,F}$ = 23.7 Hz, $^4J_{C,F}$ = 2.6 Hz, 2 C_g), 102.81 (C-1), 100.13 (C-1’), 88.89 (C-1”), 79.93 (C-3), 76.42 (C-5”), 75.68 (C-5’), 73.73 (C-5), 73.40 (C-2”), 73.27 (C-3”), 72.42 (C-3’), 71.92 (C-2’), 69.90 (C-4), 68.88 (C-2), 68.63 (C-4’), 68.33 (C-4”), 62.70 (C-6”), 62.60 (C-6’), 55.45 (OCH_3), 35.23 (C-6) ppm; ^{19}F NMR (471 MHz, methanol-d_4): δ = -119.96 ($F_{f/f'}$), -120.52 ($F_{f/f'}$) ppm; IR (ATR):

ν_{max}/cm^{-1} = 3366 (br), 2926 (w), 1607 (vs), 1556 (m), 1496 (s), 1420 (s), 1233 (vs), 1128 (m), 1102 (m), 1063 (vs), 1043 (vs), 1009 (vs), 905 (w), 841 (s); HRMS (ESI): m/z: calcd. for $C_{43}H_{46}F_4N_4O_{16}S_2$+H$^+$: 1015.23592 [*M*+H]$^+$; found: 1015.23666.

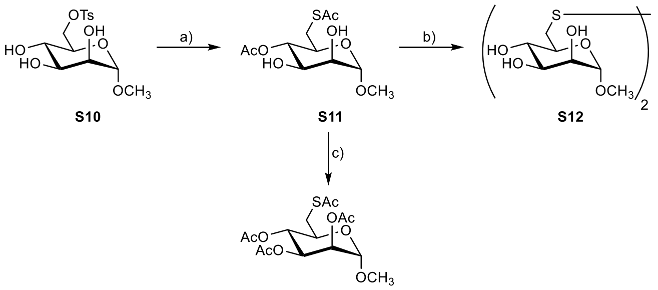

Scheme S2: Synthesis of azobenzene 6-thiomannoside **13**. Reagents and conditions: a) KSAc, MeCN, 85 °C, 4.5 h, 40%; b) NaOMe, MeOH/CH_2Cl_2 2:1, rt, 24 h 84%; c) Ac_2O, DMAP, pyridine, 3 h, rt, 97%.

Methyl 4-*O*-acetyl-6-*S*-acetyl-6-thio-α-D-mannopyranoside (S11) [4]**:** The tosyl-activated mannoside **S10** [5] (734 mg, 2.11 mmol, 1.00 equiv) and potassium thioacetate (1.20 g, 10.5 mmol, 5.00 equiv) were dissolved in acetonitrile (50 mL) and stirred for 4.5 h at 85 °C. The heating source was removed and the mixture was diluted with ethyl acetate (100 mL) and dist. water (100 mL). The phases were separated and the aq. phase was extracted with ethyl acetate (3 × 50 mL). The combined organic phases were washed with dist. water (1 × 50 mL) and brine (1 × 50 mL), dried over $MgSO_4$, filtered and the solvent was removed under reduced pressure. The raw product was purified by column chromatography (cyclohexane/ethyl acetate 3:7) and a colorless amorphous solid was obtained (247 mg, 839 µmol, 40%). R_f=0.29 (cyclohexane/ethyl acetate 3:7); $[\alpha]_D^{20}$=+66.5 (*c*=0.6 in dichloromethane); ^{1}H NMR (500 MHz, Methanol-d$_4$): δ = 5.03 (dd~t, $^3J_{3,4}$ = $^3J_{4,5}$ = 9.7 Hz, 1H, H-4), 4.60 (d, $^3J_{1,2}$ = 1.7 Hz, 1H, H-1), 3.80 (dd, $^3J_{2,3}$ = 3.4 Hz, $^3J_{1,2}$ = 1.7 Hz, 1H, H-2), 3.76 (dd, $^3J_{3,4}$ = 9.6 Hz, $^3J_{2,3}$ = 3.4 Hz, 1H, H-3), 3.66 – 3.60 (m, 1H, H-5), 3.36 (s, 3H, OCH_3), 3.23 (dd, $^2J_{6,6'}$ = 14.0 Hz, $^3J_{5,6}$ = 2.7 Hz, 1H, H-6), 2.93 (dd, $^2J_{6,6'}$ = 14.0 Hz, $^3J_{5,6'}$ = 8.3 Hz, 1H, H-6'), 2.31 (s, 3H, $SCOCH_3$), 2.12 (s, 3H, $COCH_3$) ppm; ^{13}C NMR (126 MHz, Methanol-d$_4$): δ = 196.72 (S*C*OCH$_3$), 172.60 (*C*OCH$_3$), 102.76 (C-1), 73.14 (C-4), 72.05 (C-2), 71.36 (C-5), 70.42 (C-3), 55.40 (OCH_3), 31.56 (C-6), 30.36 (SCO*C*H$_3$), 21.10 (CO*C*H$_3$) ppm; IR (ATR): ν_{max}/cm^{-1} = 3267 (br), 2927 (w), 1737 (s), 1688 (s), 1375 (m), 1235 (vs), 1103 (s), 1043 (vs); HRMS (ESI): m/z: calcd. for $C_{11}H_{18}O_7S$+NH$^+$: 312.11115 [*M*+NH]$^+$; found: 312.11089.

***S*,*S*'-Di-(methyl α-D-mannopyranoside-6-yl)-disulfide (S12)** [6]**:** The 6-*S*-acetylated mannoside **S11** (40.8 mg, 139 µmol, 1.00 equiv) was treated according to the general procedure of deacylation with a reaction time of 24 h at room temperature. After purification by reversed-phase column chromatography (MeCN/H_2O 5:95 to 95:5, 30 cv) the product was obtained as a colorless syrup (24.6 mg, 58.8 µmol, 84%). $[\alpha]_D^{20}$=+165.6 (*c*=0.2 in methanol); ^{1}H NMR (600 MHz, DMSO-d$_6$): δ = 4.95 (d, $^3J_{4,OH}$ = 5.7 Hz, 2H, 2 OH-4), 4.80 (d, $^3J_{2,OH}$ = 4.4 Hz, 2H, 2 OH-2), 4.64 (d, $^3J_{3,OH}$ = 6.1 Hz, 2H, 2 OH-3), 4.49 (d, $^3J_{1,2}$ = 1.6 Hz, 2H, 2 H-1), 3.60 (ddd, $^3J_{2,OH}$ = 4.7 Hz, $^3J_{2,3}$ = 3.4 Hz, $^3J_{1,2}$ = 1.6 Hz, 2H, 2 H-2), 3.49 (ddd~td, $^3J_{4,5}$ = $^3J_{5,6'}$ = 9.6 Hz, $^3J_{5,6}$ = 2.1 Hz, 2H, 2 H-5), 3.42 (ddd, $^3J_{3,4}$ = 9.4 Hz, $^3J_{3,OH}$ = 6.0 Hz, $^3J_{2,3}$ = 3.4 Hz, 2H, 2 H-3), 3.30 (ddd~td, $^3J_{3,4}$ = $^3J_{4,5}$ = 9.3 Hz, $^3J_{4,OH}$ = 5.7 Hz, 2H, 2 H-4), 3.26 (s, 6H, OCH_3), 3.21 (dd, $^2J_{6,6'}$ = 13.4 Hz, $^3J_{5,6}$ = 2.1 Hz, 2H, 2 H-6), 2.77 (dd, $^2J_{6,6'}$ = 13.4 Hz, $^3J_{5,6'}$ = 9.7 Hz, 2H, 2 H-6') ppm; ^{13}C NMR (151 MHz, DMSO-d$_6$): δ = 101.02 (2 C-1), 71.09 (2 C-5), 70.79 (2 C-3), 70.22 (2 C-2), 69.75 (2 C-4), 54.10 (2 OCH_3), 41.44 (2 C-6) ppm; IR (ATR): ν_{max}/cm^{-1} = 3357 (br), 2923 (m), 1657 (m), 1632 (m), 1134 (m), 1054 (m); HRMS (ESI): m/z: calcd. for $C_{14}H_{26}O_{10}S_2$+NH$_4^+$: 436.13057 [2*M*-2H+NH$_4$]$^+$; found: 436.13051.

Methyl 2,3,4-tri-*O*-acetyl-6-*S*-acetyl-6-thio-α-D-mannopyranoside (13) [7]**:** The partially acetylated thiol **S11** (197 mg, 670 µmol, 1.00 equiv) was dissolved in a mixture of pyridine and acetic anhydride 2:1 (3 mL) and DMAP (8.19 mg, 67.0 µmol, 0.10 equiv) was added to the mixture, which was stirred for 3 h at room temperature. Then, it was diluted with ethyl acetate (50 mL) and the organic phase was

washed with 1 M HCL (3 × 50 mL) and brine (3 × 50 mL), dried over $MgSO_4$, filtered and the solvent was removed under reduced pressure. Purification by column chromatography (cyclohexane/ethyl acetate 3:1 to 1:1) gave the product as a colorless syrup (247 mg, 652 µmol, 97%). R_f=0.84 (cyclohexane/ethyl acetate 3:7); $[\alpha]_D^{20}$=+43.8 (*c*=1.0 in dichloromethane); ^{1}H NMR (600 MHz, $CDCl_3$): δ = 5.28 (dd, $^3J_{3,4}$ = 10.0 Hz, $^3J_{2,3}$ = 3.5 Hz, 1H, H-3), 5.21 (dd, $^3J_{2,3}$ = 3.5 Hz, $^3J_{1,2}$ = 1.8 Hz, 1H, H-2), 5.18 (dd~t, $^3J_{3,4}$ = $^3J_{4,5}$ = 9.9 Hz, 1H, H-4), 4.64 (d, $^3J_{1,2}$ = 1.8 Hz, 1H, H-1), 3.87 – 3.81 (m, 1H, H-5), 3.38 (s, 3H, OCH_3), 3.27 (dd, $^2J_{6,6'}$ = 14.1 Hz, $^3J_{5,6}$ = 2.8 Hz, 1H, H-6), 3.01 (dd, $^2J_{6,6'}$ = 14.1 Hz, $^3J_{5,6'}$ = 8.1 Hz, 1H, H-6'), 2.35 (s, 3 H, $SCOCH_3$), 2.14, 2.10, 1.98 (each s, each 3H, 9H, 3 $COCH_3$) ppm; ^{13}C NMR (151 MHz, $CDCl_3$): δ = 194.72 (S*C*OCH$_3$), 170.10, 170.06, 169.87 (3 *C*OCH$_3$), 98.43 (C-1), 69.74 (C-5), 69.58 (C-2), 68.96 (C-3), 68.72 (C-4), 55.21 (OCH_3), 30.43 (SCO*C*H$_3$), 20.90, 20.81, 20.69 (3 CO*C*H$_3$) ppm; IR (ATR): ν_{max}/cm^{-1} = 2937 (w), 1746 (vs), 1693 (s), 1368 (s), 1242 (s), 1215 (vs), 1131 (s), 1076 (s), 1054 (s), 1039 (s); HRMS (ESI): m/z: calcd. for $C_{15}H_{22}O_9S+Na^+$: 401.08768 $[M+Na]^+$; found: 401.08707.

Methyl 2,3,4-tri-*O*-acetyl-6-*S*-[4'-*S*-(2,3,4,6-tetra-*O*-acetyl-α-D-mannopyranosyl)-2,6,2',6'-tetra-fluoro-4'-thio-azobenzene]-α-D-mannopyranoside (16): The thioacetate **13** (85.0 mg, 225 µmol, 1.00 equiv) and 1,4-dithio-D-threitol (52.0 mg, 337 µmol, 1.50 equiv) were dissolved in dry *N*,*N*-dimethylacetamide (1.5 mL) and trimethylamine (3.0 µL, 22.5 µmol, 0.10 equiv) was added. The reaction mixture was stirred for 1.1 d and poured into dist. water (20 mL). The aq. phase was extracted with toluene (3 × 20 mL) and the combined organic phases were washed with water (3 × 20 mL) and brine (3 × 20 mL). The solvent was removed under reduced pressure and the obtained raw product was purified by column chromatography (cyclohexane/ethyl acetate 3:1). The obtained yellow syrup (SAc/SH: 28:72, verified by ^{1}H NMR) was directly used (53%) according to the general procedure for the Buchwald–Hartwig–Migita cross-coupling together with the aryl iodide **15** [8] (78.8 mg, 106 µmol, 0.90 equiv, related to the SAc:SH ratio and batch size). In THF (3 mL) at 0 °C triethyl amine was added and the reaction was stirred for 18 h at room temperature. After the work-up, purification by column chromatography (cyclohexane/ethyl acetate 1:0 to 1:1) yielded the product as a red amorphous solid (71.7 mg, 75.4 µmol, 63%). R_f=0.38 (cyclohexane/ethyl acetate 1:1); $[\alpha]_D^{20}$=+67.5 (*c*=0.7 in dichloromethane); ^{1}H NMR (600 MHz, DMSO-d$_6$): δ = 7.42 – 7.39 (m, 2H, $H_{c'}$), 7.39 – 7.35 (m, 2H, H_c), 5.67 (d, $^3J_{1',2'}$ = 10.1 Hz, 1H, H-1'), 5.40 (dd~t, $^3J_{2',3'}$ = $^3J_{3',4'}$ = 9.4 Hz, 1H, H-3'), 5.14 – 5.05 (m, 3H, H-2, H-3, H-4), 5.00 (dd~t, $^3J_{3',4'}$ = $^3J_{4',5'}$ = 9.8 Hz, 1H, H-4'), 4.99 (dd~t, $^3J_{1',2'}$ = $^3J_{2',3'}$ = 9.7 Hz, 1H, H-2'), 4.77 (d, $^3J_{1,2}$ = 1.5 Hz, 1H, H-1), 4.28 (ddd, $^3J_{4',5'}$ = 10.1 Hz, $^3J_{5',6'a}$ = 5.7 Hz, $^3J_{5',6'b}$ = 3.1 Hz, 1H, H-5'), 4.16 – 4.09 (m, 2H, H-6'a, H-6'b), 3.92 (ddd~td, $^3J_{4,5}$ = $^3J_{5,6a}$ = 8.9 Hz, $^3J_{5,6b}$ = 2.6 Hz, 1H, H-5), 3.55 (dd, $^2J_{6a,6b}$ = 14.1 Hz, $^3J_{5,6b}$ = 2.6 Hz, 1H, H-6b), 3.32 (s, 3H, CH_3), 3.28 (dd, $^2J_{6a,6b}$ = 14.1 Hz, $^3J_{5,6a}$ = 8.4 Hz, 1H, H-6a), 2.11, 2.09, 2.05, 2.02, 2.02, 1.97, 1.94 (each s, each 3H, 21H, 7 $COCH_3$)ppm; ^{13}C NMR (151 MHz, DMSO-d$_6$): δ = 169.95, 169.64, 169.61, 169.53, 169.35, 169.20 (7 *C*OCH$_3$), 154.97 (dd, $^1J_{C,F}$ = 260.9 Hz, $^3J_{C,F}$ = 5.3 Hz, 2 C_b), 154.59 (dd, $^1J_{C,F}$ = 260.8 Hz, $^3J_{C,F}$ = 5.0 Hz, 2 $C_{b'}$)145.13 (t, $^3J_{C,F}$ = 10.9 Hz, 2 C_d), 139.81 (t, $^3J_{C,F}$ = 10.9 Hz, 2 $C_{d'}$), 129.03 ($C_{a'}$), 127.68 (C_a), 112.65 (d, $^2J_{C,F}$ = 24.1 Hz, 2 $C_{c'}$), 110.72 (d, $^2J_{C,F}$ = 23.4 Hz, 2 C_c), 97.63 (C-1), 81.48 (C-1'), 74.58 (C-5'), 72.70 (C-3'), 69.07 (C-2'), 68.80 (C-3), 68.68 (C-5), 68.65 (C-2), 67.97 (C-4'), 67.85 (C-4), 62.08 (C-6'), 54.62 (OCH_3), 32.73 (C-6), 20.68, 20.59, 20.41, 20.38, 20.35, 20.29, 20.26 (7 CO*C*H$_3$) ppm; ^{19}F NMR (471 MHz, DMSO-d$_6$): δ = -119.54 ($F_{b/b'}$), -119.85 ($F_{b/b'}$) ppm; IR (ATR): ν_{max}/cm^{-1} = 2939 (w), 1747 (vs), 1610 (s), 1558 (w), 1421 (m), 1369 (s), 1216 (vs), 1133 (m), 1079 (m), 1040 (vs); HRMS (ESI): m/z: calcd. for $C_{39}H_{42}F_4N_2O_{17}S_2+NH_4^+$: 968.21993 $[M+NH_4]^+$; found: 968.21843.

Methyl 6-*S*-[4'-*S*-(β-D-glucopyranosyl)-2,6,2',6'-tetrafluoro-4'-thio-azobenzene]-α-D-manno-pyranoside (3): The glycoantenna **16** (32.0 mg, 33.7 µmol, 1.00 equiv) was treated according to the general procedure for deacylation and purification by reversed-phase column chromatography (MeCN/H_2O 5:95 to 95:5, 17 cv) to give the product as a red amorphous solid (17.0 mg, 25.9 µmol, 77%). $[\alpha]_D^{20}$=+48.0 (*c*=0.7 in methanol); ^{1}H NMR (600 MHz, methanol-d$_4$): δ = 7.34 – 7.26 (m, 2H, $H_{c'}$), 7.21 – 7.14 (m, 2H, H_c), 4.88 – 4.86 (m, 1H, H-1'), 4.61 (d, $^3J_{1,2}$ = 1.7 Hz, 1H, H-1), 3.91 (dd, $^2J_{6'a,6'b}$ = 12.2 Hz, $^3J_{5',6'a}$ = 2.3 Hz, 1H, H-6'a), 3.79 (dd, $^3J_{2,3}$ = 3.3 Hz, $^3J_{1,2}$ = 1.7 Hz, 1H, H-2), 3.71 – 3.58 (m, 5H, H-3, H-4, H-5, H-6a, H-6'b), 3.49 – 3.41 (m, 2H, H-5', H-3'), 3.35 (dd~t, $^3J_{3',4'}$ = $^3J_{4',5'}$ = 9.6 Hz, 1H, H-4'), 3.34 (s, 3H, OCH_3), 3.35 – 3.31 (m, 1H, H-2'), 3.18 (dd, $^2J_{6a,6b}$ = 14.0 Hz, $^3J_{5,6b}$ = 9.0 Hz, 1H, H-6b) ppm;

^{13}C NMR (151 MHz, methanol-d$_4$): δ = 157.12 (dd, $^1J_{C,F}$ = 254.1 Hz, $^3J_{C,F}$ = 4.4 Hz, 2 C$_b$), 156.65 (dd, $^1J_{C,F}$ = 262.0 Hz, $^3J_{C,F}$ = 4.6 Hz, 2 C$_{b'}$), 147.15 (t, $^3J_{C,F}$ = 10.5 Hz, 2 C$_d$), 142.83 (t, $^3J_{C,F}$ = 10.4 Hz, 2 C$_{d'}$), 130.90 (C$_{a'}$), 129.59 (C$_a$), 113.94 (d, $^2J_{C,F}$ = 23.8 Hz, 2 C$_{c'}$), 111.18 (d, $^2J_{C,F}$ = 23.7 Hz, 2 C$_c$), 102.82 (C-1), 87.80 (C-1'), 82.28 (C-5'), 79.64 (C-3'), 73.79 (C-2'), 73.42 (C-3' or 4' or 5'), 72.46 (C-3' or 4' or 5'), 72.00 (C-2), 71.63 (C-3' or 4' or 5'), 71.29 (C-4'), 62.73 (C-6'), 55.21 (OCH$_3$), 35.15 (C-6) ppm; ^{19}F NMR (471 MHz, methanol-d$_4$): δ = -119.68 (F$_{b/b'}$), -120.06 (F$_{b/b'}$) ppm; IR (ATR): ν_{max}/cm^{-1} = 3349 (br), 2921 (m), 1609 (s), 1556 (m), 1420 (m), 1131 (m), 1097 (m), 1068 (s), 1042 (vs); HRMS (ESI): m/z: calcd. for $C_{25}H_{28}F_4N_2O_{10}S_2+H^+$: 657.11943 [*M*+H]$^+$; found: 657.11874.

Methyl 2,3,4-tri-*O*-acetyl-6-*S*-[4'-*S*-(2,3,4,6-tetra-*O*-acetyl-α-D-mannopyranosyl)-2,6,2',6'-tetrafluoro-4'-thio-azobenzene]-α-D-mannopyranoside (17): The thioacetate **13** (73.0 mg, 193 µmol, 1.00 equiv) and 1,4-dithio-D-threitol (44.6 mg, 289 µmol, 1.50 equiv) were dissolved in dry *N*,*N*-dimethylacetamide (1.3 mL) and trimethylamine (2.7 µL, 19.3 µmol, 0.10 equiv) was added. The reaction mixture was stirred for 23 h and poured into dist. water (20 mL). The aq. phase was extracted with toluene (3 × 20 mL) and the combined organic phases were washed with water (3 × 20 mL) and brine (3 × 20 mL). The solvent was removed under reduced pressure and the obtained raw product was purified by column chromatography (cyclohexane/ethyl acetate 3:1). The obtained yellow syrup (SAc/SH: 46:54, verified by ^{1}H NMR) was directly used (92%) according to the general procedure for the Buchwald–Hartwig–Migita cross-coupling together with the aryl iodide **10** (99.3 mg, 134 µmol, 2.08 equiv, related to the SAc:SH ratio and batch size). In THF (3 mL) at 0 °C triethyl amine was added and the reaction was stirred for 18 h at room temperature. After the work-up, purification by column chromatography (cyclohexane/ethyl acetate 1:0 to 1:1) yielded the product as a red amorphous solid (49.9 mg, 52.5 µmol, 73%; the detected solvent in the NMR was excluded for the correct yield). R$_f$=0.42 (cyclohexane/ethyl acetate 1:1); $[\alpha]_D^{20}$=+54.8 (*c*=0.7 in dichloromethane); ^{1}H NMR (600 MHz, DMSO-d$_6$): δ = 7.57 – 7.52 (m, 2H, H$_{c'}$), 7.40 – 7.34 (m, 2H, H$_c$), 6.16 (d, $^3J_{1',2'}$ = 1.5 Hz, 1H, H-1'), 5.39 (dd, $^3J_{2',3'}$ = 3.4 Hz, $^3J_{1',2'}$ = 1.5 Hz, 1H, H-2'), 5.20 – 5.04 (m, 4H, H-2, H-3', H-4, H-4'), 4.76 (d, $^3J_{1,2}$ = 1.5 Hz, 1H, H-1), 4.35 (ddd, $^3J_{4',5'}$ = 9.2 Hz, $^3J_{5',6'a}$ = 6.2 Hz, $^3J_{5',6'b}$ = 2.3 Hz, 1H, H-5'), 4.20 (dd, $^2J_{6'a,6'b}$ = 12.3 Hz, $^3J_{5',6'a}$ = 6.3 Hz, 1H, H-6'a), 4.04 (dd, $^2J_{6'a,6'b}$ = 12.3 Hz, $^3J_{5',6'b}$ = 2.4 Hz, 1H, H-6'b), 3.91 (ddd~td, $^3J_{4,5}$ = $^3J_{5,6a}$ = 8.8 Hz, $^3J_{5,6a}$ = 2.6 Hz, 1H, H-5), 3.55 (dd, $^2J_{6a,6b}$ = 14.1 Hz, $^3J_{5,6a}$ = 2.6 Hz, 1H, H-6a), 3.31 (s, 3H, OCH$_3$), 3.27 (dd, $^2J_{6a,6b}$ = 14.2 Hz, $^3J_{5,6b}$ = 8.4 Hz, 1H, H-6a), 2.15, 2.11, 2.08, 2.06, 1.98, 1.94, 1.92 (each s, each 3H, 21H, 7 COCH$_3$) ppm; ^{13}C NMR (151 MHz, DMSO-d$_6$): δ = 169.84, 169.73, 169.66, 169.62, 169.54, 169.47 (7 *C*OCH$_3$), 155.00 (dd, $^1J_{C,F}$ = 260.5 Hz, $^3J_{C,F}$ = 4.9 Hz, 2 C$_b$), 154.50 (dd, $^1J_{C,F}$ = 263.6 Hz, $^3J_{C,F}$ = 4.9 Hz, 2 C$_{b'}$), 145.32 (t, $^3J_{C,F}$ = 10.4 Hz, 2 C$_d$), 138.50 (t, $^3J_{C,F}$ = 11.1 Hz, 2 C$_{d'}$), 129.47 (C$_{a'}$), 127.55 (C$_a$), 114.00 (d, $^2J_{C,F}$ = 22.3 Hz, 2 C$_{c'}$), 110.72 (d, $^2J_{C,F}$ = 23.3 Hz, 2 C$_c$), 97.63 (C-1), 82.54 (C-1'), 69.69 (C-5'), 69.21 (C-2'), 68.82 (C-3'), 68.69 (C-2 or C-5'), 68.66 (C-2 or C-5'), 67.86 (C-4), 65.35 (C-4'), 61.80 (C-6'), 54.63 (OCH$_3$), 32.73 (C-6), 20.77, 20.60, 20.44, 20.42, 20.37, 20.23 (7 CO*C*H$_3$) ppm; ^{19}F NMR (471 MHz, DMSO-d$_6$): δ = -119.53 (F$_{b/b'}$), -120.25 (F$_{b/b'}$) ppm; IR (ATR): ν_{max}/cm^{-1} = 2933 (w), 1745 (vs), 1696 (m), 1602 (s), 1565 (m), 1417 (m), 1368 (s), 1218 (vs), 1131 (m), 1104 (m), 1045 (s); HRMS (ESI): m/z: calcd. for $C_{39}H_{42}F_4N_2O_{17}S_2+NH_4^+$: 968.21993 [M+NH$_4$]$^+$; found: 968.21983.

Methyl 6-*S*-[4'-*S*-(α-D-mannopyranosyl)-2,6,2',6'-tetrafluoro-4'-thio-azobenzene]-α-D-mannopyranoside (4): The acetylated glycoantenna **17** (39.6 mg, 41.6 µmol, 1.00 equiv) was treated according to the general procedure for deacylation and after purification by reversed-phase column chromatography (MeCN/H$_2$O 5:95 to 95:5, 17 cv) the product was obtained as a red amorphous solid (28.5 mg, 43.4 µmol, quant.). $[\alpha]_D^{20}$=+137.9 (*c*=0.8 in methanol); ^{1}H NMR (600 MHz, methanol-d$_4$): δ = 7.36 – 7.30 (m, 2H, H$_{c'}$), 7.19 – 7.14 (m, 2H, H$_c$), 5.73 (d, $^3J_{1,2}$ = 1.7 Hz, 1H, H-1'), 4.62 (d, $^3J_{1,2}$ = 1.7 Hz, 1H, H-1), 4.08 (dd, $^3J_{2',3'}$ = 3.4 Hz, $^3J_{1',2'}$ = 1.6 Hz, 1H, H-2'), 3.94 (ddd, $^3J_{4',5'}$ = 9.1 Hz, $^3J_{5',6'a}$ = 6.1 Hz, $^3J_{5',6'b}$ = 2.5 Hz, 1H, H-5'), 3.86 (dd, $^2J_{6'a,6'b}$ = 12.1 Hz, $^3J_{5',6'a}$ = 2.3 Hz, 1H, H-6'a), 3.80 (dd, $^3J_{2,3}$ = 3.3 Hz, $^3J_{1,2}$ = 1.7 Hz, 1H, H-2), 3.78 (dd, $^2J_{6'a,6'b}$ = 11.3 Hz, $^3J_{5',6'b}$ = 5.2 Hz, 1H, H-6'b), 3.75 (dd~t, $^3J_{3',4'}$ = $^3J_{4',5'}$ = 8.8 Hz, 1H, H-4'), 3.70 – 3.63 (m, 2H, H-3, H-5), 3.66 (dd, $^3J_{3',4'}$ = 9.2 Hz, $^3J_{2',3'}$ = 3.0 Hz, 1H, H-3'), 3.64 – 3.58 (m, 2H, H-4, H-6a), 3.34 (s, 3H, OCH$_3$), 3.18 (dd, $^2J_{6a,6b}$ = 14.0 Hz, $^3J_{5,6b}$ = 8.9 Hz, 1H, H-6b) ppm; ^{13}C NMR (151 MHz, methanol-d$_4$): δ = 157.12 (d, $^1J_{C,F}$ = 262.7 Hz, 2 C$_{b'}$), 156.69 (d, $^1J_{C,F}$ = 262.0 Hz, 2 C$_b$), 147.22 (t, $^3J_{C,F}$ = 10.5 Hz, 2 C$_d$), 142.67 (t, $^3J_{C,F}$ = 10.5 Hz, 2 C$_{d'}$), 131.03 (C$_{a'}$), 129.53 (C$_a$), 114.05 (d, $^2J_{C,F}$ = 23.0 Hz, 2 C$_{c'}$), 111.13 (d, $^2J_{C,F}$ = 23.4 Hz, 2 C$_c$),102.80 (C-1), 88.84 (C-1'), 76.35

(C-5’), 73.38, 73.33, 73.21 (C-2’, C-3’, C-5), 72.45 (C-3 or 4), 71.98 (C-2), 71.62 (C-3 or 4), 68.56 (C-4’), 62.54 (C-6’), 55.21 (OCH_3), 35.13 (C-6) ppm; ^{19}F NMR (471 MHz, methanol-d$_4$): δ = -120.00 ($F_{b/b'}$), -120.53 ($F_{b/b'}$) ppm; IR (ATR): ν_{max}/cm^{-1} = 3342 (br), 2922 (s), 1608 (s), 1556 (m), 1419 (s), 1130 (s), 1096 (s), 1065 (vs), 1040 (vs); HRMS (ESI): m/z: calcd. for $C_{25}H_{28}F_4N_2O_{10}S_2$+H$^+$: 657.11943 [*M*+H]$^+$; found: 657.11877.

Methyl 3-*O*-[4’-(α-D-mannopyranosyloxy)azobenzene]-α-D-mannopyranoside (5): The acyl-protected antenna **18** [9] (32.0 mg, 36.9 µmol, 1.00 equiv) was treated according to the general procedure for deacylation and was stirred for 2 h at room temperature. Revered-phase column chromatography (MeCN/H_2O 5:95 to 95:5, 17 cv) gave the product as an orange amorphous solid (22.7 mg, 41.1 µmol, quant.). $[\alpha]_D^{20}$=+105.7 (*c*=0.4 in dichloromethane); ^{1}H NMR (500 MHz, methanol-d$_4$): δ = 7.88 – 7.82 (m, 4H, H_b, $H_{b'}$), 7.29 – 7.22 (m, 2H, $H_{c'}$), 7.20 – 7.14 (m, 2H, H_c), 5.59 (d, $^3J_{1',2'}$ = 1.9 Hz, 1H, H-1’), 4.73 (d, $^3J_{1,2}$ = 1.9 Hz, 1H, H-1), 4.56 (dd, $^3J_{3,4}$ = 9.3 Hz, $^3J_{2,3}$ = 3.1 Hz, 1H, H-3), 4.12 (dd, $^3J_{2,3}$ = 3.2 Hz, $^3J_{1,2}$ = 1.9 Hz, 1H, H-2), 4.04 (dd, $^3J_{2',3'}$ = 3.5 Hz, $^3J_{1',2'}$ = 1.8 Hz, 1H, H-2), 4.01 (dd~t, $^3J_{3,4}$ = $^3J_{4,5}$ = 9.6 Hz, 1H, H-4), 3.94 – 3.88 (m, 2H, H-3’, H-6a), 3.81 – 3.70 (m, 4H, H-4’, H-6’a, H-6’b, H-6b), 3.68 – 3.64 (m, 1H, H-5), 3.62 – 3.57 (m, 1H, H-5’), 3.45 (s, 3H, OCH_3) ppm; ^{13}C NMR (126 MHz, methanol-d$_4$): δ = 162.11 (C_d), 159.96 ($C_{d'}$), 149.32 ($C_{a'}$), 148.60 (C_a), 125.41 (C_b or $C_{b'}$), 125.23 (C_b or $C_{b'}$), 118.03 ($C_{c'}$), 117.61 (C_c), 102.74 (C-1), 100.15 (C-1’), 80.15 (C-3), 75.70 (C-5’), 74.90 (C-5), 72.42 (C-3’), 71.92 (C-2’), 68.95 (C-2), 68.34 (C-4’), 66.86 (C-4), 62.91 (C-6), 62.71 (C-6’), 55.42 (OCH_3) ppm; IR (ATR): ν_{max}/cm^{-1} = 3347 (br), 2926 (s), 1633 (m), 1596 (s), 1582 (s), 1495 (s), 1232 (vs), 1125 (s), 1101 (s), 1059 (vs), 1009 (vs), 840 (s); HRMS (ESI): m/z: calcd. for $C_{25}H_{32}N_2O_{12}$+H$^+$: 553.20280 [*M*+H]$^+$; found: 553.20270.

2 Photochromic properties

2.1 6αMan3αMan 2

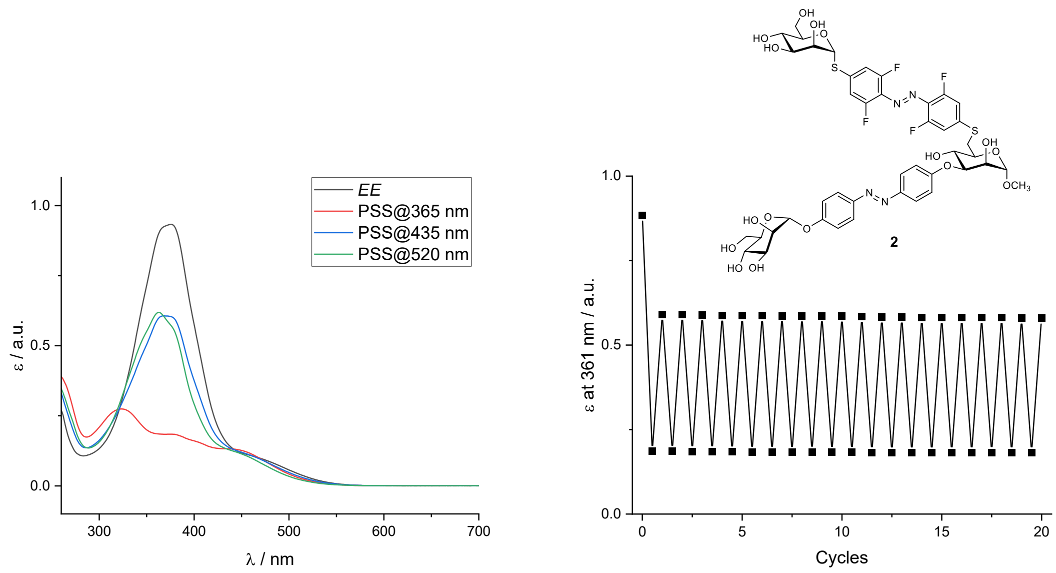

Figure S1: Left: UV–vis spectra of **2** at 25 °C in DMSO (16.5 μM) in *E* (black), and after 2 min of irradiation at 365 nm (red), 435 nm (blue), and 520 nm (green), respectively; right: fatigue resistance of **2** at 25 °C in DMSO (16.5 μM) during several switching cycles; the intensity of the absorption band at 361 nm is shown under alternate 365 nm and 435 nm irradiation of **2**.

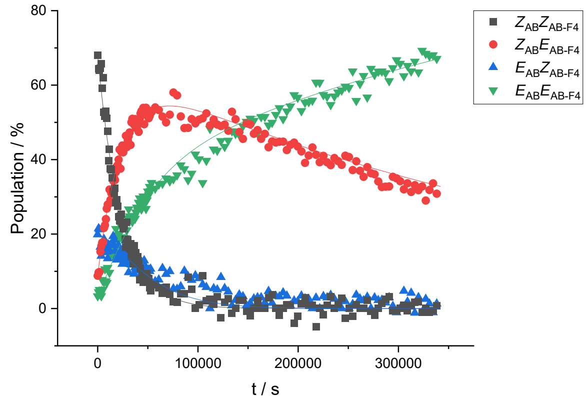

Figure S2: Kinetic traces of the thermal relaxation starting from the PSS@365 of **2** ($Z_{AB}Z_{ABF4}$ as main isomer) to $E_{AB}E_{ABF4}$ via the metastable isomers $E_{AB}Z_{ABF4}$ and $Z_{AB}E_{ABF4}$ at 310 K. The experimental data were fitted with a tailor-made fitting program [9] and the resulting rate constants k_1 to k_4 are collected in Table 2 of the main manuscript.

A

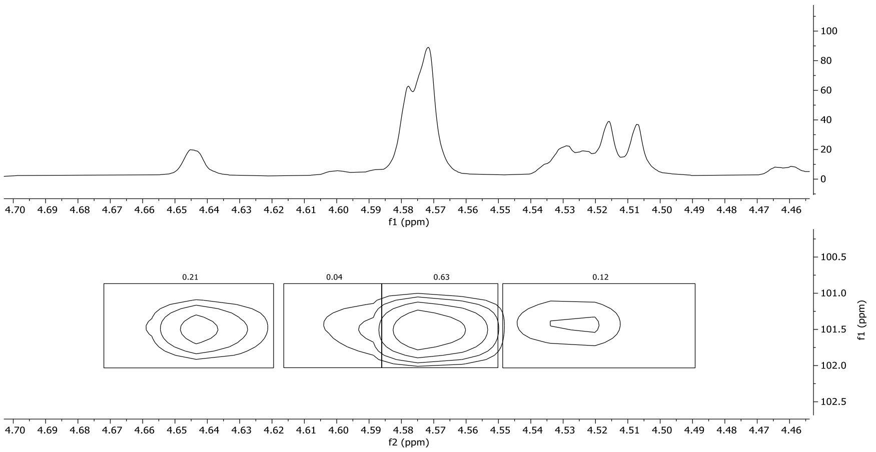

B

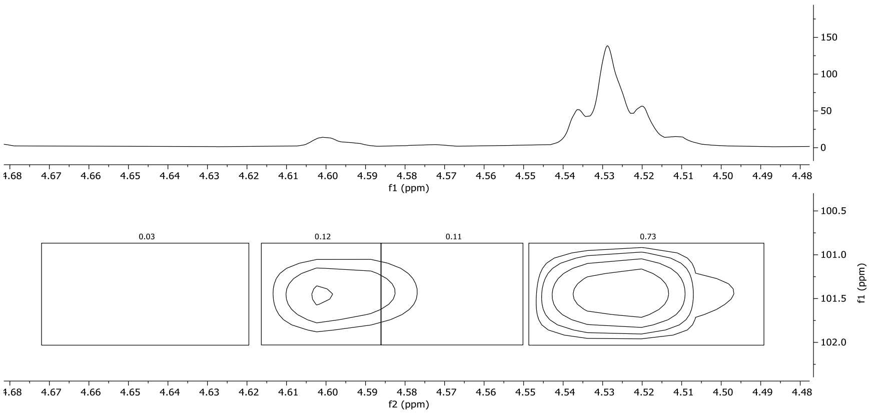

C

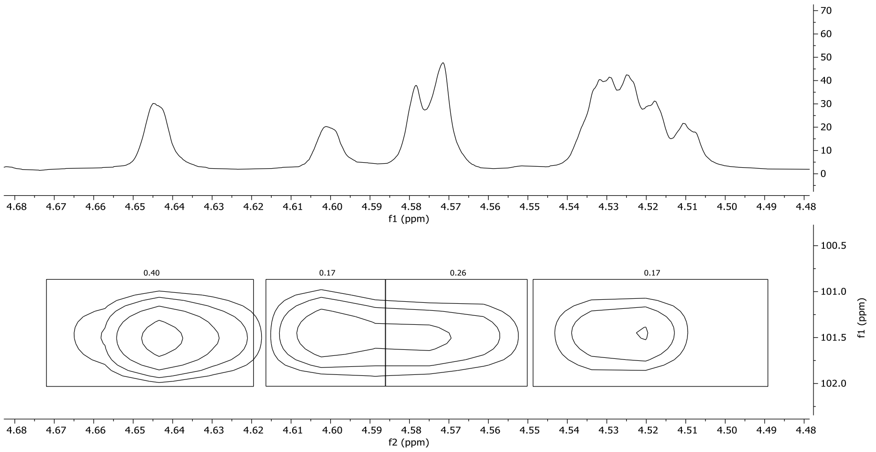

Figure S3: Expansion of the ^{1}H and ^{1}H,^{13}C HSQC NMR spectra (^{1}H: 600 MHz; ^{13}C: 126 MHz) of **2** (*c* = 2 mM in MeCN-d_3/DMSO-d_6, 8:2) after irradiation with light of the wavelength 520 nm (A), 365 nm (B), and 435 nm (C) for 5 min. Integration of the anomeric ^{1}H/^{13}C cross peak of the scaffold mannoside was used to determine the isomeric ratio in the PSS. From left to right: $E_{AB}E_{ABF4}$, $Z_{AB}E_{ABF4}$, $E_{AB}Z_{ABF4}$, and $Z_{AB}Z_{ABF4}$.

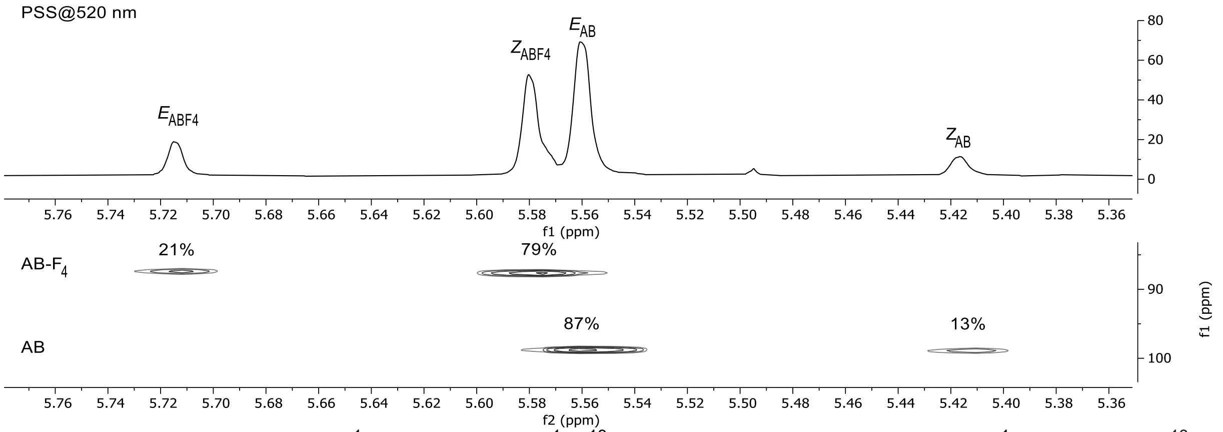

Figure S4: Expansion of the ^1H (top) and the $^1H,^{13}C$ HSQC NMR (bottom) spectra (^1H: 600 MHz; ^{13}C: 126 MHz) of **2** (c = 2 mM in MeCN-d_3/DMSO-d_6 8:2) after irradiation with light of the wavelength 520 nm for 5 min. Integration of the anomeric $^1H/^{13}C$ cross peaks of the AB and ABF_4 antennas was used to determine the isomeric *E*/*Z* ratio in the PSS.

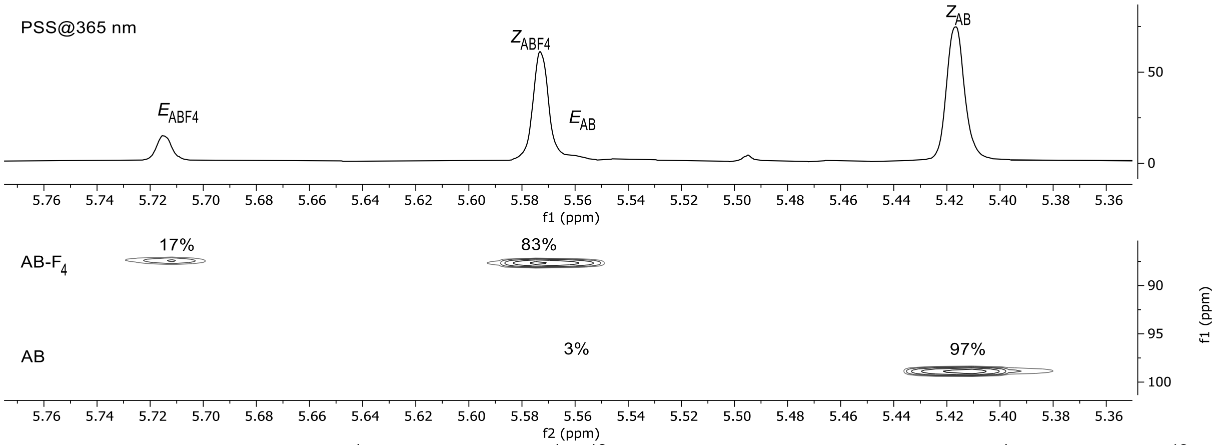

Figure S5: Expansion of the ^1H (top) and the $^1H,^{13}C$ HSQC NMR (bottom) spectra (^1H: 600 MHz; ^{13}C: 126 MHz) of **2** (c = 2 mM in MeCN-d_3/DMSO-d_6 8:2) after irradiation with light of the wavelength 365 nm for 5 min. Integration of the anomeric $^1H/^{13}C$ cross peaks of the AB and ABF_4 antennas was used to determine the isomeric *E*/*Z* ratio in the PSS.

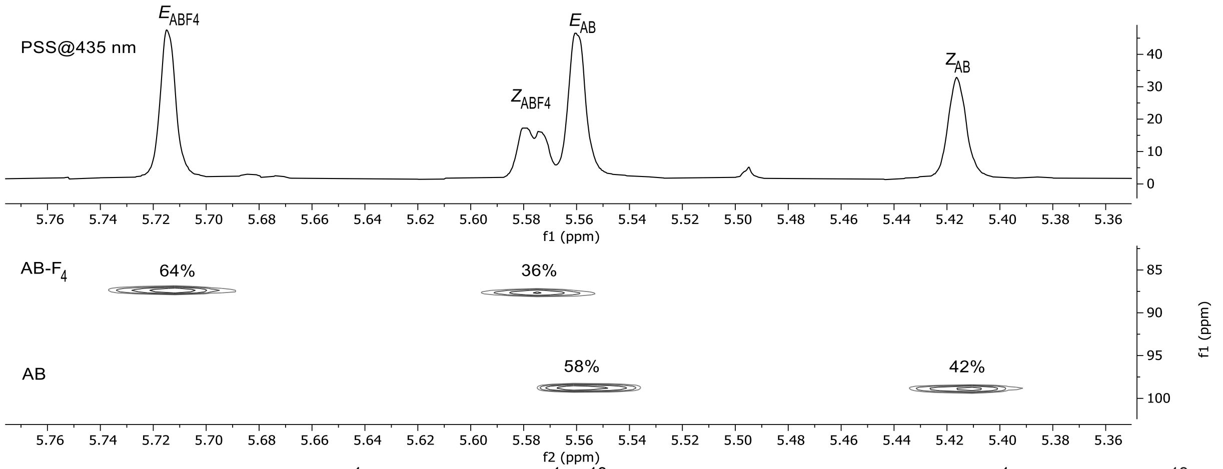

Figure S6: Expansion of the ^1H (top) and the $^1H,^{13}C$ HSQC NMR (bottom) spectra (^1H: 600 MHz; ^{13}C: 126 MHz) of **2** (c = 2 mM in MeCN-d_3/DMSO-d_6 8:2) after irradiation with light of the wavelength 435 nm for 5 min. Integration of the anomeric $^1H/^{13}C$ cross peaks of the AB and ABF_4 antennas was used to determine the isomeric *E*/*Z* ratio in the PSS.

2.2 6βGlc 3

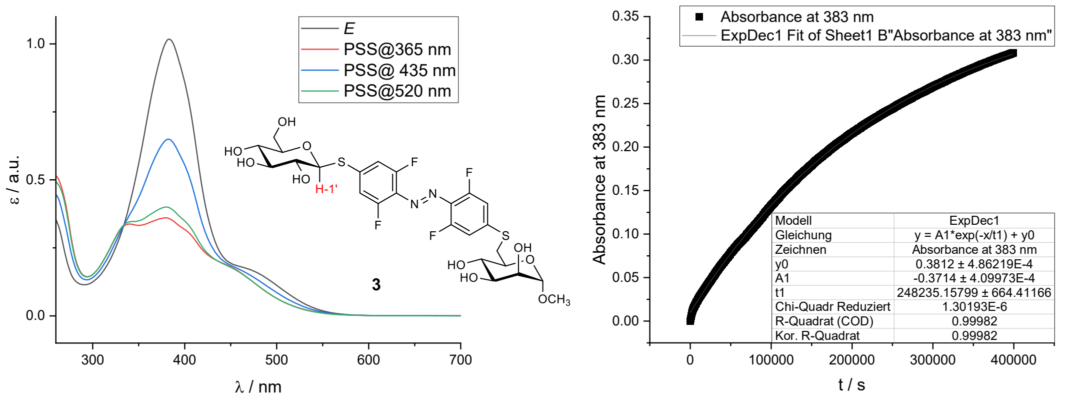

Figure S7: Left: UV–vis spectra of **3** at 25 °C in DMSO (25 µM) in *E* (black), and after 2 min of irradiation at 365 nm (red), 435 nm (blue), 520 nm (green); right: exponential growth of the absorbance at 365 nm of **3** after irradiation for 2 min with 365 nm light in DMSO (25 µM) at 37 °C.

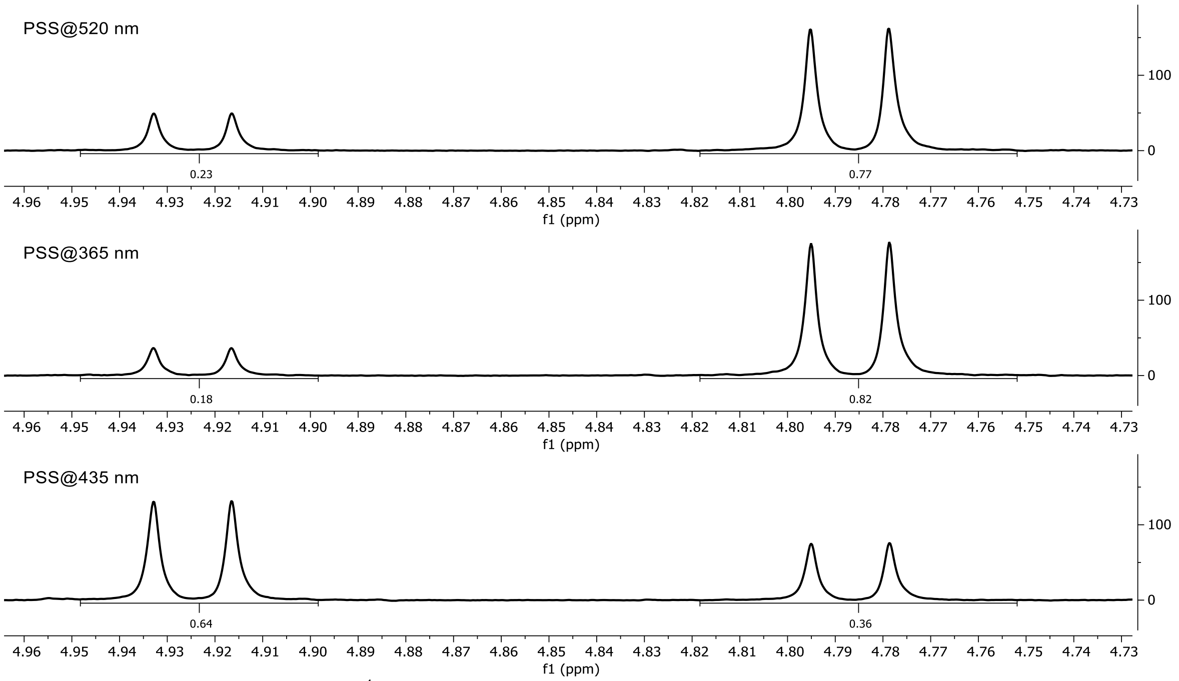

Figure S8: Expansion of the ^{1}H NMR (600 MHz) spectrum of **3** (*c* = 2 mM in MeCN-d_3/DMSO 8:2, recorded with an excitation sculpting suppression scheme (Bruker pulse sequence zgesgppe) and the center frequency was positioned on the DMSO-resonance (o1p = 2.54 ppm). Integration of the *E*-H-1' (4.92 ppm) and *Z*-H-1' (4.78 ppm) signals was used to determine the PSS values after irradiation with 520 nm, 365 nm, and 435 nm light for 5 min.

2.3 6αMan 4

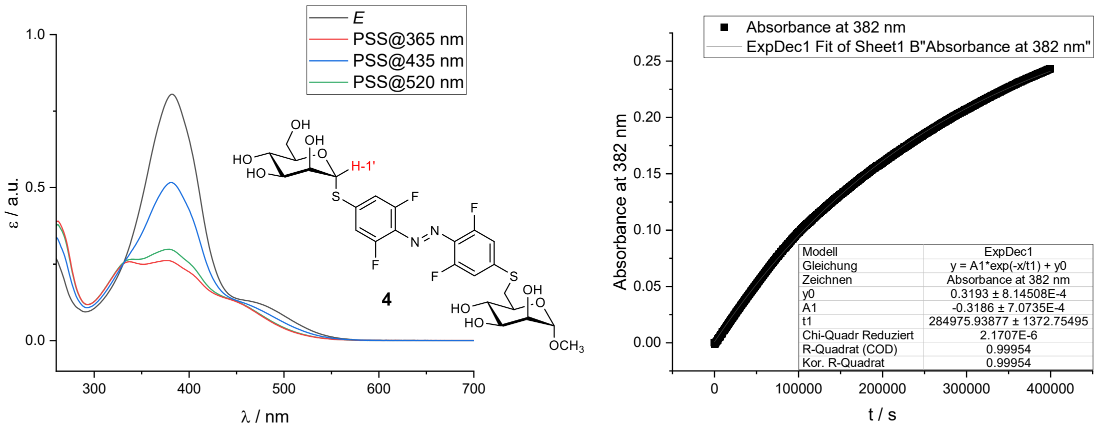

Figure S9: Left: UV–vis spectra of **4** at 25 °C in DMSO (25 µM) in *E* (black), and after 2 min of irradiation at 365 nm (red), 435 nm (blue), 520 nm (green); right: exponential growth of the absorbance at 365 nm of **4** after irradiation for 2 min with 365 nm light in DMSO (25 µM) at 37 °C.

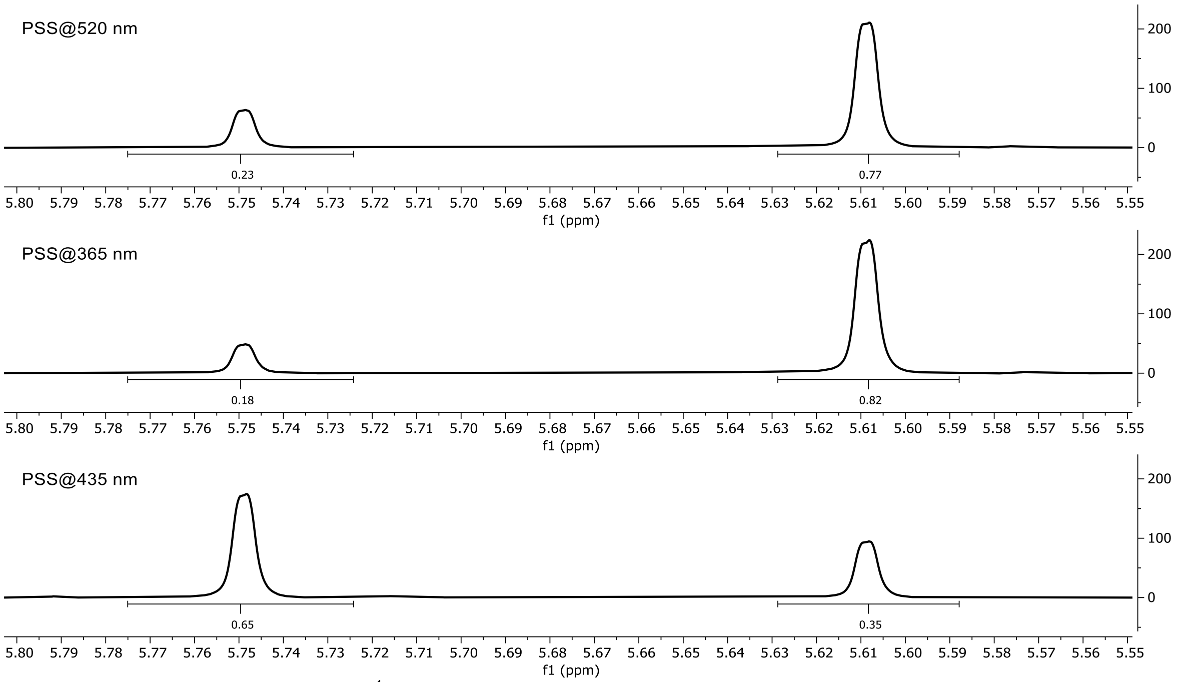

Figure S10: Expansion of the ^{1}H NMR (600 MHz) spectrum of **4** (*c* = 2 mM in MeCN-d_3/DMSO 8:2, recorded with an excitation sculpting suppression scheme (Bruker pulse sequence zgesgppe) and the center frequency was positioned on the DMSO-resonance (o1p=2.54 ppm). Integration of the *E*-H-1' (5.75 ppm) and *Z*-H-1' (5.61 ppm) signals was used to determine the PSS values after irradiation with 520 nm, 365 nm, and 435 nm light for 5 min.

2.4 3αMan 5

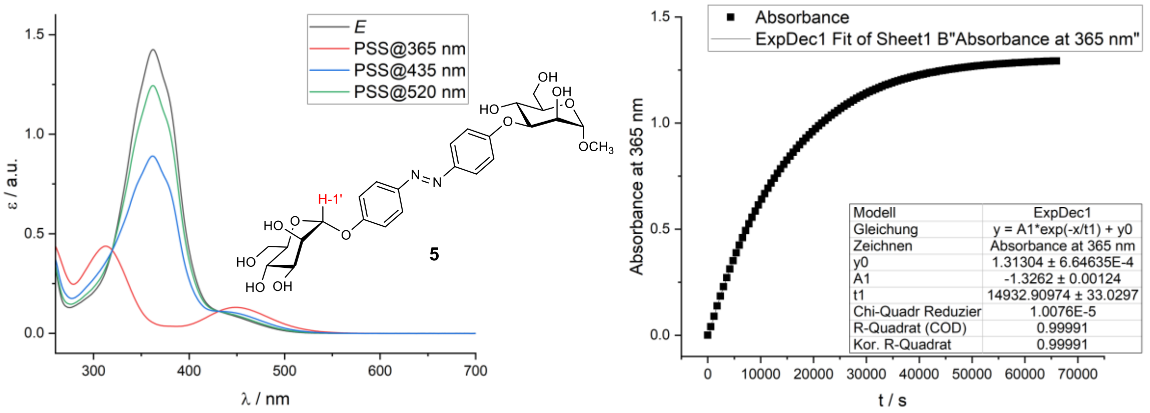

Figure S11: Left: UV–vis spectra of **5** at 25 °C in DMSO (50 µM) in *E* (black), and after 2 min of irradiation at 365 nm (red), 435 nm (blue), 520 nm (green); right: exponential growth of the absorbance at 365 nm of **5** after irradiation for 2 min with 365 nm light in DMSO (50 µM) at 37 °C.

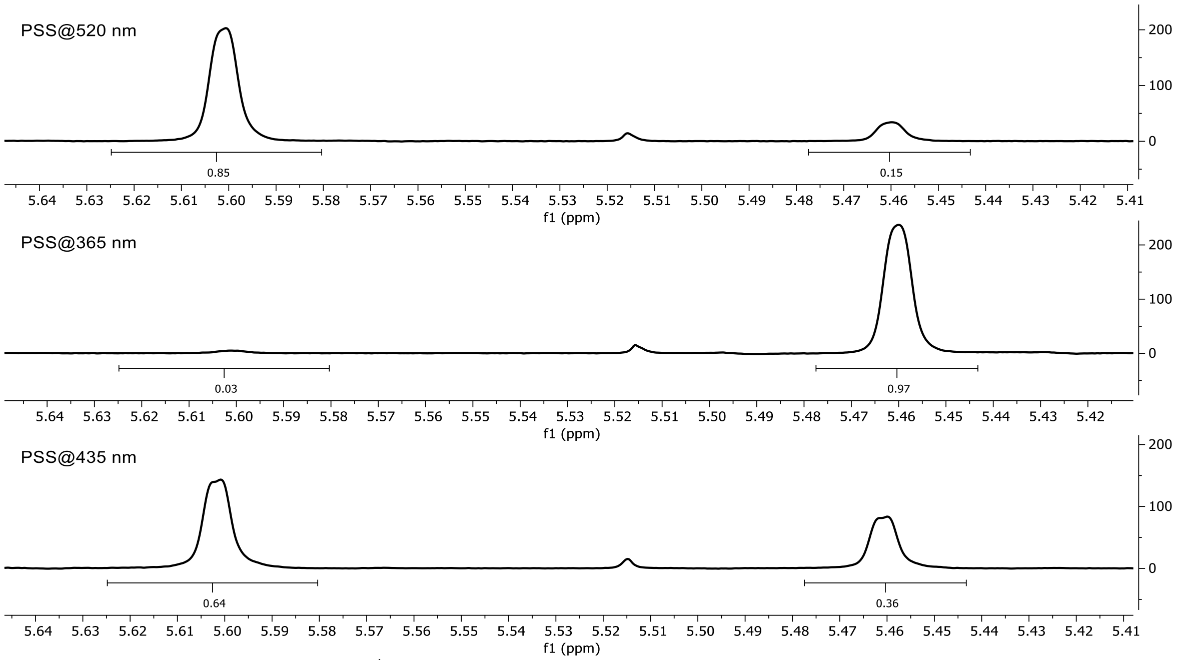

Figure S12: Expansion of the ^{1}H NMR (600 MHz) spectrum of **5** (*c* = 2 mM in MeCN-d_3/DMSO 8:2, recorded with an excitation sculpting suppression scheme (Bruker pulse sequence zgesgppe) and the center frequency was positioned on the DMSO-resonance (o1p=2.54 ppm). Integration of the *E*-H-1' (5.60 ppm) and *Z*-H-1' (5.46 ppm) signals was used to determine the PSS values after irradiation with 520 nm, 365 nm, and 435 nm light for 5 min.

3 Biological testing

3.1 General

The literature-known adhesion-inhibition assay [10] was adapted and modified as followed: Incubation took place in the GLF shaking incubator 3031. The bacteria were harvested using the Universal 320R centrifuge from Hettich Centrifuges, and the optical density (OD) was determined using the 7305 spectrophotometer from Jenway. Plates were washed using the HydroFlex™ plate washer from Tecan, and fluorescence spectroscopy was performed using the Infinite® M Nano plate reader, also from Tecan.

LB medium: In bidist. water (1 L) tryptone (10 g), yeast extract (5 g), NaCl (10 g) were dissolved and after autoclaving ampicillin (100 mg) and chloramphenicol (50 mg) were added as antibiotics.

PBS: In bidist. water (1 L) 2 PBS tablets (Gibco™) were dissolved. The final composition of the buffer contains NaCl (8 g), KCl (200 mg), $Na_2HPO_4*H_2O$ (1.44 g) and KH_2PO_4 (200 mg) with a pH of 7.2.

PBST: Tween®20 (0.5 mL, 0.05 %v/v) was added to 1 L of a PBS prepared as described above.

Carbonate buffer (pH 9.5): In bidist. water (1 L) Na_2CO_3 (1.59 g) and $NaHCO_3$ (2.52 g) were dissolved.

Bacteria: The GFP-expressing strain PKL1162 designed in the Klemm group [11] was created by introducing the palsmid pPKL174 into the SAR18 strain and was used for the binding assay. The pPKL174 plasmid contains the *fim* gene cluster, which is required for the expression of type 1 fimbriae, and the SAR18 strain carries the *gfp* gene on its chromosome, which is controlled by a constitutive promoter. The resulting bacterial strain PKL1162 expresses type 1 fimbriae as the only fimbriae type while the GPF expression enables readout by fluorescence.

Cultivation of bacteria: LB medium (10 mL) is inoculated with *E. coli* SAR18 PKL1162 from a frozen stock and incubated in a shaking incubator at 37 °C, 175 rpm overnight. Then, 5 mL of the bacterial solution was transferred to a new tube, filled up with LB medium (10 mL) and the bacterial solution was incubated at 37 °C, 100 rpm for 3 hours. The bacteria were harvested by centrifugation at 5000 rpm and 4 °C for 10 min and the supernatant was discarded. The pellet is washed by resuspending in 2 mL PBS, centrifuging at 5000 rpm and 4 °C for 10 min and removing the supernatant. A bacterial concentration of 2 mg/mL (OD600 = 0.40) was adjusted by resuspending and further dilution with PBS.

Mannan coating of microtiter plates: Mannan solution (120 µL/well with 1.2 mg/mL mannan from *Saccharomyces cerevisiae* in carbonate buffer) was added to a 96-well plate (Nunc™ Maxiorp®, flat bottom, black). The 96-well plate was desiccated overnight at 37 °C, 175 rpm. The functionalized plate was washed with PBST (3 × 400 µL/well) and filled with 120 µL/well of a 1% PVA solution in PBS. The plate was blocked at 37 °C and 100 rpm for 2 h with the lid closed and washed with PBS (3 × 400 µL/well).

Inhibition assay with GFP-PKL1162 *E. coli* bacteria: The photoswitches **1**–**5** were used as inhibitors of mannose-specific *E. coli* adhesion to mannan-coated microplates. Inhibition curves were obtained from serial dilutions (1:2, 50 µL/well over 6 to 7 steps, depending on the control section). In all assays methyl α-D-mannopyranoside (MeMan) was tested in parallel. Due to solubility problems, the samples had to be dissolved in DMSO and serial dilutions started with 2 mM DMSO solutions. Serial dilutions of MeMan, on the other hand, started with 200 mM solutions in DMSO.
On one plate, either the different isomeric states of the bis-azobenzene glycocluster **1** or **2**, respectively, namely the *EE* isomer (relaxed), PSS@520 nm, PSS@435 nm and PSS@365 nm were tested as

duplicates together with duplicates of MeMan and DMSO; or the photoswitches **3**, **4** and **5** as *E* isomer (relaxed) and PSS@365 nm as duplicates together with duplicates of MeMan and DMSO.
For thermal relaxation of the photoswitches (10 mM in DMSO) were stored for 3 d at 60 °C in the absence of light. To reach the respective PSS, the solutions were irradiated with the appropriate wavelength for 15 min and were directly applied (20 µL) to the micro plates and diluted with DMSO (80 µL) to achieve a concentration of 2 mM in the first well. Serial dilution was performed over the wells filled with PBS/DMSO (10%) (50 µL/well) and then, the prepared bacterial suspension (OD_{600} = 0.40, 50 µL/well) was added. The plate was incubated at 37 °C and 100 rpm for 45 min, washed with PBS (3 × 400 µL/well), filled up with PBS (100 µL/well) and the fluorescence (ex/em = 485/535 nm) was determined.

3.2 Inhibition curves resulting from adhesion-inhibition assays with *E. coli* bacteria (PKL1162)

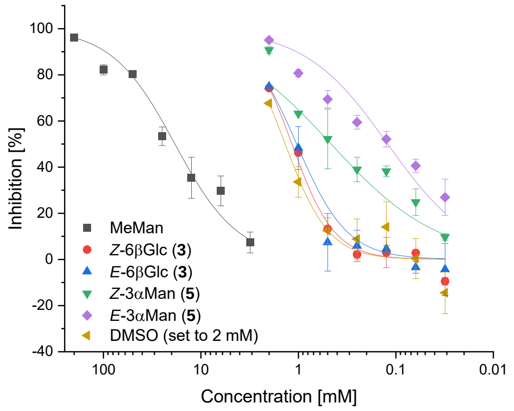

Figure S13: Inhibition curves obtained with the photoswitches 6βGlc **3** (cf. Table S1) and 3αMan **5** (cf. Table S3). MeMan and DMSO were tested in on the same plate. The start of the inhibition curve obtained with DMSO was set to 2 mM since the photoswitches were also employed as 2 mM solutions in DMSO. Serial dilutions in any case led to identical DMSO volume percentages per well. Standard deviations of duplicate results on the plate delivered the error bars. The obtained data were fitted by a non-linear regression, using a sigmoidal dose–responsive inhibition curve, fixing start and end value to 100 and 0, respectively.

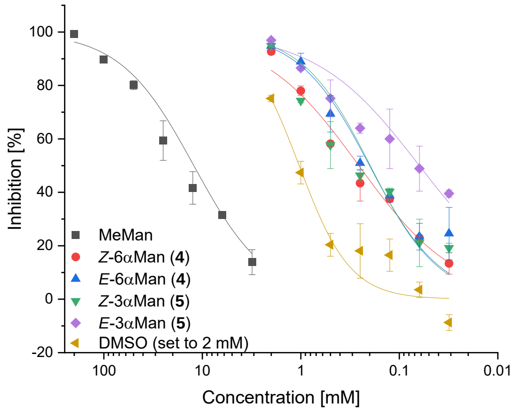

Figure S14: Inhibition curves obtained with the photoswitches 6αMan **4** (cf. Table S2) and 3αMan **5** (cf. Table S3). MeMan and DMSO were tested in on the same plate. The start of the inhibition curve obtained with DMSO was set to 2 mM since the photoswitches were also employed as 2 mM solutions in DMSO. Serial dilutions in any case led to identical DMSO volume percentages per well. Standard deviations of duplicate results on the plate delivered the error bars. The obtained data were fitted by a non-linear regression, using a sigmoidal dose-responsive inhibition curve, fixing start and end value to 100 and 0, respectively.

Table S1: IC_{50} values resulting from the inhibition curves of the adhesion-inhibition assay with *E*- and *Z*-6βGlc **3** and RIP obtained from MeMan or DMSO as reference, respectively.

Plate	Entry	MeMan	DMSO[a]	*E*-6βGlc 3[b]	*Z*-6βGlc 3[c]
A	IC_{50}[d] [mmol]	9.52 (±1.35)	0.71 (±0.11)	0.72 (±0.02)	0.95 (±0.09)
	RIP (MeMan)[e]	1.00		13.24 (±2.20)	10.05 (±2.38)
	RIP (DMSO)[e]		1.00	0.99 (±0.17)	0.75 (±0.18)
B	IC_{50}[d] [mmol]	18.47 (±2.42)	1.36 (±0.07)	1.04 (±0.07)	1.16 (±0.04)
	RIP(MeMan)[e]	1.00		17.75 (±3.55)	15.87 (±2.65)
	RIP(DMSO)[e]		1.00	1.31 (±0.16)	1.17 (±0.10)
	Mean RIP(MeMan)[f]	1.00		15.49 (±2.88)	12.96 (±2.52)
	Mean RIP(DMSO)[f]		1.00	1.15 (±0.17)	0.96 (±0.14)

[a] The concentration for plotting the inhibition of DMSO was set to 2 mM. [b] The *E* isomer was obtained by storing the inhibitor solution (10 mM in DMSO) at least for 3 d at 60 °C in the dark. [c] The *Z* isomer represents the PSS after irradiation for at least 15 min with 365 nm. [d] IC_{50} values are average values resulting from duplicate testing on one plate. The corresponding fitting errors are given in brackets. [e] Relative inhibition potency (RIP) values are based on methy α-D-mannopyranoside (MeMan) or dimethyl sulfoxide (DMSO) as a reference (IP ≡ 1), which were tested in on the same plates. RIP and error calculates as followed: $RIP = \frac{IC_{50}(\text{reference})}{IC_{50}(\text{inhibitor})}$; $\Delta RIP = \left|\frac{1}{IC_{50}(\text{inhibitor})} \times \Delta IC_{50}(\text{reference})\right| + \left|-\frac{IC_{50}(\text{reference})}{IC_{50}(\text{inhibitor})^2} \times \Delta IC_{50}(\text{inhibitor})\right|$. [f] Mean value of RIP from n independent experiments and the error in brackets calculated with: $\Delta Mean\ RIP = \sum_{i=1}^{n}\left|\frac{1}{n} \times \Delta RIP_i\right|$.

Table S2: IC_{50} values resulting from the inhibition curves of the adhesion-inhibition assay with *E*- and *Z*-6αMan **4** and RIP obtained from MeMan or DMSO as reference, respectively.

Plate	Entry	MeMan	DMSO[a]	*E*-6αMan 4[b]	*Z*-6αMan 4[c]
C	IC_{50}[d] [mmol]	18.86 (±4.72)	0.87 (±0.07)	0.09 (±0.01)	0.22 (±0.03)
	RIP(MeMan)[e]	1.00		215.91 (±70.79)	83.85 (±31.19)
	RIP(DMSO)[e]		1.00	10.02 (±1.63)	3.89 (±0.81)
D	IC_{50}[d] [mmol]	12.21 (±0.58)	1.04 (±0.10)	0.20 (±0.02)	0.26 (±0.03)
	RIP(MeMan)[e]	1.00		60.92 (±7.65)	47.39 (±7.44)
	RIP(DMSO)[e]		1.00	5.18 (±0.93)	4.03 (±0.85)
	Mean RIP(MeMan)[f]	1.00		138.46 (±39.22)	65.62 (±19.32)
	Mean RIP(DMSO)[f]		1.00	7.60 (±1.28)	3.96 (±0.83)

[a] The concentration for plotting the inhibition of DMSO was set to 2 mM. [b] The *E* isomer was obtained by storing the inhibitor solution (10 mM in DMSO) at least for 3 d at 60 °C in the dark. [c] The *Z* isomer represents the PSS after irradiation for at least 15 min with 365 nm. [d] IC_{50} values are average values resulting from duplicate testing on one plate. The corresponding fitting errors are given in brackets. [e] Relative inhibition potency (RIP) values are based on methy α-D-mannopyranoside (MeMan) or dimethyl sulfoxide (DMSO) as a reference (IP ≡ 1), which were tested in on the same plates. RIP and error calculates as followed: $RIP = \frac{IC_{50}(\text{reference})}{IC_{50}(\text{inhibitor})}$; $\Delta RIP = \left|\frac{1}{IC_{50}(\text{inhibitor})} \times \Delta IC_{50}(\text{reference})\right| + \left|-\frac{IC_{50}(\text{reference})}{IC_{50}(\text{inhibitor})^2} \times \Delta IC_{50}(\text{inhibitor})\right|$. [f] Mean value of RIP from n independent experiments and the error in brackets calculated with: $\Delta Mean\ RIP = \sum_{i=1}^{n}\left|\frac{1}{n} \times \Delta RIP_i\right|$.

Table S3: IC_{50} values resulting from the inhibition curves of the adhesion-inhibition assay with *E*- and *Z*-3αMan **5** and RIP obtained from MeMan or DMSO as reference, respectively.

Plate	Entry	MeMan	DMSO[a]	*E*-3αMan 5[b]	*Z*-3αMan 5[c]
A	IC_{50}[d] [mmol]	9.52 (±1.35)	0.71 (±0.11)	0.05 (±0.00)	0.17 (±0.06)
	RIP (MeMan)[e]	1.00		177.24 (±40.29)	55.02 (±28.41)
	RIP (DMSO)[e]		1.00	13.25 (±3.09)	4.11 (±2.15)
B	IC_{50}[d] [mmol]	18.47 (±2.42)	1.36 (±0.07)	0.12 (±0.03)	0.46 (±0.09)
	RIP(MeMan)[e]	1.00		152.46 (±56.43)	40.02 (±12.94)
	RIP(DMSO)[e]		1.00	11.26 (±3.27)	2.96 (±0.72)
C	IC_{50}[d] [mmol]	18.86 (±4.72)	0.87 (±0.07)	0.07 (±0.00)	0.21 (±0.02)
	RIP(MeMan)[e]	1.00		263.95 (±83.91)	87.74 (±32.03)
	RIP(DMSO)[e]		1.00	12.24 (±1.87)	4.07 (±0.81)
D	IC_{50}[d] [mmol]	12.21 (±0.58)	1.04 (±0.10)	0.06 (±0.02)	0.20 (±0.05)
	RIP(MeMan)[e]	1.00		195.04 (±63.01)	61.94 (±19.72)
	RIP(DMSO)[e]		1.00	16.60 (±6.25)	5.27 (±1.96)
	Mean RIP(MeMan)[f]	1.00		197.17 (±60.91)	61.18 (±23.28)
	Mean RIP(DMSO)[f]		1.00	13.34 (±3.62)	4.10 (±1.41)

[a] The concentration for plotting the inhibition of DMSO was set to 2 mM. [b] The *E* isomer was obtained by storing the inhibitor solution (10 mM in DMSO) at least for 3 d at 60 °C in the dark. [c] The *Z* isomer represents the PSS after irradiation for at least 15 min with 365 nm. [d] IC_{50} values are average values resulting from duplicate testing on one plate. The corresponding fitting errors are given in brackets. [e] Relative inhibition potency (RIP) values are based on methy α-D-mannopyranoside (MeMan) or dimethyl sulfoxide (DMSO) as a reference (IP ≡ 1), which were tested in on the same plates. RIP and error calculates as followed: $RIP = \frac{IC_{50}(\text{reference})}{IC_{50}(\text{inhibitor})}$; $\Delta RIP = \left|\frac{1}{IC_{50}(\text{inhibitor})} \times \Delta IC_{50}(\text{reference})\right| + \left|-\frac{IC_{50}(\text{reference})}{IC_{50}(\text{inhibitor})^2} \times \Delta IC_{50}(\text{inhibitor})\right|$. [f] Mean value of RIP from n independent experiments and the error in brackets calculated with: $\Delta Mean\ RIP = \sum_{i=1}^{n} \left|\frac{1}{n} \times \Delta RIP_i\right|$.

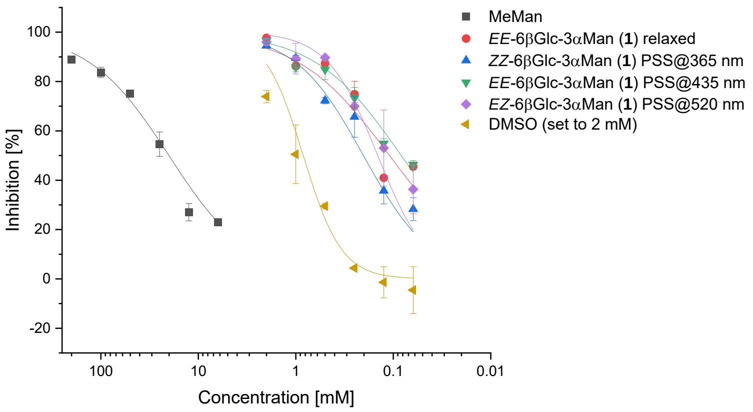

Figure S15: Inhibition curves obtained with the photoswitch 6βGlc3αMan **1** (cf. Table S4). MeMan and DMSO were tested in on the same plate. The start of the inhibition curve obtained with DMSO was set to 2 mM since the photoswitches were also employed as 2 mM solutions in DMSO. Serial dilutions in any case led to identical DMSO volume percentages per well. Standard deviations of duplicate results on the plate delivered the error bars. The obtained data were fitted by a non-linear regression, using a sigmoidal dose–responsive inhibition curve, fixing start and end value to 100 and 0, respectively.

Table S4: IC_{50} values resulting from the inhibition curves of the adhesion-inhibition assay with *EE*-(relaxed), *EE*-, *EZ*- and ZZ-6βGlc3αMan **1** and RIP obtained from MeMan or DMSO as reference, respectively.

Plate	Entry	MeMan	DMSO[a]	*EE*-6βGlc3αMan relaxed 1[b]	*EE*-6βGlc3αMan PSS@44 nm 1[c]	*EZ*-6βGlc3αMan PSS@520 nm 1[c]	*ZZ*-6βGlc3αMan PSS@365 nm 1[c]
A	IC_{50}[d] [mmol]	25.35 (±2.33)	1.05 (±0.32)	0.34 (±0.03)	0.30 (±0.05)	0.36 (±0.07)	0.33 (±0.09)
	RIP(MeMan)[e]	1.00		74.42 (±14.13)	83.61 (±21.14)	69.94 (±19.40)	76.93 (±27.65)
	RIP(DMSO)[e]		1.00	3.09 (±1.25)	3.48 (±1.63)	2.91 (±1.43)	3.20 (±1.84)
B	IC_{50}[d] [mmol]	20.05 (±1.41)	0.84 (±0.13)	0.11 (±0.04)	0.08 (±0.01)	0.14 (±0.02)	0.20 (±0.03)
	RIP(MeMan)[e]	1.00		183.41 (±86.29)	242.04 (±47.52)	139.08 (±26.90)	99.10 (±20.79)
	RIP(DMSO)[e]		1.00	7.67 (±4.27)	10.12 (±2.85)	5.82 (±1.62)	4.15 (±1.22)
C	IC_{50}[d] [mmol]	26.76 (±5.81)	1.48 (±0.66)	0.70 (±0.04)	0.34 (±0.05)	0.62 (±0.04)	0.52 (±0.06)
	RIP(MeMan)[e]	1.00		38.00 (±10.62)	79.03 (±29.83)	43.31 (±12.31)	51.34 (±16.85)
	RIP(DMSO)[e]		1.00	2.10 (±1.07)	4.36 (±2.64)	2.39 (±1.23)	2.83 (±1.58)
	Mean RIP(MeMan)[f]	1.00		98.61 (±37.01)	134.90 (±32.83)	84.11 (±19.54)	75.79 (±21.76)
	Mean RIP(DMSO)[f]		1.00	4.29 (±1.84)	5.33 (±2.02)	4.36 (±1.90)	3.39 (±1.38)

[a] The concentration for plotting the inhibition of DMSO was set to 2 mM. [b] The *E* isomer was obtained by storing the inhibitor solution (10 mM in DMSO) at least for 3 d at 60 °C in the dark. [c] The different isomeric mixtures are represented by the PSS after irradiation for at least 15 min with the given wavelength. [d] IC_{50} values are average values resulting from duplicate testing on one plate. The corresponding fitting errors are given in brackets. [e] Relative inhibition potency (RIP) values are based on methy α-D-mannopyranoside (MeMan) or dimethyl sulfoxide (DMSO) as a reference (IP ≡ 1), which were tested in on the same plates. RIP and error calculates as followed: $RIP = \frac{IC_{50}(\text{reference})}{IC_{50}(\text{inhibitor})}$; $\Delta RIP = \left|\frac{1}{IC_{50}(\text{inhibitor})} \times \Delta IC_{50}(\text{reference})\right| + \left|-\frac{IC_{50}(\text{reference})}{IC_{50}(\text{inhibitor})^2} \times \Delta IC_{50}(\text{inhibitor})\right|$. [f] Mean value of RIP from n independent experiments and the error in brackets calculated with: $\Delta Mean\,RIP = \sum_{i=1}^{n}\left|\frac{1}{n} \times \Delta RIP_i\right|$.

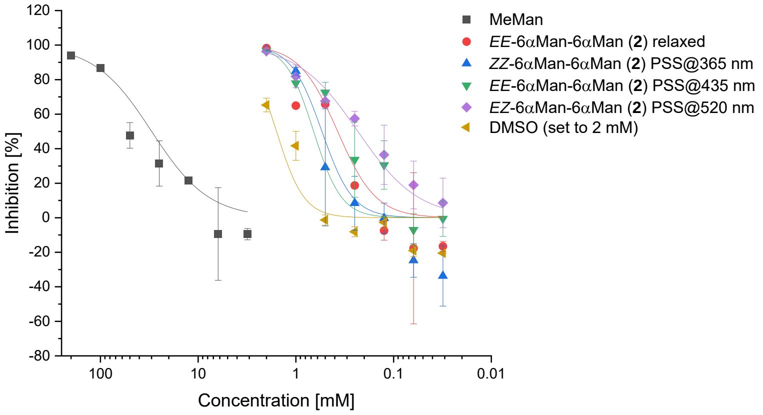

Figure S16: Inhibition curves obtained with the photoswitch 6αMan3αMan **2** (cf. Table S5). MeMan and DMSO were tested in on the same plate. The start of the inhibition curve obtained with DMSO was set to 2 mM since the photoswitches were also employed as 2 mM solutions in DMSO. Serial dilutions in any case led to identical DMSO volume percentages per well. Standard deviations of duplicate results on the plate delivered the error bars. The obtained data were fitted by a non-linear regression, using a sigmoidal dose–responsive inhibition curve, fixing start and end value to 100 and 0, respectively.

Table S5: IC_{50} values resulting from the inhibition curves of the adhesion-inhibition assay with *EE*-(relaxed), *EE*-, *EZ*- and ZZ-6αMan3αMan **2** and RIP obtained from MeMan or DMSO as reference, respectively.

Plate	Entry	MeMan	DMSO[a]	*EE*-6αMan3αMan relaxed 2[b]	*EE*-6αMan3αMan PSS@44 nm 2[c]	*EZ*-6αMan3αMan PSS@520 nm 2[c]	*ZZ*-6αMan3αMan PSS@365 nm 2[c]
A	IC_{50}[d] [mmol]	58.26 (±10.97)	1.37 (±2.07)	0.50 (±0.01)	0.51 (±0.06)	0.35 (±0.04)	0.50 (±0.07)
	RIP(MeMan)[e]	1.00		116.13 (±24.27)	114.91 (±34.99)	164.96 (±51.59)	117.04 (±38.12)
	RIP(DMSO)[e]		1.00	2.73 (±4.18)	2.70 (±4.39)	3.88 (±6.33)	2.75 (±4.53)
B	IC_{50}[d] [mmol]	29.92 (±1.52)	1.58 (±3.74)	0.37 (±0.04)	0.66 (±0.02)	0.23 (±0.03)	0.54 (±0.09)
	RIP(MeMan)[e]	1.00		80.10 (±11.81)	45.49 (±3.65)	129.06 (±23.50)	55.31 (±11.57)
	RIP(DMSO)[e]		1.00	4.22 (±10.43)	2.40 (±5.76)	6.81 (±17.05)	2.92 (±7.38)
	Mean RIP(MeMan)[f]	1.00		98.11 (±18.04)	80.20 (±19.32)	147.01 (±37.54)	86.18 (±24.84)
	Mean RIP(DMSO)[f]		1.00	3.48 (±5.22)	3.14 (±5.15)	4.75 (±10.72)	2.83 (±5.78)

[a] The concentration for plotting the inhibition of DMSO was set to 2 mM. [b] The *E* isomer was obtained by storing the inhibitor solution (10 mM in DMSO) at least for 3 d at 60 °C in the dark. [c] The different isomeric mixtures are represented by the PSS after irradiation for at least 15 min with the given wavelength. [d] IC_{50} values are average values resulting from duplicate testing on one plate. The corresponding fitting errors are given in brackets. [e] Relative inhibition potency (RIP) values are based on methy α-D-mannopyranoside (MeMan) or dimethyl sulfoxide (DMSO) as a reference (IP ≡ 1), which were tested in on the same plates. RIP and error calculates as followed: $RIP = \frac{IC_{50}(\text{reference})}{IC_{50}(\text{inhibitor})}$; $\Delta RIP = \left|\frac{1}{IC_{50}(\text{inhibitor})} \times \Delta IC_{50}(\text{reference})\right| + \left|-\frac{IC_{50}(\text{reference})}{IC_{50}(\text{inhibitor})^2} \times \Delta IC_{50}(\text{inhibitor})\right|$. [f] Mean value of RIP from n independent experiments and the error in brackets calculated with: $\Delta Mean\ RIP = \sum_{i=1}^{n} \left|\frac{1}{n} \times \Delta RIP_i\right|$.

Table S6: Mean relative inhibitory potencies (RIP) of the photoswitches 6αMan **3**, 6βGlc **4**, and 3αMan **5** in the *E* or *Z* state, respectively, and of the glycoclusters 6βGlc3αMan **1** and 6αMan3αMan **2** as *EE* isomer (relaxed) and as isomeric mixtures with the *EE*, *EZ*, or *ZZ* isomer predominating, respectively. The RIP values were obtained from duplicate results in adhesion-inhibition assays with the *E. coli* strain PKL1162 and are relative to MeMan and DMSO, respectively.

Inhibitor	Isomer[a]	Mean RIP(MeMan)[b]	Mean RIP(DMSO)[b]
6βGlc3αMan **1**	*EE* (relaxed)	98.61 (±37.01)	4.29 (±1.84)
	EE (435 nm)	134.90 (±32.83)	5.33 (±2.02)
	EZ (520 nm)	84.11 (±19.54)	4.36 (±1.90)
	ZZ (365 nm)	75.79 (±21.76)	3.39 (±1.38)
6αMan3αMan **2**	*EE* (relaxed)	98.11 (±18.04)	3.48 (±5.22)
	EE (435 nm)	80.20 (±19.32)	3.14 (±5.15)
	EZ (520 nm)	147.01 (±37.54)	4.75 (±10.72)
	ZZ (365 nm)	86.18 (±24.84)	2.83 (±5.78)
6βGlc **3**	*E* (relaxed)	15.49 (±2.88)	1.15 (±0.17)
	Z (520 nm)	12.96 (±2.52)	0.96 (±0.14)
6αMan **4**	*E* (relaxed)	138.46 (±39.22)	7.60 (±1.28)
	Z (520 nm)	65.62 (±19.32)	3.96 (±0.83)
3αMan **5**	*E* (relaxed)	197.17 (±60.91)	13.34 (±3.62)
	Z (365 nm)	61.18 (±23.28)	4.10 (±1.41)

[a]Irradiation of a 10 mM stock solution for at least 15 min with the specified wavelength; [b]Mean RIP values of at least two independent experiments are listed, relative to MeMan (with IP ≡ 1) and DMSO (with IP ≡ 1) as reference. MeMan and DMSO were tested on the same plate. RIP(inhibitor) = IC50(reference)/IC50(inhibitor), error propagation in brackets (cf. Tables S1–S5 and Figure 3).

4 Docking studies

General

The Maestro interface of the Schrödinger software package was used for the docking studies [12,13]. The glycoclusters 6βGlc3αMan **1** and 6αMan3αMan **5** as well as the antennas 6βGlc **3**, 6αMan **4**, and 3αMan **5** were prepared in the Ligand Builder. Protein structures of open and closed gate conformations of FimH were prepared using the protein preparation workflow [14].

In general, poses obtained from glide or induced fit (IFD) docking, where the glucosyl residue and not the mannosyl portion of the 6βGlc3αMan **4** glycocluster was bound into the binding pocket and poses showing only interactions with the periphery of the mannose-specific lectin FimH without any interactions with the binding pocket, were removed. All calculations were also performed for the 6βGlc **3** antenna to check the stability of binding into the binding pocket with the IFD-MD. In this case, poses with the glucose residue complexed in the binding pocket were tolerated.

Glide docking [15,16]

Receptor grids for the protein structures of the open (pdb: 1KLF) [17] and closed gate (pdb: 1UWF) [18] conformations of FimH were created using Gilde. Thereby the simulation box for ligands was set to 36 Å with a ligand diameter midpoint box of 30 × 30 × 30 Å. For the open-gate conformation, the grid was calculated with the OPLS4 [19] force field and for the closed-gate conformation the OPLS3e [20] force field was used. Tyrosine residues Tyr48 and Tyr137 were set rotatable and ligand docking was then performed with extra precision (XP). Ligands were set flexible for sampling with an energy window for ring sampling of 2.5 kcal/mol and nitrogen inversion as well as ring inversion were not allowed. Epic state penalties were added to the docking score. Up to 20 poses per ligand were obtained and subjected to post-docking minimization with a threshold for rejecting minimized poses of 0.50 kcal/mol. Duplicates were removed if the RMSD was less than 0.5 Å and the maximum atomic displacement was less than 1.3 Å. The results are shown in Table S7–Table S12.

The binding energy (ΔG_{Bind} in kcal/mol) of the up to five poses with the best glide docking score was calculated using Prime MM-GBSA [21] (molecular mechanics with generalized Born and surface-area solvation). For this purpose, the solvation model VSGB and the force field OPLS4 were used in a minimization sampling method. The results are shown in Table S13–Table S18.

Induced fit docking [22]

IFD was performed using the standard protocol and the OPLS_2005 [23] force field. The simulation box for ligands was set to 36 Å, an energy window of 2.5 kcal/mol was selected for the sample ring conformation and the conformation of non-planar amide bonds was restricted. Glide redocking with XP was performed for up to 20 structures separated by up to 30 kcal/mol from the best structure (Table S19–Table S21).

The top five poses of each receptor–ligand complex according to IFD soring were inserted into a binding pose metadynamic simulation [24] with ten trials per pose (Table S22–Table S24). The most stable receptor–ligand complexes with the lowest metadynamics composite score determined by this method were incorporated into a MM-GBSA calculation in order to calculate the binding energy (ΔG_{Bind} in kcal/mol) as described above (Table S25).

Table S7: Scoring values for docking of the isomers *EE*, *ZZ*, *EZ*, and *ZE* of glycocluster 6βGlc3αMan **1** into the closed-gate binding pocket of FimH (pdb: 1UWF) using Glide.

Isomer of 6βGlc3αMan 1	Docking Score	Glide evdw	Glide ecoul	Glide energy	Glide emodel	XP HBond
EE	-12.134	-40.078	-43.219	-83.297	-133.934	-9.255
EE	-11.759	-39.776	-43.800	-83.576	-133.154	-9.255
EE	-11.381	-33.637	-47.692	-81.329	-138.150	-9.255
EE	-9.785	-41.878	-39.418	-81.296	-135.911	-9.255
ZZ	-8.698	-44.545	-31.160	-75.704	-125.540	-6.523
ZZ	-8.637	-41.065	-35.215	-76.280	-128.842	-6.523
ZZ	-8.054	-44.848	-35.791	-80.640	-126.803	-6.523
ZZ	-7.379	-35.913	-36.155	-72.068	-127.765	-6.523
ZZ	-7.290	-44.744	-38.279	-83.022	-131.322	-6.523
ZZ	-7.287	-35.018	-38.472	-73.490	-127.064	-6.523
ZZ	-6.899	-41.333	-39.029	-80.362	-127.666	-6.523
ZZ	-6.576	-43.001	-37.792	-80.793	-130.283	-6.523
EZ	-12.728	-33.073	-52.457	-85.531	-140.249	-8.605
EZ	-10.658	-34.495	-48.095	-82.590	-136.760	-8.605
EZ	-10.619	-35.515	-43.095	-78.610	-135.176	-8.605
ZE	-10.651	-47.627	-39.281	-86.908	-134.135	-8.556
ZE	-10.325	-40.056	-43.233	-83.288	-139.305	-8.556
ZE	-9.250	-38.592	-40.946	-79.538	-132.055	-8.556
ZE	-9.197	-36.734	-38.900	-75.634	-133.993	-8.556
ZE	-8.443	-41.014	-37.052	-78.066	-133.654	-8.556
ZE	-8.403	-40.918	-37.322	-78.240	-134.118	-8.556
ZE	-8.088	-45.555	-37.719	-83.275	-135.985	-8.556
ZE	-7.266	-48.731	-34.083	-82.814	-143.107	-8.556
ZE	-7.245	-44.956	-36.019	-80.975	-134.354	-8.556
ZE	-7.222	-50.229	-37.575	-87.804	-145.635	-8.556
ZE	-7.143	-48.734	-33.384	-82.118	-145.795	-8.556
ZE	-7.123	-50.809	-30.739	-81.548	-143.918	-8.556
ZE	-7.075	-50.421	-31.485	-81.905	-142.235	-8.556
ZE	-6.840	-48.181	-34.339	-82.521	-138.061	-8.556
ZE	-6.775	-49.980	-31.498	-81.478	-142.628	-8.556
ZE	-6.268	-37.068	-36.396	-73.463	-135.059	-8.556
ZE	-6.005	-44.716	-36.055	-80.771	-134.988	-8.556
ZE	-4.490	-42.088	-30.477	-72.565	-133.845	-8.556

Table S8: Scoring values for docking of the isomers *EE*, *ZZ*, *EZ*, and *ZE* of glycocluster 6βGlc3αMan **1** into the open-gate binding pocket of FimH (pdb: 1KLF) using Glide.

**Isomer of 6βGlc3αMan 1**	**Docking Score**	**Glide evdw**	**Glide ecoul**	**Glide energy**	**Glide emodel**	**XP HBond**
EE	-10.520	-29.636	-38.981	-68.617	-84.424	-6.209
EE	-9.404	-29.740	-38.786	-68.526	-84.765	-6.209
EE	-8.801	-22.898	-31.795	-54.693	-88.429	-6.209
EE	-8.556	-26.773	-37.509	-64.282	-93.474	-6.209
EE	-8.189	-27.499	-30.152	-57.651	-89.289	-6.209
EE	-7.839	-27.393	-30.499	-57.892	-88.744	-6.209
EE	-7.631	-20.016	-40.341	-60.358	-87.071	-6.209
EE	-7.050	-19.357	-31.872	-51.229	-86.968	-6.209
ZZ	-11.787	-37.260	-36.442	-73.702	-99.752	-7.172
ZZ	-11.784	-43.023	-30.721	-73.744	-100.189	-7.172
ZZ	-11.705	-40.220	-32.139	-72.360	-100.069	-7.172
ZZ	-11.503	-38.224	-33.455	-71.680	-100.705	-7.172
ZZ	-11.468	-41.113	-31.757	-72.869	-101.223	-7.172
ZZ	-11.302	-38.761	-33.137	-71.898	-101.399	-7.172
ZZ	-11.183	-43.170	-30.780	-73.950	-99.693	-7.172
ZZ	-10.877	-43.759	-30.802	-74.561	-103.508	-7.172
ZZ	-10.724	-39.890	-35.012	-74.902	-100.576	-7.172
EZ	-7.452	-28.763	-26.601	-55.364	-65.633	-4.814
EZ	-6.920	-26.718	-25.938	-52.656	-66.157	-4.814
EZ	-6.824	-29.574	-25.401	-54.975	-66.163	-4.814
EZ	-6.613	-27.899	-25.445	-53.344	-69.576	-4.814
EZ	-6.353	-27.908	-24.215	-52.122	-69.087	-4.814
EZ	-6.329	-32.044	-24.439	-56.483	-65.946	-4.814
EZ	-6.209	-27.704	-25.450	-53.154	-65.627	-4.814
EZ	-6.096	-32.508	-23.233	-55.741	-70.326	-4.814
EZ	-5.787	-32.252	-23.790	-56.042	-65.406	-4.814
EZ	-5.186	-29.133	-22.520	-51.653	-65.188	-4.814
ZE	-10.942	-40.799	-32.299	-73.098	-90.210	-8.099
ZE	-10.703	-37.832	-37.551	-75.383	-87.728	-8.099
ZE	-10.362	-41.311	-31.827	-73.138	-89.726	-8.099
ZE	-8.774	-30.398	-32.074	-62.472	-88.099	-8.099
ZE	-8.168	-29.498	-32.813	-62.310	-87.596	-8.099

Table S9: Scoring values for docking of the isomers *EE*, *ZZ*, *EZ*, and *ZE* of glycocluster 6αMan3αMan **2** into the closed-gate binding pocket of FimH (pdb: 1UWF) using Glide.

**Isomer of 6αMan3αMan 2**	**Docking Score**	**Glide evdw**	**Glide ecoul**	**Glide energy**	**Glide emodel**	**XP HBond**
EE	-10.154	-38.295	-38.707	-77.002	-125.024	-7.263
EE	-9.904	-38.183	-38.611	-76.794	-122.609	-7.263
EE	-8.143	-43.747	-30.035	-73.783	-121.766	-7.263
EE	-8.102	-39.402	-32.909	-72.312	-120.091	-7.263
EE	-7.813	-38.706	-32.647	-71.353	-119.805	-7.263
ZZ	-10.528	-34.694	-47.137	-81.831	-146.436	-8.627
ZZ	-10.418	-35.347	-48.526	-83.872	-138.899	-8.627
ZZ	-9.934	-33.251	-50.152	-83.403	-143.896	-8.627
EZ	-11.273	-30.282	-38.069	-68.351	-124.077	-7.077
EZ	-10.939	-37.223	-38.659	-75.882	-121.399	-7.077
EZ	-10.782	-34.063	-40.651	-74.715	-126.278	-7.077
EZ	-10.293	-22.222	-44.057	-66.279	-122.249	-7.077
EZ	-9.748	-45.765	-32.631	-78.396	-124.303	-7.077
EZ	-9.728	-33.549	-33.902	-67.451	-121.598	-7.077
EZ	-9.622	-31.354	-35.271	-66.625	-120.996	-7.077
EZ	-9.611	-40.949	-38.094	-79.043	-126.118	-7.077
EZ	-9.596	-43.017	-36.516	-79.533	-124.946	-7.077
EZ	-9.570	-42.624	-36.789	-79.412	-125.618	-7.077
EZ	-9.507	-40.859	-37.959	-78.819	-124.107	-7.077
EZ	-9.471	-38.942	-36.311	-75.254	-126.867	-7.077
EZ	-9.442	-40.000	-31.210	-71.210	-121.038	-7.077
EZ	-9.252	-34.532	-37.178	-71.710	-120.886	-7.077
EZ	-8.786	-46.137	-29.281	-75.418	-121.461	-7.077
EZ	-8.727	-42.424	-36.239	-78.663	-125.527	-7.077
EZ	-8.465	-26.546	-37.997	-64.543	-121.485	-7.077
EZ	-8.334	-43.931	-35.492	-79.423	-122.153	-7.077
EZ	-7.625	-42.569	-37.047	-79.616	-121.227	-7.077
ZE	-10.181	-44.422	-35.387	-79.809	-138.868	-8.428
ZE	-9.873	-41.120	-39.161	-80.281	-136.343	-8.428
ZE	-9.360	-43.039	-42.717	-85.756	-138.296	-8.428
ZE	-8.525	-36.969	-39.700	-76.669	-136.011	-8.428
ZE	-8.382	-39.865	-38.234	-78.099	-133.268	-8.428
ZE	-8.281	-41.773	-41.706	-83.479	-138.840	-8.428

Table S10: Scoring values for docking of the isomers *EE*, *ZZ*, *EZ*, and *ZE* of glycocluster 6αMan3αMan **2** into the open-gate binding pocket of FimH (pdb: 1KLF) using Glide.

**Isomer of 6αMan3αMan 2**	**Docking Score**	**Glide evdw**	**Glide ecoul**	**Glide energy**	**Glide emodel**	**XP HBond**
EE	-8.726	-40.172	-31.645	-71.817	-96.544	-6.653
EE	-8.389	-30.871	-35.869	-66.740	-99.201	-6.653
EE	-8.289	-39.840	-31.314	-71.155	-94.379	-6.653
ZZ	-10.035	-39.787	-30.833	-70.620	-88.095	-6.884
ZZ	-9.753	-37.268	-33.253	-70.521	-95.222	-6.884

ZZ	-9.510	-37.942	-33.012	-70.954	-92.102	-6.884
ZZ	-9.267	-39.032	-32.637	-71.669	-95.953	-6.884
ZZ	-9.166	-38.231	-32.000	-70.231	-88.996	-6.884
ZZ	-9.060	-38.718	-30.632	-69.350	-94.428	-6.884
ZZ	-8.999	-37.802	-31.650	-69.451	-87.269	-6.884
EZ	-11.233	-33.558	-32.636	-66.194	-84.099	-6.484
EZ	-11.087	-33.047	-34.426	-67.473	-85.734	-6.484
EZ	-11.002	-32.938	-34.361	-67.298	-86.615	-6.484
EZ	-10.995	-32.327	-34.245	-66.572	-86.794	-6.484
EZ	-10.984	-31.059	-34.587	-65.646	-86.712	-6.484
EZ	-10.980	-31.687	-34.387	-66.073	-81.976	-6.484
EZ	-10.924	-32.879	-34.591	-67.470	-83.872	-6.484
EZ	-10.916	-31.794	-34.493	-66.287	-84.415	-6.484
EZ	-10.912	-33.656	-33.403	-67.059	-84.818	-6.484
EZ	-10.911	-33.003	-33.766	-66.770	-86.434	-6.484
EZ	-10.806	-32.505	-34.333	-66.838	-78.540	-6.484
EZ	-10.774	-32.696	-34.191	-66.887	-83.239	-6.484
EZ	-10.011	-33.747	-29.255	-63.002	-78.473	-6.484
ZE	-9.862	-30.311	-36.716	-67.026	-93.599	-5.885
ZE	-9.778	-30.504	-36.704	-67.207	-90.123	-5.885
ZE	-9.678	-31.132	-35.834	-66.966	-88.426	-5.885
ZE	-9.558	-31.282	-36.167	-67.449	-89.895	-5.885
ZE	-9.288	-32.635	-33.123	-65.758	-93.799	-5.885
ZE	-9.198	-31.496	-34.970	-66.466	-89.588	-5.885
ZE	-9.159	-30.696	-38.048	-68.744	-88.285	-5.885
ZE	-9.110	-32.466	-31.334	-63.800	-88.163	-5.885
ZE	-9.107	-32.043	-36.529	-68.572	-90.154	-5.885
ZE	-9.014	-32.945	-33.881	-66.826	-94.121	-5.885
ZE	-8.976	-33.139	-33.004	-66.144	-88.803	-5.885
ZE	-8.882	-32.991	-32.889	-65.880	-88.810	-5.885
ZE	-8.785	-31.548	-37.291	-68.839	-88.232	-5.885
ZE	-8.747	-33.664	-35.513	-69.177	-93.590	-5.885
ZE	-8.720	-29.430	-37.414	-66.843	-90.477	-5.885
ZE	-8.620	-33.247	-33.596	-66.842	-87.815	-5.885
ZE	-8.393	-31.955	-33.402	-65.357	-92.865	-5.885
ZE	-8.332	-32.588	-33.885	-66.473	-87.968	-5.885
ZE	-8.178	-30.543	-32.886	-63.429	-93.776	-5.885
ZE	-7.338	-31.332	-31.325	-62.656	-89.838	-5.885

Table S11: Scoring values for docking of the antennas 6βGlc **3**, 6αMan **4**, and 3αMan **5** as their *E* and *Z* isomer, respectively, into the closed-gate binding pocket of FimH (pdb: 1UWF) using Glide.

**Antennas**	**Docking Score**	**Glide evdw**	**Glide ecoul**	**Glide energy**	**Glide emodel**	**XP HBond**
E-6βGlc **3**	-8.926	-31.115	-30.647	-61.762	-93.472	-4.502
E-6βGlc **3**	-8.797	-27.558	-29.445	-57.003	-89.324	-4.502
E-6βGlc **3**	-8.779	-28.684	-29.098	-57.782	-92.097	-4.502
E-6βGlc **3**	-8.653	-32.607	-28.437	-61.043	-96.017	-4.502
E-6βGlc **3**	-8.638	-31.605	-28.986	-60.591	-92.965	-4.502
E-6βGlc **3**	-8.357	-33.550	-26.241	-59.791	-90.512	-4.502
E-6βGlc **3**	-8.348	-32.196	-28.760	-60.956	-94.637	-4.502
E-6βGlc **3**	-8.348	-33.030	-25.253	-58.283	-92.706	-4.502
E-6βGlc **3**	-8.336	-31.633	-30.574	-62.207	-93.768	-4.502
E-6βGlc **3**	-8.022	-32.679	-24.381	-57.061	-91.915	-4.502
Z-6βGlc **3**	-9.367	-27.215	-28.798	-56.013	-77.811	-5.120
Z-6βGlc **3**	-9.288	-26.610	-28.331	-54.941	-77.851	-5.120
Z-6βGlc **3**	-9.091	-26.513	-30.003	-56.517	-82.245	-5.120
Z-6βGlc **3**	-8.949	-25.728	-27.871	-53.599	-82.450	-5.120
Z-6βGlc **3**	-8.865	-23.820	-31.253	-55.073	-81.559	-5.120
Z-6βGlc **3**	-8.685	-24.178	-29.563	-53.740	-81.704	-5.120
Z-6βGlc **3**	-8.665	-26.522	-29.261	-55.783	-86.588	-5.120
Z-6βGlc **3**	-8.447	-27.558	-26.772	-54.330	-78.835	-5.120
Z-6βGlc **3**	-8.436	-26.247	-24.578	-50.825	-78.736	-5.120
Z-6βGlc **3**	-8.050	-25.327	-29.038	-54.365	-81.596	-5.120
Z-6βGlc **3**	-7.971	-27.282	-29.103	-56.385	-81.392	-5.120
Z-6βGlc **3**	-7.525	-30.978	-23.087	-54.066	-75.772	-5.120
Z-6βGlc **3**	-7.443	-26.051	-29.235	-55.285	-79.620	-5.120
Z-6βGlc **3**	-6.549	-17.925	-36.863	-54.788	-82.130	-5.120
E-6αMan **4**	-10.222	-33.869	-24.375	-58.245	-86.587	-5.834
E-6αMan **4**	-9.949	-33.514	-24.365	-57.880	-86.236	-5.834
E-6αMan **4**	-9.929	-33.038	-24.163	-57.201	-87.471	-5.834
E-6αMan **4**	-9.917	-33.324	-24.199	-57.523	-91.963	-5.834
E-6αMan **4**	-9.904	-32.955	-23.312	-56.267	-86.917	-5.834
E-6αMan **4**	-9.839	-34.887	-22.528	-57.415	-90.616	-5.834
E-6αMan **4**	-9.820	-34.469	-21.360	-55.829	-86.133	-5.834
E-6αMan **4**	-9.755	-36.038	-20.505	-56.543	-88.248	-5.834
E-6αMan **4**	-9.704	-33.472	-24.005	-57.477	-89.496	-5.834
E-6αMan **4**	-9.699	-32.922	-23.967	-56.889	-87.907	-5.834
Z-6αMan **4**	-9.340	-29.371	-27.340	-56.711	-89.581	-5.870
Z-6αMan **4**	-8.807	-24.236	-34.561	-58.797	-85.420	-5.870
Z-6αMan **4**	-8.711	-30.004	-27.646	-57.650	-83.117	-5.870
Z-6αMan **4**	-7.918	-26.879	-25.653	-52.532	-81.706	-5.870
Z-6αMan **4**	-7.882	-26.455	-30.200	-56.655	-86.122	-5.870
Z-6αMan **4**	-7.859	-27.339	-28.184	-55.523	-85.183	-5.870
E-3αMan **5**	-9.980	-28.869	-20.160	-49.029	-79.138	-5.391
E-3αMan **5**	-9.915	-29.785	-20.408	-50.193	-82.440	-5.391
E-3αMan **5**	-9.370	-26.921	-26.077	-52.998	-84.162	-5.391

E-3αMan **5**	-9.332	-30.006	-21.448	-51.454	-83.869	-5.391
E-3αMan **5**	-9.307	-30.745	-21.515	-52.259	-85.108	-5.391
E-3αMan **5**	-9.286	-29.919	-21.410	-51.329	-84.303	-5.391
E-3αMan **5**	-9.240	-30.954	-21.188	-52.142	-85.078	-5.391
E-3αMan **5**	-9.212	-29.915	-22.234	-52.149	-84.747	-5.391
E-3αMan **5**	-9.182	-30.074	-21.498	-51.572	-84.629	-5.391
E-3αMan **5**	-9.174	-31.049	-20.907	-51.956	-84.003	-5.391
E-3αMan **5**	-9.089	-29.858	-22.918	-52.776	-85.496	-5.391
E-3αMan **5**	-9.056	-27.803	-22.529	-50.331	-83.220	-5.391
E-3αMan **5**	-9.040	-28.526	-26.591	-55.117	-83.003	-5.391
E-3αMan **5**	-9.014	-29.696	-21.481	-51.177	-83.545	-5.391
E-3αMan **5**	-8.995	-28.663	-22.154	-50.817	-81.607	-5.391
E-3αMan **5**	-8.900	-30.040	-21.073	-51.113	-86.708	-5.391
E-3αMan **5**	-8.790	-30.424	-22.553	-52.977	-85.730	-5.391
E-3αMan **5**	-8.760	-27.848	-23.696	-51.544	-81.796	-5.391
E-3αMan **5**	-7.923	-26.823	-21.999	-48.822	-81.235	-5.391
E-3αMan **5**	-7.110	-29.555	-21.519	-51.073	-83.801	-5.391
Z-3αMan **5**	-9.650	-24.253	-33.593	-57.846	-92.167	-6.966
Z-3αMan **5**	-9.601	-33.068	-25.387	-58.454	-92.505	-6.966
Z-3αMan **5**	-9.519	-30.119	-28.743	-58.862	-86.155	-6.966
Z-3αMan **5**	-9.491	-26.067	-34.704	-60.771	-88.772	-6.966
Z-3αMan **5**	-9.340	-28.269	-34.762	-63.030	-87.947	-6.966
Z-3αMan **5**	-9.228	-24.387	-31.781	-56.168	-87.759	-6.966
Z-3αMan **5**	-8.832	-27.791	-26.009	-53.799	-86.555	-6.966

Table S12: Scoring values for docking of the antennas 6βGlc **3**, 6αMan **4**, and 3αMan **5** as their *E* and *Z* isomer, respectively, into the open-gate binding pocket of FimH (pdb: 1KLF) using Glide.

Antennas	Docking Score	Glide evdw	Glide ecoul	Glide energy	Glide emodel	XP HBond
E-6βGlc **3**	-8.552	-24.148	-24.744	-48.892	-62.144	-3.245
E-6βGlc **3**	-8.499	-24.219	-28.320	-52.539	-67.442	-3.245
E-6βGlc **3**	-7.926	-19.090	-29.383	-48.473	-63.815	-3.245
E-6βGlc **3**	-7.808	-23.254	-28.143	-51.397	-61.241	-3.245
E-6βGlc **3**	-7.772	-21.787	-30.166	-51.953	-62.274	-3.245
E-6βGlc **3**	-7.734	-20.503	-30.320	-50.824	-62.739	-3.245
E-6βGlc **3**	-7.694	-21.760	-29.016	-50.776	-64.270	-3.245
E-6βGlc **3**	-7.429	-19.412	-29.353	-48.766	-61.907	-3.245
E-6βGlc **3**	-7.327	-21.391	-27.183	-48.574	-62.462	-3.245
E-6βGlc **3**	-6.096	-20.972	-25.388	-46.360	-63.186	-3.245
Z-6βGlc **3**	-6.489	-20.480	-17.848	-38.328	-40.339	-3.088
Z-6βGlc **3**	-6.020	-19.766	-19.242	-39.008	-43.393	-3.088
Z-6βGlc **3**	-5.803	-21.073	-17.502	-38.574	-38.664	-3.088
Z-6βGlc **3**	-5.610	-21.252	-17.767	-39.019	-39.539	-3.088
E-6αMan **4**	-10.440	-24.343	-33.680	-58.023	-85.282	-7.175
E-6αMan **4**	-10.117	-24.086	-34.034	-58.120	-80.638	-7.175
E-6αMan **4**	-10.114	-22.821	-34.358	-57.179	-85.315	-7.175
E-6αMan **4**	-10.108	-26.849	-25.407	-52.256	-78.577	-7.175
E-6αMan **4**	-9.977	-27.487	-31.603	-59.090	-89.899	-7.175
E-6αMan **4**	-9.882	-25.016	-33.933	-58.949	-78.728	-7.175
E-6αMan **4**	-9.765	-26.012	-32.745	-58.757	-80.242	-7.175
Z-6αMan **4**	-10.190	-23.577	-33.740	-57.317	-65.452	-4.454
Z-6αMan **4**	-10.175	-23.782	-28.356	-52.138	-66.770	-4.454
Z-6αMan **4**	-10.164	-24.072	-32.785	-56.857	-59.530	-4.454
Z-6αMan **4**	-9.758	-24.458	-31.905	-56.363	-67.059	-4.454
Z-6αMan **4**	-9.528	-24.995	-31.840	-56.835	-61.214	-4.454
Z-6αMan **4**	-9.419	-23.779	-24.496	-48.276	-61.182	-4.454
Z-6αMan **4**	-9.279	-25.851	-29.768	-55.619	-61.331	-4.454
Z-6αMan **4**	-7.373	-19.786	-30.633	-50.420	-61.448	-4.454
E-3αMan **5**	-8.195	-15.688	-29.137	-44.825	-64.691	-4.815
E-3αMan **5**	-8.190	-14.864	-30.436	-45.300	-63.978	-4.815
E-3αMan **5**	-8.155	-14.060	-30.525	-44.584	-64.401	-4.815
E-3αMan **5**	-7.922	-22.666	-24.696	-47.362	-63.292	-4.815
E-3αMan **5**	-7.719	-21.510	-25.151	-46.660	-65.095	-4.815
E-3αMan **5**	-7.503	-21.373	-25.254	-46.627	-64.786	-4.815
E-3αMan **5**	-7.374	-21.892	-25.279	-47.171	-63.152	-4.815
E-3αMan **5**	-7.325	-22.027	-25.422	-47.449	-63.487	-4.815
E-3αMan **5**	-7.014	-22.967	-24.971	-47.938	-62.762	-4.815
E-3αMan **5**	-6.745	-21.130	-25.431	-46.562	-63.447	-4.815
E-3αMan **5**	-6.740	-23.425	-24.315	-47.740	-68.326	-4.815
E-3αMan **5**	-6.629	-21.987	-25.250	-47.238	-62.899	-4.815
E-3αMan **5**	-6.629	-22.057	-25.361	-47.419	-62.973	-4.815
E-3αMan **5**	-5.357	-21.402	-25.161	-46.563	-63.363	-4.815

Z-3αMan **5**	-10.375	-20.605	-33.940	-54.545	-78.253	-7.127
Z-3αMan **5**	-10.029	-18.987	-34.697	-53.684	-79.079	-7.127
Z-3αMan **5**	-10.026	-20.384	-32.779	-53.162	-79.100	-7.127
Z-3αMan **5**	-9.965	-20.524	-31.549	-52.074	-76.042	-7.127
Z-3αMan **5**	-9.869	-21.874	-31.298	-53.172	-78.544	-7.127
Z-3αMan **5**	-9.758	-20.548	-30.477	-51.024	-76.602	-7.127
Z-3αMan **5**	-9.699	-20.665	-32.962	-53.627	-77.678	-7.127
Z-3αMan **5**	-9.390	-21.640	-31.168	-52.808	-78.262	-7.127
Z-3αMan **5**	-9.262	-20.367	-33.544	-53.911	-77.755	-7.127
Z-3αMan **5**	-9.219	-21.344	-29.850	-51.193	-77.158	-7.127
Z-3αMan **5**	-8.162	-23.610	-34.442	-58.053	-81.958	-7.127

Table S13: Binding energy values obtained by MM-GBSA calculation of the up to five best scoring docking results of the isomers *EE*, *ZZ*, *EZ*, and *ZE* of glycocluster 6βGlc3αMan **1** via Gilde for docking into the closed-gate conformation of FimH (pdb: 1UWF).

Isomer of 6βGlc3αMan 1	Docking Score	ΔG_{Bind}	ΔG_{Bind} Coulomb	ΔG_{Bind} Covalent	ΔG_{Bind} Hbond	ΔG_{Bind} Lipo	ΔG_{Bind} Solv GB	ΔG_{Bind} vdW	Lig Strain Energy
EE	-12.134	-91.17	-58.20	1.21	-8.64	-21.59	43.53	-45.30	16.486
EE	-11.759	-91.28	-58.21	1.21	-8.64	-21.56	43.44	-45.35	16.305
EE	-11.381	-65.89	-57.65	4.48	-8.99	-9.62	37.17	-28.91	46.498
EE	-9.785	-84.95	-50.23	0.94	-8.59	-18.80	36.30	-42.38	25.556
ZZ	-8.698	-85.49	-50.12	6.53	-7.78	-18.67	36.30	-50.41	15.552
ZZ	-8.637	-85.39	-50.01	6.45	-7.79	-18.67	36.33	-50.38	15.551
ZZ	-8.054	-83.66	-48.62	7.89	-8.07	-18.58	35.32	-50.35	25.612
ZZ	-7.379	-72.63	-43.58	7.45	-7.28	-17.07	32.27	-42.98	22.303
ZZ	-7.290	-61.60	-35.95	7.58	-8.36	-12.72	38.74	-48.77	44.824
EZ	-12.728	-92.79	-63.02	5.41	-9.50	-18.26	39.59	-44.37	27.613
EZ	-10.658	-92.17	-63.40	6.57	-9.50	-18.27	39.32	-44.26	28.108
EZ	-10.619	-92.55	-65.56	6.43	-9.50	-18.27	41.09	-44.09	27.815
ZE	-10.651	-74.81	-56.07	12.88	-9.52	-18.00	44.76	-46.93	33.444
ZE	-10.325	-94.37	-70.67	5.96	-9.35	-23.95	52.52	-47.58	15.888
ZE	-9.250	-71.39	-47.00	3.89	-8.61	-14.40	34.54	-36.23	41.543
ZE	-9.197	-64.61	-44.28	11.34	-8.38	-15.98	35.28	-42.44	37.489
ZE	-8.443	-74.91	-49.11	-0.05	-8.47	-11.14	33.45	-36.74	35.909

Table S14: Binding energy values obtained by MM-GBSA calculation of the up to five best scoring docking results of the isomers *EE*, *ZZ*, *EZ*, and *ZE* of glycocluster 6βGlc3αMan **1** via Gilde for docking into the open-gate conformation of FimH (pdb: 1KLF).

Isomer of 6βGlc3αMan 1	Docking Score	ΔG_{Bind}	ΔG_{Bind} Coulomb	ΔG_{Bind} Covalent	ΔG_{Bind} Hbond	ΔG_{Bind} Lipo	ΔG_{Bind} Solv GB	ΔG_{Bind} vdW	Lig Strain Energy
EE	-10.520	-80.30	-57.06	20.74	-7.30	-24.75	31.26	-40.09	24.052
EE	-9.404	-80.17	-52.04	17.00	-7.29	-23.56	27.80	-39.09	22.608
EE	-8.801	-79.25	-47.46	19.65	-6.62	-26.15	32.18	-48.61	21.983
EE	-8.556	-81.48	-60.73	20.68	-6.74	-26.83	39.01	-43.78	21.348
EE	-8.189	-81.04	-50.71	19.63	-6.93	-26.61	33.68	-47.08	25.051
ZZ	-11.787	-71.17	-51.01	20.04	-7.68	-27.28	42.76	-46.51	28.804
ZZ	-11.784	-72.34	-45.83	17.67	-6.78	-28.82	43.85	-50.91	24.703
ZZ	-11.705	-69.89	-45.14	17.40	-8.37	-29.20	47.04	-50.13	29.161
ZZ	-11.503	-59.23	-58.97	17.78	-7.30	-15.65	43.61	-37.23	48.005
ZZ	-11.468	-78.82	-51.02	12.87	-7.38	-26.94	43.27	-47.51	19.859
EZ	-7.452	-58.30	-32.39	3.34	-5.07	-13.70	16.76	-25.18	16.806
EZ	-6.920	-70.75	-47.28	15.07	-5.84	-21.83	30.42	-39.42	13.286
EZ	-6.824	-70.94	-47.07	15.03	-5.85	-21.83	30.02	-39.34	13.002
EZ	-6.613	-67.64	-35.74	14.41	-5.07	-25.67	27.99	-42.05	15.780
EZ	-6.353	-59.16	-30.77	12.41	-5.78	-22.73	31.74	-42.31	13.388
ZE	-10.942	-63.99	-61.76	12.86	-7.20	-12.61	41.34	-34.65	40.187
ZE	-10.703	-69.03	-65.09	13.78	-7.30	-14.78	43.00	-36.10	39.747
ZE	-10.362	-62.55	-56.62	3.15	-7.89	-12.47	46.22	-32.44	38.154

ZE	-8.774	-53.38	-28.32	12.48	-7.66	-23.69	40.44	-45.18	19.937
ZE	-8.168	-63.12	-46.34	9.33	-7.01	-20.57	44.05	-40.64	20.273

Table S15: Binding energy values obtained by MM-GBSA calculation of the up to five best scoring docking results of the isomers *EE*, *ZZ*, *EZ*, and *ZE* of glycocluster 6αMan3αMan **2** via Gilde for docking into the closed-gate conformation of FimH (pdb: 1UWF).

Isomer of 6αMan3αMan 2	Docking Score	ΔG_{Bind}	ΔG_{Bind} Coulomb	ΔG_{Bind} Covalent	ΔG_{Bind} Hbond	ΔG_{Bind} Lipo	ΔG_{Bind} Solv GB	ΔG_{Bind} vdW	Lig Strain Energy
EE	-10.154	-86.42	-61.17	10.16	-8.37	-23.00	46.97	-48.82	18.733
EE	-9.904	-90.00	-56.22	8.45	-8.44	-24.04	43.78	-51.52	18.342
EE	-8.143	-86.54	-48.08	6.19	-7.74	-23.16	43.31	-53.43	12.121
EE	-8.102	-93.05	-57.20	4.70	-8.19	-23.72	44.30	-49.40	8.989
EE	-7.813	-85.17	-47.46	5.08	-7.78	-23.05	42.27	-50.71	12.481
ZZ	-10.528	-76.17	-70.18	8.42	-10.14	-10.09	43.54	-36.06	43.220
ZZ	-10.418	-93.76	-85.12	18.11	-10.14	-18.98	52.31	-48.28	25.597
ZZ	-9.934	-69.90	-55.54	5.81	-10.14	-10.87	40.93	-38.43	49.469
EZ	-11.273	-68.14	-46.21	-1.53	-7.89	-12.77	31.47	-28.69	35.072
EZ	-10.939	-83.87	-43.55	10.05	-8.02	-22.58	28.28	-45.33	26.383
EZ	-10.782	-102.04	-57.94	4.98	-8.02	-25.88	34.03	-46.50	8.249
EZ	-10.293	-70.31	-48.18	-2.07	-7.79	-12.81	30.77	-27.56	32.477
EZ	-9.748	-84.15	-44.74	2.12	-7.65	-19.46	35.62	-49.73	20.990
ZE	-10.181	-75.82	-39.23	2.18	-8.31	-21.91	42.07	-48.91	26.741
ZE	-9.873	-85.46	-76.86	10.91	-9.88	-18.39	60.16	-51.35	18.708
ZE	-9.360	-85.35	-78.53	12.17	-9.87	-18.04	60.05	-50.90	19.950
ZE	-8.525	-79.64	-54.07	5.75	-9.06	-18.44	46.31	-48.07	13.082
ZE	-8.382	-75.61	-52.26	5.03	-9.01	-18.10	49.21	-48.30	17.601

Table S16: Binding energy values obtained by MM-GBSA calculation of the up to five best scoring docking results of the isomers *EE*, *ZZ*, *EZ*, and *ZE* of glycocluster 6αMan3αMan **2** via Gilde for docking into the open-gate conformation of FimH (pdb: 1KLF).

Isomer of 6αMan3αMan 2	Docking Score	ΔG_{Bind}	ΔG_{Bind} Coulomb	ΔG_{Bind} Covalent	ΔG_{Bind} Hbond	ΔG_{Bind} Lipo	ΔG_{Bind} Solv GB	ΔG_{Bind} vdW	Lig Strain Energy
EE	-8.726	-59.07	-51.25	5.32	-6.28	-8.78	41.26	-39.13	25.864
EE	-8.389	-60.12	-44.46	2.60	-6.24	-10.10	32.17	-34.10	27.881
EE	-8.289	-60.92	-53.83	6.02	-6.28	-8.85	41.47	-39.23	24.247
ZZ	-10.035	-65.70	-48.06	15.30	-7.17	-20.04	39.42	-43.67	35.642
ZZ	-9.753	-80.27	-57.25	14.19	-7.89	-25.03	41.65	-43.98	20.485
ZZ	-9.510	-72.07	-52.67	15.71	-7.23	-22.98	40.77	-44.26	24.037
ZZ	-9.267	-78.30	-54.81	14.36	-7.61	-23.13	41.13	-45.97	23.478
ZZ	-9.166	-81.22	-58.34	14.15	-7.92	-24.47	41.17	-43.83	18.919
EZ	-11.233	-80.04	-48.75	2.96	-5.68	-20.99	26.98	-34.55	14.190
EZ	-11.087	-77.01	-44.18	4.19	-5.68	-21.36	24.63	-34.61	17.715
EZ	-11.002	-77.01	-43.17	4.53	-5.68	-21.28	23.57	-34.98	17.770
EZ	-10.995	-76.91	-44.02	4.55	-5.68	-21.34	24.37	-34.77	17.784
EZ	-10.984	-77.25	-43.76	2.95	-5.67	-20.91	24.95	-34.80	16.839

ZE	-9.862	-80.29	-65.53	13.34	-8.77	-22.37	45.47	-39.74	14.875
ZE	-9.778	-68.78	-59.18	14.59	-8.01	-21.60	46.39	-38.63	20.962
ZE	-9.678	-65.49	-57.43	19.27	-8.52	-23.20	46.45	-39.68	24.510
ZE	-9.558	-66.23	-53.45	14.17	-7.99	-21.62	43.35	-38.36	23.107
ZE	-9.288	-72.65	-53.31	20.59	-7.40	-24.46	44.14	-49.70	31.763

Table S17: Binding energy values obtained by MM-GBSA calculation of the up to five best scoring docking results of antennas 6βGlc **3**, 6αMan **4**, and 3αMan **5** as their *E* and *Z* isomers, respectively, via Gilde for docking into the closed-gate conformation of FimH (pdb: 1UWF).

**Antennas**	**Docking Score**	**ΔG_{Bind}**	**ΔG_{Bind} Coulomb**	**ΔG_{Bind} Covalent**	**ΔG_{Bind} Hbond**	**ΔG_{Bind} Lipo**	**ΔG_{Bind} Solv GB**	**ΔG_{Bind} vdW**	**Lig Strain Energy**
E-6βGlc **3**	-8.926	-67.86	-38.71	9.74	-6.10	-20.60	31.29	-41.18	12.472
E-6βGlc **3**	-8.797	-71.75	-36.81	8.32	-6.40	-20.63	27.84	-41.75	9.348
E-6βGlc **3**	-8.779	-70.65	-37.10	6.91	-6.06	-21.38	30.92	-42.10	9.554
E-6βGlc **3**	-8.653	-64.24	-26.45	6.74	-5.84	-21.07	24.99	-40.84	14.473
E-6βGlc **3**	-8.638	-63.78	-29.18	7.86	-5.85	-20.15	26.15	-40.36	14.141
Z-6βGlc **3**	-9.367	-44.37	-37.30	6.58	-6.37	-10.44	25.00	-21.70	26.128
Z-6βGlc **3**	-9.288	-35.69	-21.26	6.09	-6.20	-10.25	26.08	-29.95	30.694
Z-6βGlc **3**	-9.091	-56.08	-36.03	9.87	-6.70	-15.05	23.15	-30.88	21.599
Z-6βGlc **3**	-8.949	-46.42	-30.91	8.25	-6.23	-13.92	25.61	-29.07	19.121
Z-6βGlc **3**	-8.865	-47.33	-36.11	10.21	-6.58	-13.74	27.43	-28.32	20.715
E-6αMan **4**	-10.222	-70.01	-30.98	4.05	-6.28	-19.53	25.14	-40.68	7.492
E-6αMan **4**	-9.949	-69.72	-31.01	3.97	-6.28	-19.50	25.42	-40.60	7.842
E-6αMan **4**	-9.929	-68.44	-30.88	4.05	-6.29	-18.95	24.79	-39.49	8.013
E-6αMan **4**	-9.917	-64.85	-25.21	2.93	-6.43	-17.84	20.43	-37.42	10.955
E-6αMan **4**	-9.904	-70.08	-29.70	2.53	-6.29	-19.01	23.65	-39.58	6.425
Z-6αMan **4**	-9.340	-60.96	-40.01	5.76	-6.16	-13.48	22.12	-28.45	20.548
Z-6αMan **4**	-8.807	-59.67	-52.70	4.32	-7.73	-7.01	28.10	-24.65	16.968
Z-6αMan **4**	-8.711	-55.51	-33.04	1.47	-6.87	-11.25	24.48	-30.13	17.746
Z-6αMan **4**	-7.918	-41.40	-16.40	2.50	-5.83	-9.69	19.18	-30.90	27.173
Z-6αMan **4**	-7.882	-60.82	-36.61	-0.56	-8.25	-9.21	20.16	-26.35	16.866
E-3αMan **5**	-9.980	-71.08	-40.28	4.70	-5.53	-19.42	25.01	-33.09	5.393
E-3αMan **5**	-9.915	-70.91	-40.53	4.75	-5.52	-19.41	25.45	-33.16	5.366
E-3αMan **5**	-9.370	-72.94	-36.99	5.03	-5.70	-20.74	25.24	-37.26	4.546
E-3αMan **5**	-9.332	-73.04	-40.60	5.38	-5.56	-20.36	25.88	-35.05	5.770
E-3αMan **5**	-9.307	-73.11	-40.64	5.41	-5.56	-20.36	25.81	-35.06	5.811
Z-3αMan **5**	-9.650	-59.43	-52.42	10.50	-8.48	-9.80	30.79	-29.18	25.640
Z-3αMan **5**	-9.601	-73.06	-42.90	8.42	-6.05	-19.95	29.46	-39.96	8.250
Z-3αMan **5**	-9.519	-69.14	-34.94	7.74	-6.04	-20.03	26.36	-40.14	12.265
Z-3αMan **5**	-9.491	-65.66	-56.27	17.14	-8.48	-13.98	30.75	-33.98	19.270
Z-3αMan **5**	-9.340	-66.13	-48.65	7.38	-8.33	-11.77	31.31	-34.15	21.308

Table S18: Binding energy values obtained by MM-GBSA calculation of the up to five best scoring docking results of antennas 6βGlc **3**, 6αMan **4**, and 3αMan **5** as their *E* and *Z* isomers, respectively, via Gilde for docking into the open-gate conformation of FimH (pdb: 1KLF).

Antennas	Docking Score	ΔG_{Bind}	ΔG_{Bind} Coulomb	ΔG_{Bind} Covalent	ΔG_{Bind} Hbond	ΔG_{Bind} Lipo	ΔG_{Bind} Solv GB	ΔG_{Bind} vdW	Lig Strain Energy
E-6βGlc **3**	-8.552	-50.06	-43.13	13.11	-5.41	-17.95	32.23	-28.15	20.116
E-6βGlc **3**	-8.499	-60.73	-51.93	12.16	-6.45	-18.11	32.58	-28.25	18.429
E-6βGlc **3**	-7.926	-42.10	-33.00	16.05	-6.10	-16.04	26.79	-29.79	24.395
E-6βGlc **3**	-7.808	-48.84	-32.94	10.86	-5.96	-17.55	25.12	-27.67	21.617
E-6βGlc **3**	-7.772	-55.30	-39.39	7.98	-6.10	-16.93	29.37	-28.86	15.645
Z-6βGlc **3**	-6.489	-38.17	-27.68	5.49	-2.64	-16.36	25.70	-20.83	14.487
Z-6βGlc **3**	-6.020	-38.19	-27.81	5.49	-2.64	-16.35	25.73	-20.76	14.474
Z-6βGlc **3**	-5.803	-39.26	-30.92	8.84	-2.64	-17.70	27.56	-22.54	13.375
Z-6βGlc **3**	-5.610	-38.12	-27.65	5.48	-2.64	-16.36	25.74	-20.85	14.539
E-6αMan **4**	-10.440	-65.53	-45.95	7.09	-5.55	-18.74	33.34	-35.06	10.844
E-6αMan **4**	-10.117	-67.21	-52.37	8.04	-5.55	-18.73	37.19	-35.14	9.519
E-6αMan **4**	-10.114	-65.95	-48.00	8.11	-5.55	-18.77	33.95	-35.04	10.665
E-6αMan **4**	-10.108	-50.09	-43.17	11.00	-5.01	-16.06	29.22	-26.07	17.920
E-6αMan **4**	-9.977	-61.17	-46.25	9.89	-5.56	-17.96	32.12	-32.86	15.136
Z-6αMan **4**	-10.190	-64.64	-35.70	7.33	-6.24	-18.55	17.47	-28.77	24.171
Z-6αMan **4**	-10.175	-54.46	-32.08	7.99	-5.22	-17.63	19.26	-26.59	23.991
Z-6αMan **4**	-10.164	-65.96	-43.98	8.93	-6.24	-16.84	18.36	-25.98	22.913
Z-6αMan **4**	-9.758	-64.80	-35.68	7.31	-6.24	-18.54	17.34	-28.78	24.064
Z-6αMan **4**	-9.528	-71.11	-44.81	8.53	-6.14	-20.66	21.09	-28.87	15.370
E-3αMan **5**	-8.195	-59.19	-31.44	11.71	-5.10	-21.82	21.38	-31.40	14.480
E-3αMan **5**	-8.190	-55.08	-34.38	5.36	-5.07	-14.37	19.45	-24.90	9.095
E-3αMan **5**	-8.155	-62.79	-38.89	6.70	-5.08	-17.56	20.71	-26.57	5.353
E-3αMan **5**	-7.922	-57.54	-32.11	14.67	-5.10	-20.62	21.23	-33.38	15.531
E-3αMan **5**	-7.719	-61.90	-34.12	19.31	-5.68	-24.33	22.27	-36.79	21.022
Z-3αMan **5**	-10.375	-62.18	-38.51	2.06	-5.50	-16.20	22.75	-25.37	16.322
Z-3αMan **5**	-10.029	-65.96	-45.25	8.86	-5.50	-19.56	25.84	-28.93	12.611
Z-3αMan **5**	-10.026	-61.49	-40.04	2.32	-5.49	-16.38	25.01	-25.47	15.720
Z-3αMan **5**	-9.965	-61.48	-40.05	8.13	-5.19	-18.15	26.53	-30.60	12.305
Z-3αMan **5**	-9.869	-64.36	-41.17	2.33	-5.50	-16.14	22.30	-24.76	14.197

Table S19: Scoring values for docking of the isomers *EE*, *ZZ*, *EZ*, and *ZE* of glycocluster 6βGlc3αMan **1** into the closed gate binding pocket of FimH (pdb: 1UWF) using IFD.

Isomer of 6βGlc3αMan 1	IFD Score	Docking Score	Glide evdw	Glide ecoul	Glide energy	Glide emodel	XP HBond
EE	-342.16	-10.252	-34.738	-35.697	-70.435	-112.873	-7.411
EE	-338.49	-6.624	-38.566	-14.027	-52.594	-82.523	-5.157
EE	-337.48	-6.531	-37.707	-22.736	-60.444	-76.215	-4.995
EE	-337.32	-5.767	-39.290	-15.049	-54.339	-74.250	-5.301
EE	-336.56	-4.991	-46.359	-11.998	-58.358	-71.280	-2.887
EE	-336.44	-4.637	-42.148	-13.634	-55.782	-75.656	-4.141
ZZ	-343.48	-12.842	-33.175	-55.661	-88.836	-157.461	-10.578
ZZ	-343.26	-12.498	-43.121	-45.843	-88.964	-141.309	-9.582
ZZ	-342.57	-12.006	-35.469	-44.692	-80.161	-139.014	-10.063
ZZ	-342.28	-11.179	-42.876	-49.640	-92.516	-151.770	-8.843
ZZ	-341.86	-12.018	-35.492	-47.267	-82.759	-120.051	-9.958
ZZ	-341.77	-11.970	-38.301	-51.256	-89.558	-138.366	-9.161
ZZ	-341.72	-11.705	-36.709	-37.904	-74.614	-122.268	-8.284
ZZ	-341.23	-11.195	-40.705	-39.700	-80.405	-116.871	-7.747
ZZ	-341.09	-11.071	-35.949	-45.687	-81.636	-124.901	-8.349
ZZ	-341.02	-10.771	-45.369	-27.596	-72.965	-109.983	-6.839
ZZ	-340.69	-10.656	-40.528	-38.996	-79.524	-119.670	-8.050
ZZ	-340.48	-10.683	-44.313	-31.299	-75.612	-110.837	-6.676
ZZ	-339.39	-9.667	-35.288	-36.226	-71.514	-112.789	-6.207
EZ	-344.06	-11.623	-40.882	-42.198	-83.081	-132.780	-8.096
EZ	-343.55	-11.726	-29.127	-30.525	-59.653	-116.921	-8.225
EZ	-343.31	-12.006	-36.820	-41.628	-78.448	-119.823	-8.432
EZ	-343.27	-11.162	-38.182	-43.524	-81.707	-121.978	-7.696
EZ	-343.25	-10.696	-34.135	-39.986	-74.121	-115.339	-7.206
EZ	-343.20	-11.660	-28.304	-46.901	-75.205	-121.943	-7.963
EZ	-343.08	-10.992	-30.077	-39.048	-69.125	-115.523	-7.098
EZ	-343.01	-11.050	-32.481	-36.723	-69.204	-123.674	-7.404
EZ	-342.99	-11.565	-34.907	-41.163	-76.070	-113.513	-8.130
EZ	-342.73	-11.366	-28.564	-37.640	-66.204	-115.724	-8.095
EZ	-342.72	-11.434	-31.198	-36.639	-67.836	-102.069	-7.416
EZ	-342.72	-10.797	-32.902	-37.208	-70.110	-111.563	-7.628
EZ	-342.68	-10.975	-29.255	-35.629	-64.884	-114.540	-7.139
EZ	-341.68	-10.308	-32.717	-37.468	-70.185	-118.242	-6.863
ZE	-342.95	-11.884	-39.552	-43.757	-83.309	-137.325	-8.873
ZE	-342.90	-12.683	-37.713	-41.757	-79.470	-117.403	-9.338
ZE	-342.43	-11.890	-40.173	-41.624	-81.796	-139.088	-8.546
ZE	-342.04	-11.612	-36.921	-42.380	-79.301	-124.214	-8.329
ZE	-341.97	-11.395	-40.951	-42.226	-83.177	-141.009	-8.163
ZE	-341.92	-11.419	-38.410	-41.309	-79.718	-133.330	-7.890
ZE	-341.90	-11.736	-41.669	-39.395	-81.064	-129.940	-8.781
ZE	-341.77	-11.749	-35.240	-43.170	-78.410	-131.059	-8.419
ZE	-341.33	-10.453	-38.848	-39.780	-78.628	-119.034	-7.274
ZE	-337.60	-7.741	-35.742	-21.694	-57.437	-83.352	-4.891

ZE	-336.93	-7.134	-36.568	-22.464	-59.032	-76.636	-4.469

Table S20: Scoring values for docking of the isomers *EE*, *ZZ*, *EZ*, and *ZE* of glycocluster 6αMan3αMan **2** into the closed gate binding pocket of FimH (pdb: 1UWF) using IFD.

**Isomer of 6αMan3αMan 2**	**IFD Score**	**Docking Score**	**Glide evdw**	**Glide ecoul**	**Glide energy**	**Glide emodel**	**XP HBond**
EE	-344.82	-12.635	-43.202	-38.349	-81.550	-122.765	-8.884
EE	-344.16	-12.562	-30.477	-40.330	-70.807	-116.629	-8.484
EE	-343.87	-12.020	-40.252	-36.365	-76.617	-117.463	-8.017
EE	-343.66	-11.785	-34.567	-37.524	-72.091	-122.547	-7.995
EE	-343.58	-12.086	-34.343	-39.028	-73.371	-121.396	-8.628
EE	-343.36	-11.123	-37.805	-39.327	-77.133	-117.261	-7.049
EE	-343.23	-11.759	-33.332	-38.913	-72.246	-121.703	-7.799
EE	-343.15	-11.413	-28.183	-37.754	-65.936	-111.642	-7.482
EE	-342.41	-10.530	-26.527	-37.886	-64.413	-116.320	-7.062
ZZ	-344.40	-13.665	-39.271	-50.864	-90.135	-147.886	-10.876
ZZ	-344.38	-13.831	-39.138	-49.519	-88.657	-146.832	-10.904
ZZ	-343.83	-13.437	-40.616	-51.402	-92.018	-157.674	-11.112
ZZ	-343.76	-13.316	-39.154	-49.060	-88.214	-144.099	-10.833
ZZ	-343.63	-13.263	-41.211	-47.487	-88.698	-141.586	-10.166
ZZ	-343.58	-13.611	-44.699	-42.608	-87.307	-140.943	-10.271
ZZ	-342.63	-13.100	-45.922	-37.922	-83.845	-128.423	-8.458
ZZ	-341.75	-12.151	-34.881	-41.909	-76.790	-122.330	-8.256
ZZ	-341.75	-10.757	-35.919	-39.553	-75.472	-119.803	-8.812
ZZ	-341.54	-11.907	-33.107	-41.914	-75.021	-117.826	-7.804
ZZ	-339.12	-9.505	-36.570	-25.399	-61.969	-85.875	-7.308
ZZ	-338.11	-7.864	-43.608	-28.248	-71.856	-97.022	-4.800
EZ	-346.23	-14.225	-43.230	-42.960	-86.190	-130.458	-8.973
EZ	-344.98	-12.893	-38.982	-43.151	-82.133	-115.697	-8.443
EZ	-344.70	-12.452	-40.070	-40.603	-80.673	-131.794	-8.242
EZ	-344.66	-12.692	-40.718	-36.855	-77.573	-123.668	-8.381
EZ	-344.32	-12.195	-36.053	-40.144	-76.196	-124.404	-8.237
EZ	-343.98	-12.146	-37.086	-37.718	-74.804	-117.273	-8.079
EZ	-343.94	-12.431	-42.446	-37.381	-79.827	-122.529	-7.638
EZ	-343.60	-12.407	-40.797	-40.089	-80.886	-130.760	-8.600
EZ	-343.22	-11.747	-40.191	-33.382	-73.573	-107.019	-7.745
EZ	-342.91	-10.958	-34.612	-34.424	-69.037	-108.455	-7.955
EZ	-342.82	-11.804	-38.180	-42.015	-80.195	-118.556	-7.925
EZ	-342.52	-11.084	-34.921	-37.506	-72.428	-116.354	-7.706
EZ	-342.39	-10.580	-38.443	-41.497	-79.940	-123.584	-7.200
EZ	-341.67	-10.447	-46.088	-38.200	-84.288	-121.528	-6.668
ZE	-345.17	-13.771	-34.601	-37.461	-72.062	-109.015	-9.647
ZE	-345.07	-13.358	-36.420	-38.793	-75.213	-110.417	-8.640
ZE	-345.03	-12.708	-34.269	-36.353	-70.622	-120.704	-9.016
ZE	-344.83	-12.873	-42.065	-41.773	-83.838	-117.138	-8.634
ZE	-344.81	-12.758	-37.115	-43.574	-80.688	-119.904	-9.204

ZE	-344.43	-12.765	-41.315	-36.181	-77.496	-123.403	-8.118
ZE	-343.78	-12.410	-35.081	-38.315	-73.395	-107.764	-8.115
ZE	-343.12	-12.122	-32.283	-45.101	-77.383	-122.823	-9.387
ZE	-342.74	-11.446	-34.222	-45.278	-79.500	-128.324	-8.785
ZE	-342.66	-11.161	-35.211	-38.775	-73.986	-115.688	-7.115
ZE	-342.27	-11.404	-32.852	-38.442	-71.294	-123.961	-7.900
ZE	-338.16	-7.061	-30.493	-20.301	-50.795	-69.696	-4.948

Table S21: Scoring values for docking of the antennas 6βGlc **3**, 6αMan **4**, and 3αMan **5** as their *E* and *Z* isomer, respectively, into the closed gate binding pocket of FimH (pdb: 1UWF) using IFD.

Antennas	IFD Score	Docking Score	Glide evdw	Glide ecoul	Glide energy	Glide emodel	XP HBond
E-6βGlc **3**	-342.36	-12.133	-31.531	-30.978	-62.508	-96.064	-7.604
E-6βGlc **3**	-342.23	-12.420	-31.601	-31.467	-63.068	-95.858	-7.848
E-6βGlc **3**	-342.00	-11.724	-31.532	-30.620	-62.152	-93.685	-7.387
E-6βGlc **3**	-341.76	-11.841	-23.611	-33.383	-56.994	-86.479	-7.340
E-6βGlc **3**	-340.95	-11.081	-31.863	-31.169	-63.032	-98.523	-6.844
E-6βGlc **3**	-340.61	-11.234	-34.161	-25.482	-59.643	-89.537	-6.925
E-6βGlc **3**	-340.29	-10.790	-31.987	-27.286	-59.273	-85.294	-6.367
E-6βGlc **3**	-340.26	-10.925	-26.711	-33.504	-60.216	-85.855	-6.720
E-6βGlc **3**	-340.09	-10.703	-27.940	-29.951	-57.891	-81.849	-7.017
E-6βGlc **3**	-340.06	-10.171	-31.527	-28.399	-59.925	-86.487	-5.989
E-6βGlc **3**	-339.87	-9.931	-26.595	-29.775	-56.370	-82.444	-5.748
E-6βGlc **3**	-339.85	-10.534	-32.382	-28.193	-60.575	-95.600	-6.002
E-6βGlc **3**	-339.58	-9.742	-32.148	-30.503	-62.651	-99.817	-5.552
E-6βGlc **3**	-339.41	-9.987	-31.001	-22.868	-53.869	-79.059	-5.875
E-6βGlc **3**	-339.29	-9.913	-27.635	-21.044	-48.678	-66.811	-5.552
Z-6βGlc **3**	-340.89	-10.943	-27.000	-33.321	-60.321	-87.812	-6.686
Z-6βGlc **3**	-340.78	-10.896	-23.690	-29.980	-53.670	-77.562	-7.209
Z-6βGlc **3**	-340.67	-10.914	-29.964	-26.291	-56.255	-87.723	-6.783
Z-6βGlc **3**	-340.45	-11.006	-29.215	-26.927	-56.142	-81.035	-7.222
Z-6βGlc **3**	-340.32	-9.953	-28.096	-25.877	-53.973	-77.542	-6.883
Z-6βGlc **3**	-340.11	-10.755	-25.206	-35.899	-61.104	-93.003	-6.735
Z-6βGlc **3**	-339.79	-10.086	-28.267	-30.261	-58.528	-86.250	-6.911
Z-6βGlc **3**	-339.74	-10.225	-25.128	-34.432	-59.561	-89.109	-7.235
Z-6βGlc **3**	-339.50	-10.627	-24.515	-37.992	-62.507	-96.397	-7.230
Z-6βGlc **3**	-339.43	-10.367	-22.815	-37.680	-60.495	-95.116	-7.151
Z-6βGlc **3**	-339.36	-10.350	-24.798	-30.384	-55.182	-83.722	-6.911
Z-6βGlc **3**	-338.58	-9.033	-25.295	-31.965	-57.259	-90.767	-5.649
Z-6βGlc **3**	-338.58	-9.485	-28.887	-24.440	-53.326	-81.932	-5.847
Z-6βGlc **3**	-338.55	-9.009	-28.268	-26.347	-54.615	-80.609	-5.455
E-6αMan **4**	-342.21	-12.790	-31.074	-32.128	-63.202	-95.102	-7.673
E-6αMan **4**	-340.97	-11.258	-30.573	-32.431	-63.004	-99.138	-6.837
E-6αMan **4**	-340.96	-11.199	-30.549	-27.805	-58.354	-82.866	-7.056
E-6αMan **4**	-340.92	-11.468	-30.203	-34.309	-64.512	-92.838	-6.904
E-6αMan **4**	-340.76	-11.266	-32.316	-34.598	-66.914	-95.910	-6.466
E-6αMan **4**	-340.67	-10.621	-31.387	-35.891	-67.277	-97.639	-6.426

E-6αMan **4**	-340.52	-10.896	-29.565	-28.871	-58.436	-84.410	-6.733
E-6αMan **4**	-340.50	-10.892	-30.344	-30.448	-60.791	-87.036	-6.444
E-6αMan **4**	-340.44	-11.079	-32.044	-32.652	-64.696	-92.171	-6.458
E-6αMan **4**	-340.36	-10.498	-31.794	-32.989	-64.783	-95.278	-5.923
E-6αMan **4**	-340.31	-11.062	-33.802	-30.477	-64.280	-91.220	-6.119
E-6αMan **4**	-340.29	-10.942	-27.675	-34.394	-62.069	-91.466	-6.460
E-6αMan **4**	-340.06	-10.957	-23.983	-27.815	-51.798	-81.731	-6.586
E-6αMan **4**	-340.04	-10.962	-31.568	-29.787	-61.355	-90.640	-6.224
E-6αMan **4**	-339.91	-10.520	-29.625	-30.163	-59.788	-84.532	-6.077
E-6αMan **4**	-339.81	-10.548	-29.807	-27.307	-57.114	-77.252	-5.985
E-6αMan **4**	-339.34	-10.779	-33.208	-33.976	-67.184	-87.932	-6.441
E-6αMan **4**	-337.21	-8.572	-21.766	-27.489	-49.254	-76.342	-5.456
Z-6αMan **4**	-342.29	-12.792	-20.685	-37.868	-58.553	-100.509	-9.730
Z-6αMan **4**	-341.34	-11.752	-20.747	-35.692	-56.438	-94.111	-8.104
Z-6αMan **4**	-341.07	-11.481	-22.820	-38.711	-61.530	-89.247	-7.710
Z-6αMan **4**	-340.92	-11.444	-26.784	-30.863	-57.647	-83.638	-7.260
Z-6αMan **4**	-340.45	-11.414	-24.082	-35.854	-59.936	-87.858	-7.200
Z-6αMan **4**	-340.30	-11.237	-27.833	-34.971	-62.804	-91.309	-7.356
Z-6αMan **4**	-340.09	-11.327	-23.232	-36.137	-59.368	-91.738	-8.247
Z-6αMan **4**	-339.59	-10.701	-27.105	-34.996	-62.101	-92.128	-7.223
Z-6αMan **4**	-339.29	-10.226	-23.862	-27.784	-51.646	-79.226	-5.843
Z-6αMan **4**	-339.23	-10.388	-24.796	-37.766	-62.563	-89.357	-6.967
Z-6αMan **4**	-339.06	-10.376	-28.709	-27.440	-56.150	-84.439	-6.133
Z-6αMan **4**	-338.74	-9.644	-21.153	-43.880	-65.032	-103.051	-6.619
Z-6αMan **4**	-338.57	-9.676	-30.846	-30.601	-61.448	-94.522	-5.517
Z-6αMan **4**	-338.53	-8.985	-25.819	-22.526	-48.345	-64.413	-6.240
Z-6αMan **4**	-337.97	-8.999	-28.656	-28.582	-57.238	-77.268	-5.464
Z-6αMan **4**	-337.80	-8.961	-27.517	-26.428	-53.944	-76.417	-5.059
Z-6αMan **4**	-337.46	-8.820	-21.753	-25.141	-46.894	-62.404	-5.280
Z-6αMan **4**	-336.70	-8.371	-23.569	-24.021	-47.590	-74.212	-5.058
E-3αMan **5**	-340.69	-12.288	-29.689	-33.339	-63.028	-78.287	-7.834
E-3αMan **5**	-340.44	-12.471	-27.965	-31.741	-59.705	-77.559	-8.002
E-3αMan **5**	-340.41	-12.293	-28.187	-32.393	-60.580	-79.695	-7.817
E-3αMan **5**	-340.33	-12.292	-25.516	-32.585	-58.101	-80.111	-8.000
E-3αMan **5**	-340.30	-12.314	-28.816	-32.578	-61.394	-72.613	-7.561
E-3αMan **5**	-339.87	-11.871	-26.215	-32.767	-58.982	-77.950	-7.435
E-3αMan **5**	-339.29	-11.410	-25.399	-35.131	-60.531	-75.111	-6.797
E-3αMan **5**	-339.24	-11.398	-26.886	-28.222	-55.109	-71.793	-7.017
E-3αMan **5**	-339.24	-11.290	-27.006	-28.563	-55.569	-72.660	-6.555
E-3αMan **5**	-339.18	-11.126	-26.041	-30.970	-57.011	-75.200	-6.552
E-3αMan **5**	-339.11	-11.195	-25.101	-33.769	-58.871	-72.111	-7.092
E-3αMan **5**	-338.95	-11.101	-28.994	-30.519	-59.513	-73.495	-6.448
E-3αMan **5**	-338.94	-11.260	-28.157	-29.997	-58.153	-78.545	-6.549
E-3αMan **5**	-338.87	-11.007	-25.018	-31.469	-56.487	-74.930	-6.653
E-3αMan **5**	-338.61	-11.246	-27.513	-31.022	-58.534	-75.708	-6.715
E-3αMan **5**	-338.54	-10.705	-26.283	-32.054	-58.337	-74.865	-6.483
E-3αMan **5**	-338.43	-10.584	-25.208	-32.659	-57.867	-77.535	-6.467

E-3αMan **5**	-338.10	-10.714	-26.100	-32.101	-58.200	-74.546	-6.272
E-3αMan **5**	-338.06	-10.692	-26.529	-31.592	-58.120	-74.399	-6.419
Z-3αMan **5**	-339.36	-12.297	-26.564	-35.862	-62.426	-101.533	-8.445
Z-3αMan **5**	-339.06	-12.334	-20.513	-33.526	-54.039	-84.252	-8.371
Z-3αMan **5**	-338.90	-11.241	-28.716	-29.808	-58.524	-78.637	-7.016
Z-3αMan **5**	-338.82	-11.602	-24.685	-37.234	-61.919	-86.218	-8.136
Z-3αMan **5**	-338.74	-11.830	-24.454	-32.883	-57.337	-84.960	-7.533
Z-3αMan **5**	-338.52	-11.297	-18.307	-36.892	-55.199	-79.468	-7.310
Z-3αMan **5**	-338.49	-11.808	-16.452	-40.869	-57.321	-96.435	-8.216
Z-3αMan **5**	-338.44	-10.788	-19.594	-32.124	-51.717	-83.953	-7.012
Z-3αMan **5**	-338.32	-11.059	-25.125	-33.401	-58.526	-81.923	-7.186
Z-3αMan **5**	-338.26	-11.606	-22.809	-42.642	-65.451	-92.374	-7.817
Z-3αMan **5**	-337.93	-10.819	-26.325	-28.455	-54.779	-75.489	-6.702
Z-3αMan **5**	-337.81	-10.899	-27.082	-32.124	-59.207	-92.491	-7.231
Z-3αMan **5**	-337.65	-11.299	-29.058	-33.096	-62.154	-96.024	-6.866
Z-3αMan **5**	-337.49	-10.584	-22.920	-36.531	-59.450	-86.613	-6.667
Z-3αMan **5**	-337.33	-10.169	-22.763	-32.329	-55.092	-89.455	-6.252
Z-3αMan **5**	-336.55	-9.625	-23.858	-21.510	-45.367	-67.383	-6.180
Z-3αMan **5**	-336.33	-10.052	-27.133	-25.618	-52.751	-56.406	-6.453
Z-3αMan **5**	-334.87	-8.411	-23.960	-21.613	-45.573	-68.795	-5.155

Table S22: Scoring values of binding pose metadynamic calculation of the isomers *EE*, *ZZ*, *EZ*, and *ZE* of glycocluster 6βGlc3αMan **1** for the top five scoring binding poses from IFD. Lower composite (Comp.) scores correlate to the stability of the protein–ligand complexes.

Isomer of 6βGlc3αMan 1	IFD Score	Comp. Score	Pose Score	Persis-tence	Persis-tence Length	Persis-tence Sum	HBond Persis-tence	HBond Persis-tence Length	HBond Persis-tence Sum
EE	-342.16	2.477	3.386	0.182	11.000	2.000	0.182	11.000	2.000
EE	-338.49	2.780	4.223	0.289	4.000	1.155	0.289	4.000	1.155
EE	-336.56	6.451	6.963	0.102	4.000	0.409	0.102	4.000	0.409
EE	-337.48	6.684	7.063	0.076	3.000	0.227	0.076	3.000	0.227
EE	-337.32	8.022	8.086	0.013	5.000	0.064	0.013	5.000	0.064
ZZ	-343.48	-1.772	1.754	0.705	14.000	9.873	0.705	14.000	9.873
ZZ	-341.86	2.397	5.281	0.577	13.000	7.500	0.577	13.000	7.500
ZZ	-342.57	2.543	5.903	0.672	11.000	7.391	0.672	11.000	7.391
ZZ	-343.26	3.973	6.923	0.590	11.000	6.491	0.590	11.000	6.491
ZZ	-342.28	4.108	6.809	0.540	14.000	7.564	0.540	14.000	7.564
EZ	-343.31	0.661	3.740	0.616	11.000	6.773	0.616	11.000	6.773
EZ	-343.55	1.364	4.773	0.682	7.000	4.773	0.682	7.000	4.773
EZ	-344.06	2.921	5.863	0.588	11.000	6.473	0.588	11.000	6.473
EZ	-343.25	3.589	7.034	0.689	9.000	6.200	0.689	9.000	6.200
EZ	-343.27	5.560	8.344	0.557	12.000	6.682	0.557	12.000	6.682
ZE	-341.97	-0.086	2.940	0.605	12.000	7.264	0.605	12.000	7.264
ZE	-342.95	0.955	4.409	0.691	11.000	7.600	0.691	11.000	7.600
ZE	-342.43	1.235	4.417	0.636	11.000	7.000	0.636	11.000	7.000
ZE	-342.90	2.116	5.085	0.594	12.000	7.127	0.594	12.000	7.127
ZE	-342.04	2.336	5.977	0.728	9.000	6.555	0.728	9.000	6.555

Table S23: Scoring values of binding pose metadynamic calculation of the isomers *EE*, *ZZ*, *EZ*, and *ZE* of glycocluster 6αMan3αMan **2** for the top five scoring binding poses from IFD. Lower composite (Comp.) scores correlate to the stability of the protein–ligand complexes.

Isomer of 6αMan3αMan 2	IFD Score	Comp. Score	Pose Score	Persis-tence	Persis-tence Length	Persis-tence Sum	HBond Persis-tence	HBond Persis-tence Length	HBond Persis-tence Sum
EE	-343.58	0.681	4.004	0.665	9.000	5.982	0.665	9.000	5.982
EE	-343.66	0.861	3.823	0.592	12.000	7.109	0.592	12.000	7.109
EE	-343.87	2.152	5.129	0.595	12.000	7.145	0.595	12.000	7.145
EE	-344.82	3.352	6.472	0.624	11.000	6.864	0.624	11.000	6.864
EE	-344.16	6.093	9.977	0.777	9.000	6.991	0.777	9.000	6.991
ZZ	-343.63	0.388	2.512	0.425	15.000	6.373	0.425	15.000	6.373
ZZ	-343.83	0.748	3.052	0.461	16.000	7.373	0.461	16.000	7.373
ZZ	-343.76	1.157	3.588	0.486	15.000	7.291	0.486	15.000	7.291
ZZ	-344.40	1.643	4.127	0.497	15.000	7.455	0.497	15.000	7.455
ZZ	-344.38	2.236	4.517	0.456	16.000	7.300	0.456	16.000	7.300
EZ	-344.66	-1.001	2.706	0.741	9.000	6.673	0.741	9.000	6.673
EZ	-344.32	-0.753	3.514	0.853	8.000	6.827	0.853	8.000	6.827
EZ	-346.23	1.722	4.781	0.612	10.000	6.118	0.612	10.000	6.118
EZ	-344.70	2.133	5.756	0.725	10.000	7.245	0.725	10.000	7.245
EZ	-344.98	3.204	6.714	0.702	10.000	7.018	0.702	10.000	7.018
ZE	-345.03	0.432	4.903	0.894	8.000	7.155	0.894	8.000	7.155
ZE	-345.17	1.701	5.139	0.688	11.000	7.564	0.688	11.000	7.564
ZE	-345.07	1.808	4.921	0.623	10.000	6.227	0.623	10.000	6.227
ZE	-344.83	2.492	6.124	0.726	10.000	7.264	0.726	10.000	7.264
ZE	-344.81	2.866	6.325	0.692	11.000	7.609	0.692	11.000	7.609

Table S24: Scoring values of binding pose metadynamic calculation of the antennas 6βGlc **3**, 6αMan **4**, and 3αMan **5** as their *E* and *Z* isomer, respectively, for the top five scoring binding poses from IFD. Lower composite (Comp.) scores correlate to the stability of the protein–ligand complexes.

Antennas	IFD Score	Comp. Score	Pose Score	Persis-tence	Persis-tence Length	Persis-tence Sum	HBond Persis-tence	HBond Persis-tence Length	HBond Persis-tence Sum
E-6βGlc **3**	-342.23	1.120	4.266	0.629	9.000	5.664	0.629	9.000	5.664
E-6βGlc **3**	-341.76	1.162	4.497	0.667	8.000	5.336	0.667	8.000	5.336
E-6βGlc **3**	-340.95	2.277	5.674	0.679	7.000	4.755	0.679	7.000	4.755
E-6βGlc **3**	-342.36	2.321	5.217	0.579	7.000	4.055	0.579	7.000	4.055
E-6βGlc **3**	-342.00	3.037	6.241	0.641	8.000	5.127	0.641	8.000	5.127
Z-6βGlc **3**	-340.67	1.341	4.358	0.603	8.000	4.827	0.603	8.000	4.827
Z-6βGlc **3**	-340.89	1.442	3.761	0.464	9.000	4.173	0.464	9.000	4.173
Z-6βGlc **3**	-340.45	2.053	4.275	0.444	8.000	3.555	0.444	8.000	3.555
Z-6βGlc **3**	-340.78	2.516	5.488	0.594	8.000	4.755	0.594	8.000	4.755
Z-6βGlc **3**	-340.32	3.592	6.722	0.626	7.000	4.382	0.626	7.000	4.382
E-6αMan **4**	-340.76	0.877	3.665	0.558	9.000	5.018	0.558	9.000	5.018
E-6αMan **4**	-342.21	1.240	4.078	0.568	9.000	5.109	0.568	9.000	5.109
E-6αMan **4**	-340.92	1.438	4.257	0.564	9.000	5.073	0.564	9.000	5.073

E-6αMan **4**	-340.96	1.651	4.975	0.665	8.000	5.318	0.665	8.000	5.318
E-6αMan **4**	-340.97	1.909	5.034	0.625	8.000	5.000	0.625	8.000	5.000
Z-6αMan **4**	-340.45	0.108	3.303	0.639	7.000	4.473	0.639	7.000	4.473
Z-6αMan **4**	-341.07	0.190	4.150	0.792	8.000	6.336	0.792	8.000	6.336
Z-6αMan **4**	-341.34	0.500	3.893	0.678	8.000	5.427	0.678	8.000	5.427
Z-6αMan **4**	-342.29	3.514	6.504	0.598	9.000	5.382	0.598	9.000	5.382
Z-6αMan **4**	-340.92	3.785	6.426	0.528	9.000	4.755	0.528	9.000	4.755
E-3αMan **5**	-340.33	0.570	3.810	0.648	7.000	4.536	0.648	7.000	4.536
E-3αMan **5**	-340.30	0.594	3.574	0.596	9.000	5.364	0.596	9.000	5.364
E-3αMan **5**	-340.69	0.793	4.170	0.675	7.000	4.727	0.675	7.000	4.727
E-3αMan **5**	-340.41	0.811	4.158	0.669	8.000	5.355	0.669	8.000	5.355
E-3αMan **5**	-340.44	1.081	4.365	0.657	8.000	5.255	0.657	8.000	5.255
Z-3αMan **5**	-338.74	1.700	4.618	0.584	10.000	5.836	0.584	10.000	5.836
Z-3αMan **5**	-339.36	3.376	6.495	0.624	8.000	4.991	0.624	8.000	4.991
Z-3αMan **5**	-338.90	3.442	7.048	0.721	6.000	4.327	0.721	6.000	4.327
Z-3αMan **5**	-338.82	4.896	7.314	0.484	10.000	4.836	0.484	10.000	4.836
Z-3αMan **5**	-339.06	5.202	7.402	0.440	10.000	4.400	0.440	10.000	4.400

Table S25: Binding energy values obtained by MM-GBSA calculation of the most stable protein–ligand complexes from IFD according to the binding pose metadynamic calculation of the antennas 6βGlc **3**, 6αMan **4**, and 3αMan **5** as their *E* and *Z* isomer, respectively, as well as of the isomers *EE*, *ZZ*, *EZ*, and *ZE* of glycocluster 6βGlc3αMan **1** and 6αMan3αMan **2** into the closed-gate (pdb: 1UWF) conformation of FimH.

**Isomer of 6βGlc3αMan 1**	**IFD Score**	**ΔG_{Bind}**	**ΔG_{Bind} Coulomb**	**ΔG_{Bind} Covalent**	**ΔG_{Bind} Hbond**	**ΔG_{Bind} Lipo**	**ΔG_{Bind} Solv GB**	**ΔG_{Bind} vdW**	**Lig Strain Energy**
EE	-342.16	-69.57	-50.20	3.64	-7.62	-16.78	33.02	-29.03	24.261
ZZ	-343.48	-107.61	-88.69	0.57	-11.32	-19.32	48.71	-36.90	14.105
EZ	-343.31	-88.33	-58.10	10.13	-8.37	-22.75	36.01	-42.96	34.858
ZE	-341.97	-82.98	-47.55	5.43	-9.77	-24.83	38.97	-42.97	39.063
**Isomer of 6αMan3αMan 2**									
EE	-343.58	-89.99	-52.98	11.33	-7.41	-27.77	34.33	-45.33	16.398
ZZ	-343.63	-96.22	-78.23	8.02	-10.54	-19.98	49.47	-44.63	19.556
EZ	-344.66	-69.20	-28.38	-1.70	-7.56	-20.28	27.07	-35.87	29.720
ZE	-345.03	-76.98	-51.49	8.25	-8.45	-21.98	36.72	-37.80	32.223
**Antennas**									
E-6βGlc **3**	-342.23	-75.19	-47.01	4.90	-7.21	-19.66	27.75	-30.82	11.685
Z-6βGlc **3**	-340.67	-35.95	-19.86	10.45	-6.46	-11.07	20.52	-28.04	38.996
E-6αMan **4**	-340.76	-83.07	-41.89	4.11	-5.77	-23.22	22.54	-35.89	15.266
Z-6αMan **4**	-340.45	-64.40	-43.09	4.04	-5.60	-18.52	28.25	-28.26	11.618
E-3αMan **5**	-340.33	-71.41	-40.12	6.76	-5.79	-20.74	21.12	-30.42	14.741
Z-3αMan **5**	-338.74	-67.74	-40.86	6.23	-6.62	-17.32	20.34	-27.56	19.047

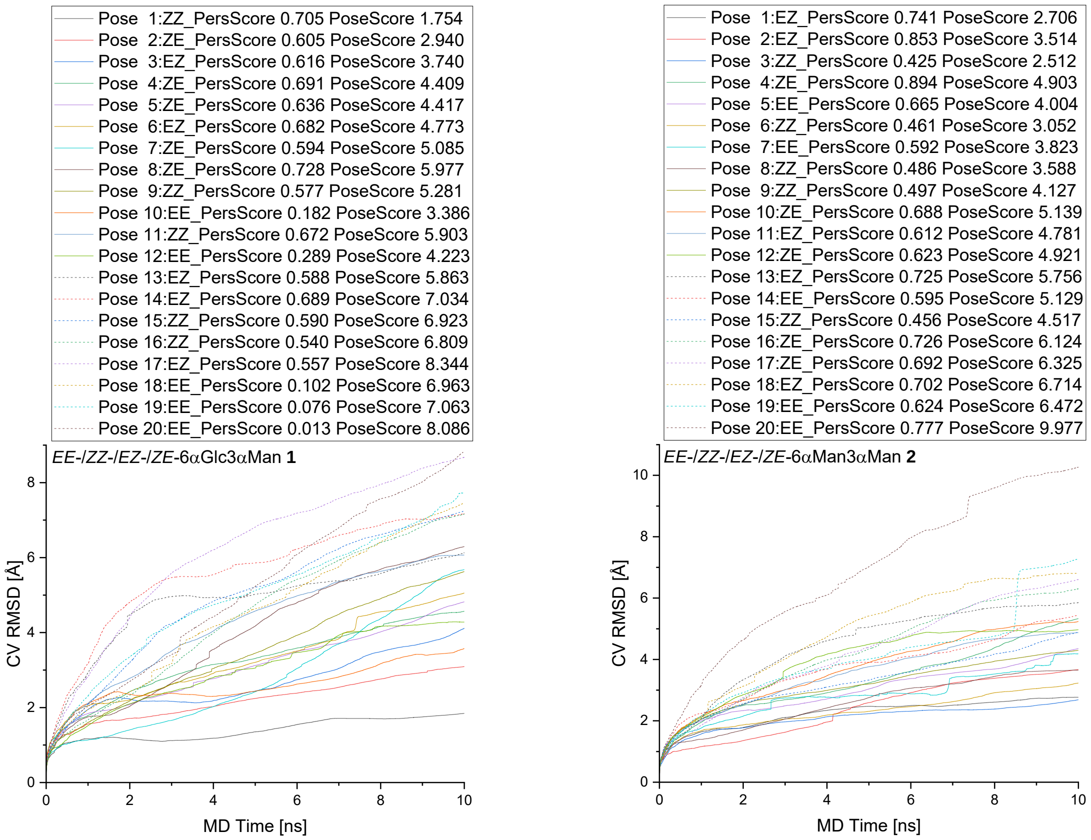

Figure S17: Averaged RMSD plotted as a collective variable (CV) in Å over the period of the binding pose metadynamics simulation of 6βGlc3αMan **1** (left) and 6αMan3αMan **2** (right). The most stable receptor–ligand complexes of each isomer (*EE*, *ZZ*, *EZ*, *ZE*) were incorporated into a MM-GBSA calculation (cf. Table S25).

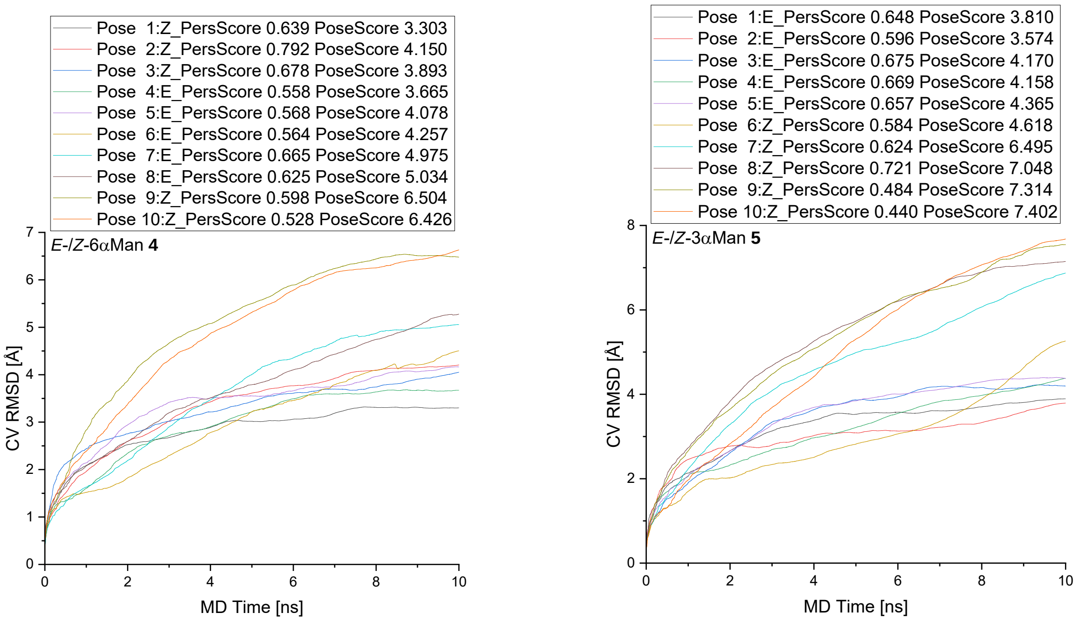

Figure S18: Averaged RMSD plotted as a collective variable (CV) in Å over the period of the binding pose metadynamics simulation of 6αMan **4** (left) and 3αMan **5** (right). The most stable receptor–ligand complexes of the respective *E* and *Z* isomer were incorporated into a MM-GBSA calculation (cf. Table S25).

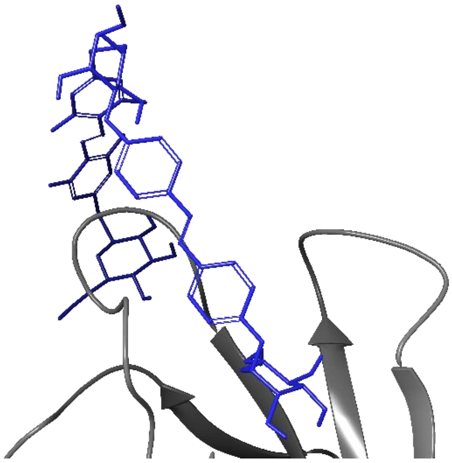

EE-6βGlc3αMan **1**

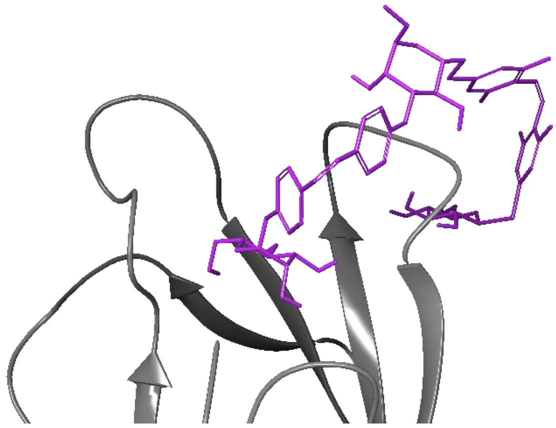

ZZ-6βGlc3αMan **1**

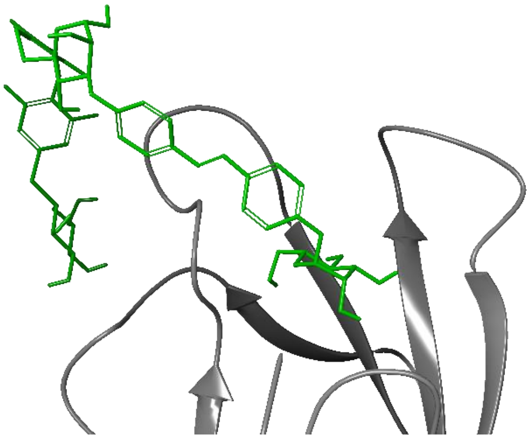

EZ-6βGlc3αMan **1**

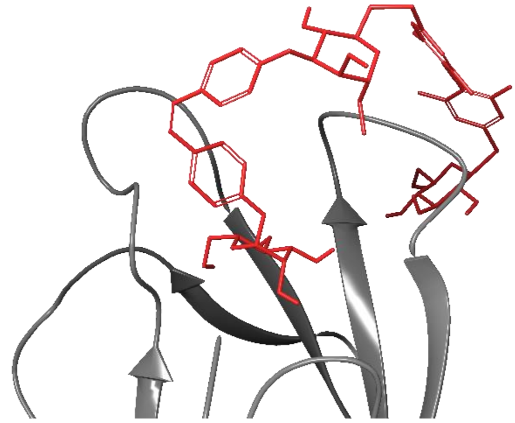

ZE-6βGlc3αMan **1**

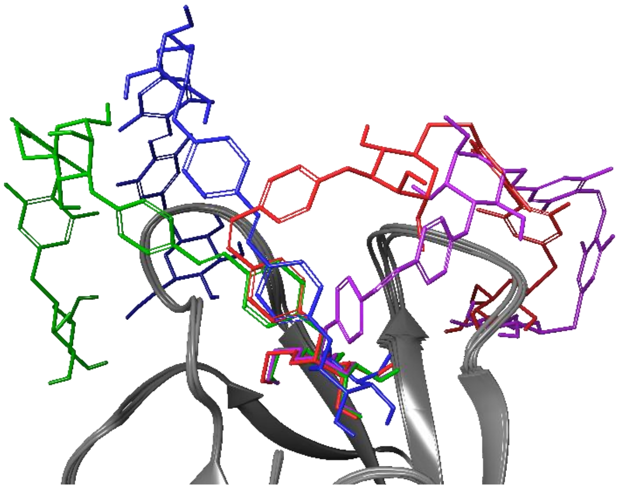

EE-/*ZZ*-/*EZ*-/*ZE*-6βGlc3αMan **1** superimposed

Figure S19: Three-dimensional representation of the most stable ligand-protein-complexes from IFD in 3D space of the four isomers *EE*, *ZZ*, *EZ*, and *ZE* of glycocluster 6βGlc3αMan **1** together with the superimposed conformations. The protein FimH (1UWF) is illustrated as ribbon diagram and the ligands are displayed as stick models (*EE*: blue; *ZZ*: violet; *EZ*: green; *ZE*: red). Superposition of the ligands shows the similarity of the binding of the terminal mannosides with FimH and the different orientation of the respective non-bonded antenna in three-dimensional space.

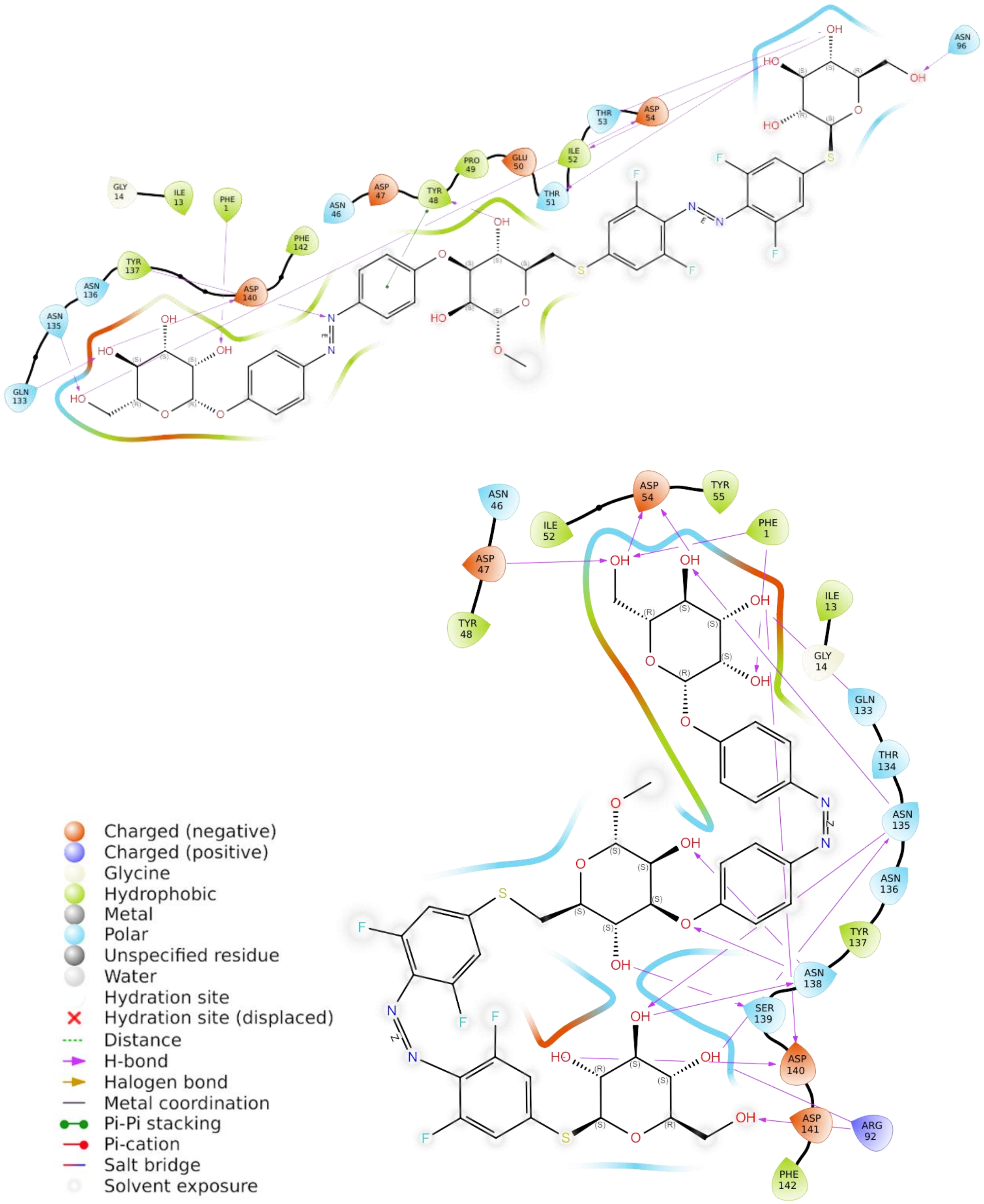

Figure S20: Left: Legend of symbols used for interactions present in the various ligand–protein complexes. Right: Two-dimensional representation of the most stable ligand–protein complexes from IFD of the isomers *EE* (top) and *ZZ* (bottom) of glycocluster 6βGlc3αMan **1**. The π–π interaction of the bound azobenzene glycoconjugate with Tyr48 further stabilizes the binding of the *EE* isomer in the binding pocket of FimH. The strong binding energy of the *ZZ* isomer can be explained by the additional hydrogen bonds of the terminal sugar of the non-bounded antenna with Arg92, Asn135, Asn138 and Asp140.

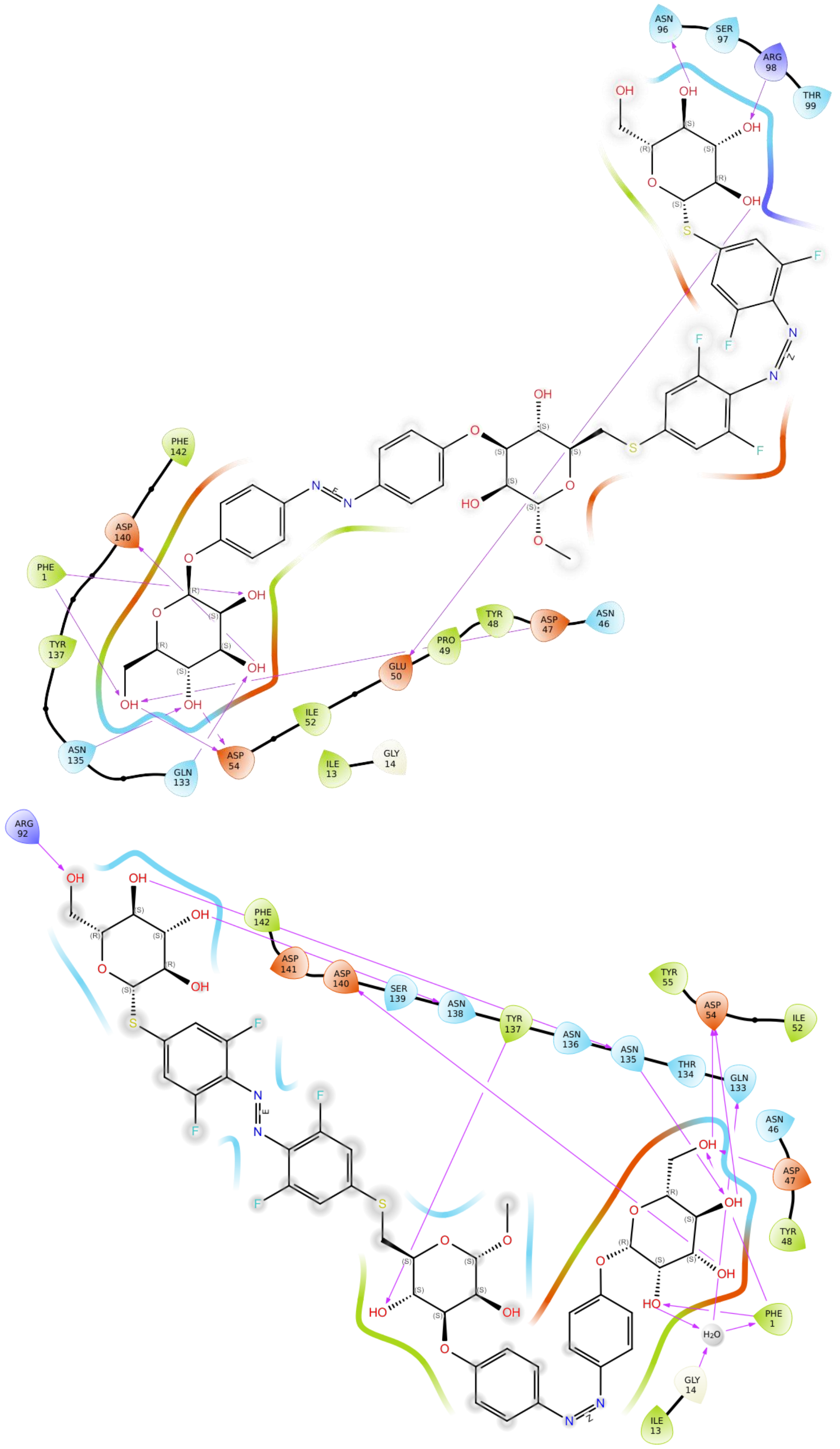

Figure S21: Two-dimensional representation of the most stable ligand–protein complexes from IFD of the isomers *EZ* (top) and *ZE* (bottom) of glycocluster 6βGlc3αMan **1**. The similar binding energy of the *EZ* isomer and *ZE* isomer despite the different spatial orientation of the second antenna can be explained by additional interactions of the terminal glucoside moiety with Asn96, Arg98 and Glu50 (for *EZ*) and Arg92, Asn138 and Asn135 (for *ZE*).

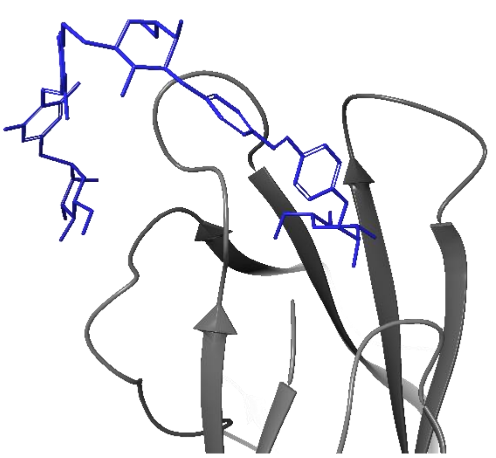

EE-6αMan3αMan **2**

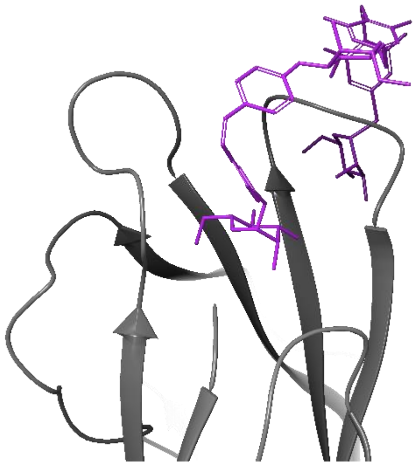

ZZ-6αMan3αMan **2**

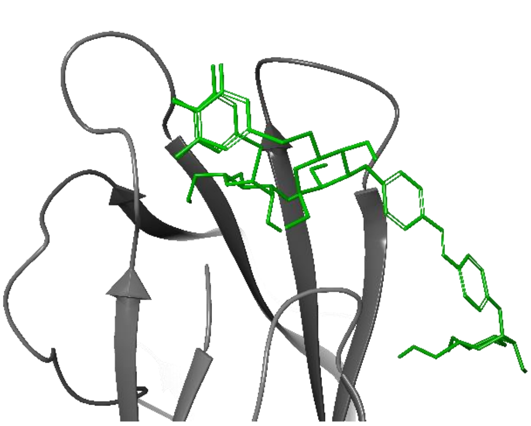

EZ-6αMan3αMan **2**

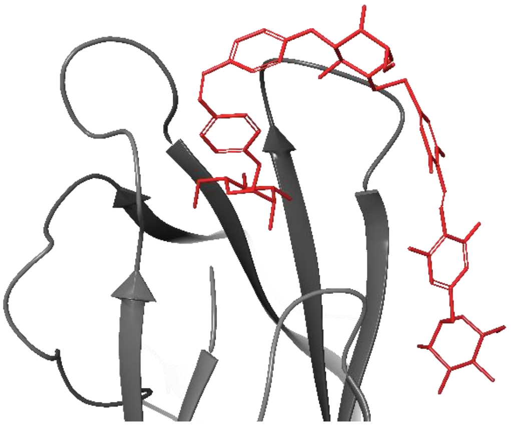

ZE-6αMan3αMan **2**

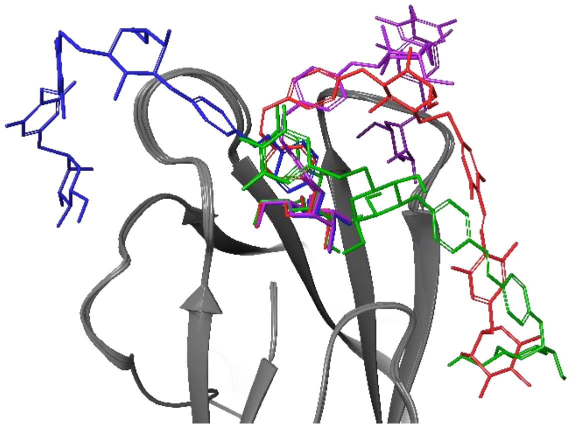

EE-/*ZZ*-/*EZ*-/*ZE*-6αMan3αMan **2** superimposed

Figure S22: Three-dimensional representation of the most stable ligand–protein complexes from IFD of the isomers *EE*, *ZZ*, *EZ*, and *ZE* of glycocluster 6αMan3αMan **2** together with the superimposed conformations. The protein FimH (1UWF) is illustrated as ribbon diagram and the ligands are displayed as stick models (*EE*: blue; *ZZ*: violet; *EZ*: green; *ZE*: red). Superposition of the ligands shows that the interaction of the terminal mannoside moiety with FimH is similar in all isomeric states, whereas and the orientation of the respective non-bounded glycoantenna is different.

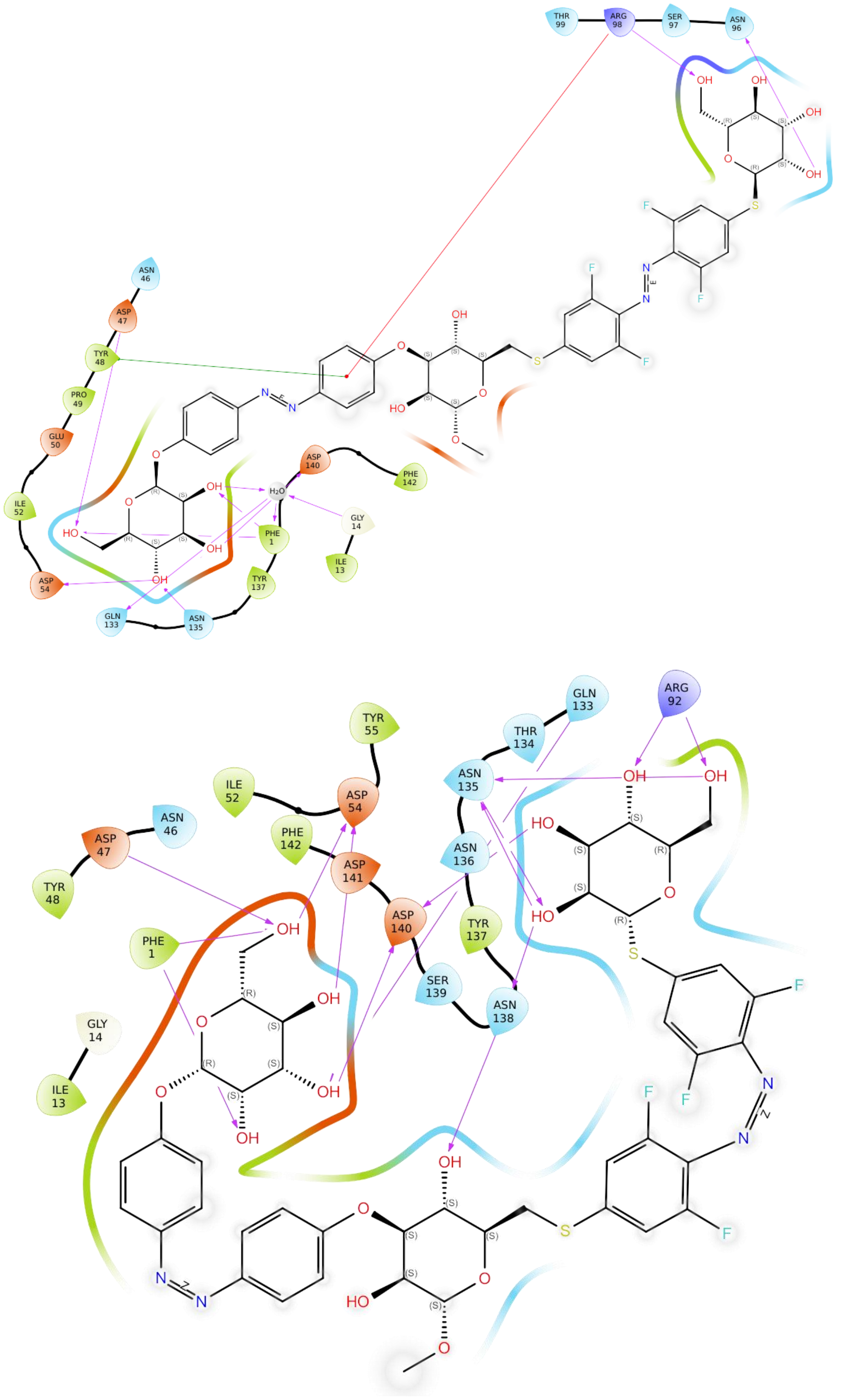

Figure S23: Two-dimensional representation of the most stable ligand–protein complexes from IFD of the isomers *EE* (top) and *ZZ* (bottom) of glycocluster 6αMan3αMan **2**. The π–π interaction of the bounded azobenzene glycoconjugate with Tyr48 further stabilizes the complexation of the *EE* isomer in the binding pocket of FimH. The strong binding energy of the *ZZ* isomer can be explained by additional hydrogen bonds of the terminal sugar moiety of the non-bounded antenna with Arg92, Asn135, Asn138 and Asp140.

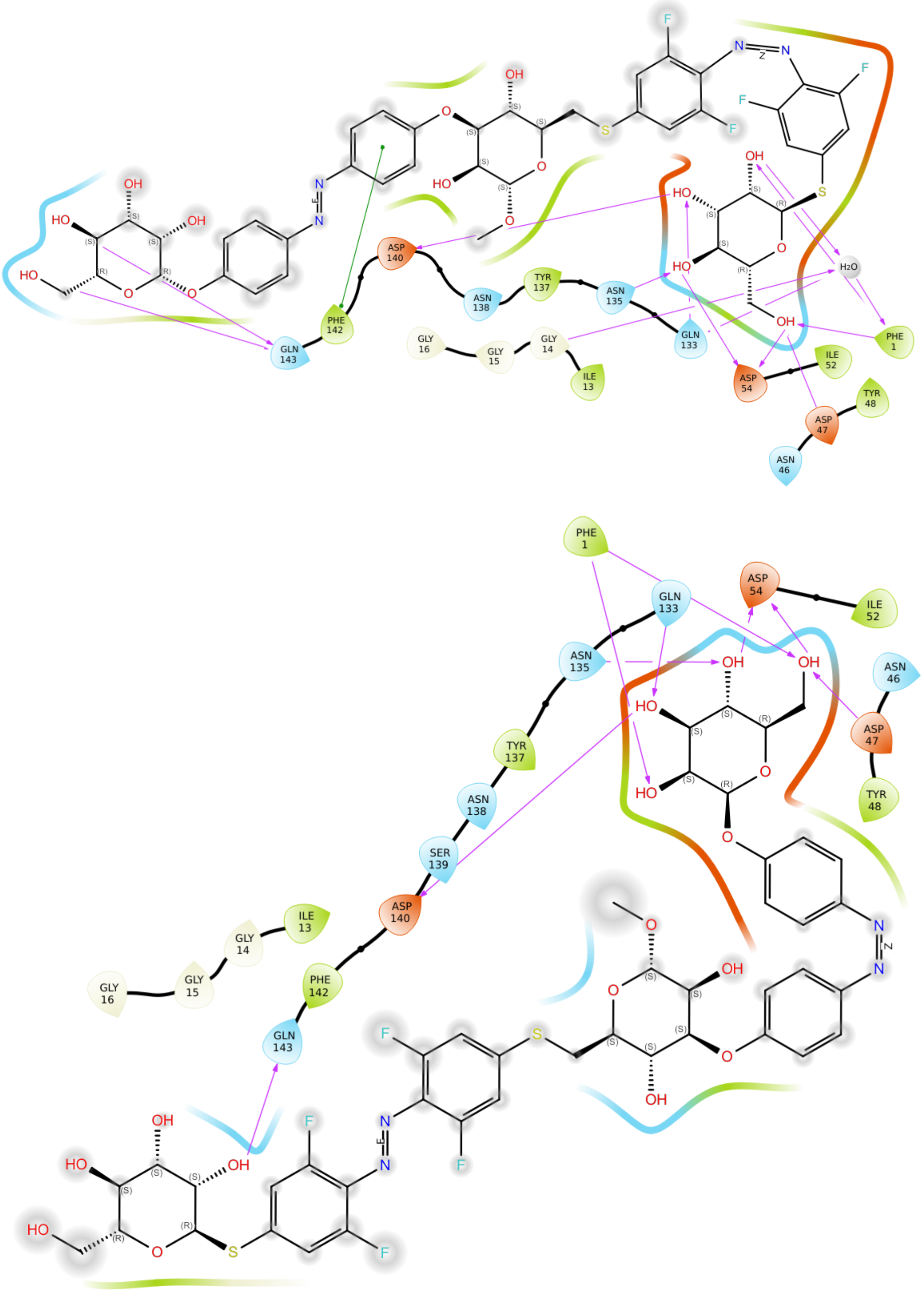

Figure S24: Two-dimensional representation of the most stable ligand–protein complexes from IFD of the isomers *EZ* (top) and *ZE* (bottom) of glycocluster 6αMan3αMan **2**. In both cases the *Z*-antenna is docked into the binding pocket of FimH. Interestingly, the non-bounded terminal sugar of the isomers both interact with Gln143 located at the backside of the binding pocket of FimH.

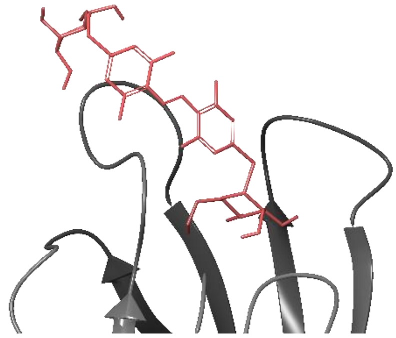

E-6αMan **4**

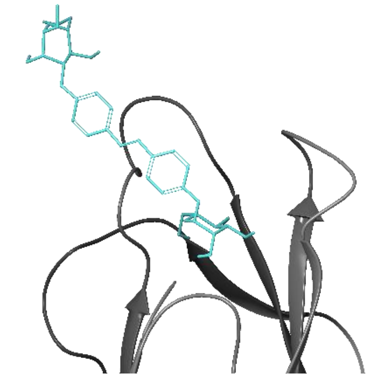

E-3αMan **5**

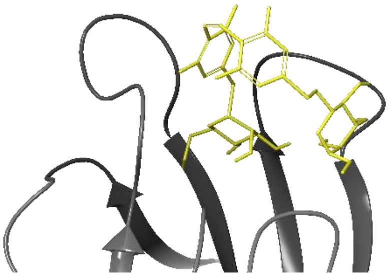

Z-6αMan **4**

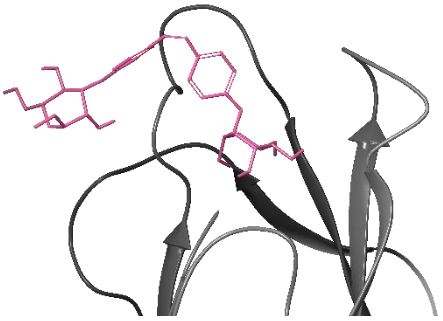

Z-3αMan **5**

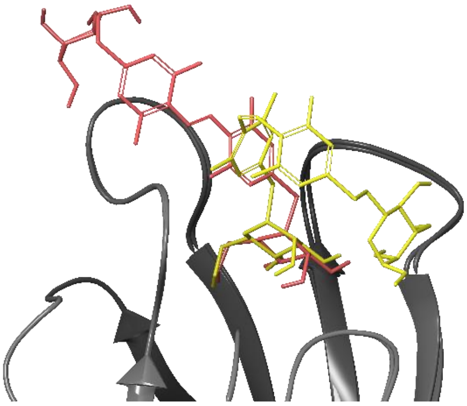

E- and *Z*-6αMan **4** superimposed

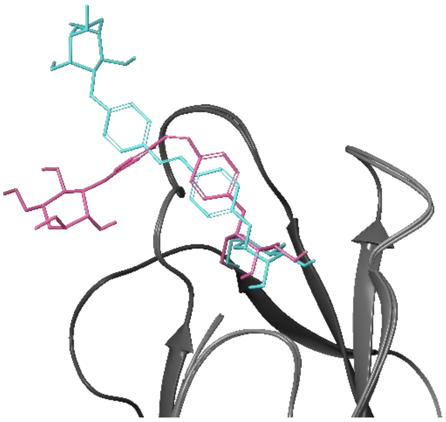

E- and *Z*-3αMan **5** superimposed

Figure S25: Three-dimensional representation of the most stable ligand–protein complexes from IFD of the isomers *E* and *Z* of the antennas 6αMan **4** (left) and 3αMan **5** (right) together with the superimposed conformations of **4** and **5**, respectively. The protein FimH (1UWF) is illustrated as ribbon diagram and the ligands are displayed as stick models (6αMan **4** (right): *E*: bright red; *Z*: yellow; 3αMan **5** (left): *E*: turquoise; *Z*: bright pink). Superposition of the ligands shows the similarity of the binding of the terminal mannoside moieties with FimH and the different orientation of the respective non-bonded scaffold of the antennas in the different isomeric states.

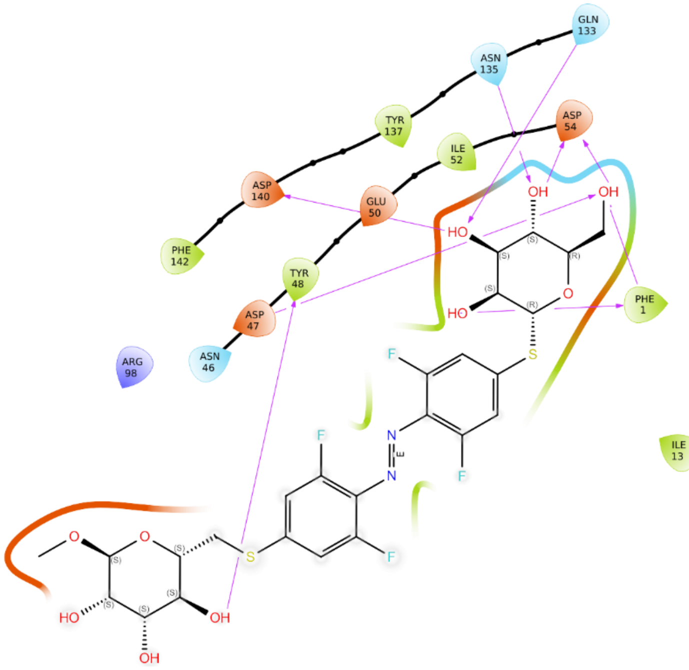

E-6αMan **4**

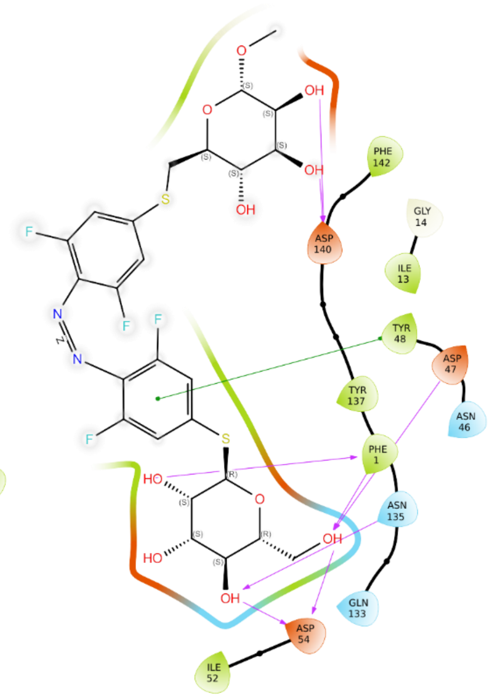

Z-6αMan **4**

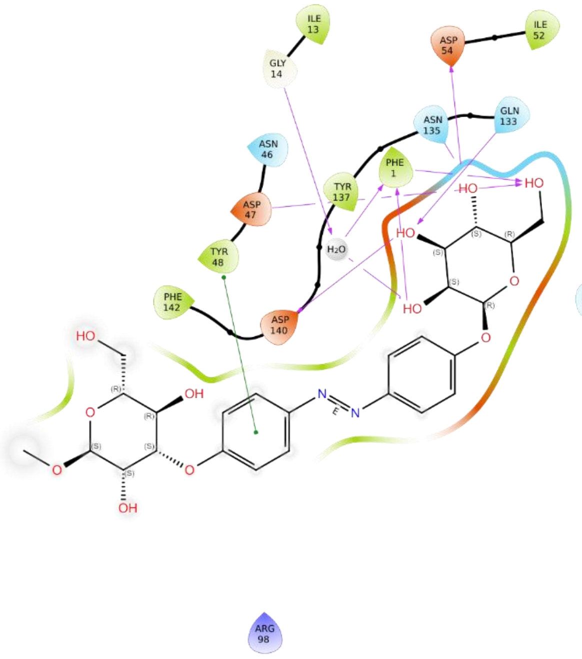

E-3αMan **5**

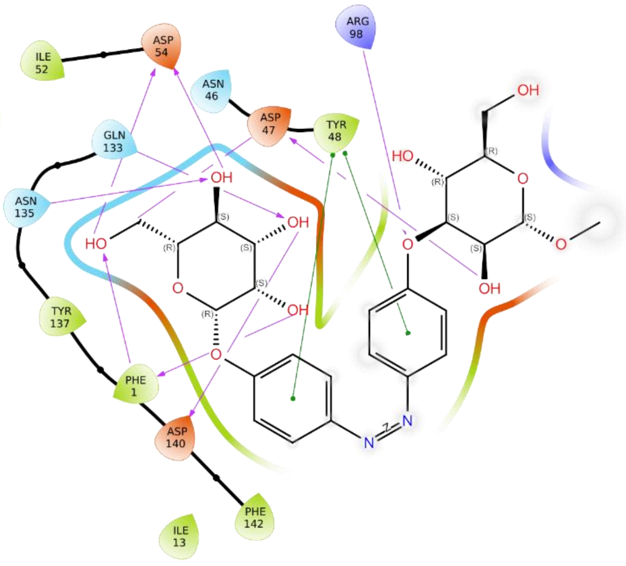

Z-3αMan **5**

Figure S26: Two-dimensional representation of the most stable ligand–protein complexes from IFD of the isomers *E* and *Z* of the antennas 6αMan **4** (top) and 3αMan **5** (bottom). Even with the calculated π–π interaction to Tyr48 and the interaction of the scaffold mannoside to Asp140 of the *Z* isomer of 6αMan, a difference in binding energy between the *E* and *Z* isomers is seen. The similar binding energies of the *E* and *Z* isomers of 3αMan can be explained by the additional π–π interaction to Tyr48 and H-bond interaction of the scaffold mannoside of the *Z* isomer with Asn46 and Arg98.

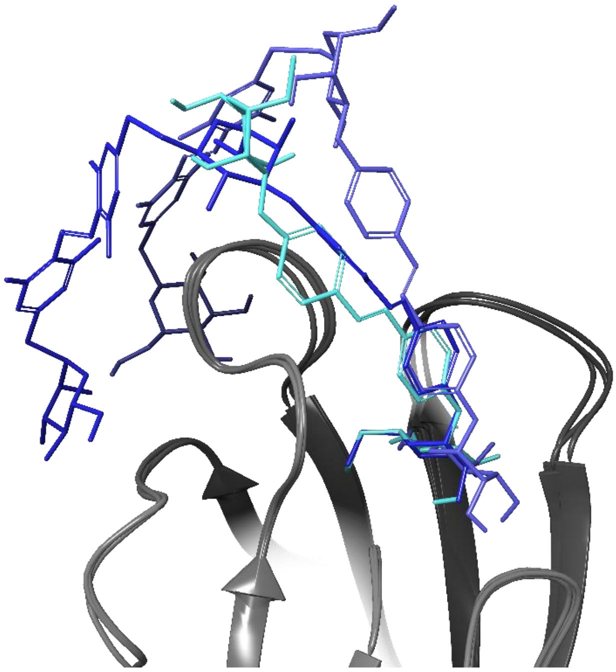

Figure S27: Superimposition of the antenna *E*-3αMan **5** (turquoise) with glycocluster *EE*-6αGlc3αMan **1** (bright blue) as well as *EE*-6αMan3αMan **2** (blue) illustrating the same spatial orientation of the ligands in the periphery of the binding pocket.

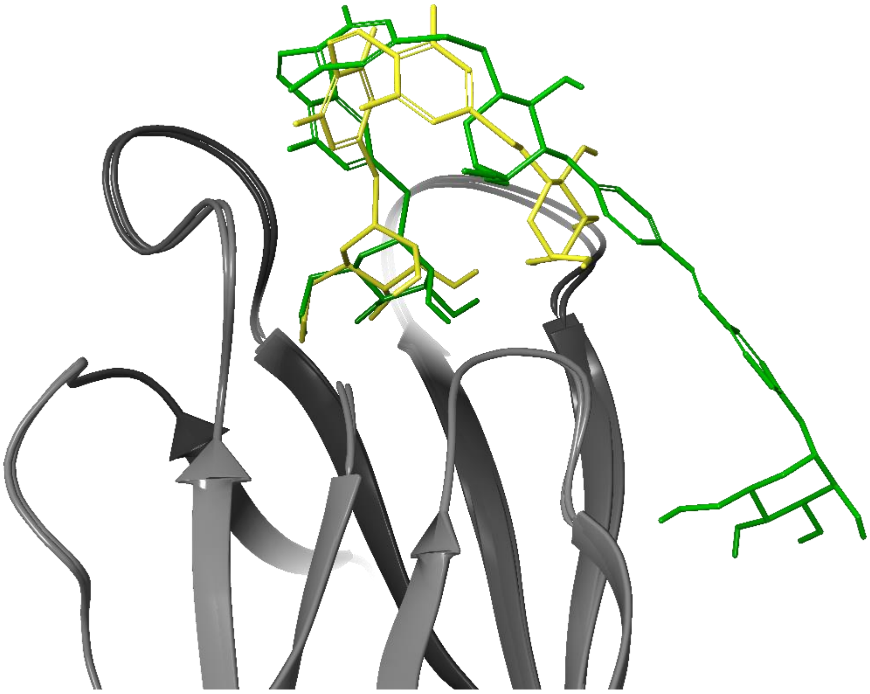

Figure S28: Superimposition of the antenna *Z*-6αMan **4** (yellow) with glycocluster *EZ*-6αMan3αMan **2** (green) illustrates the same spatial orientation of the ligands in the periphery of the binding pocket.

5 ^{1}H, ^{19}F and ^{13}C NMR spectra

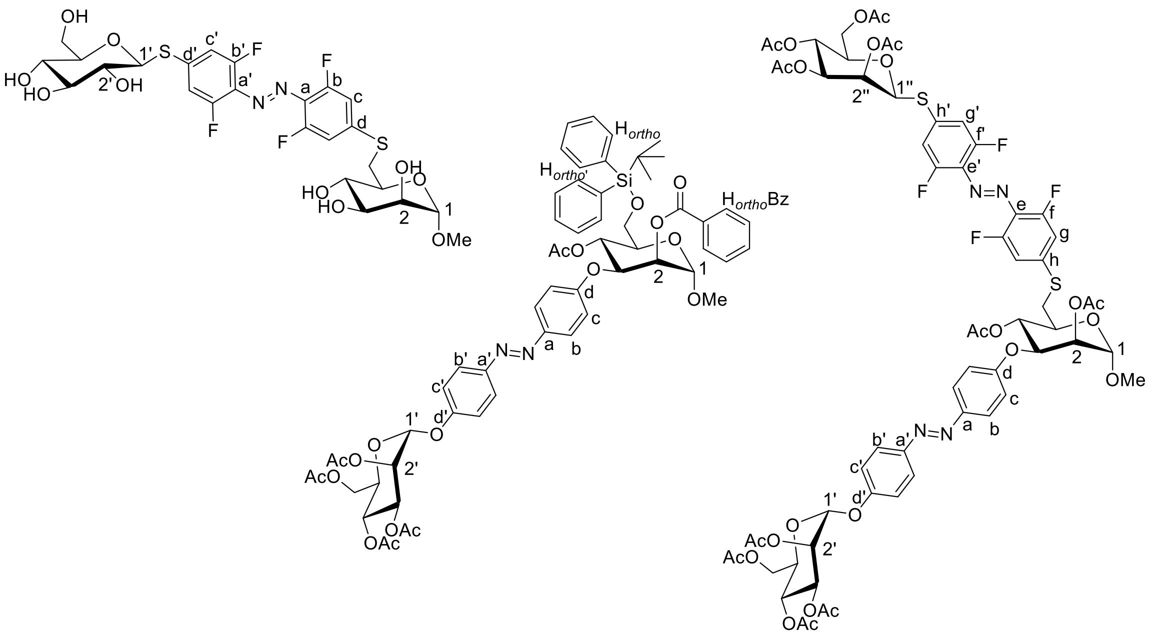

Scheme S3: For NMR assignment the depicted numbering and indices were used.

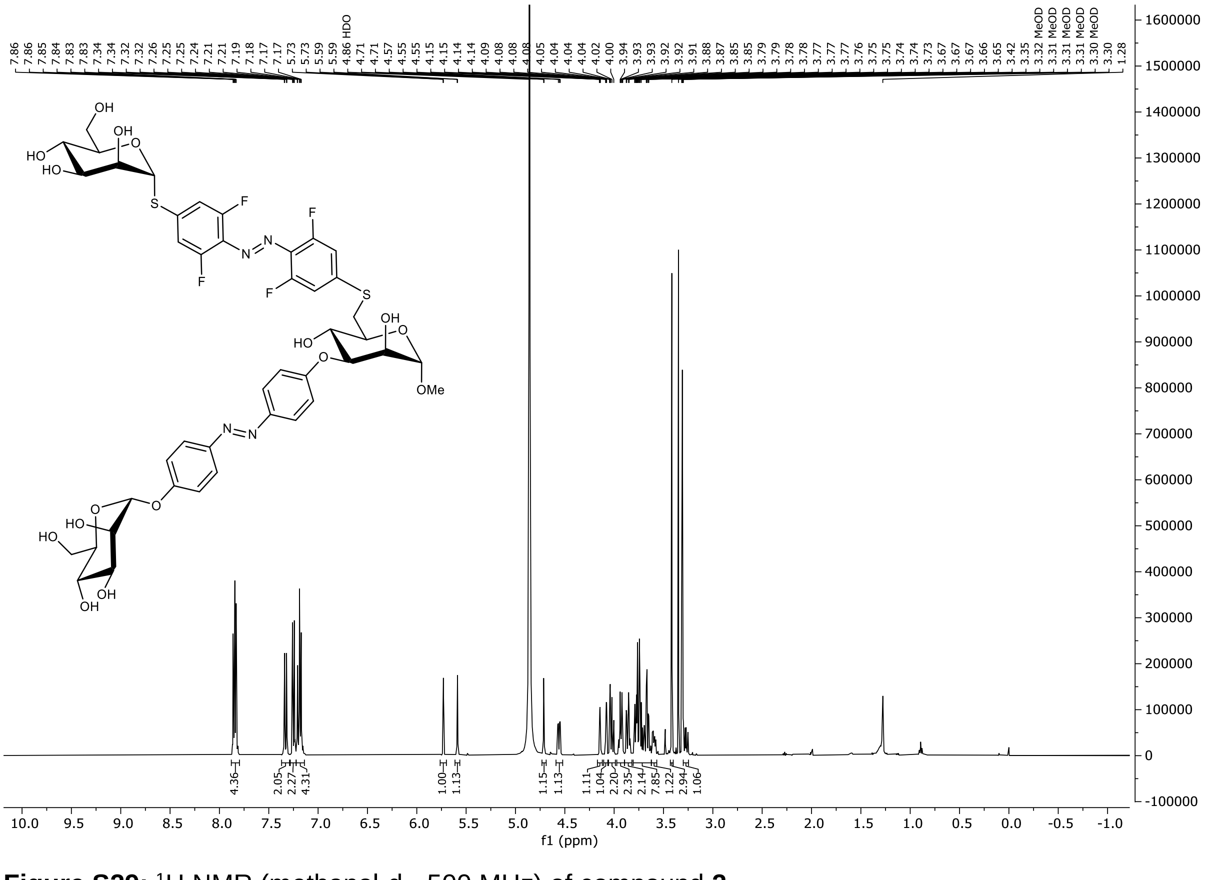

Figure S29: ^{1}H NMR (methanol-d$_4$, 500 MHz) of compound **2**.

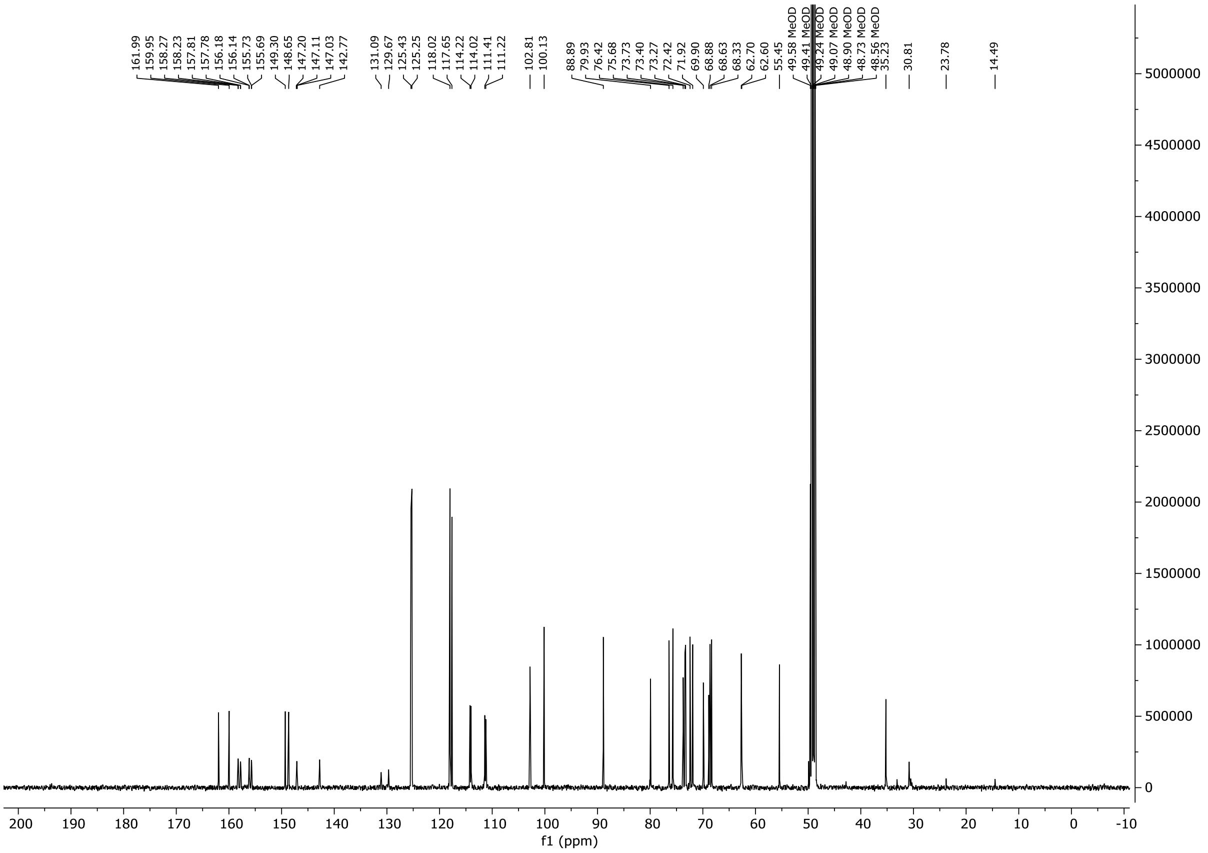

Figure S30: ^{13}C NMR (methanol-d$_4$, 126 MHz) of compound **2**.

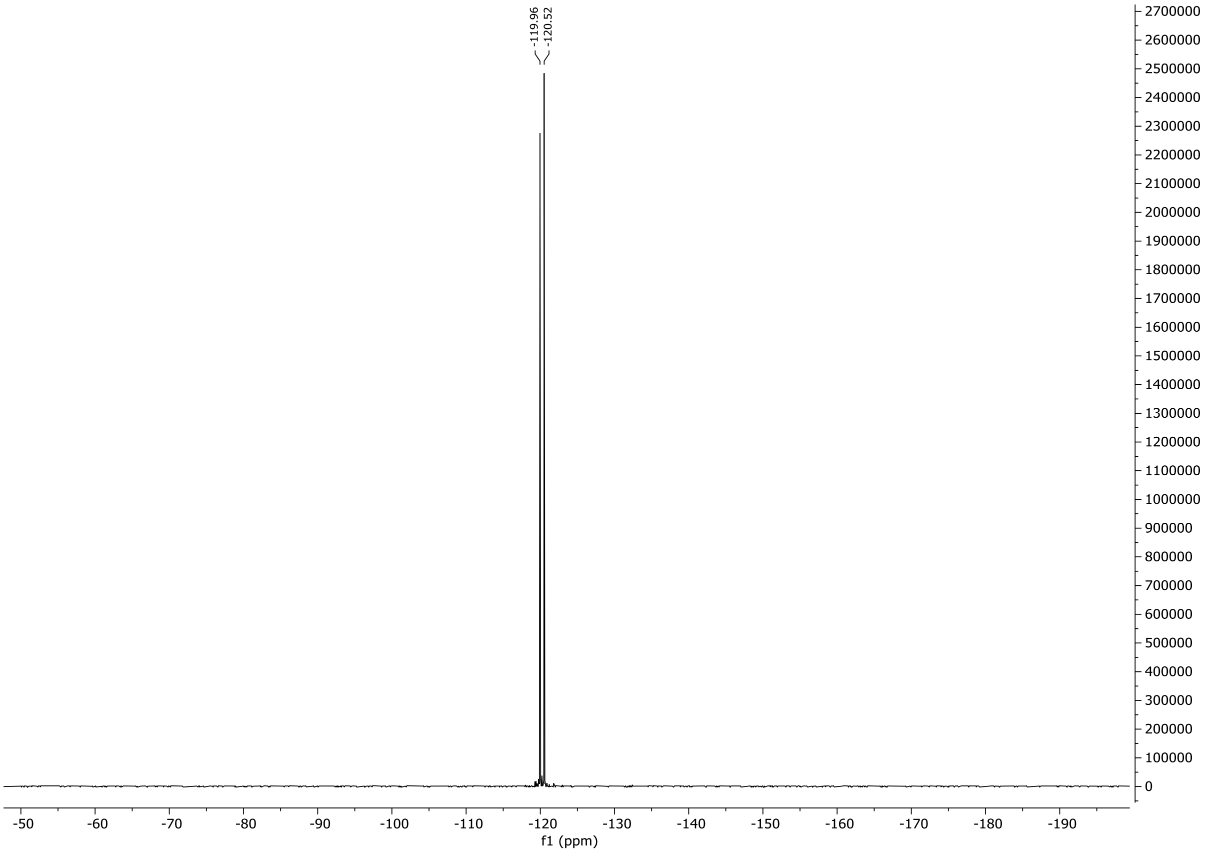

Figure S31: ^{19}F NMR (methanol-d$_4$, 471 MHz) of compound **2**.

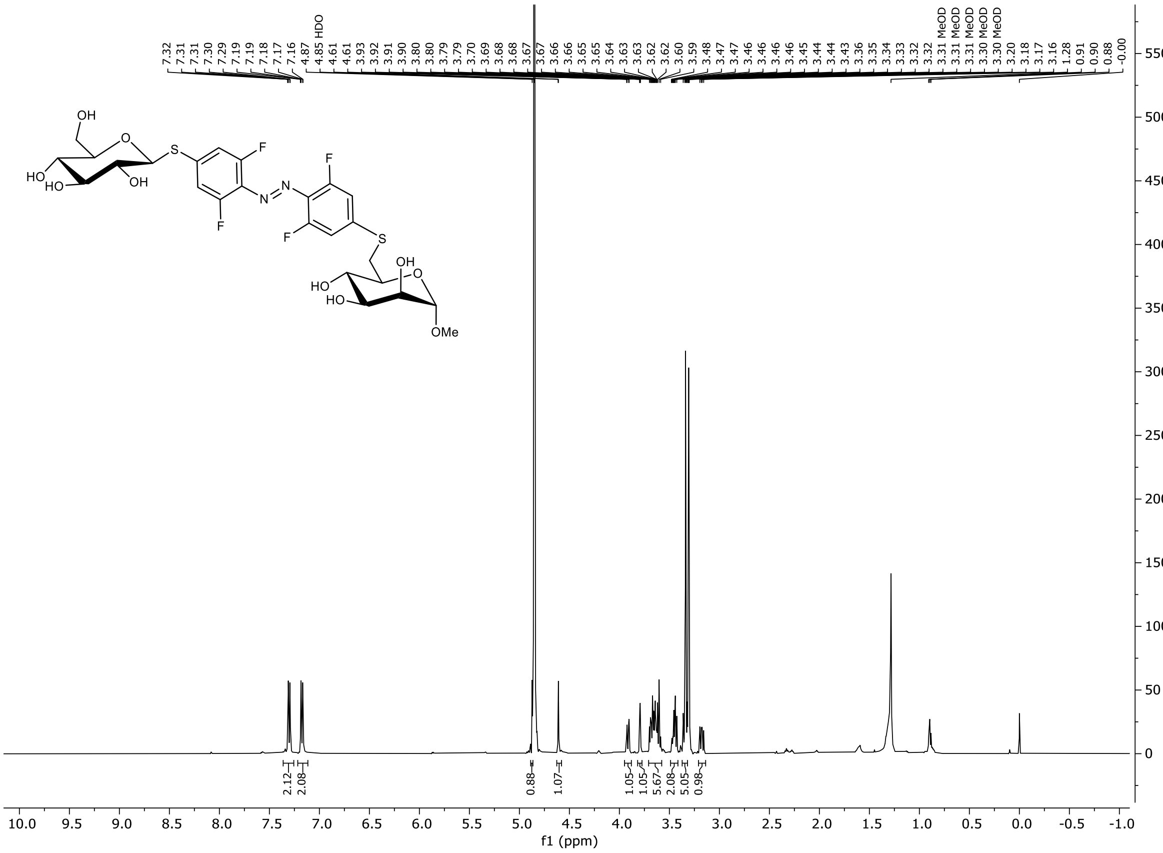

Figure S32: ^{1}H NMR (methanol-d$_4$, 600 MHz) of compound **3**.

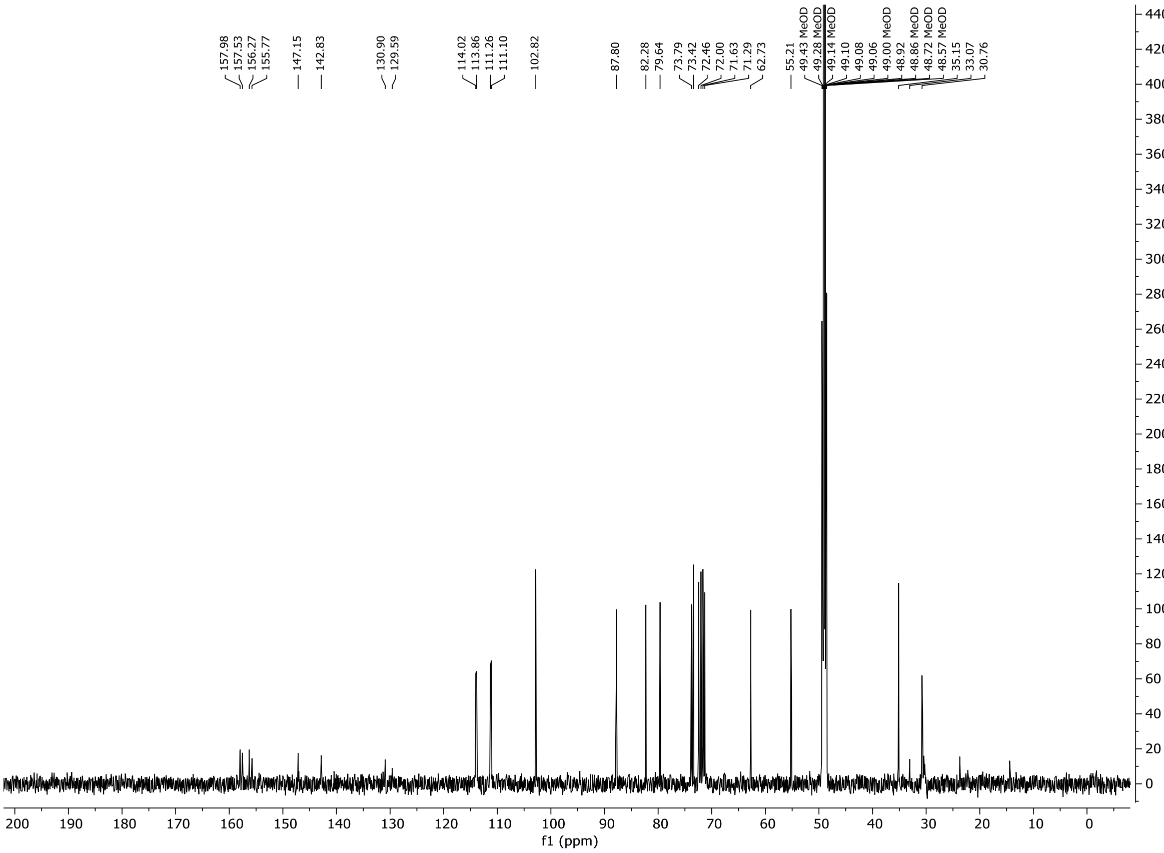

Figure S33: ^{13}C NMR (methanol-d$_4$, 151 MHz) of compound **3**.

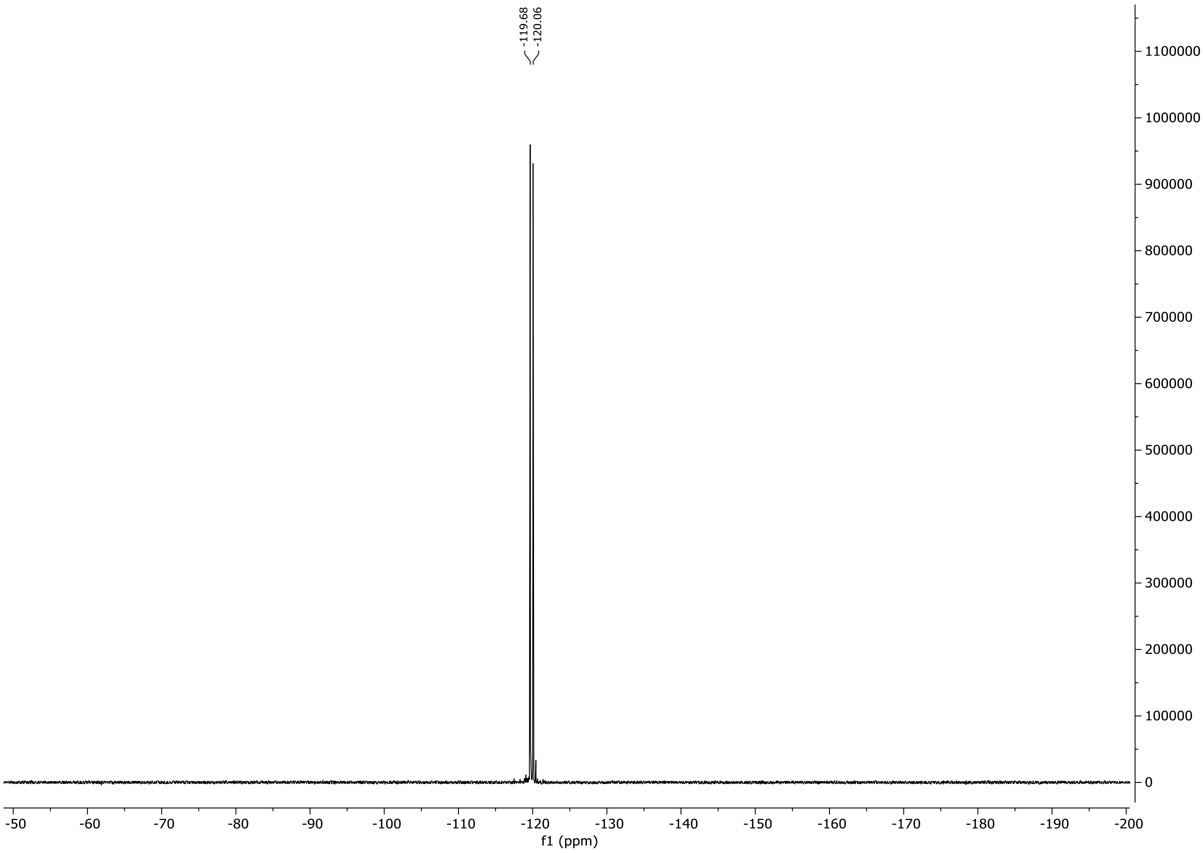

Figure S34: ^{19}F NMR (methanol-d$_4$, 471 MHz) of compound **3**.

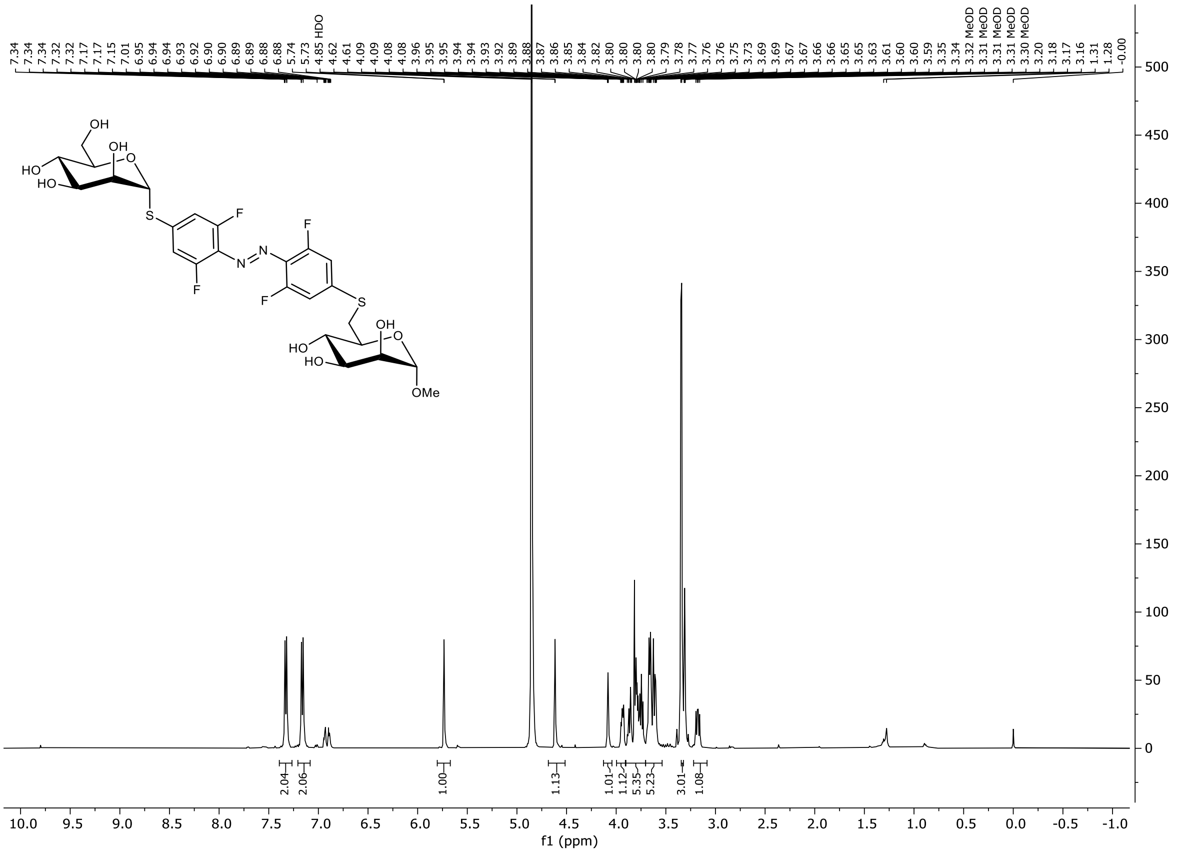

Figure S35: ^{1}H NMR (methanol-d$_4$, 600 MHz) of compound **4**.

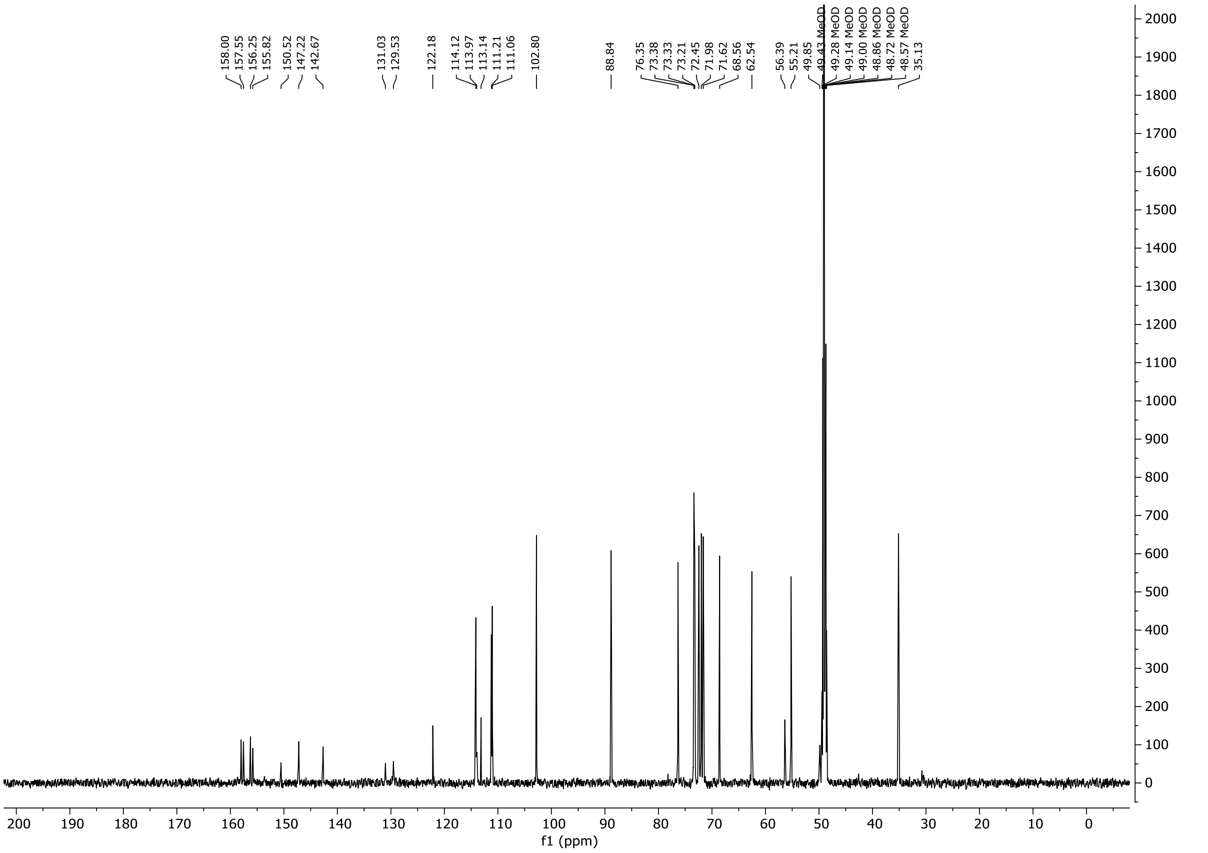

Figure S36: ^{13}C NMR (methanol-d$_4$, 151 MHz) of compound **4**.

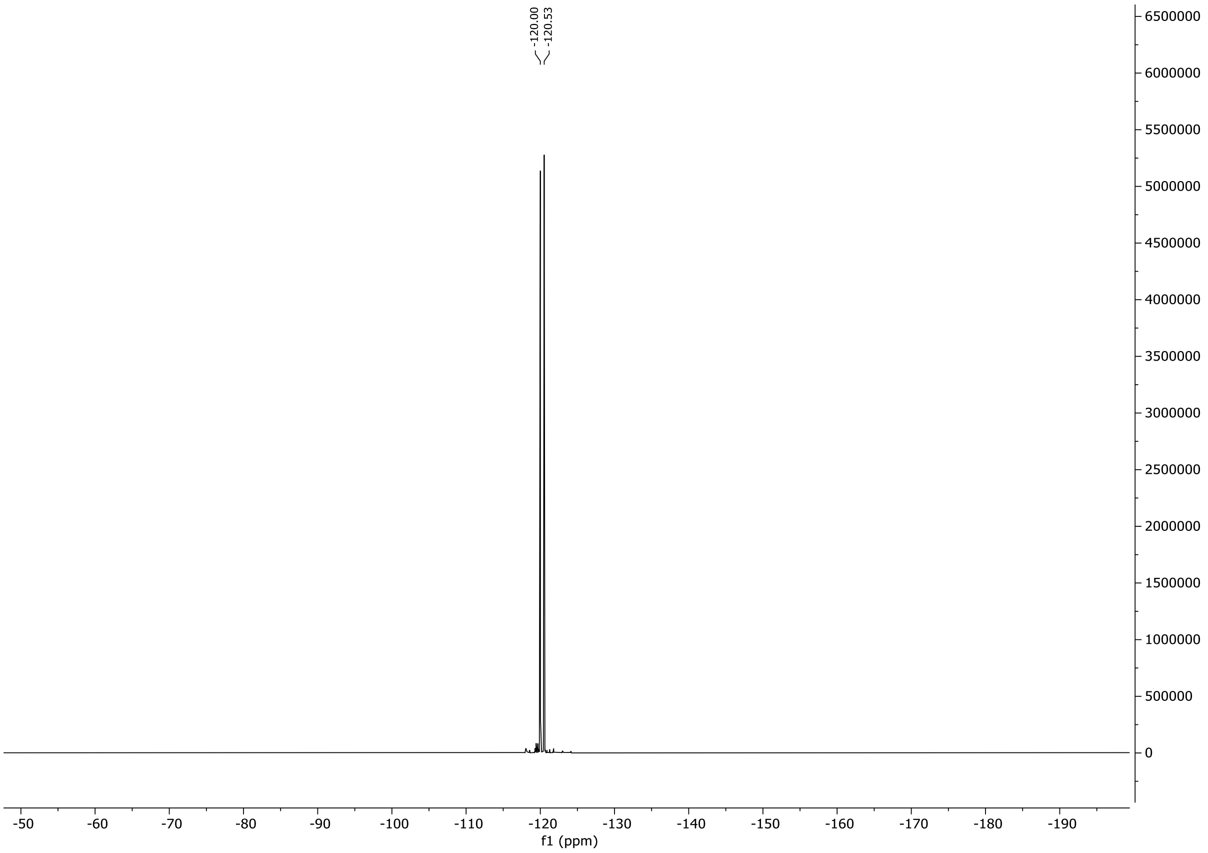

Figure S37: ^{19}F NMR (DMSO-d$_6$, 471 MHz) of compound **4**.

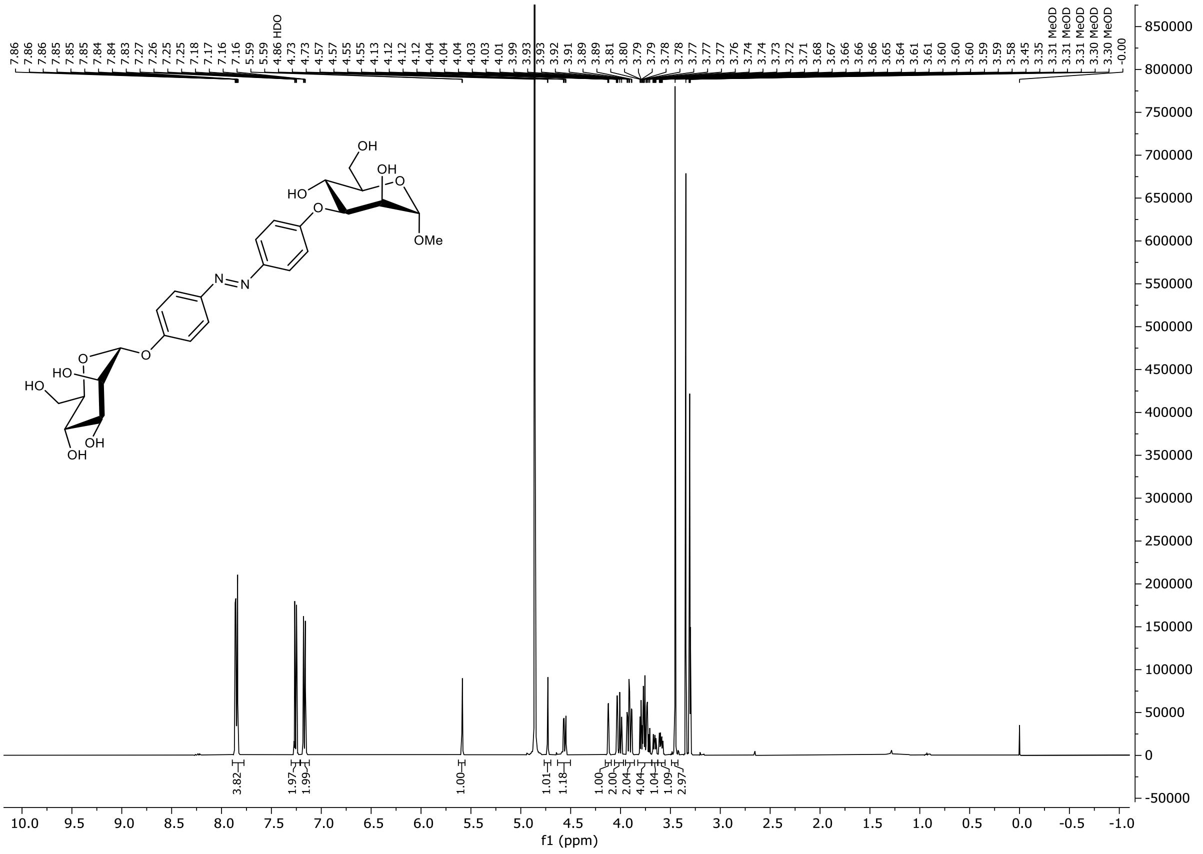

Figure S38: ^{1}H NMR (methanol-d_4, 500 MHz) of compound **5**.

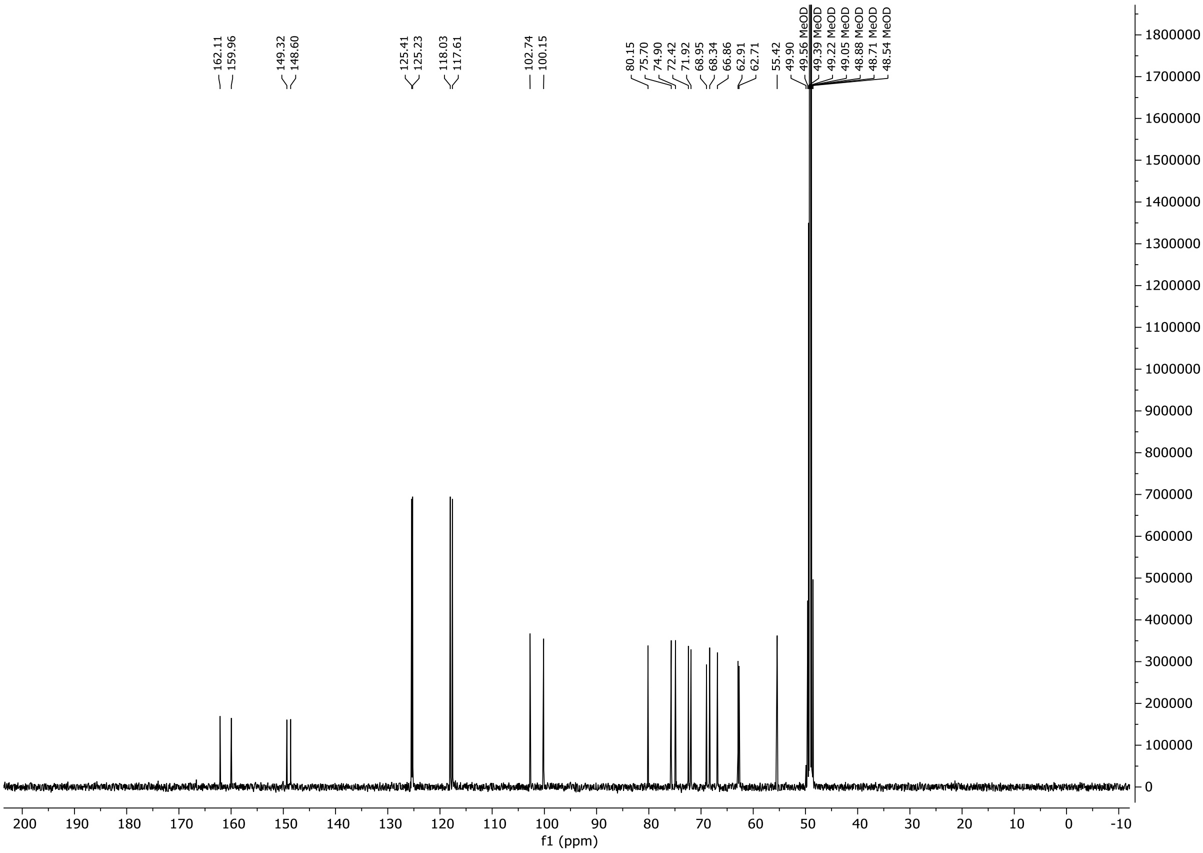

Figure S39: ^{13}C NMR (methanol-d_4, 126 MHz) of compound **5**.

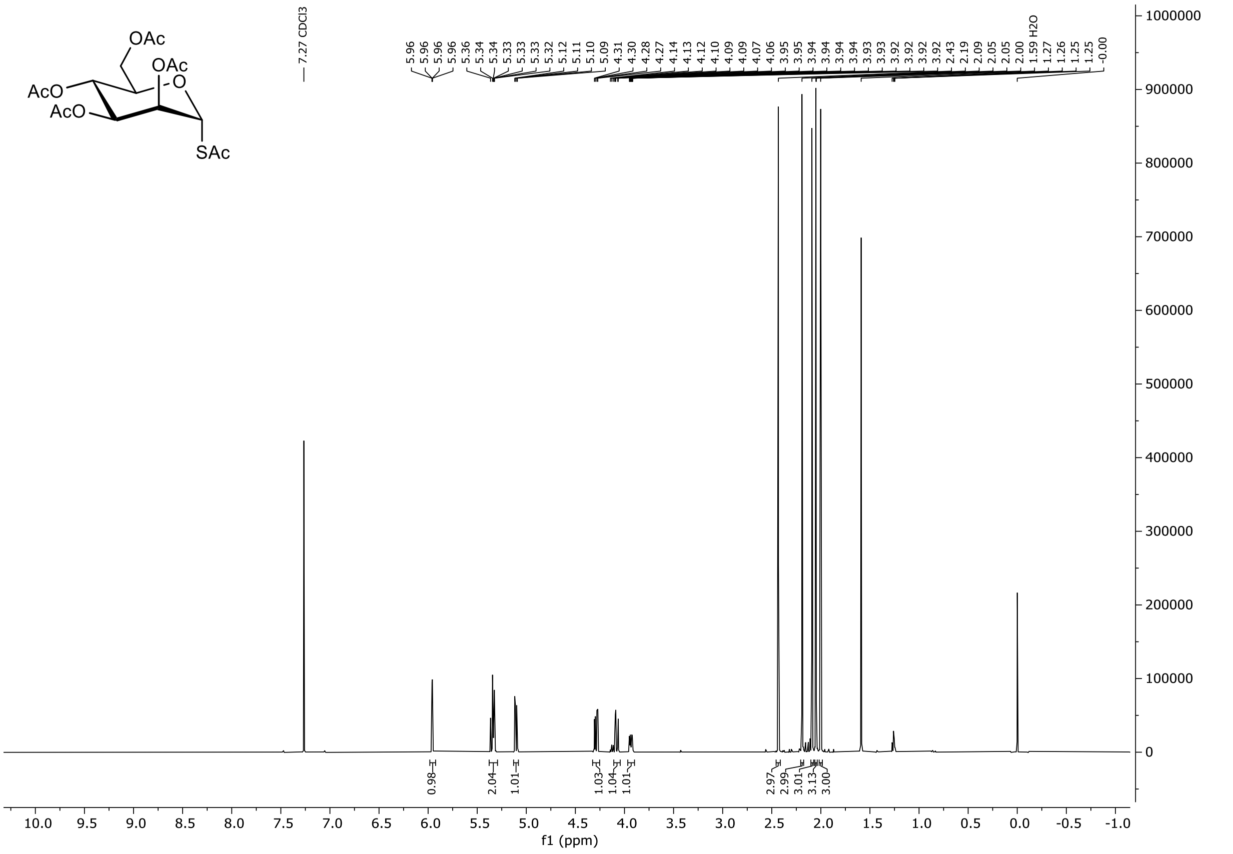

Figure S40: ^{1}H NMR ($CDCl_3$, 500 MHz) of compound **7**.

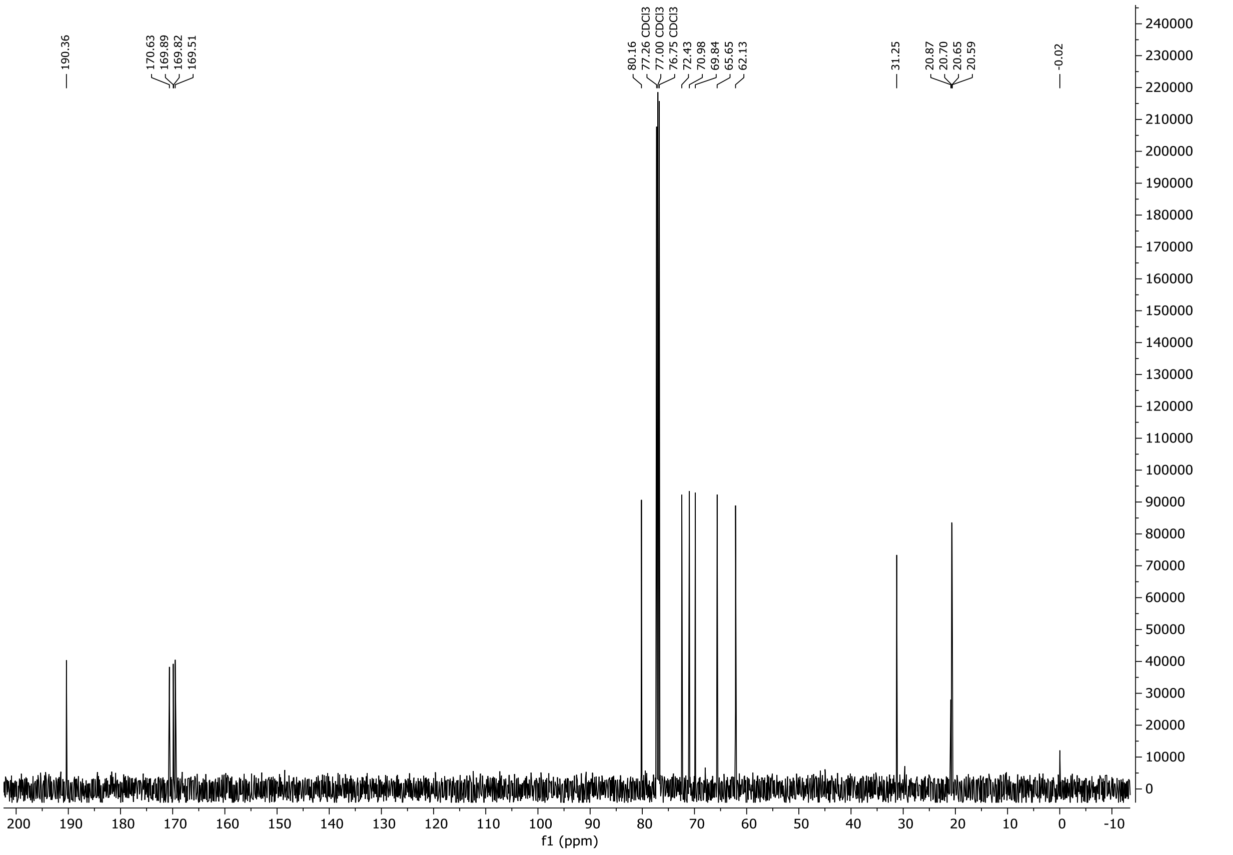

Figure S41: ^{13}C NMR ($CDCl_3$, 126 MHz) of compound **7**.

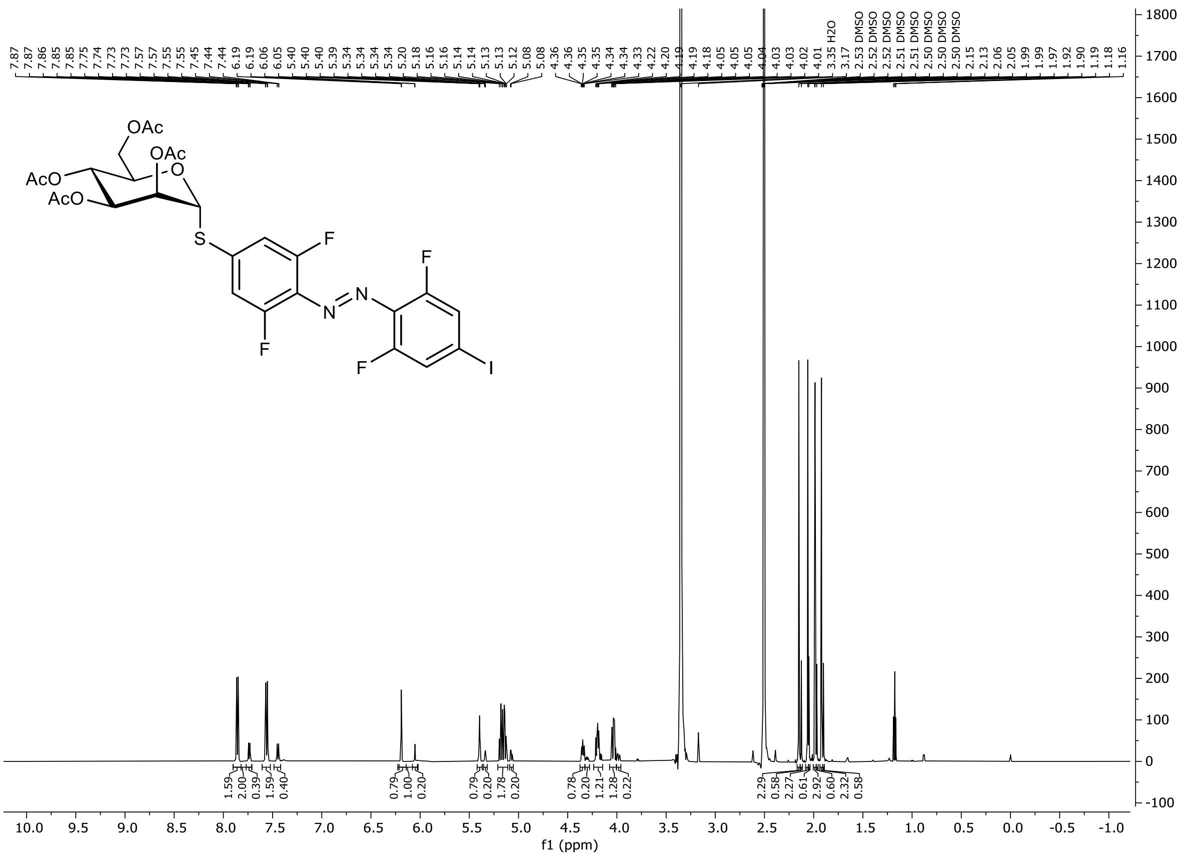

Figure S42: ^{1}H NMR (DMSO-d$_6$, 600 MHz) of compound **10** (*E*/*Z* ratio 8:2).

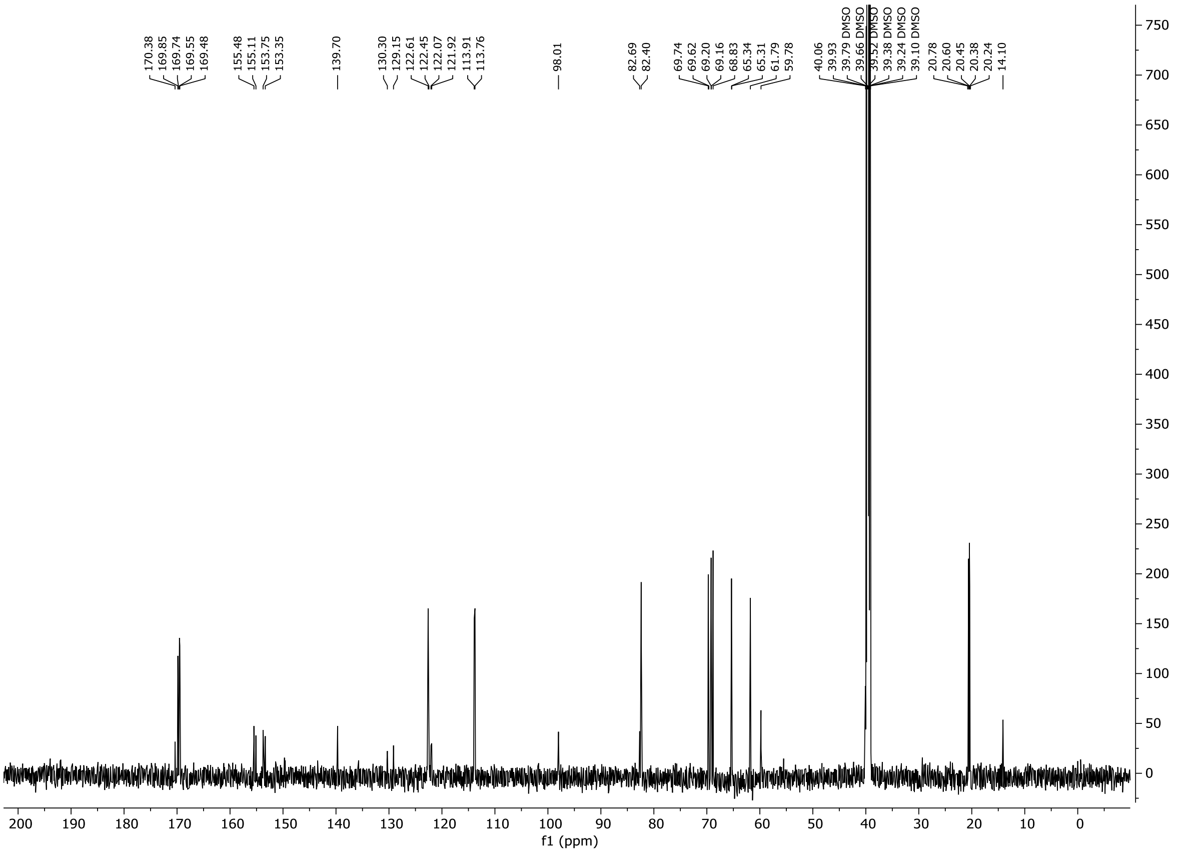

Figure S43: ^{13}C NMR (DMSO-d$_6$, 151 MHz) of compound **10** (*E*/*Z* ratio 8:2).

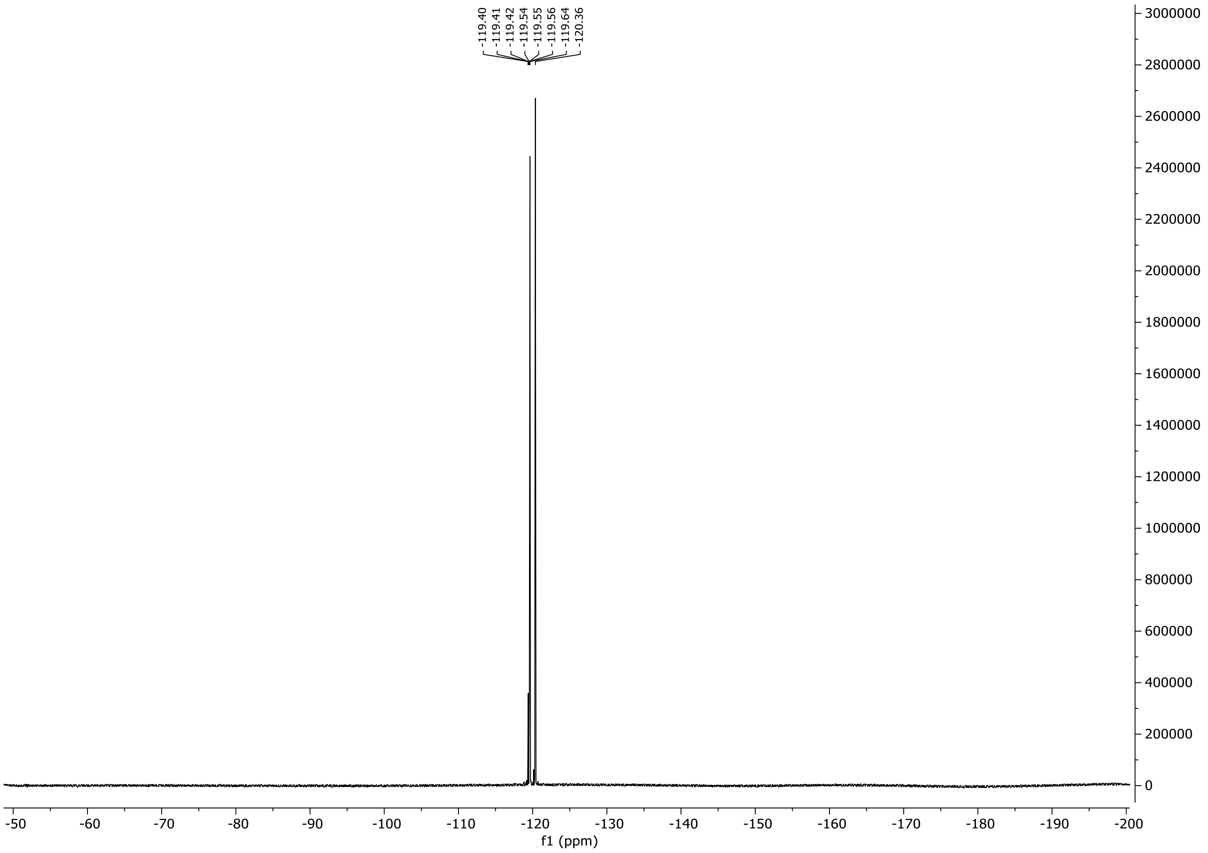

Figure S44: ^{19}F NMR (DMSO-d_6, 471 MHz) of compound **10** (*E*/*Z* ratio 8:2).

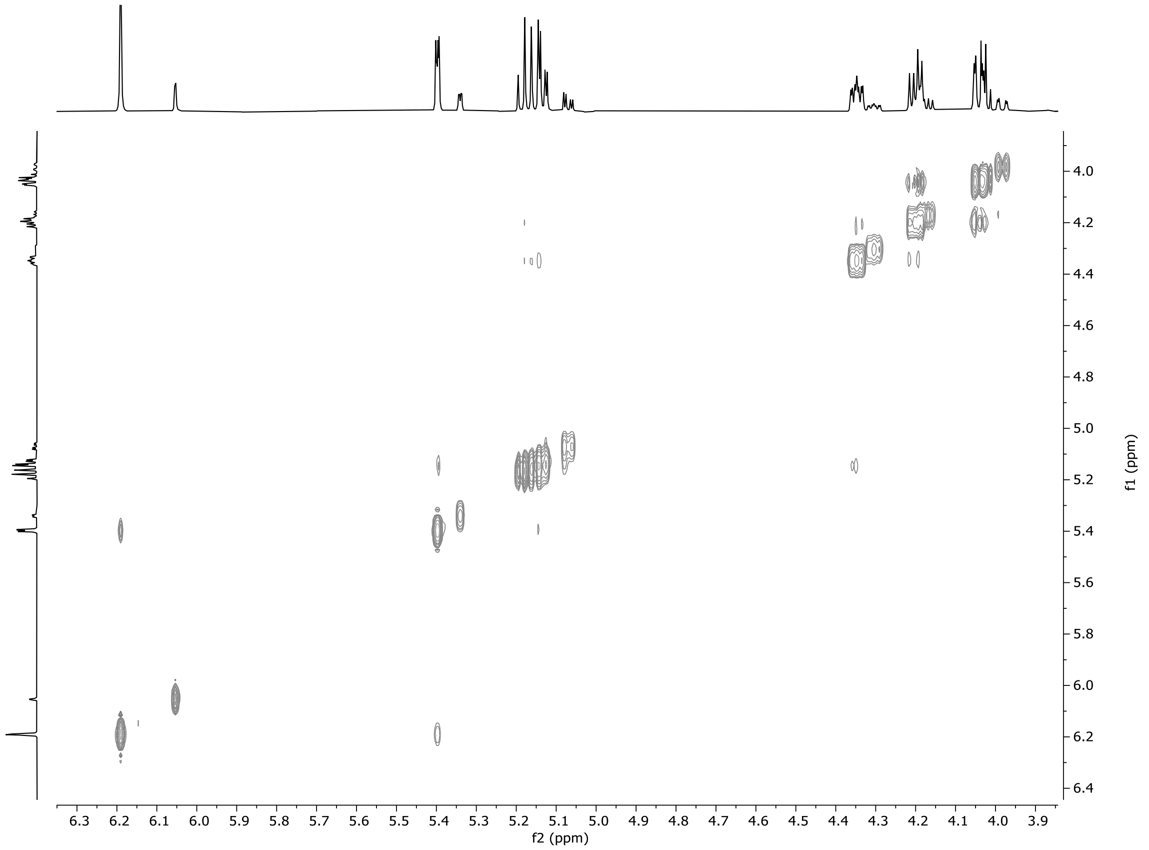

Figure S45: ^{1}H,^{1}H NOESY (DMSO-d_6, 600 MHz, 600 MHz) of compound **10** (*E*/*Z* ratio 8:2). No cross peaks neither between H-1 and H-3 nor between H-1 and H-5 can be detected.

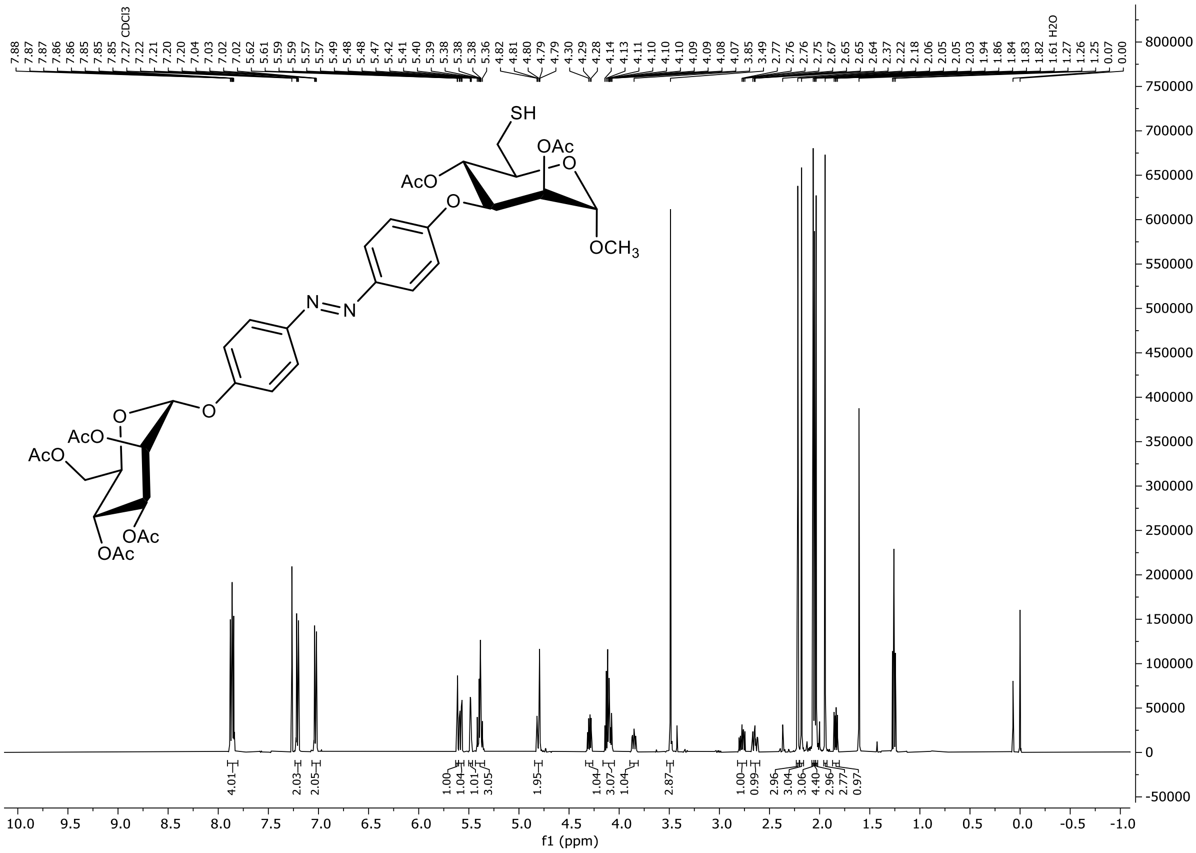

Figure S46: ^{1}H NMR ($CDCl_3$, 500 MHz) of compound **11**.

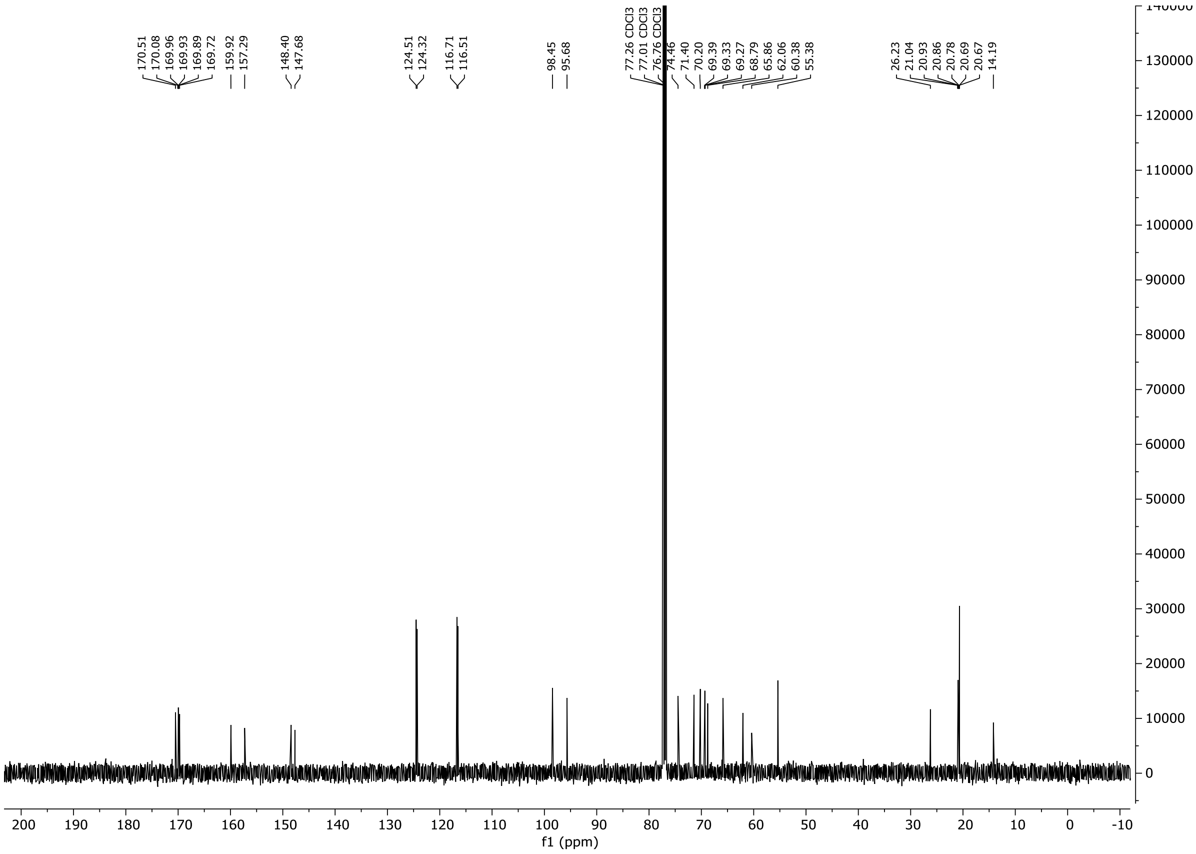

Figure S47: ^{13}C NMR ($CDCl_3$, 126 MHz) of compound **11**.

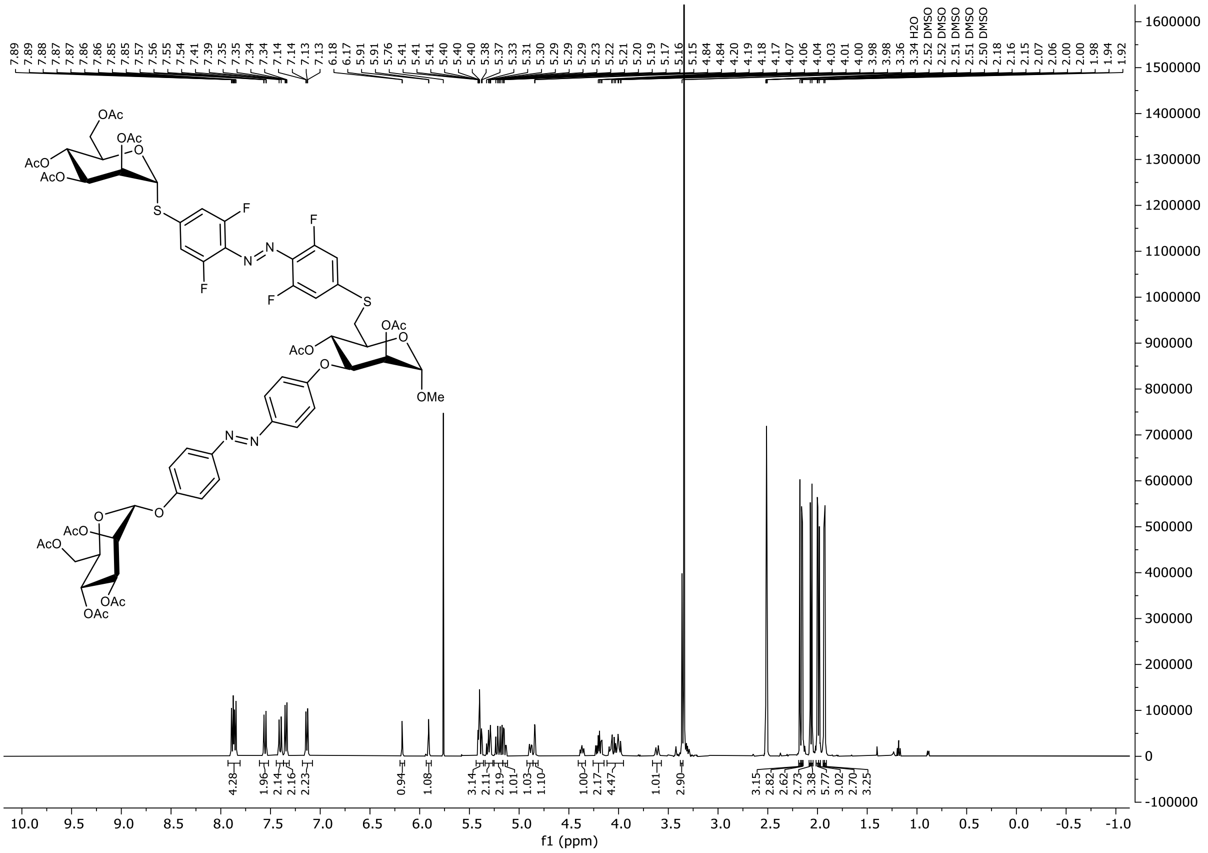

Figure S48: ^{1}H NMR (DMSO-d_6, 500 MHz) of compound **12**.

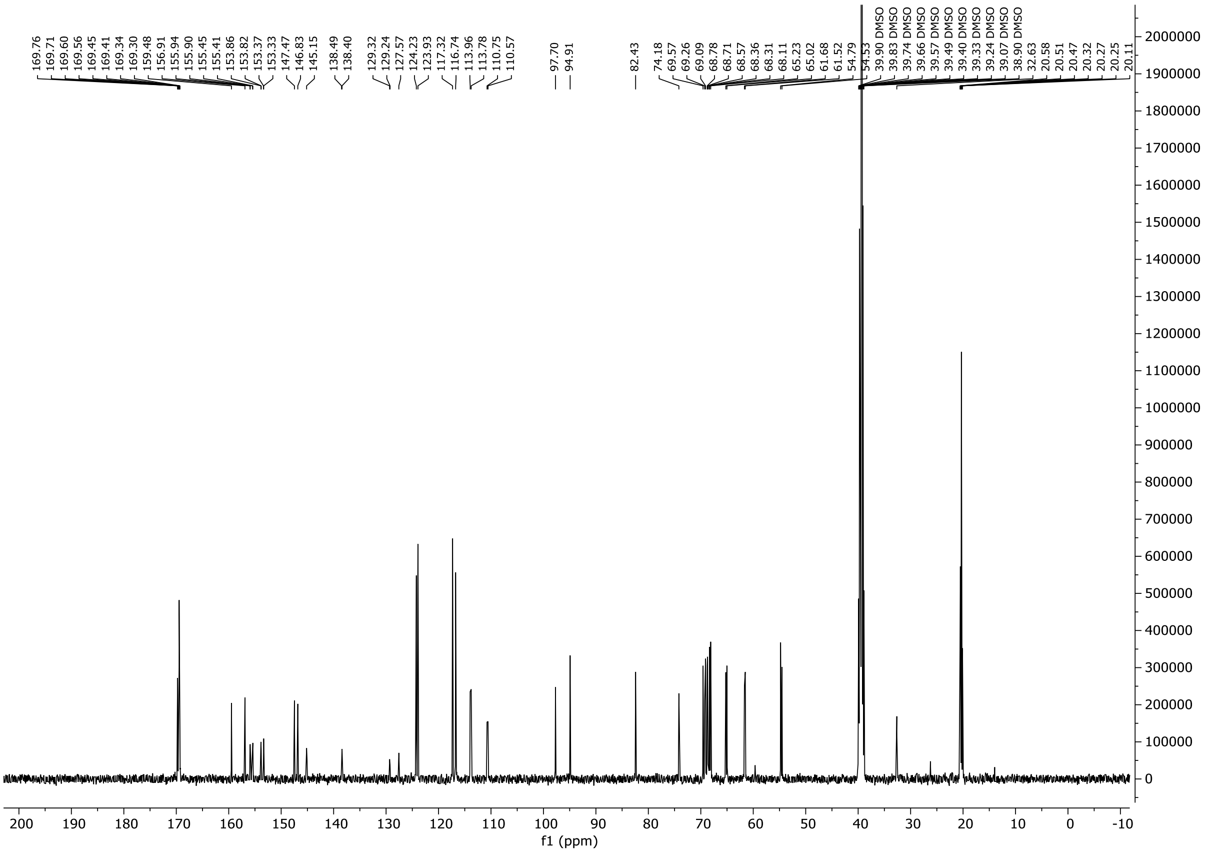

Figure S49: ^{13}C NMR (DMSO-d_6, 126 MHz) of compound **12**.

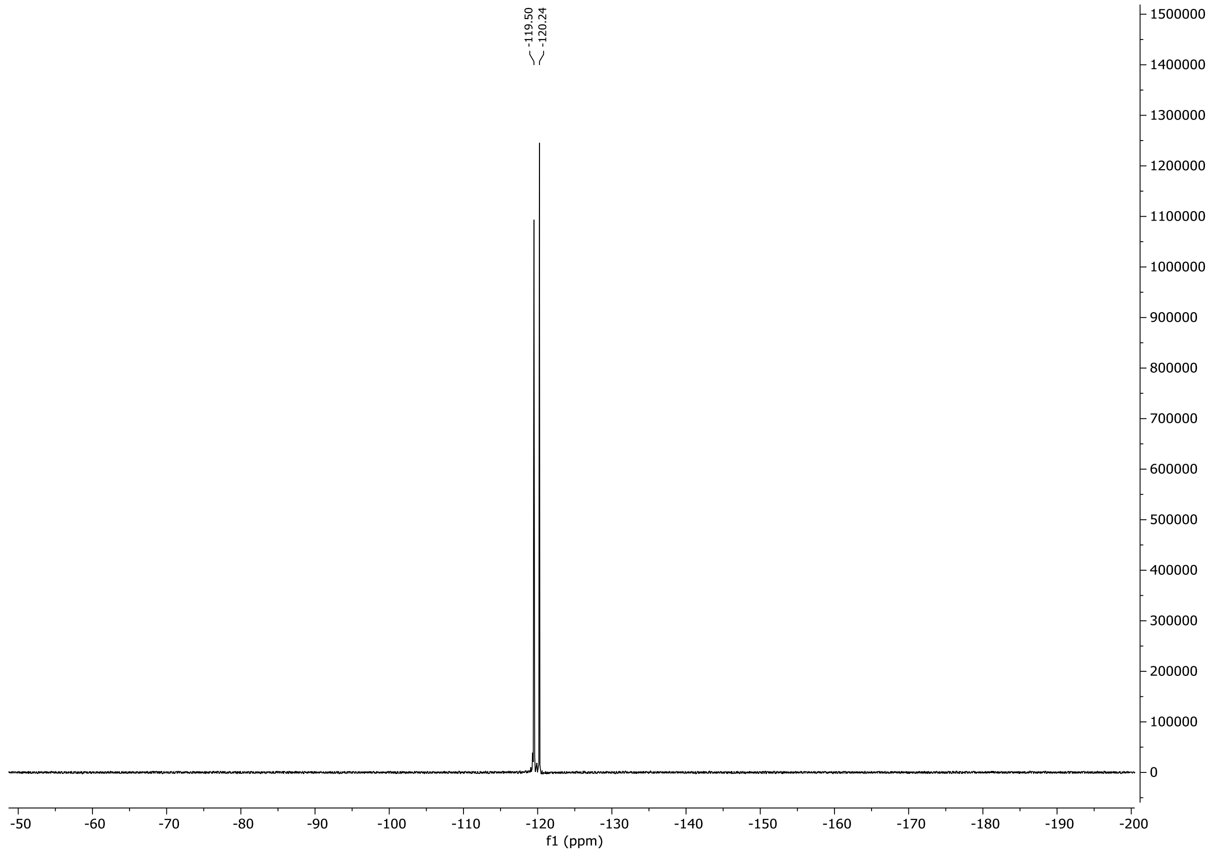

Figure S50: ^{19}F NMR (DMSO-d_6, 471 MHz) of compound **12**.

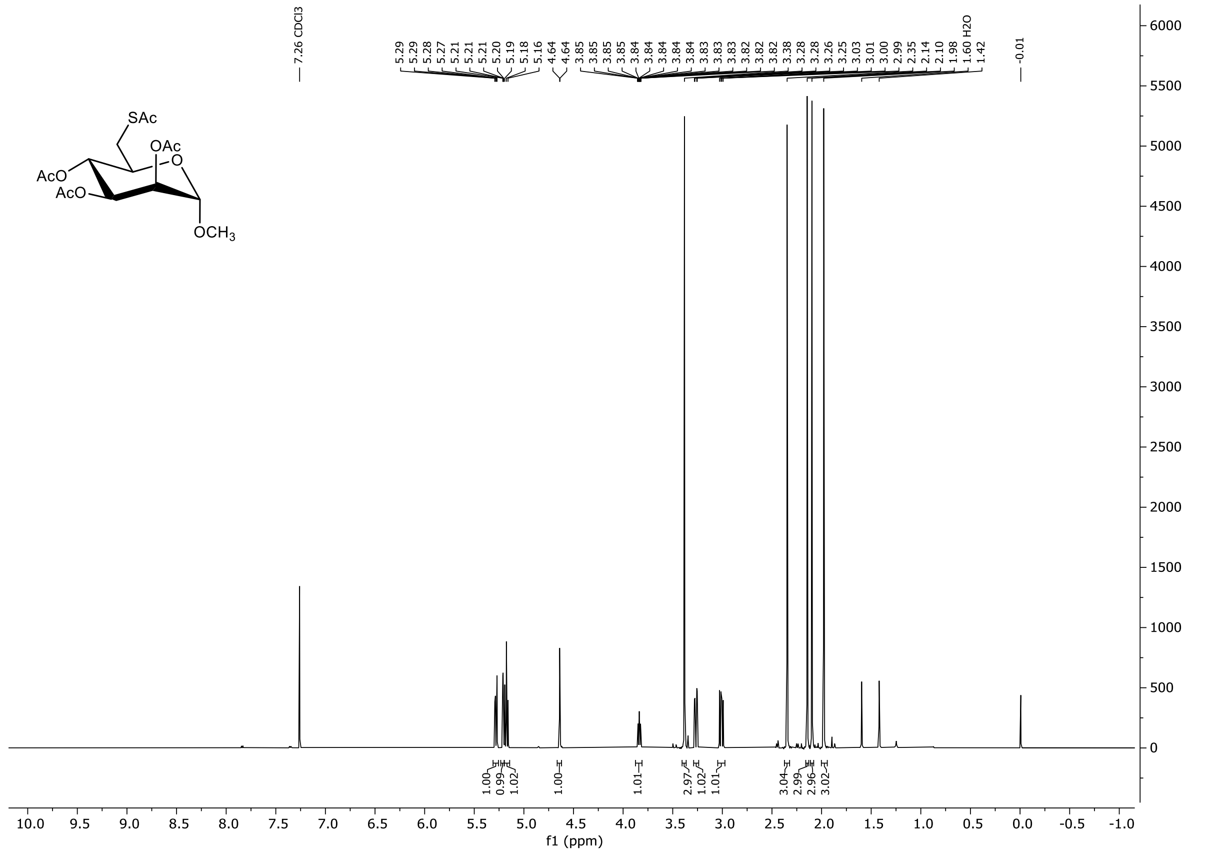

Figure S51: ^{1}H NMR ($CDCl_3$, 600 MHz) of compound **13**.

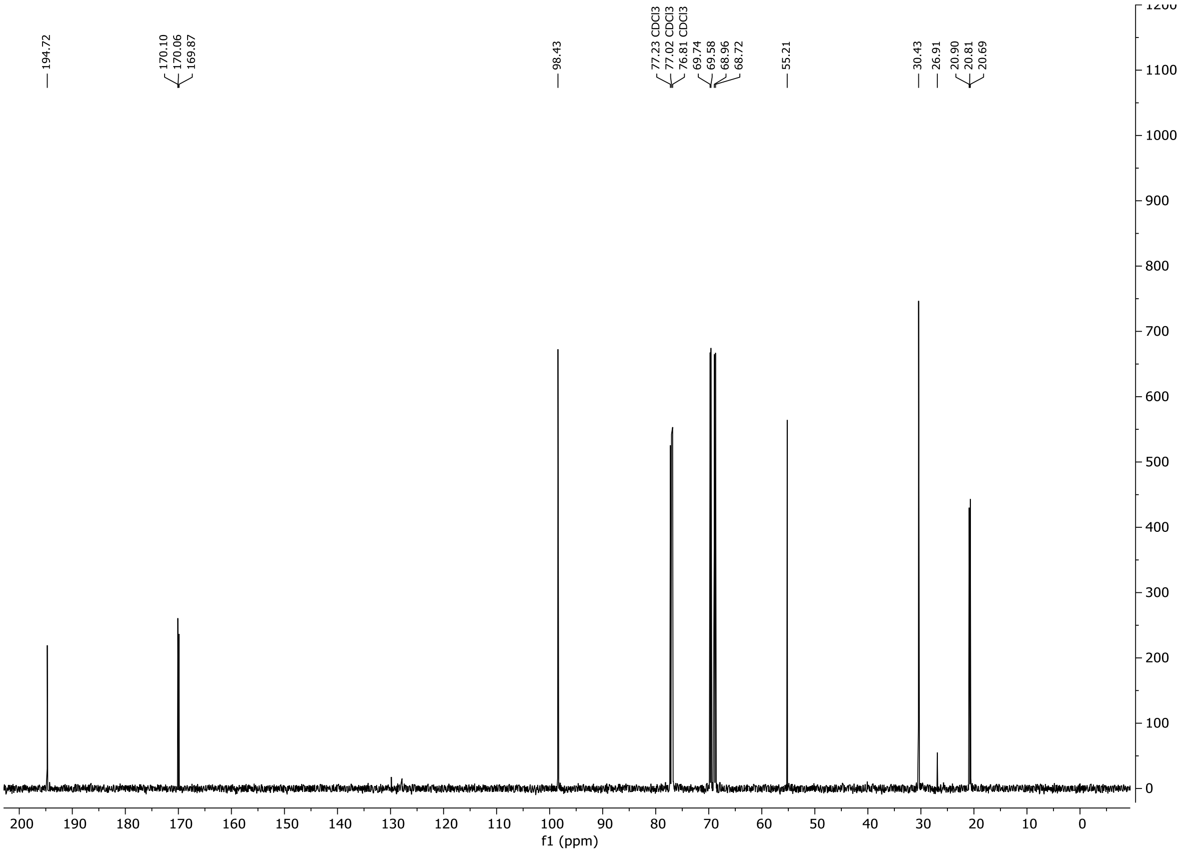

Figure S52: ^{13}C NMR ($CDCl_3$, 151 MHz) of compound **13**.

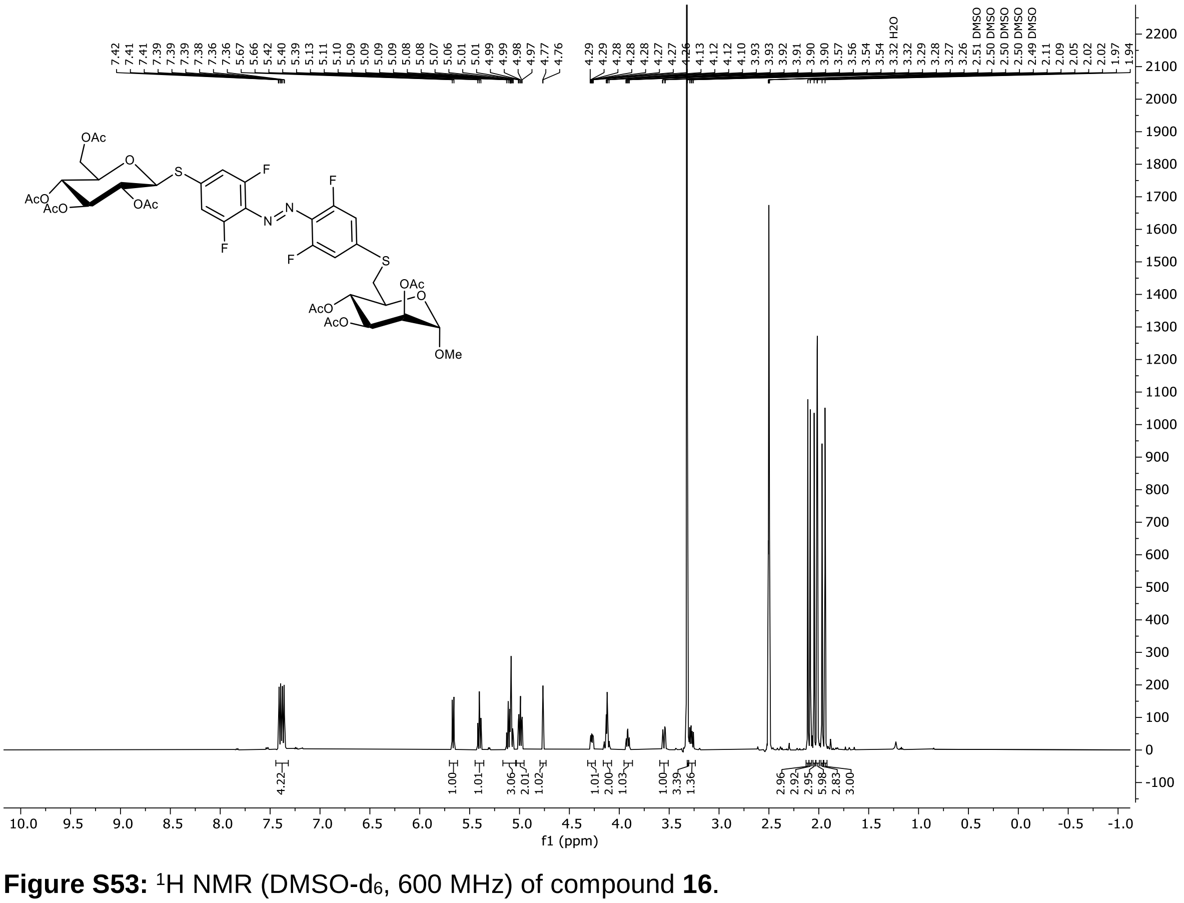

Figure S53: ^{1}H NMR (DMSO-d_6, 600 MHz) of compound **16**.

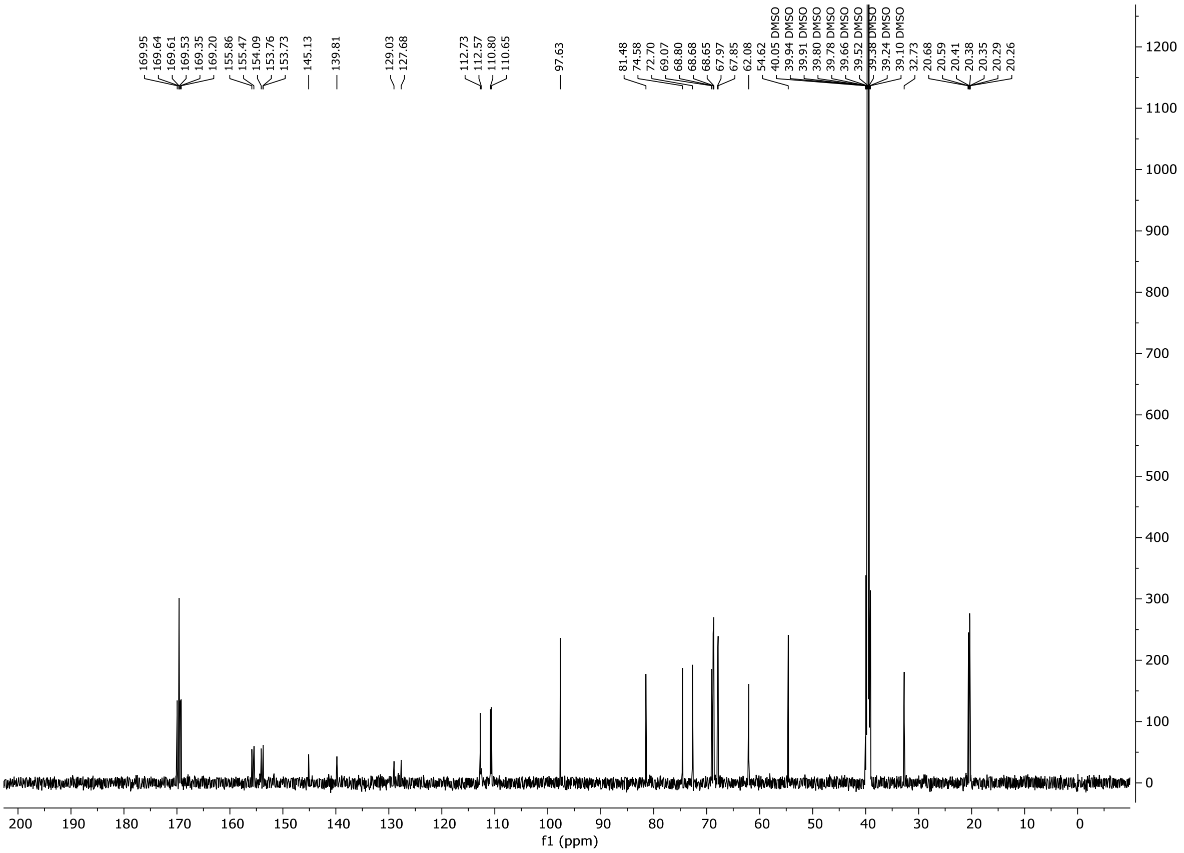

Figure S54: ^{13}C NMR (DMSO-d_6, 151 MHz) of compound **16**.

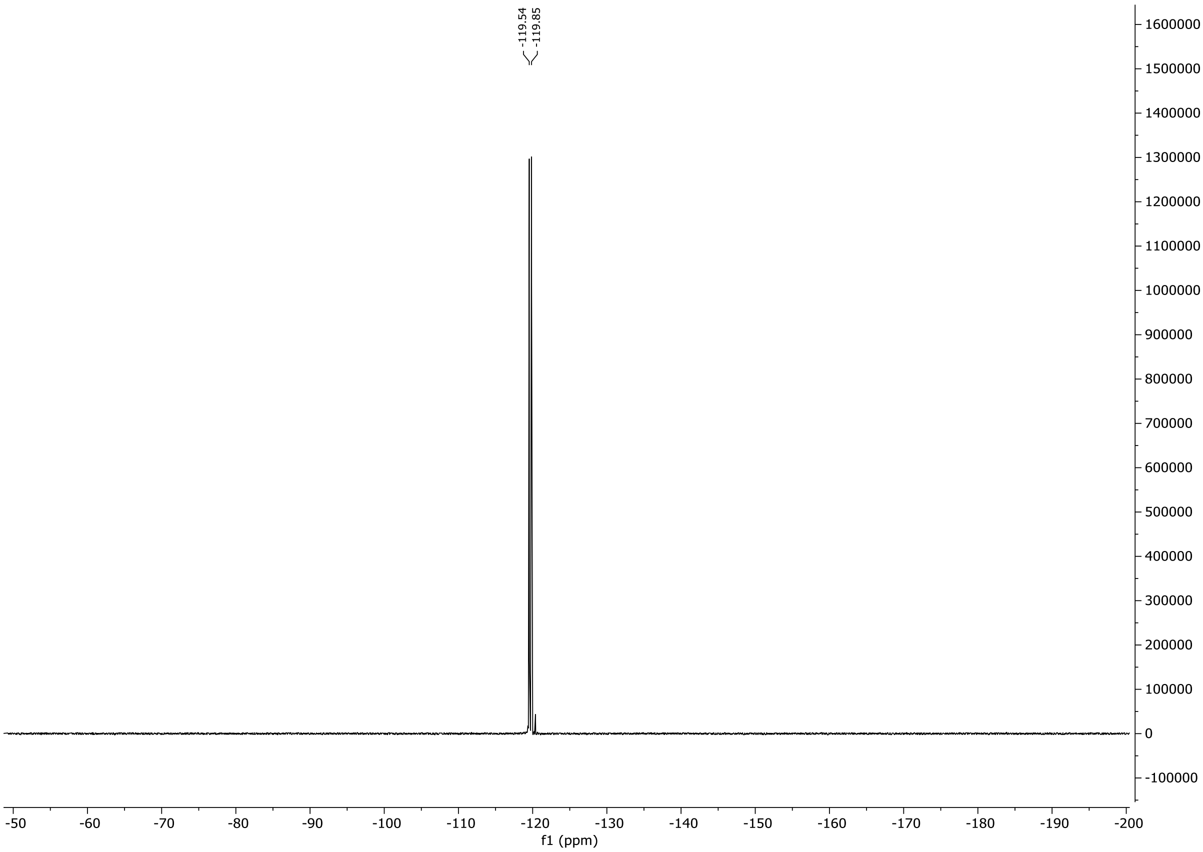

Figure S55: ^{19}F NMR (methanol-d_4, 471 MHz) of compound **16**.

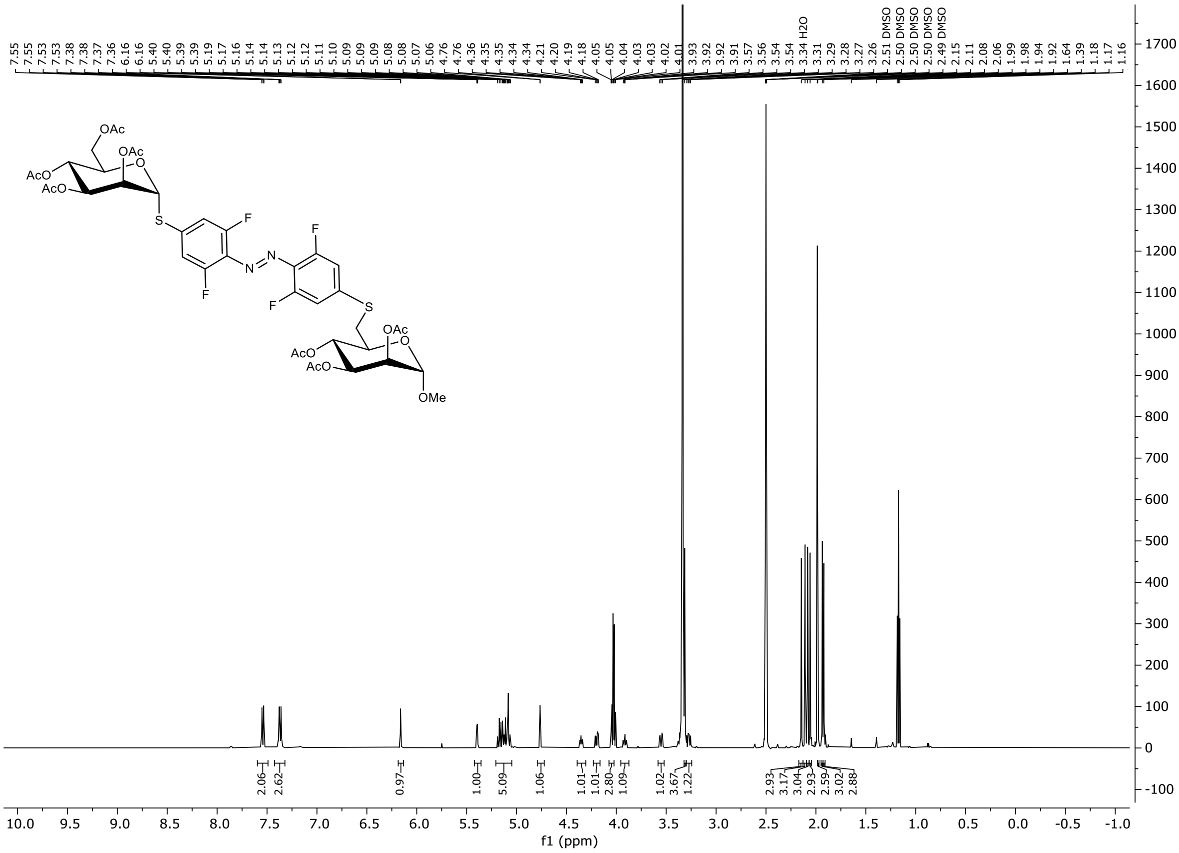

Figure S56: ^{1}H NMR (DMSO-d_6, 600 MHz) of compound **17**.

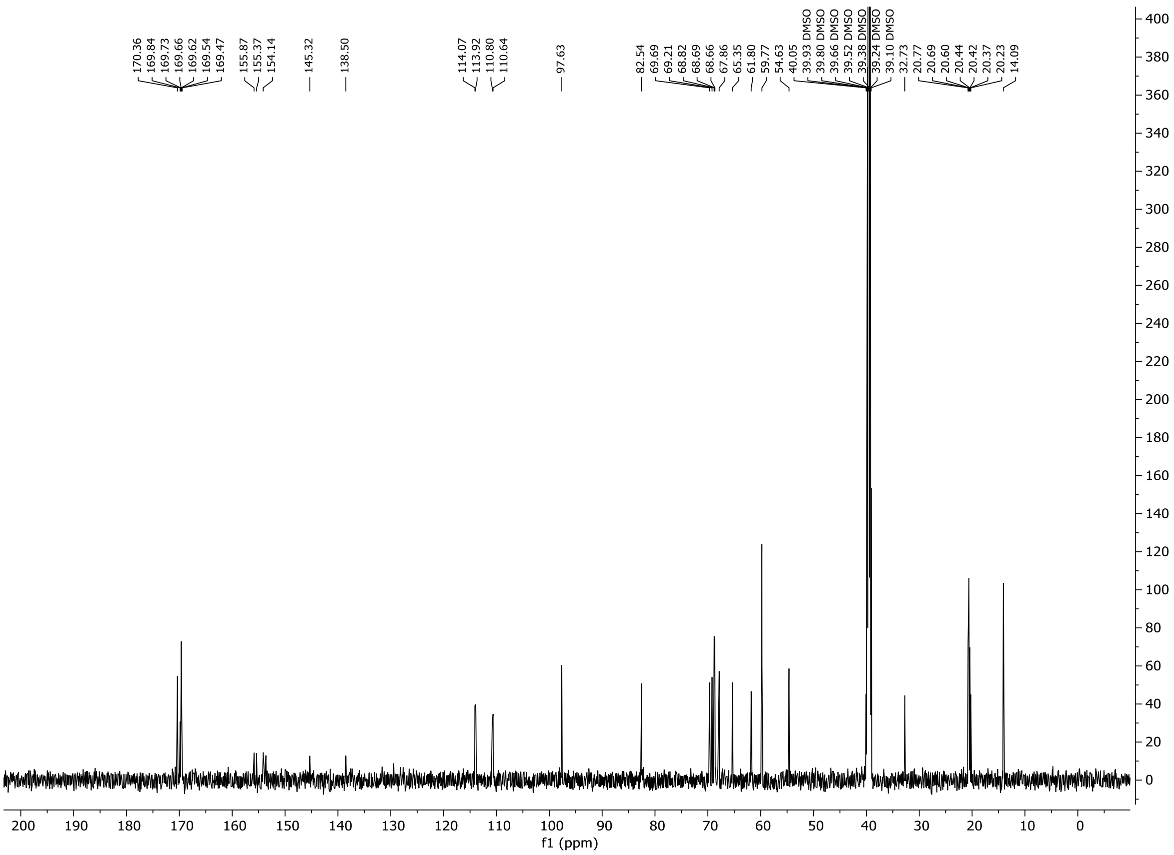

Figure S57: ^{13}C NMR (DMSO-d_6, 151 MHz) of compound **17**.

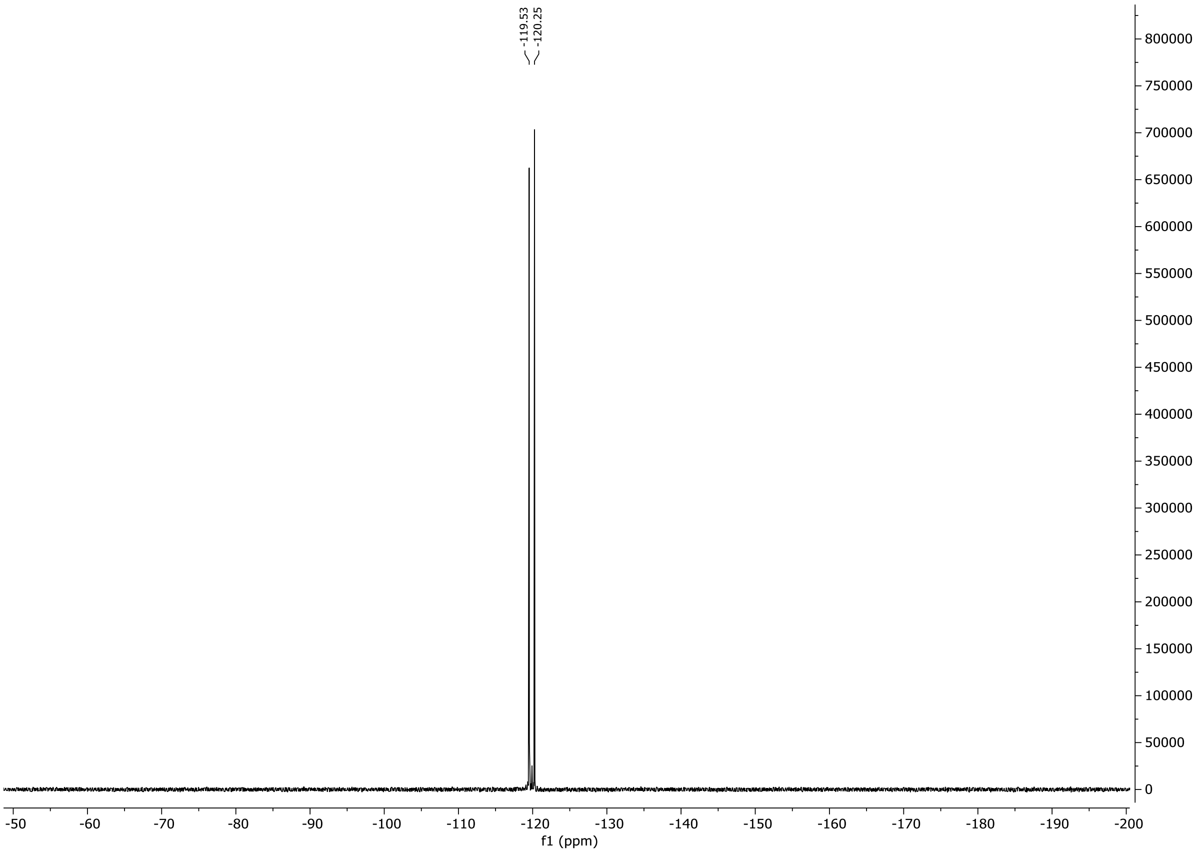

Figure S58: ^{19}F NMR (DMSO-d6, 471 MHz) of compound **17**.

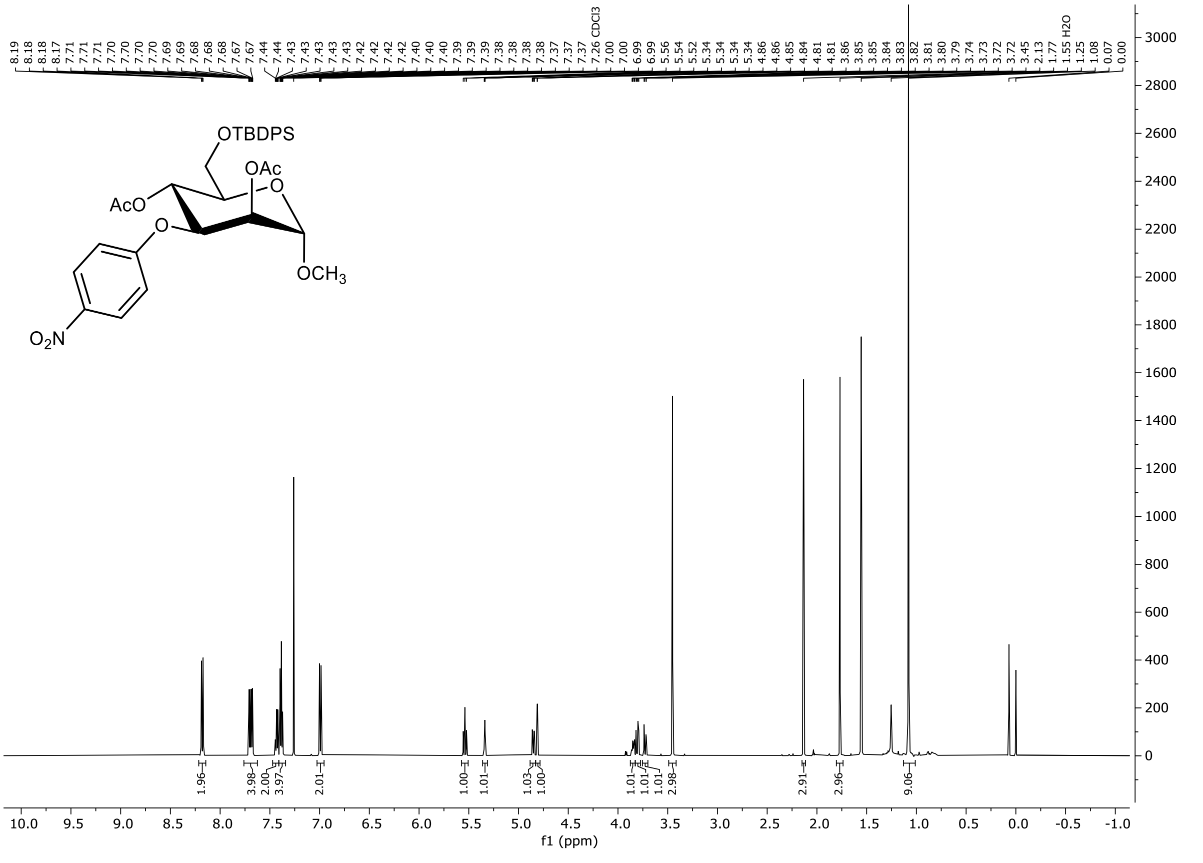

Figure S59: ^{1}H NMR ($CDCl_3$, 600 MHz) of compound **S2**.

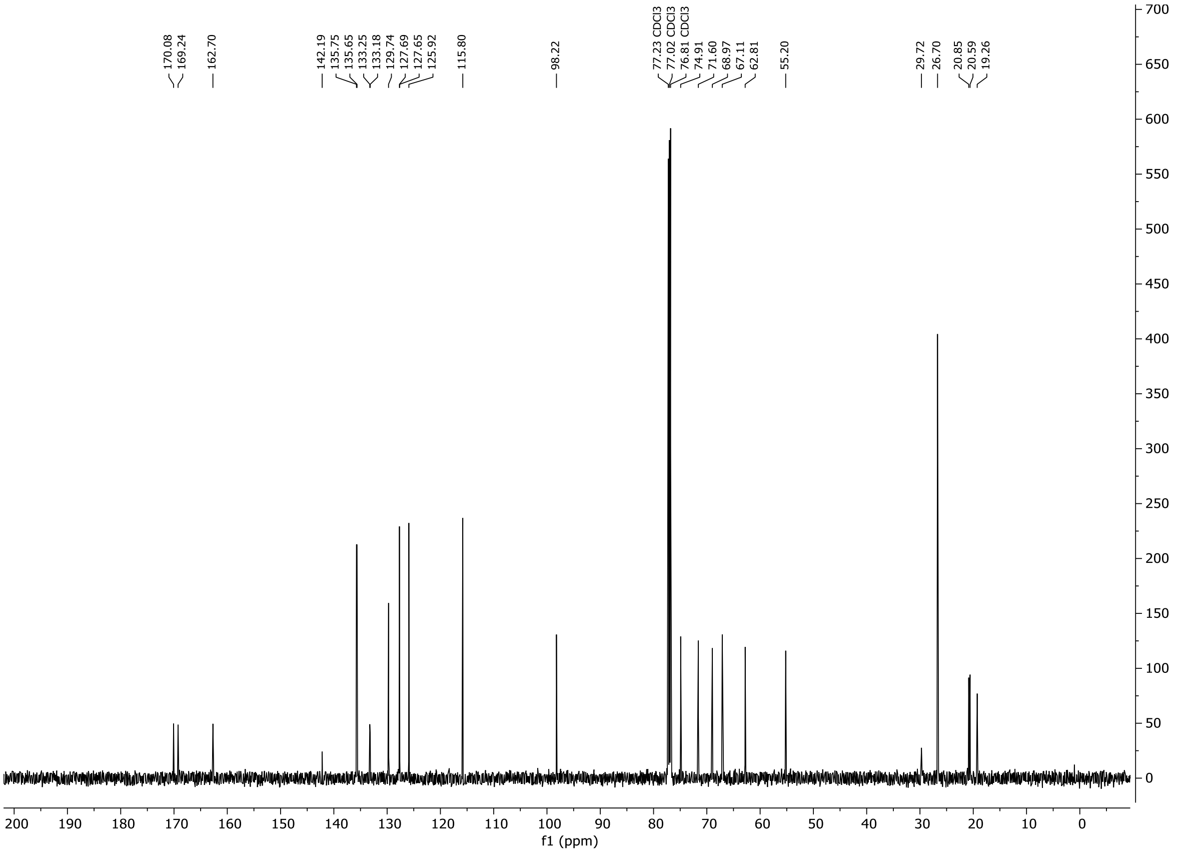

Figure S60: ^{13}C NMR ($CDCl_3$, 151 MHz) of compound **S2**.

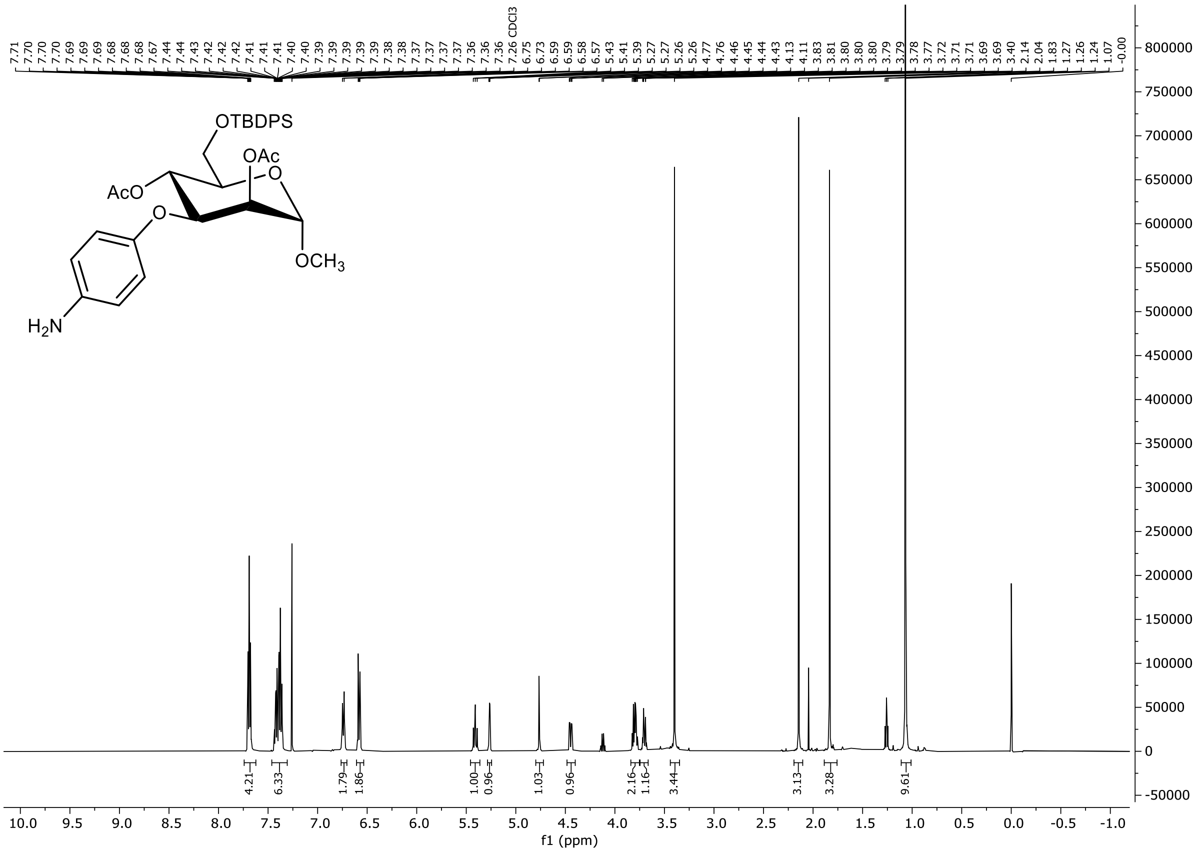

Figure S61: ^{1}H NMR ($CDCl_3$, 500 MHz) of compound **S3**.

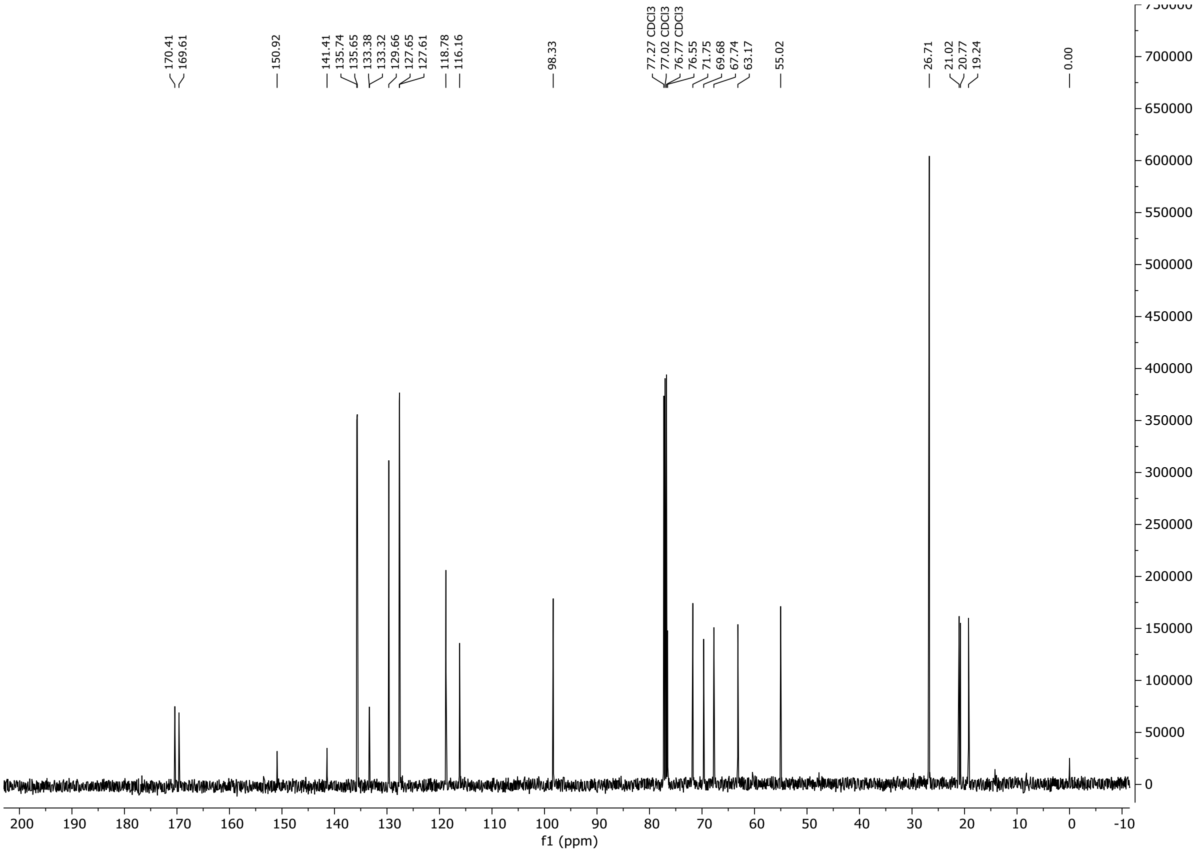

Figure S62: ^{13}C NMR ($CDCl_3$, 126 MHz) of compound **S3**.

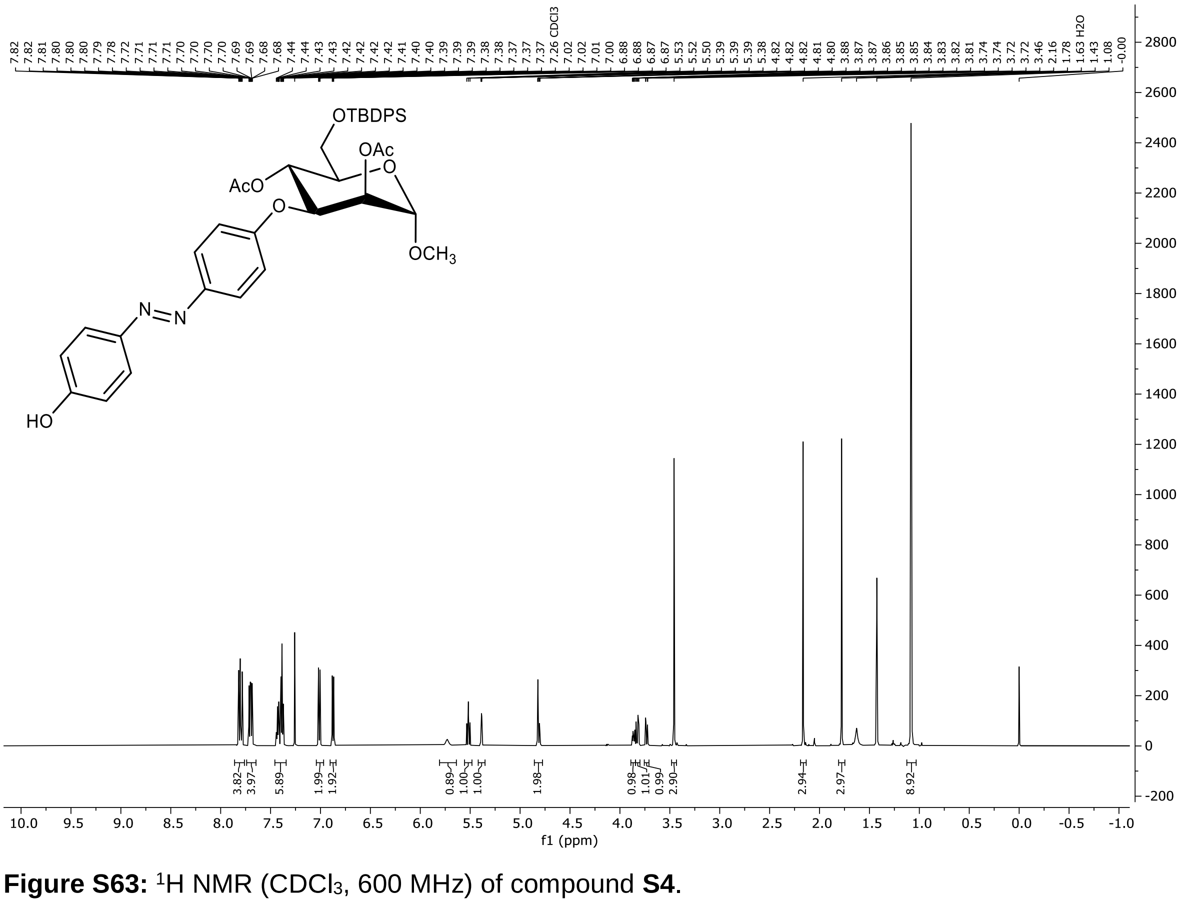

Figure S63: ^{1}H NMR ($CDCl_3$, 600 MHz) of compound **S4**.

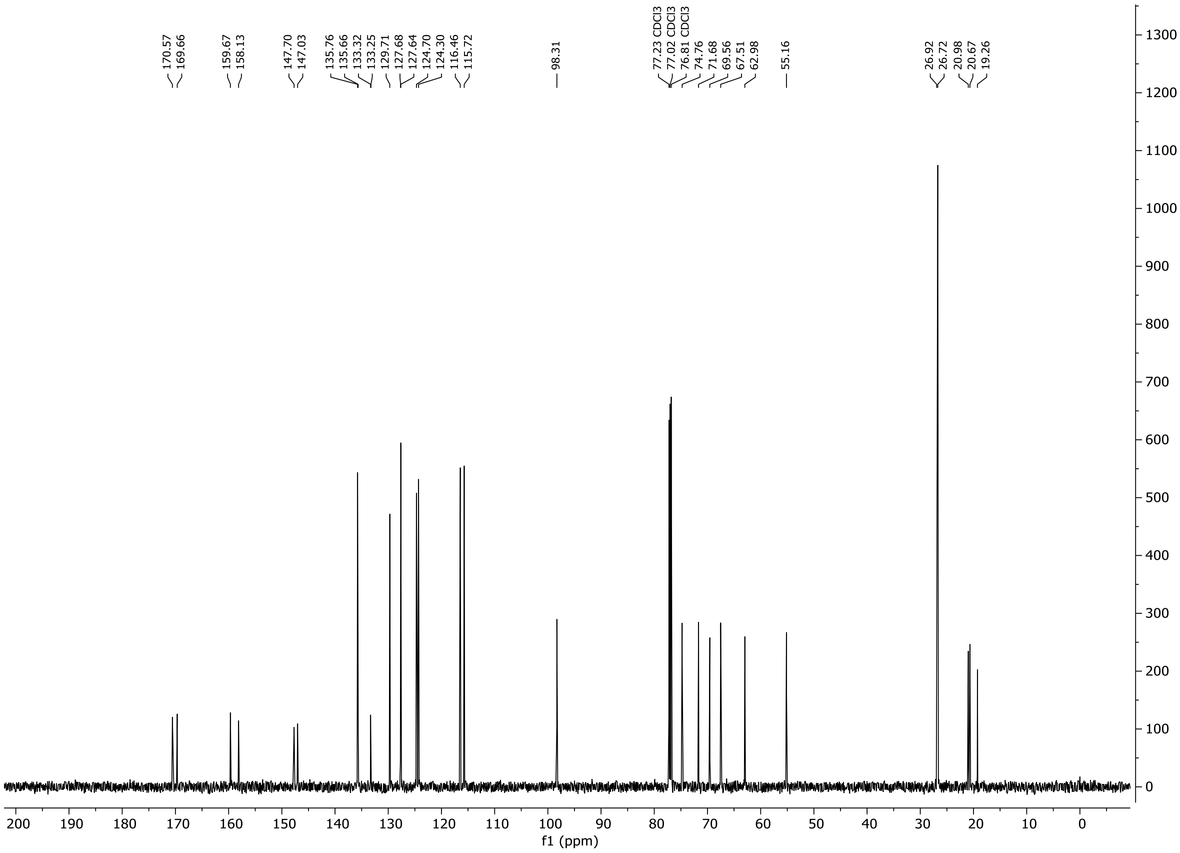

Figure S64: ^{13}C NMR ($CDCl_3$, 151 MHz) of compound **S4**.

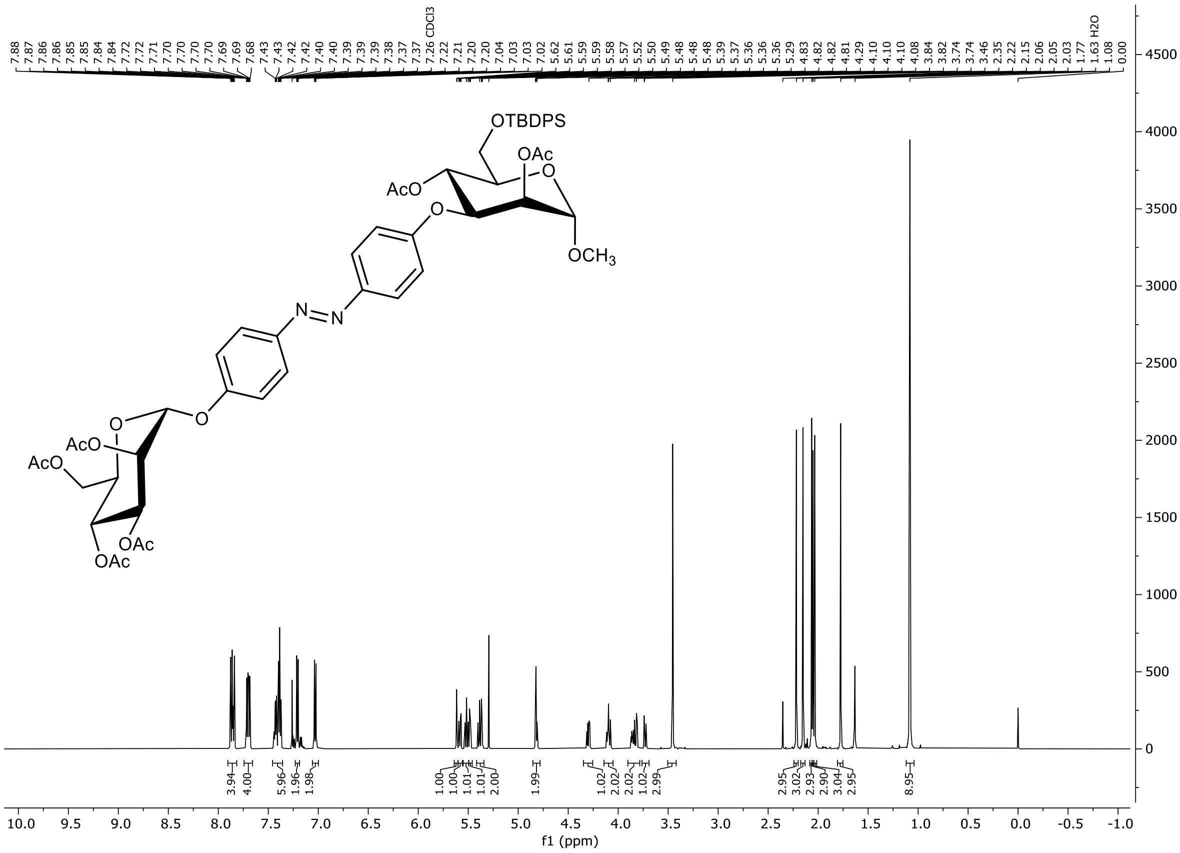

Figure S65: ^{1}H NMR ($CDCl_3$, 600 MHz) of compound **S5**.

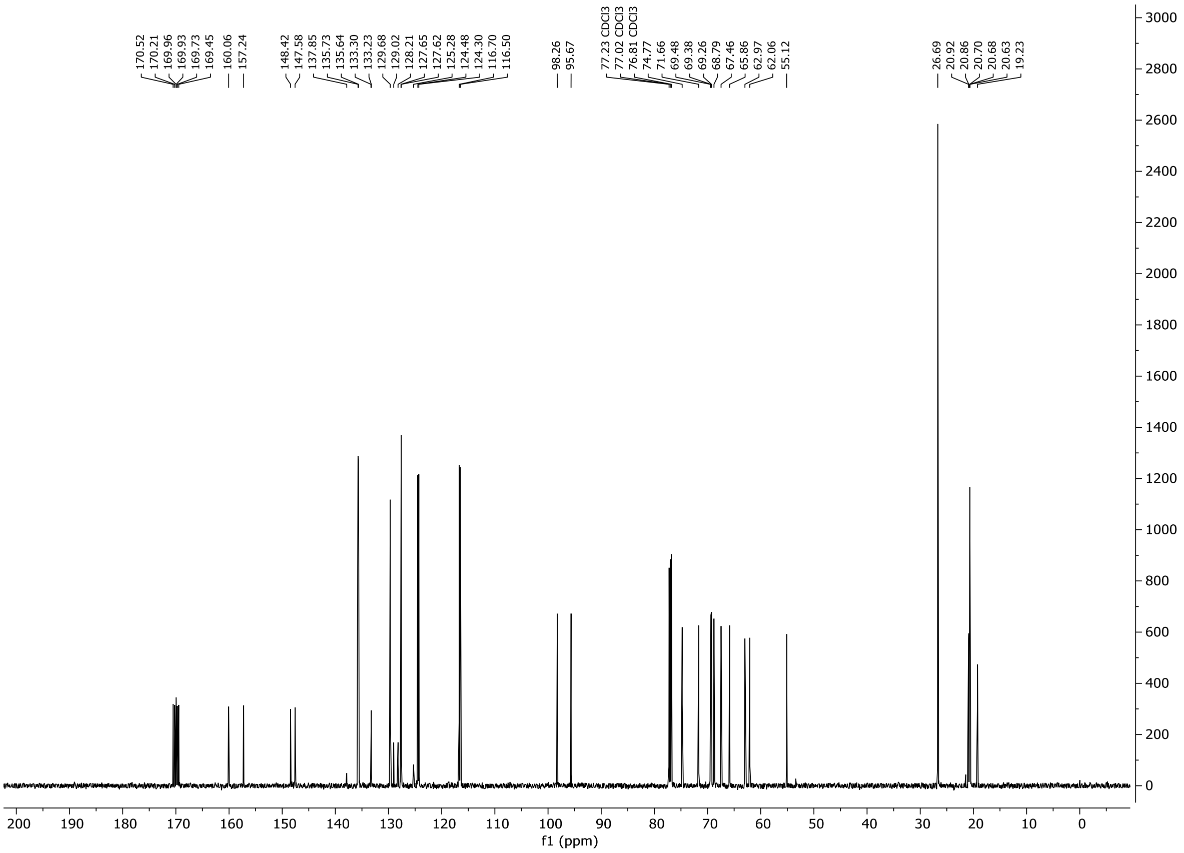

Figure S66: ^{13}C NMR ($CDCl_3$, 151 MHz) of compound **S5**.

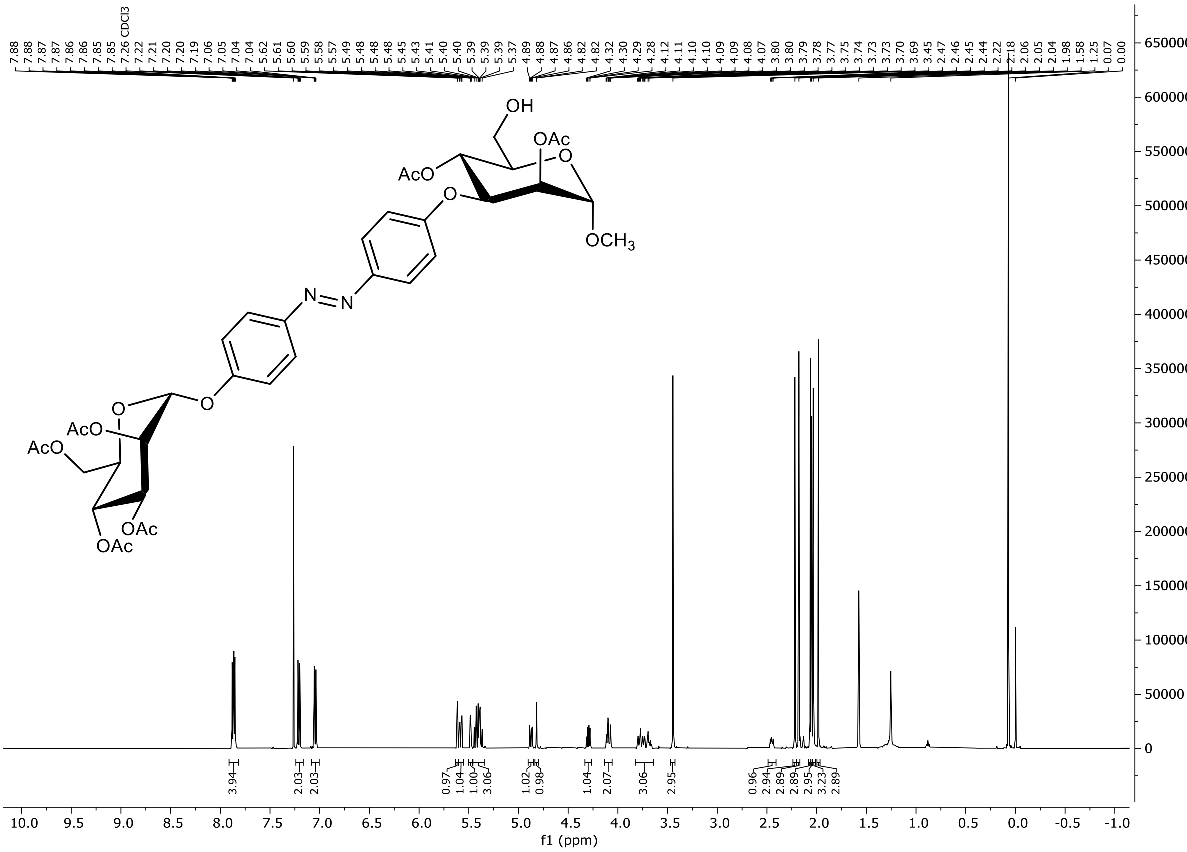

Figure S67: ^{1}H NMR ($CDCl_3$, 500 MHz) of compound **S6**.

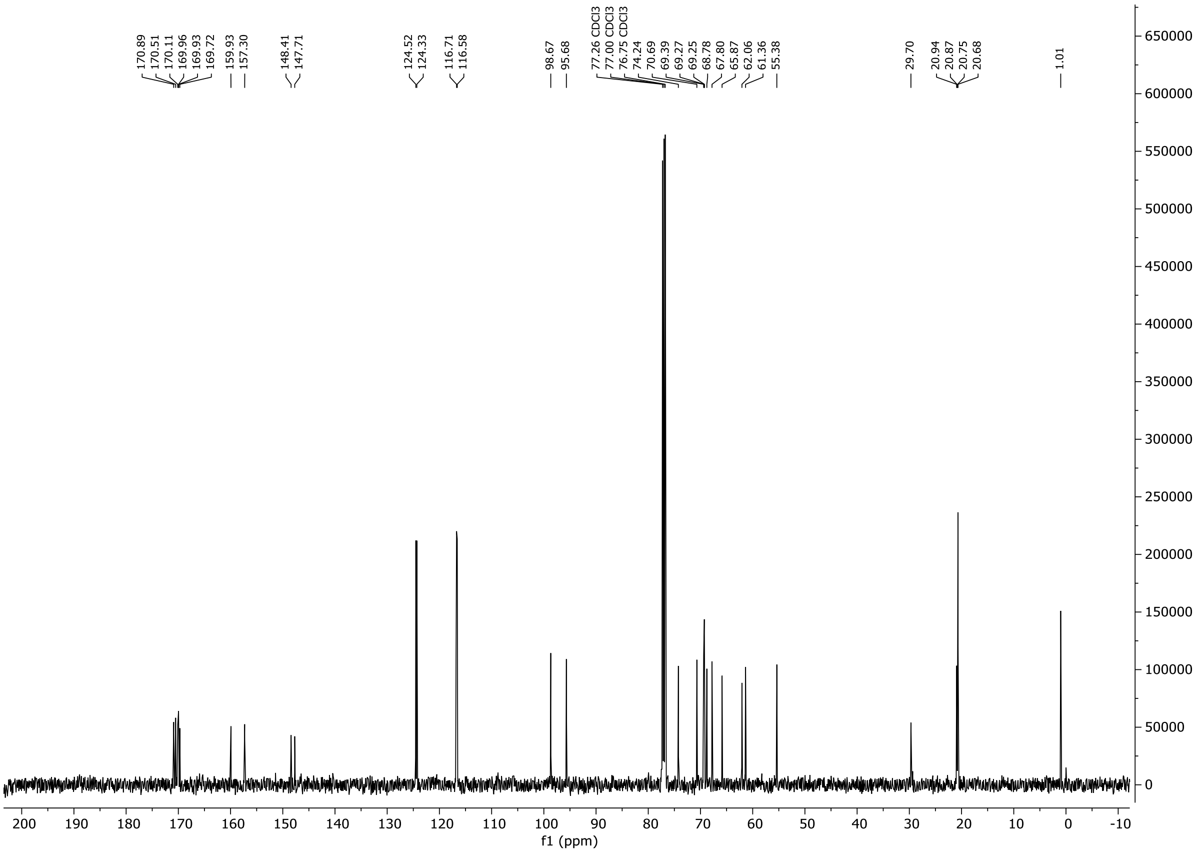

Figure S68: ^{13}C NMR ($CDCl_3$, 126 MHz) of compound **S6**.

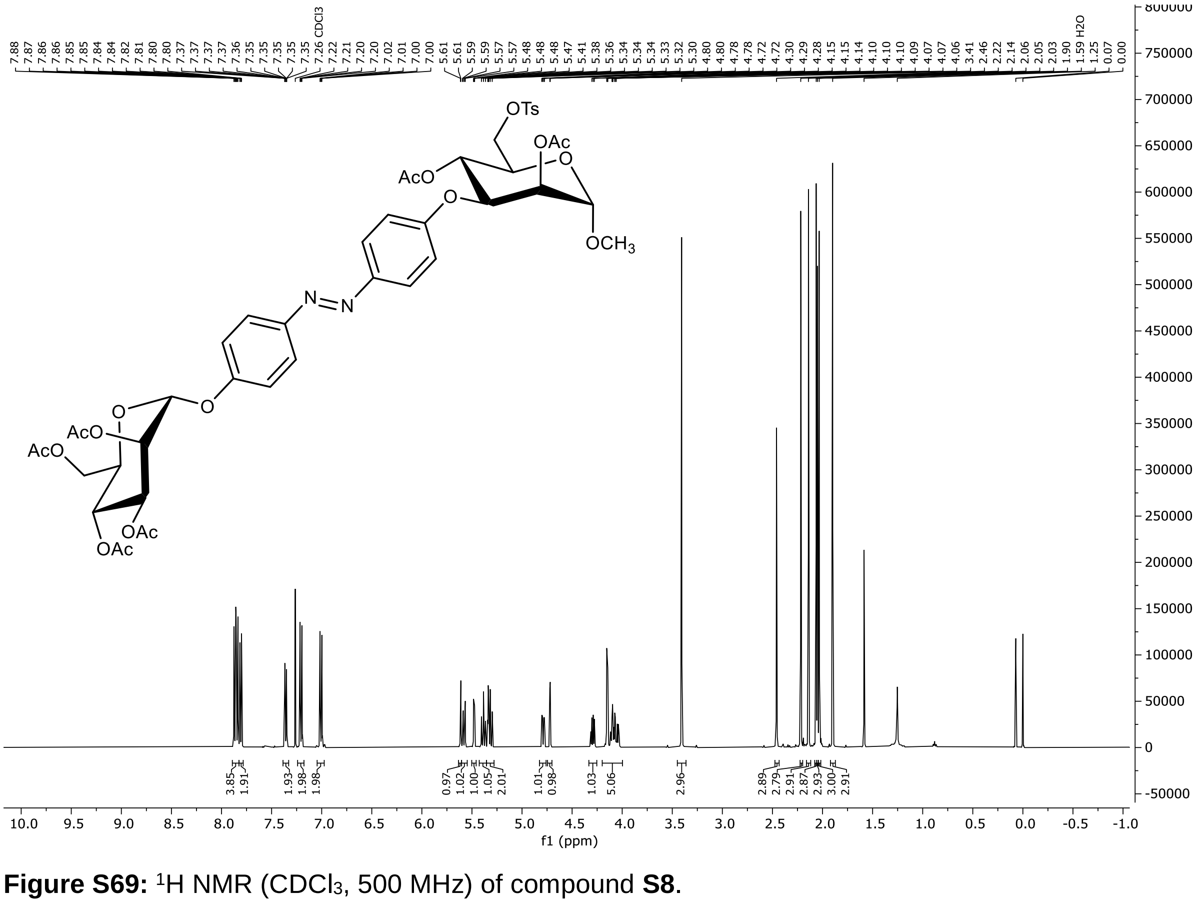

Figure S69: ^{1}H NMR ($CDCl_3$, 500 MHz) of compound **S8**.

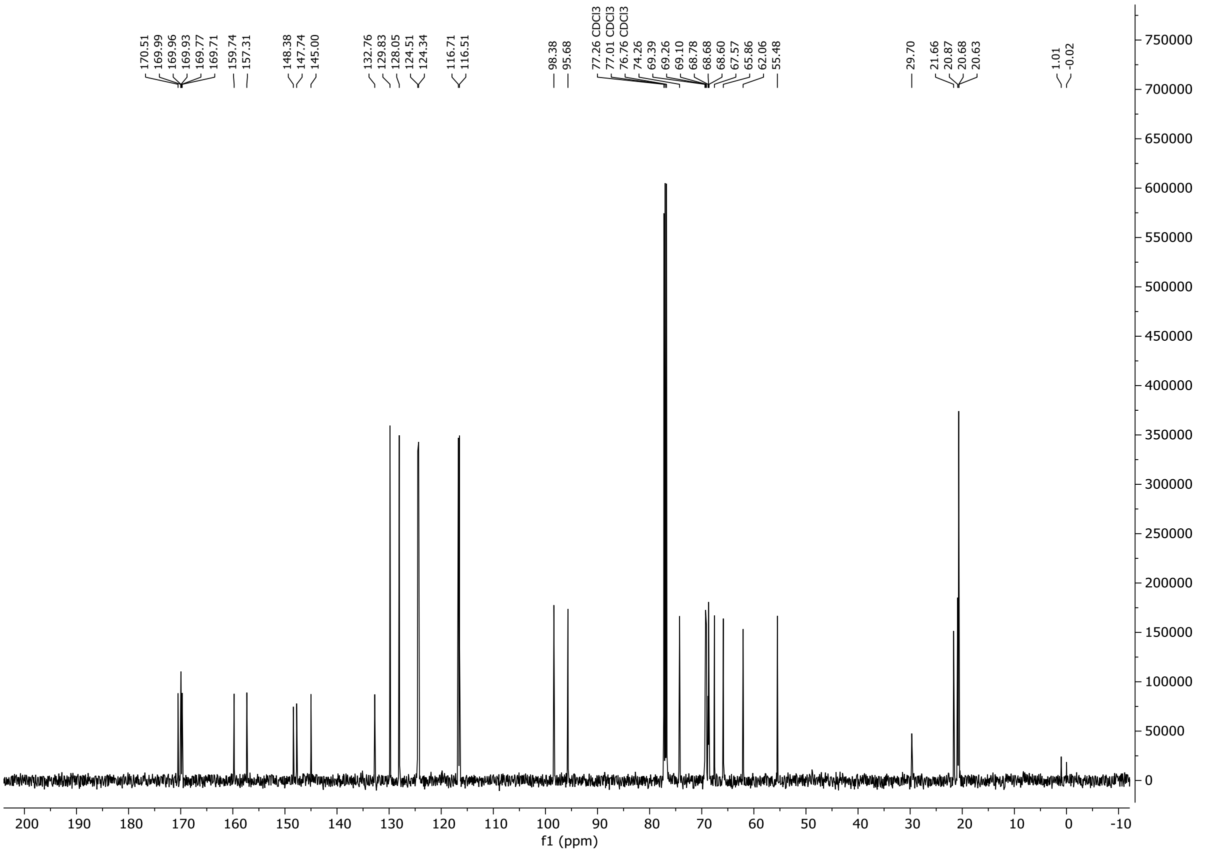

Figure S70: ^{13}C NMR ($CDCl_3$, 126 MHz) of compound **S8**.

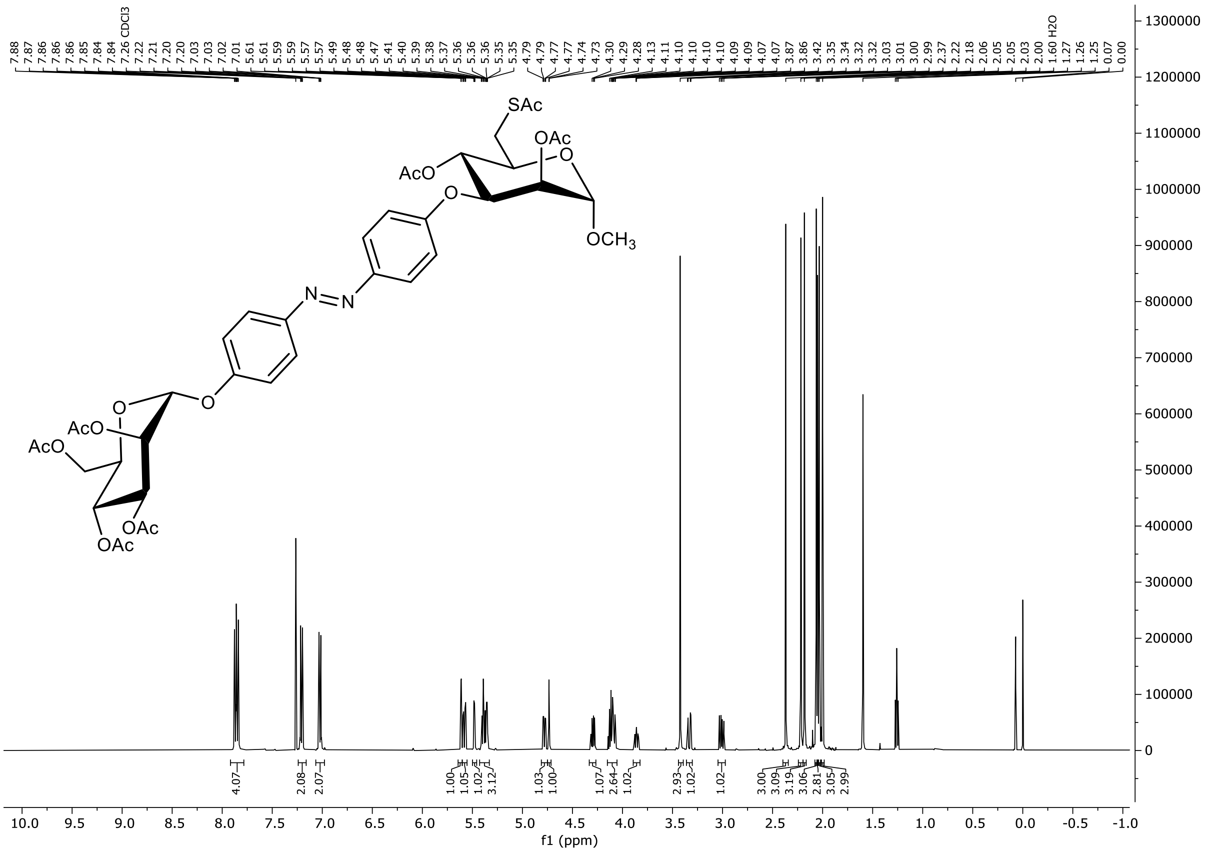

Figure S71: ^{1}H NMR ($CDCl_3$, 500 MHz) of compound **S9**.

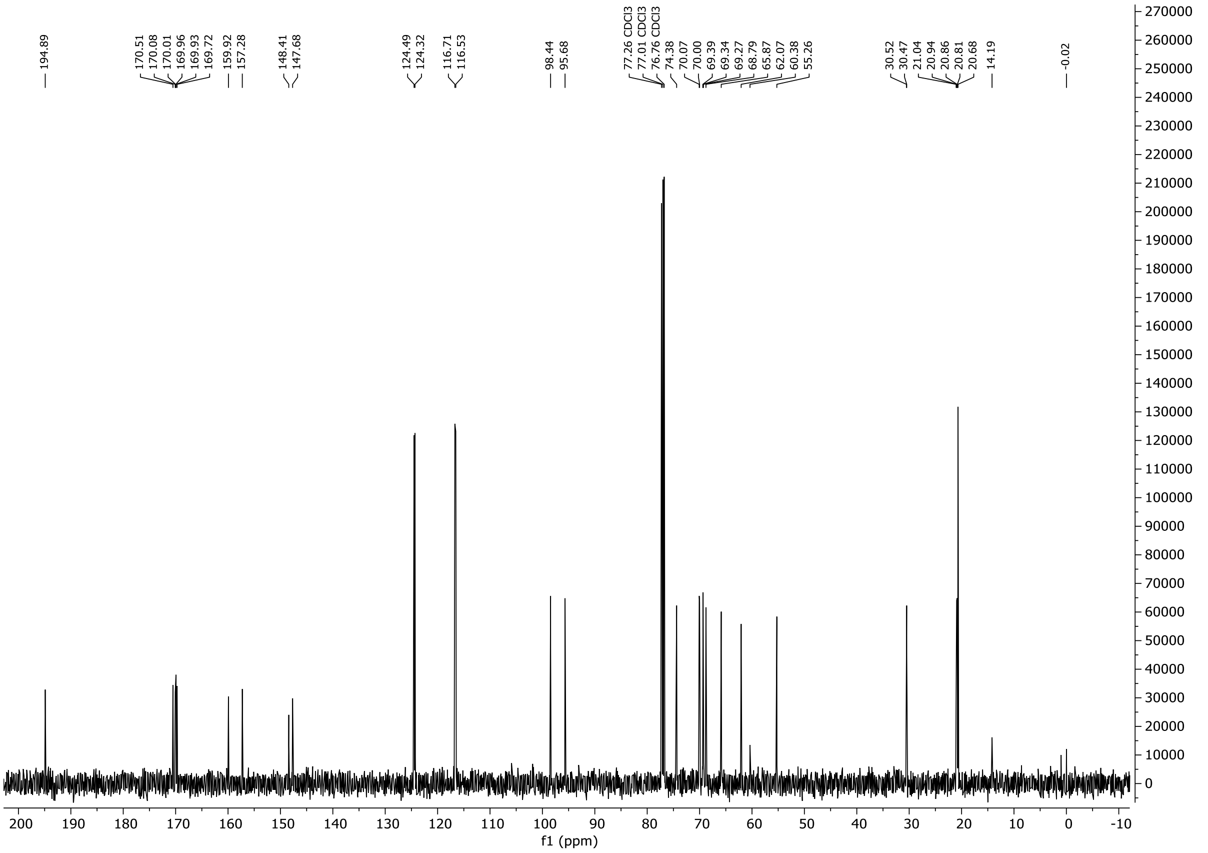

Figure S72: ^{13}C NMR ($CDCl_3$, 126 MHz) of compound **S9**.

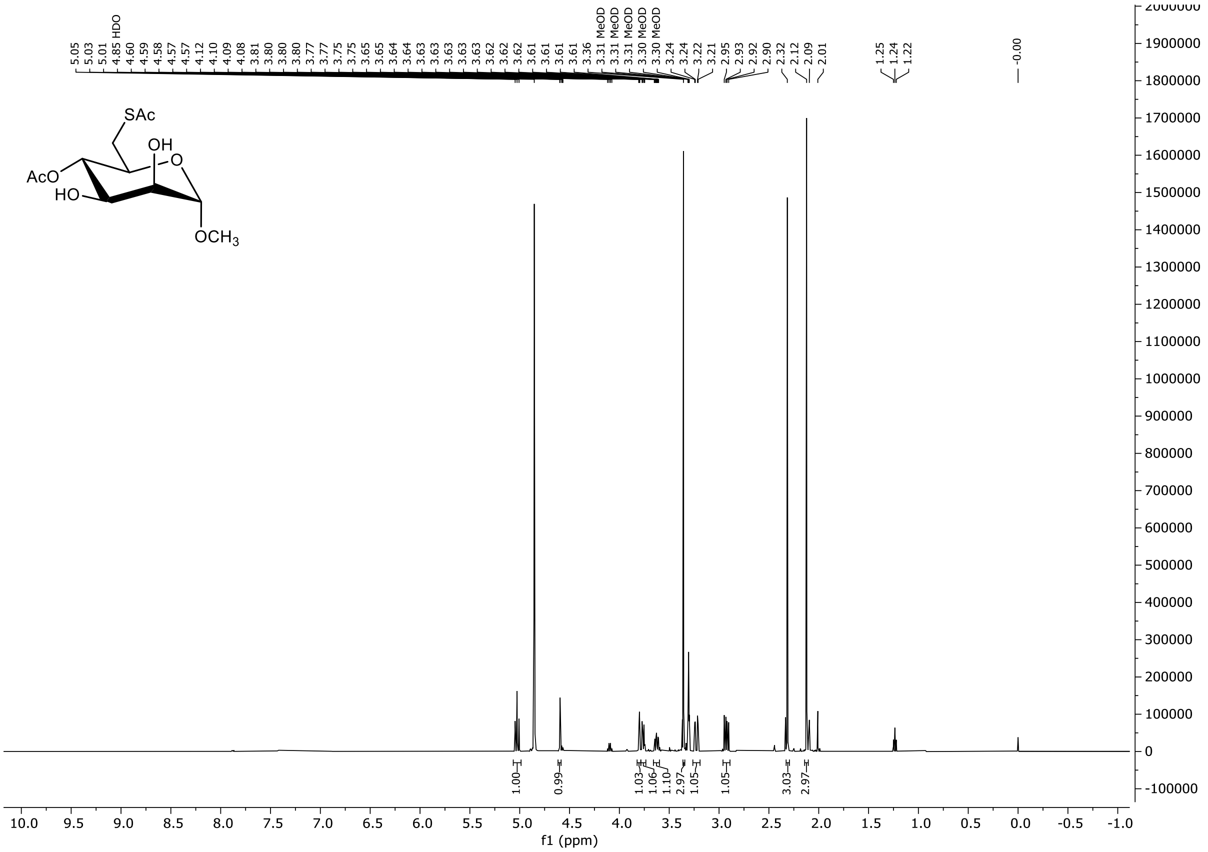

Figure S73: ^{1}H NMR ($CDCl_3$, 500 MHz) of compound **S11**.

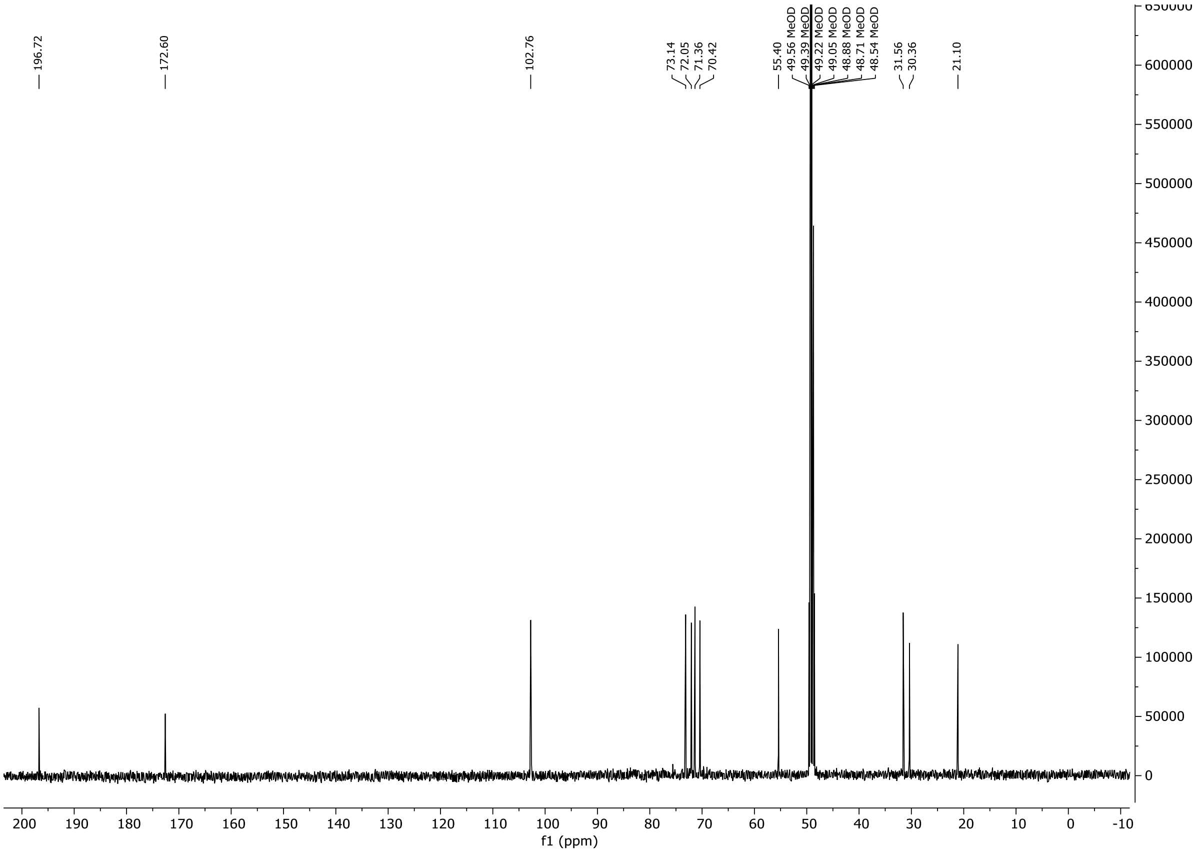

Figure S74: ^{13}C NMR ($CDCl_3$, 126 MHz) of compound **S11**.

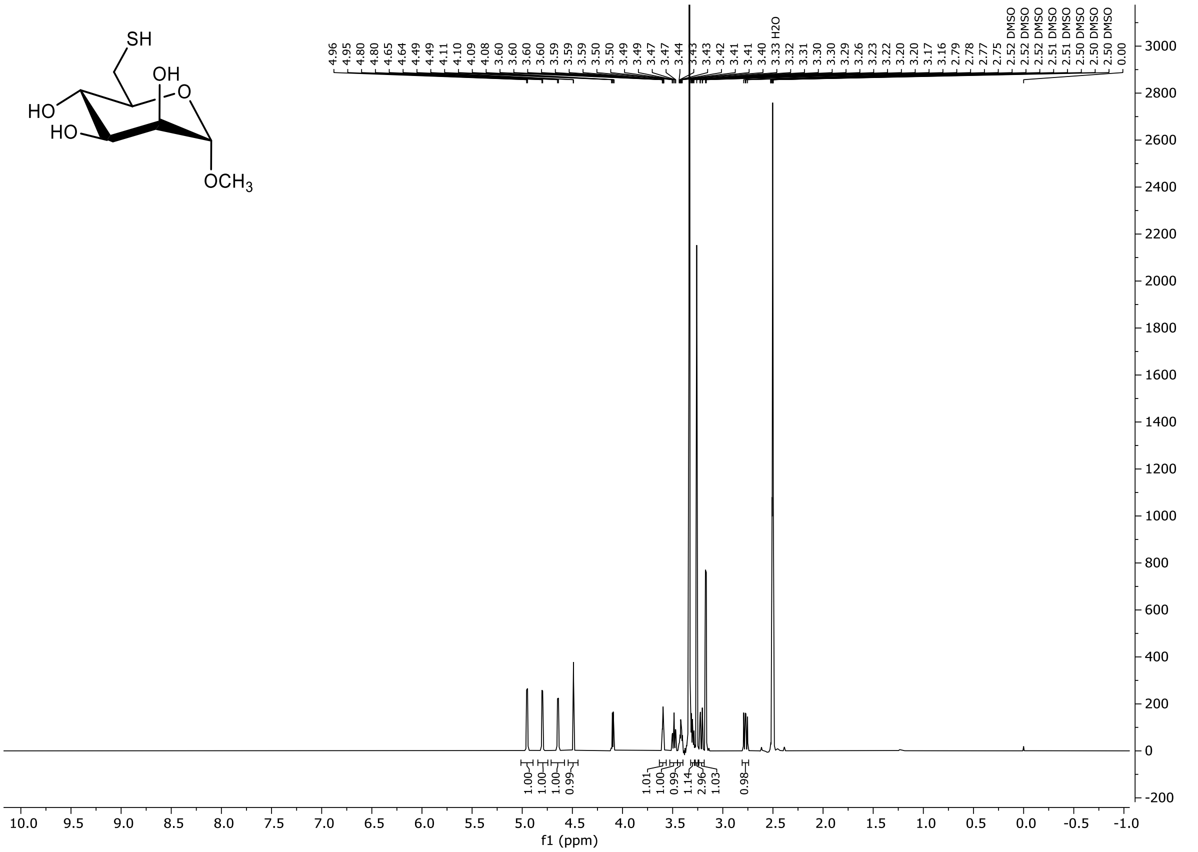

Figure S75: ^{1}H NMR (DMSO-d_6, 600 MHz) of compound **S12**.

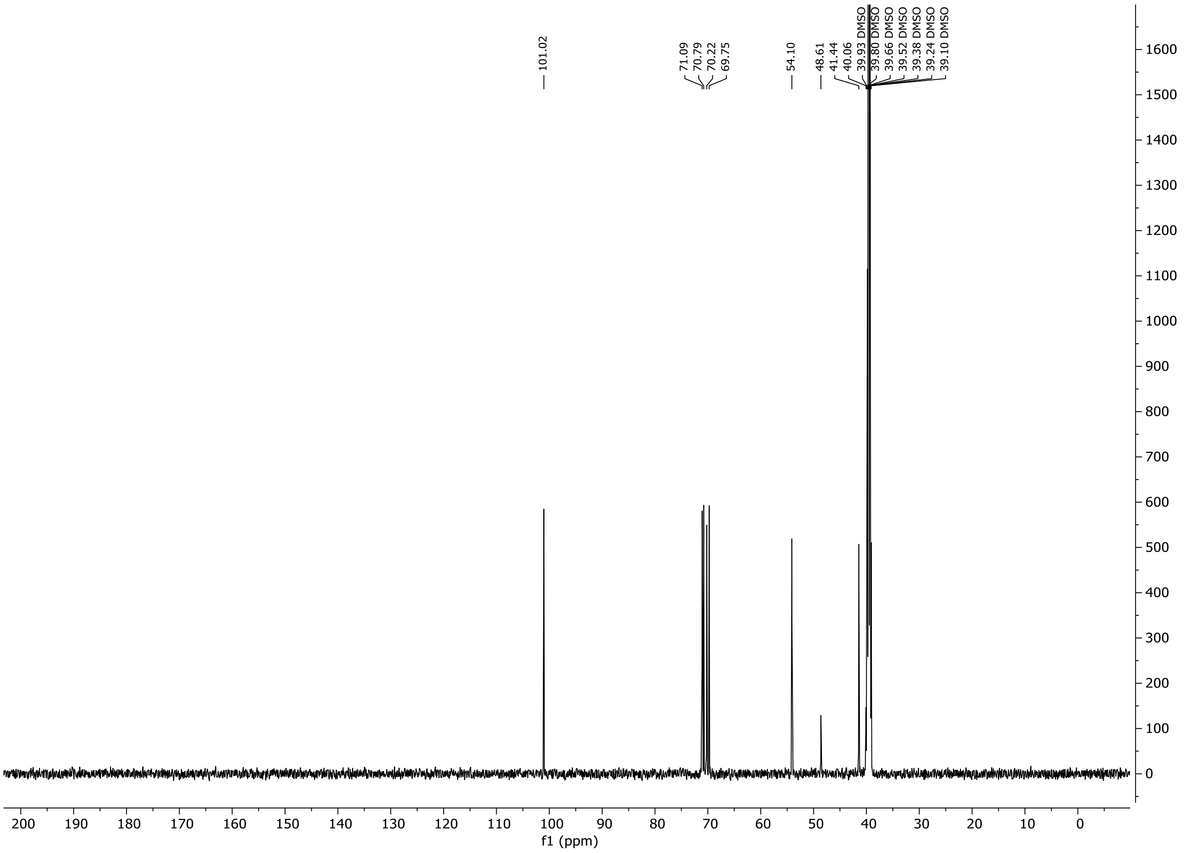

Figure S76: ^{13}C NMR (DMSO-d_6, 151 MHz) of compound **S12**.

6 References

1. Jung, K.-H.; Hoch, M.; Schmidt, R. R. *Liebigs Ann. Chem.* **1989,** *1989* (11), 1099–1106. DOI: 10.1002/jlac.198919890276.
2. A. John, A.; Lin, Q. *J. Org. Chem.* **2017,** *82* (18), 9873–9876. DOI: 10.1021/acs.joc.7b01530.
3. L. M. Friedrich, C. Lütjohann, B. Hartke, T. K. Lindhorst. *Chem. Eur. J.* **2024**, e202403117. DOI: 10.1002/chem.202403117.
4. Pei, Z.; Larsson, R.; Aastrup, T.; Anderson, H.; Lehn, J.-M.; Ramström, O. *Biosens. Bioelectron.* **2006,** *22* (1), 42–48. DOI: 10.1016/j.bios.2005.11.024.
5. Kondo, Y. *Carbohydr. Res.* **1986,** *154* (1), 305–309. DOI: 10.1016/S0008-6215(00)90046-6.
6. Sivapriya, K.; Chandrasekaran, S. *Carbohydr. Res.* **2006,** *341* (13), 2204–2210. DOI: 10.1016/j.carres.2006.06.001.
7. Ge, J.-T.; Zhou, L.; Zhao, F.-L.; Dong, H. *J. Org. Chem.* **2017,** *82* (23), 12613–12623. DOI: 10.1021/acs.joc.7b02367.
8. Berry, J.; Lindhorst, T. K.; Despras, G. *Chem. Eur. J.* **2022,** *28* (39), e202200354. DOI: 10.1002/chem.202200354.
9. Friedrich, L. M.; Hartke, B.; Lindhorst, T. K. *Chem. Eur. J.* **2024**, e202402125. DOI: 10.1002/chem.202402125.
10. Hartmann, M.; Horst, A. K.; Klemm, P.; Lindhorst, T. K. *Chem. Commun.* **2010,** *46* (2), 330–332. DOI: 10.1039/b922525k.
11. Reisner, A.; Haagensen, J. A. J.; Schembri, M. A.; Zechner, E. L.; Molin, S. *Mol. Microbiol.* **2003,** *48* (4), 933–946. DOI: 10.1046/j.1365-2958.2003.03490.x.
12. Schrödinger Release 2022-2: *Maestro*, Schrödinger, LLC, New York, NY, **2022**.
13. Schrödinger Release 2024-1: *Maestro*, Schrödinger, LLC, New York, NY, **2024**.
14. Schrödinger Release 2022-2: *Protein Preparation Wizard; Epic*, New York, NY,2022. Impact, Schrödinger, New York, NY, **2022**.
15. Schrödinger Release 2022-2: *Glide*, Schrödinger, LLC, New York, NY, **2022**.
16. Schrödinger Release 2024-1: *Glide*, Schrödinger, LLC, New York, NY, **2024**.
17. Hung, C.-S.; Bouckaert, J.; Hung, D.; Pinkner, J.; Widberg, C.; DeFusco, A.; Auguste, C. G.; Strouse, R.; Langermann, S.; Waksman, G.; Hultgren, S. J. *Mol. Microbiol.* **2002,** *44* (4), 903–915. DOI: 10.1046/j.1365-2958.2002.02915.x.
18. Bouckaert, J.; Berglund, J.; Schembri, M.; Genst, E. de; Cools, L.; Wuhrer, M.; Hung, C.-S.; Pinkner, J.; Slättegård, R.; Zavialov, A.; Choudhury, D.; Langermann, S.; Hultgren, S. J.; Wyns, L.; Klemm, P.; Oscarson, S.; Knight, S. D.; Greve, H. de. *Mol. Microbiol.* **2005,** *55* (2), 441–455. DOI: 10.1111/j.1365-2958.2004.04415.x.
19. Lu, C.; Wu, C.; Ghoreishi, D.; Chen, W.; Wang, L.; Damm, W.; Ross, G. A.; Dahlgren, M. K.; Russell, E.; Bargen, C. D. von; Abel, R.; Friesner, R. A.; Harder, E. D. *J. Chem. Theory Comput.* **2021,** *17* (7), 4291–4300. DOI: 10.1021/acs.jctc.1c00302.
20. Roos, K.; Wu, C.; Damm, W.; Reboul, M.; Stevenson, J. M.; Lu, C.; Dahlgren, M. K.; Mondal, S.; Chen, W.; Wang, L.; Abel, R.; Friesner, R. A.; Harder, E. D. *J. Chem. Theory Comput.* **2019,** *15* (3), 1863–1874. DOI: 10.1021/acs.jctc.8b01026.
21. Schrödinger Release 2024-1: *Prime*, Schrödinger, LLC, New York, NY, **2024**.

22. Schrödinger Release 2024-1: *Induced Fit Docking protocol*, Prime, Schrödinger, LLC, New York, NY,2024. Glide, Schrödinger, LLC, New York, NY, **2024**.

23. Banks, J. L.; Beard, H. S.; Cao, Y.; Cho, A. E.; Damm, W.; Farid, R.; Felts, A. K.; Halgren, T. A.; Mainz, D. T.; Maple, J. R.; Murphy, R.; Philipp, D. M.; Repasky, M. P.; Zhang, L. Y.; Berne, B. J.; Friesner, R. A.; Gallicchio, E.; Levy, R. M. *J. Chem. Theory Comput.* **2005,** *26* (16), 1752–1780. DOI: 10.1002/jcc.20292.

24. Schrödinger Release 2024-1: *Desmond Molecular Dynamics System*, D. E. Shaw Research, New York, NY,2024. Maestro-Desmond Interoperability Tools, Schrödinger, New York, NY, **2024**.
